# Power Management IC for a Dual-Input-Triple-Output Energy Harvester

**DOI:** 10.3390/mi11100937

**Published:** 2020-10-15

**Authors:** Kai-Meng Mui, Mei-Kum Khaw, Faisal Mohd-Yasin

**Affiliations:** 1 Lee Kong Chian Faculty of Engineering and Science, University Tunku Abdul Rahman, Bandar Sungai Long, Kajang 43000, Selangor, Malaysia; a.k_demen@hotmail.com (K.-M.M.); khawmk@utar.edu.my (M.-K.K.); 2 Queensland Micro- and Nanotechnology Centre, Griffith University, Nathan 4111, Australia

**Keywords:** energy harvesting, piezoelectric, radio frequency, power management unit

## Abstract

We present the design of a power management integrated circuit that processes harvested energy from radio frequency waves and piezoelectric vibrations. The rectification of piezoelectric and RF sources has a power conversion efficiency (PCE) of 87.73% and 74.70%, respectively. The asynchronous and microcontroller-less integrated circuit (IC) is designed to be low power, so the bulk of the harvested energy goes to three loads. The output peak powers of 111 μW, 156 μW, and 128 μW will be sufficient to run small devices for RF communication systems.

## 1. Introduction

The industry has been improving the productivity and efficiency of automated monitoring systems. Instead of favoring the old-fashioned wired system, wireless sensor networks (WSN) have gained attraction [1]. However, they still require a battery or constant power supply to operate continuously and reliably [2]. As replacement batteries are difficult and expensive in access-restrictive areas, research groups have attempted to reduce the dependency on batteries by harvesting energy from the surrounding environments [3,4,5,6,7].

One of the most widely available energy sources is radio frequency (RF) signal. Kim et al. [8] and Visser et al. [9] presented comprehensive reviews on using RF as a primary source to power electronic devices for a variety of applications. Examples include machine-to-machine communications and IoT sensor networks [10], implantable devices [11], and space stations [12]. Several groups have presented different methods for extracting and processing RF energy [13,14,15,16]. However, one could not depend solely on RF waves to harvest energy because of two factors. First, RF signals can only be transmitted periodically or when triggered. Second, RF signals are weakened by distance [17].

Another widely available energy source in nature is mechanical vibrations. We can harvest vibrations by subjecting piezoelectric materials to them. Energy is produced by the deformation of piezoelectric materials, which produces electrical charges. Several review papers cover this topic, and among the most cited are those by Kim et al. [18] and Bowen et al. [19]. They cover progress that is being made in utilizing piezoelectric energy to harvest electrical energy [20,21].

The next evolution in energy harvesting is the integration of multiple input sources, as summarized by Bai et al. [22]. This approach solves the aforementioned problem of relying on a single source, i.e., RF waves. However, it has a tradeoff; that is, the power management unit (PMU) or power management integrated circuits (PMICs) to process the input energy become more complex. Estrada-Lopez et al. [23] presented a review that describes such complexities, as well as a list of selected works that offer solutions. Among these works, three of them combined the RF signal and a piezoelectric source. The first and second groups [24,25] produced a PMIC that was able to harvest energy from three sources: RF, piezoelectric, and photovoltaic. In the third group, Dini et al. [26] added complexities by harvesting from four sources: RF, piezoelectric, photovoltaics, and thermoelectric. Most articles discussing multiple-input energy harvesting schemes were published in the past 10 years. With continuing demands, there is room for improvement; for example, the ability of PMU or PMIC to cater to multiple outputs.

In this paper, we propose a design methodology to produce a PMIC that manages the collection of energy from RF waves and piezoelectric vibration. The stored energy is distributed to three different loads with a peak power ranging from 111 μW to 156 μW. The control unit of the PMIC is based on logic gates and is asynchronous. This low-powered system could be used to power small devices.

## 2. Methods

### 2.1. Description of the Whole System

**Figure 1:**
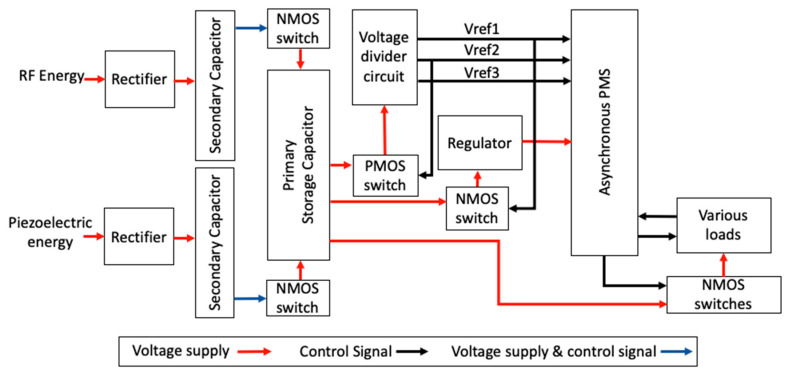
Block diagram of the entire circuit. The input sources are RF and piezoelectric energy. They are first rectified and stored in two storage capacitors. The red lines represent energy that is passed on in the form of a voltage signal. The black lines represent the signals that control the switches or provide status to PMS. The blue lines are both the control signal and voltage supply as the gate and drain of NMOS switches are both tied to the output of the secondary capacitor. From the primary storage, the voltage is sent to the voltage divider, voltage regulator, and loads. The control unit of the system is referred to in the figure as “asynchronous PMS”. The voltage regulator supplies power to the control unit. The voltage divider informs the control unit of the amount of the voltage that is available at the primary storage (in three levels). The NMOS (N-type metal-oxide semiconductor) and PMOS (P-type metal-oxide semiconductor) switches are used to control the flow of the voltage signals.

The block diagram is illustrated in Figure 1, showing all modules from the input sources to the output loads. The RF and vibration voltages were rectified individually before being stored in the secondary capacitors. There are NMOS (N-type metal-oxide semiconductor) switches between the secondary and primary storage methods. These switches ensure that a sufficient level of voltage has been collected by the secondary capacitors before passing it to the primary storage capacitor. At the primary storage, the output voltage is passed to three separate switches that feed voltage divider, regulator, and loads. The voltage divider has three roles. First, it supplies the status of Vref1, Vref2, and Vref3 to the control unit (labeled asynchronous PMS (Power Management System) in Figure 1). Second, it uses Vref2 to control the voltage from the primary storage. This is to avoid energy wastage in the primary storage capacitor. Third, the voltage regulator (via Vref1) also controls the flow of voltage from the primary storage to the voltage regulator. This regulator is used to power the asynchronous PMS. Once the PMS is turned on, it will check for the status of Vref1, Vref2, and Vref3. If all three signals are high at 1.2 V (or equal to logic “1”), the asynchronous PMS will distribute pulsed energy to the loads.

The whole system in Figure 1 was simulated and verified using Synopsys educational design suites (Synopsys, Mountain View, CA, USA), which is distributed by Malaysian distributor, (Intelligent Circuit Engineering Sdn. Bhd, Puchong, Malaysia). We employed Verilog & VHDL Mixed Simulator Design Compiler, and IC Compiler to simulate, synthesize, place, and route the digital design. Custom Compiler was used to complete the schematic and final layout by integrating digital and analog modules. We also used CustomSim to co-simulate the digital and analog designs. The SAED (Synopsys) 90 nm educational process design kit (EPDK) was chosen to implement the IC. A detailed description of each module in Figure 1 is covered from Section 2.1.1, Section 2.1.2, Section 2.1.3, Section 2.1.4, Section 2.1.5, Section 2.1.6, Section 2.1.7 and Section 2.1.8.

#### 2.1.1. RF Waves

RFID technologies are categorized into three bands: low-, high-, and ultra-high-frequency (UHF) [27]. We chose the latter for this work. Here, two examples of studies that harvested UHF signals are given. Vyas et al. [28] harvested ambient Digital TV signals, and Pinuela et al. [29] captured multiple RF sources such as Digital TV, GSM900, GSM1800, 3G, and WiFi. In order to simulate RF signals, the RF voltage source (component name VMRF) was taken from the analoglib that comes with the Synopsys Custom Compiler. There are many parameters in this component that can be modified. The most important are those that describe the signal as sinusoidal, namely voltage amplitude (3.7 V), carrier frequency (865 MHz), and carrier phase (0°). We also set the modulation method as Quadrature Phase Shift Keying. Finally, the data pattern was represented in hexadecimal format and set at ffff0000ffff0000ff00ff00h, with a bit rate of 1 M/s. Finally, Ferdix et al. [30] discussed the impact of harvested RF power with distance from the PMIC. We took this factor into consideration in our simulation, as detailed later in Section 3.4.

#### 2.1.2. Piezoelectric

**Figure 2:**
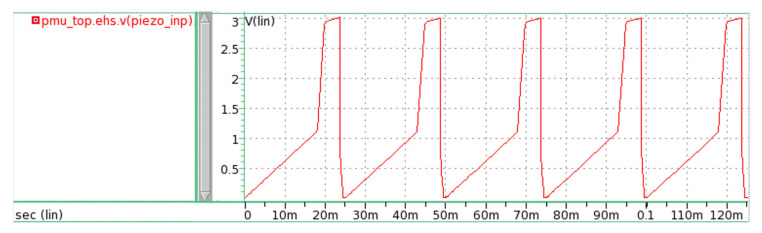
Piezoelectric current input to harvester (pmu_top.ehs.piezo_inp) versus time.

Piezoelectric materials convert vibration or motion into electrical energy. We adopted the piezoelectric input source that was developed by Romani et al. [31]. In their system, the piezoelectric cantilever vibrates at 40 Hz to produce an output voltage of 3 V and current from 8 to 120 μA. In our simulation, this piezoelectric source was modeled using the piecewise linear voltage from analoglib. The simulated input is shown in Figure 2. It produced an average output current of 51 μA and an average output power of 143 μW at a duration of 25 ms.

#### 2.1.3. Rectifier

**Figure 3:**
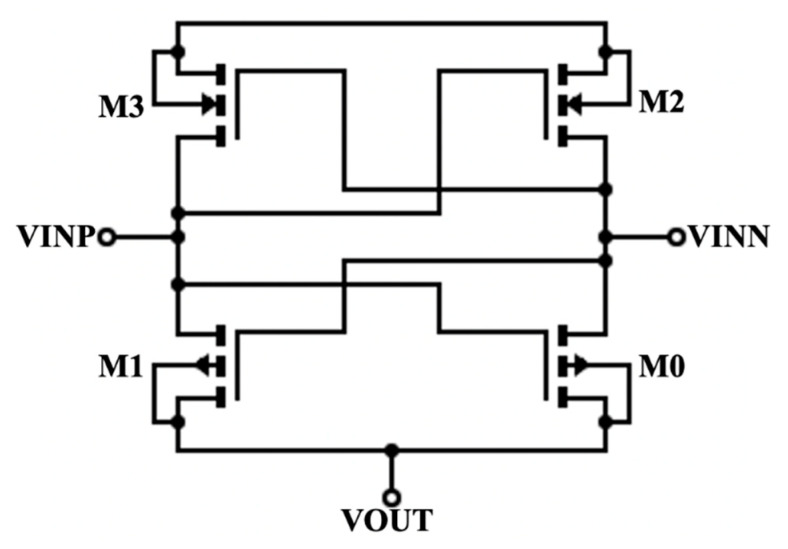
Schematic of the rectifier.

The raw voltage from the input source is rectified to a positive DC voltage. We chose a transistor-based full-wave rectifier to minimize the area, as shown in Figure 3. There are two input nodes, and VINN is the inverse of VINP. When VINP is negative, M2 turns on and M0 turns off. This allows VINN, which has a positive value, to pass through M2 to the VOUT port. When VINN is negative, M3 is on and M1 is off. This allows VINP to pass through the VOUT port. M0 and M1 are PMOS with a W/L ratio of 0.72/0.1, whereas M2 and M3 are NMOS with a W/L ratio of 0.24/0.1.

#### 2.1.4. Energy Storage

Most power management units choose an external battery source or a discreet capacitor to store energy [24]. In our approach, we employed an integrated capacitor to fit all the modules in an IC. It was taken from the EPDK. The area of the capacitors was approximately 90 μm^2^. The values of the primary and secondary capacitors were 194.4 aF and 45.96 aF, respectively.

#### 2.1.5. Voltage Divider

**Figure 4:**
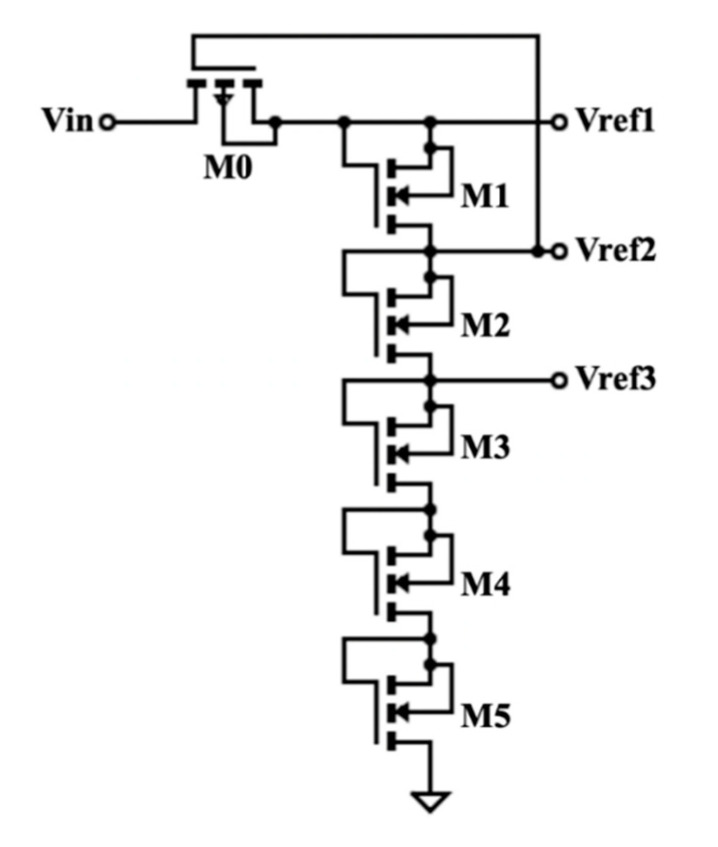
Schematic of voltage divider.

The voltage divider consists of transistors that act as linear resistors, as shown in Figure 4. Vin from the primary storage capacitor is fed to M0. It was controlled by the output Vref2, which had an initial value of zero. In other words, Vin was allowed to flow through when the Vref2 was low, and the former’s value reduced gradually as the latter’s output increased. Vin must be higher than 1.2 V to be recognized as logic “1”. When Vref1, Vref2, and Vref3 are equal to logic “1”, the control unit, i.e., asynchronous PMS, will commence operation. The output voltage across each transistor can be expressed as:

(1) $$V_{R\left(n\right)}=V_{ref1}\left(\frac{R_{n}}{R_{T}}\right)$$

where *V_R_*_(*n*)_ is the voltage drop across the transistor, *R_n_* is the resistance value across transistor, and *R_T_* is the total resistance.

#### 2.1.6. Voltage Regulator

**Figure 5:**
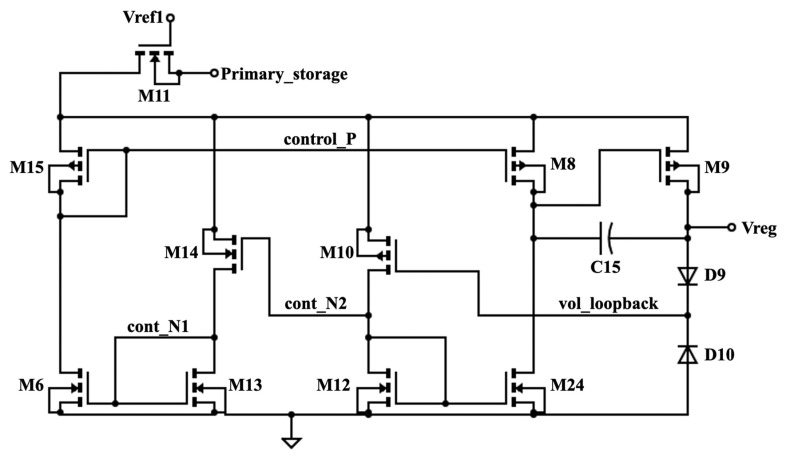
Schematic of the voltage regulator.

The voltage regulator provides a stable voltage to power the control unit. Its schematic is shown in Figure 5. The voltage from the primary storage capacitor is supplied through a NMOS transistor switch (M11) to be connected to M8, M9, M10, M14, and M15. This switch is controlled by Vref1 from the voltage divider (Figure 4). The node “control_P” has an initial value of 0 V to turn on M15 and M8. Because M15 has its gate and drain shorted, the voltage flow through this transistor and M8 will be limited. The same mechanism occurs at M10, where the node “vol_loopback” of 0 V turns on M10. In turn, this allows the voltage to rise at the node “cont_N2”. This node then turns on M14, which allows the voltage to flow through the node “cont_N1”. As a result, M13 drains the voltage to the ground. At the same time, the voltage at the node “control_P” drains through M6. This causes a voltage drop in this node, causing a “push and pull” tug of war to occur among the transistors. In this case, if the voltage from the primary storage capacitor is low, control_P will be low as well, allowing most of this input voltage to pass through M8. The drain of M8 is connected to the gate of M9. Hence, if the voltage that passes through M8 is high, it would turn off M9. There are three nontransistor components in the schematic of Figure 5. First, capacitor C15 is connected to the drains of M8 and M9 to increase the output voltage. Second, diode D9 allows a small amount of feedback voltage from the node “vol_loopback” to the node “cont_N2” to limit the amount of voltage that can pass through M8. Third, diode D10 blocks the voltage from flowing to the ground.

#### 2.1.7. Control Unit (Asynchronous PMS)

**Figure 6:**
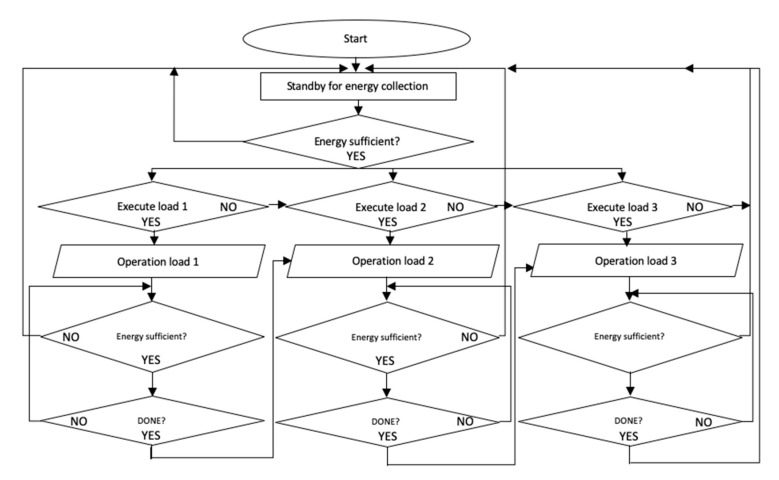
PMS flow chart diagram.

This is the only digital part of the power management IC. The main function is to feed the three loads. In order to reduce power consumption, we used logic gates instead of a microcontroller. In our logic, the distribution of voltage to the three loads was prewired to avoid the use of a clock. Before the operation commenced, inputs from the voltage divider allowed the control unit to determine the sufficiency of energy in storage. Vref1, Vref2, and Vref3 represent the amplitude of the voltage at low, medium, and high levels, respectively. They must be at least 1.2 V to turn on the corresponding loads. The flow chart is shown in Figure 6. The Verilog code of the control unit is presented in the Supplementary Materials.

#### 2.1.8. Loads

There is a wide choice of loads, ranging from a computing unit such as a microprocessor unit to general sensors like temperature and humidity sensors. We have proposed three loads to power the following chips: (1) a RF transceiver [32] that consumes 98 μW during transmission, (2) a low-power asynchronous 8051 microcontroller [33] that consumes an average power of 70 μW, and a low-power AES [34] that consumes 0.4 μW/MHz with a maximum frequency of 130.9 MHz. Table 1 summarizes these chosen loads and their power consumption. When all three chips are powered simultaneously, they can be used for data transmission with encryption.

The load circuits consist of resistors, capacitors, and buffers. The buffers are used to increase the delay of the return signal to the control unit to indicate that the operation has finished. The values of the resistors are 255 kOhms, 315 kOhms, and 305 kOhms, which are paired with the capacitors at 0.37 fF, 90 fF, and 100 aF for Loads 1, 2, and 3, respectively. In practice, the applications of the loads can be changed as long as they can be operated within the harvested voltage level.

## 3. Results and Discussion

**Figure 7:**
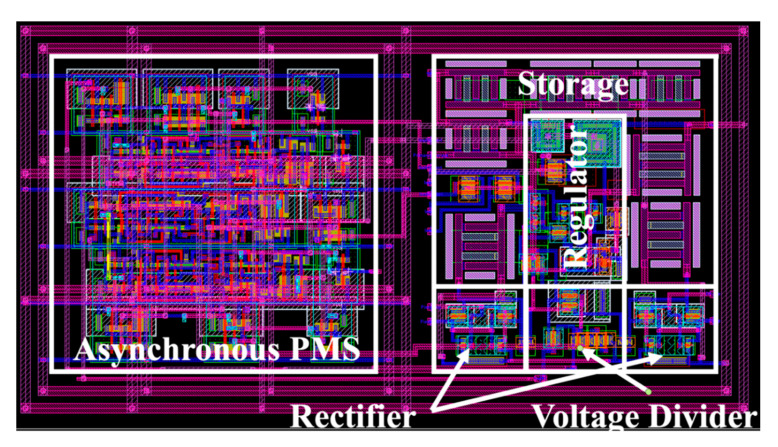
Layout of the entire circuitry.

The design was separated into digital and analog parts. The digital part consisted of the control unit. The rest of the circuits formed the analog block. For this power management IC, we implemented the Synopsys Mixed-Signal design flow. The layout of the entire circuit is shown in Figure 7. More than half of the total area was consumed by the logic gates (labeled “asynchronous PMS” in the figure), which were generated from Verilog. The rest of the IC consisted of the analog modules and the interconnects.

### 3.1. Analog Circuits

**Figure 8:**
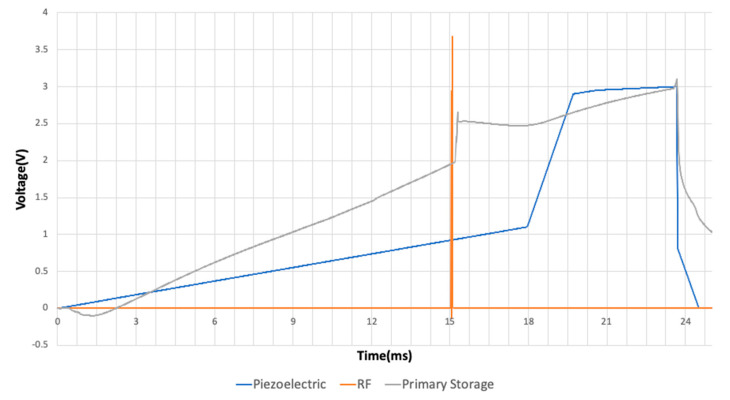
Transient characteristics of the Piezoelectric input, RF input, and accumulated voltage at the primary storage capacitor.

The analog circuit consisted of rectifiers, voltage dividers, voltage regulators, and switches. The input source of the RF and piezoelectric were randomized to mimic a real environment. Figure 8 shows the original voltages of both sources. They were rectified individually to get positive voltages at the input of the secondary capacitor. The rectification of the RF and piezoelectric sources had a power conversion efficiency (PCE) of 87.73 and 74.70%, respectively. The calculation was performed based on Equation (2):

(2) $$PCE=\frac{P_{rectified}}{P_{received}}*\;100\%$$

**Figure 9:**
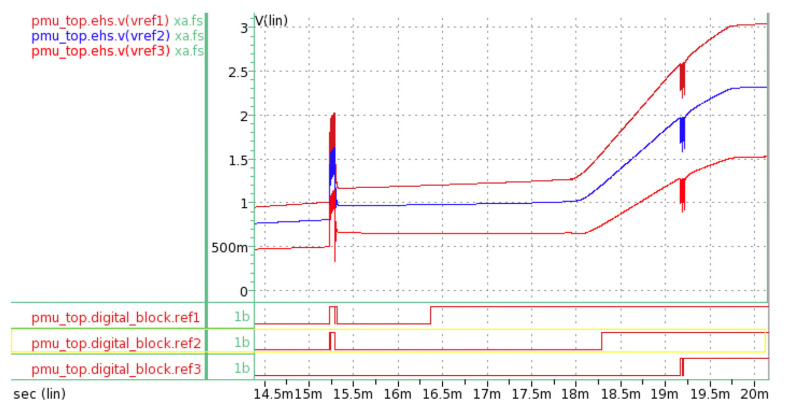
Plot of Vref1 (brown color), Vref2 (blue color), and Vref 3 (red color) in the analog domain with corresponding signals in the digital domain. As the voltage passed 1.2 V, Vref1, Vref2 and Vref3 caused a transition from logic “0” to logic “1” at the digital domain. The commencement of the Load 1 operation caused a momentary voltage drop because the power source for the voltage divider, regulator, and control unit was at the same node.

The rectified voltage flowed to the voltage divider, regulator, and loads. Figure 9 shows the timing graphs at the voltage divider. As stated in Section 2.1.7, in order to activate the control unit, the values for Vref1, Vref2, and Vref3 must be equal to or higher than 1.2 V. Since this condition was met, the logic levels turned to high (“1”), which commenced operation for the control unit.

**Figure 10:**
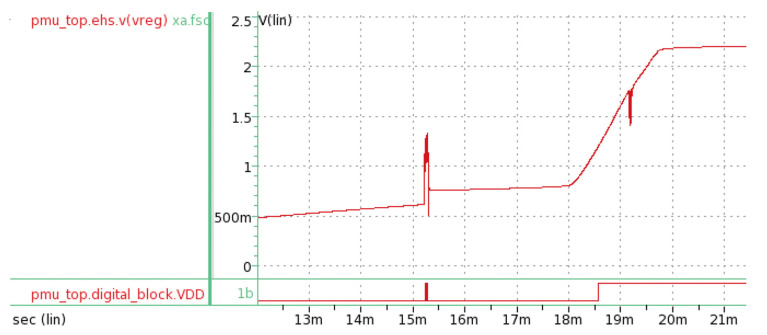
Plot of v_reg_ in both the digital and analog domains. The global operating voltage was set at 1.2 V.

Figure 10 shows the simulation result at the voltage regulator. The operating voltage for the control unit was set at 1.2 V, since we used SAED 90 nm EPDK.

### 3.2. Control Unit

**Figure 11:**
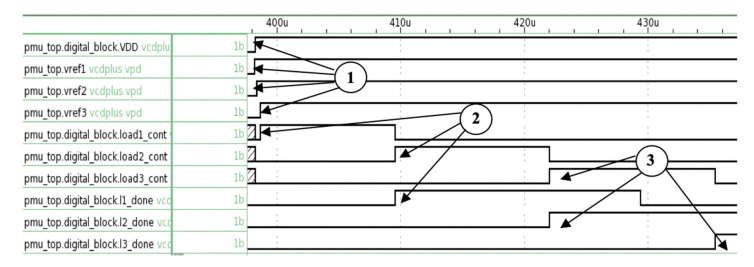
Timing diagram for the control unit for the scenario where sufficient energy was harvested for the three loads.

Figure 11 shows the timing diagram for the operation of the control unit. At Mark 1, the unit is powered up with VDD, and Vref1, Vref2, and Vref3 are designated as high. At Mark 2, PMS transmits load1_cont signal to activate Load 1. After 100 ns, l1_done signal is transmitted to end operation for that load. At the same time, the operation for Load 2 commences. This step is repeated at Mark 3 for Load 3. Once completed, the control unit waits for energy to be harvested to repeat the process.

**Figure 12:**
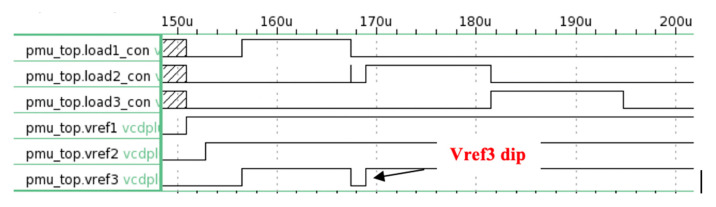
The timing graph shows Vref3 status changes from logic “1” to logic “0” when there is insufficient energy being harvested. As a result, the control unit drops the operation of Load 2. Once Vref3 regains logic “1”, Load 2 operations resume.

Figure 11 shows the “successful” operation when the primary storage capacitor has sufficient energy to power all three loads. There are instances when the available energy at the primary storage capacitor is not sufficient to power all the loads. Figure 12 depicts this scenario. In Figure 12, Vref3 drops below 0.9 V and indicates logic “0”. As a result, the control unit shuts down the operation of Load 2 to preserve energy. When Vref3 rises above 1.2 V and gives logic “1” again, the control unit sends the logic “1” signal to Load 2.

### 3.3. Power Generation and Consumption

**Figure 13:**
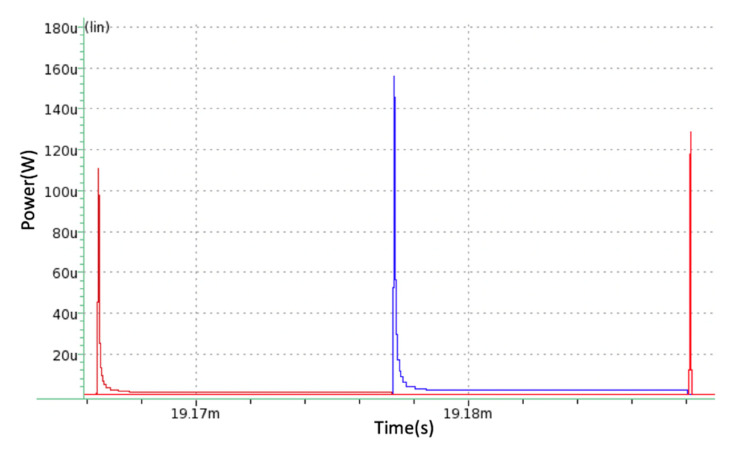
Peak power of Load 1 (brown), Load 2 (blue), and Load 3 (red). Their peak powers are 111 μW, 156 μW, and 129 μW, respectively.

Figure 13 shows the output power of all three loads. Each is activated by the control unit at different times with a delay of approximately 100 ns. The peak powers for Loads 1, 2, and 3 are 111 μW, 156 μW, and 129 μW, respectively. As stated in Section 2.1.8, these power values are more than sufficient to power the three ICs that make up of an encrypted RF communication system.

The power management IC only consumes a total of 12 μW during active operation, where the digital and analog blocks use 2.2 μW and 9.87 μW, respectively. In comparison to the total peak powers that are outputted to the three loads, the total consumption of the power management IC is very minimal, demonstrating the efficiency of this design.

### 3.4. Power Generation at Varying Distance of RF Source

One practical issue that needs to be tackled is the varying distance of the RF source from the PMIC. Simply put, the further the distance, the lower the power that can be harvested. We used the data of generated RF power at four distances (2.5 m, 5 m, 7.5 m, and 10 m) from Ferdik et al. [30]. The second row of Table 2 displays the generated RF power at the input node, while the third row shows the generated RF power after rectification. Finally, we must also deduct the power consumption by the PMIC, which is 12 μW. Hence, the fourth row shows the generated RF power that is available to the loads. From Table 1, the total amount of power consumption that is needed by these loads is 220 μW. It is clear from the data that if the distance between the RF input and PMIC is 2.5 m, the generated RF power of 477 μW is more than enough to single-handedly power the whole operation.

However, at a distance of 5 m, the generated RF power drops rapidly, and help from the piezoelectric input is necessary to supplement the remaining balance. We modeled our piezoelectric source from Romani et al. [31] and found that the maximum generated power from a single cantilever is 143.4 μW. This amount is sufficient to supplement the generated amount of RF power of 110 μW at a distance of 5 m to power the three loads. This information is reflected in Rows 5 and 6 of Table 2.

At a distance of 7.5 m and 10 m, the generated RF power drops further. In both cases, the generated piezoelectric power of 143 μW from a single cantilever plus the generated RF power are not sufficient to power the loads. The best solution is to employ two piezo inputs in parallel to double the amount of their generated power.

The last point is as follows. At a sufficiently long distance, the amount of generated power from both the RF and piezoelectric sources will not be sufficient to power the loads. In this case, our PMIC will activate the scenario in Figure 12, where it will wait until the next sufficient power input to continue the operation.

## 4. Conclusions

The demand for so-called green electronics will continue to rise in the future. Many researchers have proposed the harvesting of energy that is abundantly available, such as RF waves and piezoelectric. The purpose of this paper was to come up with a design methodology for power management IC that is able to process both sources efficiently, and channel the outputs to three different loads that could power small devices for RF communication. We achieved this objective while minimizing the power consumption of the IC.

## Figures and Tables

**Table 1:** Power consumption for the respective loads and the total power consumption of loads.

Loads	Power Consumption (μW)
AES	52.36
MCU	70
RF Transceiver	98
Total power consumption	220.36

**Table 2:** Power calculation based on RF input power from four distances and a minimum amount of piezoelectric input power needed to complete the whole operation.

Distance	2.5 m	5 m	7.5 m	10 m
RF input power (μW)	654.7	163.7	72.7	40.9
Generated power after rectifier (μW)	489.0609	122.2839	54.3069	30.5523
Generated power after deducting power consumption by PMIC (μW)	476.9909	110.2139	42.2369	18.4823
Minimum amount of power from piezoelectric input that is needed to supplement power from RF input (μW)	0	122.2161	190.1931	213.9477
Min. number of Piezoelectric input source (times)	0	1	2	2
Piezoelectric input power (μW)	0	143.4	286.8	286.8
Piezoelectric input power after rectifier (μW)	0	125.80	251.60	251.60

## Supplementary Materials

The following are available online at https://www.mdpi.com/2072-666X/11/10/937/s1, PMS Verilog Code: PMS.v.
