# Supplementary material for: Power Management IC for a Dual-Input-Triple-Output Energy Harvester
Source: Micromachines (Basel). 2020 Oct 15;11(10):937. doi: 10.3390/mi11100937 (PMC7602376; doi:10.3390/mi11100937)
Supplement: Supplementary file 1 [file micromachines-11-00937-s001.pdf]

```
*Analogue block
*  Generated for: HSPICE
*  Design library name: EHS
*  Design cell name: EHS_tb
*  Design view name: config
.option search='/home/Anthony/EHS_saed90/SAED_PDK90nm/hspice'


.option PARHIER = LOCAL
.option PORT_VOLTAGE_SCALE_TO_2X = 1

.option WDF=1
.temp 25
.lib 'SAED90nm.lib' TT_12
.lib 'SAED90nm.lib' TT_12_HVT
.lib 'SAED90nm.lib' TT_12_LVT
.lib
'/home/Anthony/silterra_pdk/models/hspice/c18_core18_io33_header_hspice.modlib
' c18_hp_tt
.lib
'/home/Anthony/silterra_pdk/models/hspice/c18_core18_io33_header_hspice.modlib
' c18_finger_cap_tt
*Custom Compiler Version P-2019.06-4
*Mon Apr 27 19:53:28 2020

.global 0 gnd!
********************************************************************************
* Library          : Rectifier0
* Cell             : Piezo_input1
* View             : schematic
* View Search List : schematic symbol hspice
* View Stop List   : symbol
********************************************************************************
.subckt piezo_input1 pi_out_n pi_out_p
c4 pi_out_p gnd! c=400p
c5 pi_out_n gnd! c=400p
c4a pi_out_p gnd! c=800p
c5a pi_out_n gnd! c=800p
c4b pi_out_p gnd! c=800p
c5b pi_out_n gnd! c=800p
v1 pi_out_p pi_out_n pwl ( 0 0.0 17.95m 2.2 19.7m 5.8 20.6m 5.9 23.65m 6.0 23.7m 1.6
24.5m 0.0 25m 0.0
+ r=0.0 td=0u )
```

```
.ends piezo_input1

************************************************************************
* Library          : Rectifier
* Cell             : load_3f
* View             : schematic
* View Search List : schematic symbol hspice
* View Stop List   : symbol
************************************************************************
.subckt load_3f vsupply lm_done
r33 net74 net73 r=2180k
r32 net72 net71 r=2180k
r31 net67 net68 r=2180k
r0 vsupply gnd! r=305k
xc29 net73 gnd! gnd! fm3m1 sp_length=4.22232m m=1
xc19 lm_done gnd! gnd! fm3m1 sp_length=4.22232m m=1
xc24 net55 gnd! gnd! fm3m1 sp_length=4.22232m m=1
xc25 net68 gnd! gnd! fm3m1 sp_length=4.22232m m=1
xc27 net71 gnd! gnd! fm3m1 sp_length=4.22232m m=1
xc1 net71a gnd! ccap w=0.900324 l=0.94u nf=4 startmetal=1 endmetal=2
xc2 net71a gnd! ccap w=0.900324 l=0.94u nf=4 startmetal=1 endmetal=2
xc3 net71a gnd! ccap w=0.900324 l=0.94u nf=4 startmetal=1 endmetal=2
xc4 net71a gnd! ccap w=0.900324 l=0.94u nf=4 startmetal=1 endmetal=2
xc33 vsupply gnd! ccap w=0.300324 l=2u nf=4 startmetal=1 endmetal=2

m15 lm_done net73 gnd! gnd! n12_lvt w=0.23u l=0.1u nf=1.0 m=1 ad=0.3456n
as=0.35904n
+  pd=1.30160m ps=1.36644m nrd=0.3375 nrs=0.350625 sa=0.48u sb=0.48u sd=0.54u
m13 net74 net71a gnd! gnd! n12_lvt w=0.23u l=0.1u nf=1.0 m=1 ad=0.3456n
as=0.35904n
+  pd=1.30160m ps=1.36644m nrd=0.3375 nrs=0.350625 sa=0.48u sb=0.48u sd=0.54u

m15a net71a net74a gnd! gnd! n12_lvt w=0.23u l=0.1u nf=1.0 m=1 ad=0.3456n
as=0.35904n
+  pd=1.30160m ps=1.36644m nrd=0.3375 nrs=0.350625 sa=0.48u sb=0.48u sd=0.54u
m13a net74a net71 gnd! gnd! n12_lvt w=0.23u l=0.1u nf=1.0 m=1 ad=0.3456n
as=0.35904n
+  pd=1.30160m ps=1.36644m nrd=0.3375 nrs=0.350625 sa=0.48u sb=0.48u sd=0.54u

m11 net72 net68 gnd! gnd! n12_lvt w=0.23u l=0.1u nf=1.0 m=1 ad=0.3456n
as=0.35904n
+  pd=1.30160m ps=1.36644m nrd=0.3375 nrs=0.350625 sa=0.48u sb=0.48u sd=0.54u
m9 net67 net55 gnd! gnd! n12_lvt w=0.23u l=0.1u nf=1.0 m=1 ad=0.3456n as=0.35904n
```

```
+  pd=1.30160m ps=1.36644m nrd=0.3375 nrs=0.350625 sa=0.48u sb=0.48u sd=0.54u
m3 net55 net13 gnd! gnd! n12_lvt w=0.23u l=0.1u nf=1.0 m=1 ad=0.3456n as=0.35904n
+  pd=1.30160m ps=1.36644m nrd=0.3375 nrs=0.350625 sa=0.48u sb=0.48u sd=0.54u
m141 net13 vsupply gnd! gnd! n12_lvt w=0.23u l=0.1u nf=1.0 m=1 ad=0.3456n
as=0.35904n
+  pd=1.30160m ps=1.36644m nrd=0.3375 nrs=0.350625 sa=0.48u sb=0.48u sd=0.54u
v23 net86 gnd! dc=1.2
m14 lm_done net73 net86 net86 p12_hvt w=0.23u l=0.1u nf=1.0 m=1 ad=0.108n
as=0.1164n
+  pd=0.410800m ps=0.451640m nrd=0.27 nrs=0.291 sa=0.48u sb=0.48u sd=0.54u
m12 net74 net71a net86 net86 p12_hvt w=0.23u l=0.1u nf=1.0 m=1 ad=0.3456n
as=0.35904n
+  pd=1.30160m ps=1.36644m nrd=0.3375 nrs=0.350625 sa=0.48u sb=0.48u sd=0.54u

m14a net71a net74a net86 net86 p12_hvt w=0.23u l=0.1u nf=1.0 m=1 ad=0.108n
as=0.1164n
+  pd=0.410800m ps=0.451640m nrd=0.27 nrs=0.291 sa=0.48u sb=0.48u sd=0.54u
m12a net74a net71 net86 net86 p12_hvt w=0.23u l=0.1u nf=1.0 m=1 ad=0.3456n
as=0.35904n
+  pd=1.30160m ps=1.36644m nrd=0.3375 nrs=0.350625 sa=0.48u sb=0.48u sd=0.54u

m10 net72 net68 net86 net86 p12_hvt w=0.23u l=0.1u nf=1.0 m=1 ad=0.3456n
as=0.35904n
+  pd=1.30160m ps=1.36644m nrd=0.3375 nrs=0.350625 sa=0.48u sb=0.48u sd=0.54u
m8 net67 net55 net86 net86 p12_hvt w=0.23u l=0.1u nf=1.0 m=1 ad=0.3456n
as=0.35904n
+  pd=1.30160m ps=1.36644m nrd=0.3375 nrs=0.350625 sa=0.48u sb=0.48u sd=0.54u
m2 net55 net13 vsupply vsupply p12_hvt w=0.23u l=0.1u nf=1.0 m=1 ad=0.3456n
as=0.35904n
+  pd=1.30160m ps=1.36644m nrd=0.3375 nrs=0.350625 sa=0.48u sb=0.48u sd=0.54u
m140 net13 vsupply vsupply vsupply p12_hvt w=0.23u l=0.1u nf=1.0 m=1
ad=0.3456n
+ as=0.35904n pd=1.30160m ps=1.36644m nrd=0.3375 nrs=0.350625 sa=0.48u sb=0.48u
+ sd=0.54u

.ends load_3f

************************************************************************
* Library          : EHS
* Cell             : EHS_ip
* View             : starrc
* View Search List : schematic symbol hspice
* View Stop List   : symbol
```

```
******************************************************************************
.subckt ehs_ip vref1 vref2 vref3 load1_cont load2_cont load3_cont l1done l2done
l3done vreg

r147775 gnd! gnd1 r=0
x8 rf_inn rf_inp rf_input
xi15 load1 l1done load_3c
xi23 load2 l2done load_3c
xi21 load3 l3done load_3f

cg3873 1196:net43 0  c=1.14285e-17
c3872 1196:net43 108:ln_n52  c=1.38106e-17
c3871 1196:net43 m9|gate  c=1.47995e-18
c3870 1196:net43 96:ln_n52  c=2.47309e-18
c3869 1196:net43 c15|plus  c=1.80805e-18
c3868 1196:net43 1129:vol_loopback  c=1.68396e-19
c3867 1196:net43 1088:cont_n3  c=1.20757e-17
c3866 1196:net43 1126:vol_loopback  c=4.31763e-18
c3865 1196:net43 1120:cont_n3  c=1.91539e-18
c3864 1196:net43 1087:cont_n3  c=2.36543e-19
c3863 1196:net43 1:ln_n124  c=4.21612e-18
cg3862 1195:net43 0  c=3.68339e-18
c3861 1195:net43 108:ln_n52  c=1.36099e-17
c3860 1195:net43 m10|gate  c=1.2897e-18
c3859 1195:net43 1167:control_p  c=1.28867e-17
c3858 1195:net43 96:ln_n52  c=4.65081e-18
c3857 1195:net43 1166:control_p  c=1.63064e-18
c3856 1195:net43 1154:control_p  c=1.31968e-17
c3855 1195:net43 1084:cont_n3  c=1.06528e-18
c3854 1195:net43 1145:vol_loopback  c=4.72455e-19
c3853 1195:net43 1137:vol_loopback  c=3.48036e-17
c3852 1195:net43 1129:vol_loopback  c=9.80159e-18
c3851 1195:net43 1060:primary_storage  c=1.65711e-20
c3850 1195:net43 1061:primary_storage  c=1.42253e-18
c3849 1195:net43 1126:vol_loopback  c=2.30767e-18
c3848 1195:net43 1165:control_p  c=1.33621e-20
c3847 1195:net43 m11|gate  c=5.61082e-18
c3846 1195:net43 1:ln_n124  c=1.99581e-17
cg3845 1194:net43 0  c=9.96454e-18
c3844 1194:net43 108:ln_n52  c=9.85038e-17
c3843 1194:net43 m10|gate  c=7.95525e-19
c3842 1194:net43 1167:control_p  c=3.0574e-20
c3841 1194:net43 m10|drn  c=5.16569e-19
```

c3840 1194:net43 m9|gate c=2.52999e-18
c3839 1194:net43 96:ln_n52 c=1.5222e-17
c3838 1194:net43 1084:cont_n3 c=1.25074e-18
c3837 1194:net43 531:cont_n2 c=2.85746e-18
c3836 1194:net43 m14|gate c=5.6164e-21
c3835 1194:net43 1145:vol_loopback c=5.59497e-18
c3834 1194:net43 521:cont_n2 c=1.33471e-18
c3833 1194:net43 1137:vol_loopback c=7.6104e-18
c3832 1194:net43 1129:vol_loopback c=1.26657e-17
c3831 1194:net43 1088:cont_n3 c=2.07487e-17
c3830 1194:net43 1126:vol_loopback c=1.62549e-17
c3829 1194:net43 1119:cont_n3 c=4.08777e-19
c3828 1194:net43 1120:cont_n3 c=8.1941e-20
c3827 1194:net43 1087:cont_n3 c=2.88586e-19
c3826 1194:net43 1:ln_n124 c=2.2123e-17
cg3825 1193:net43 0 c=1.2785e-17
c3824 1193:net43 108:ln_n52 c=1.425e-17
c3823 1193:net43 1167:control_p c=5.06826e-18
c3822 1193:net43 96:ln_n52 c=6.67799e-18
c3821 1193:net43 1166:control_p c=6.82072e-18
c3820 1193:net43 1084:cont_n3 c=1.25732e-18
c3819 1193:net43 1154:control_p c=5.98218e-18
c3818 1193:net43 m20|drn c=5.74819e-19
c3817 1193:net43 1150:control_p c=2.35088e-19
c3816 1193:net43 1137:vol_loopback c=3.06494e-19
c3815 1193:net43 1060:primary_storage c=6.96003e-19
c3814 1193:net43 1061:primary_storage c=7.89076e-18
c3813 1193:net43 334:vref1 c=8.39243e-19
c3812 1193:net43 1148:control_p c=4.73231e-19
c3811 1193:net43 1116:cont_n3 c=4.42173e-20
c3810 1193:net43 1117:cont_n3 c=2.43195e-19
c3809 1193:net43 m11|gate c=2.08953e-18
c3808 1193:net43 1:ln_n124 c=2.72188e-17
c3807 1193:net43 m11|drn c=6.51814e-19
cg3806 1192:net43 0 c=7.25498e-19
c3805 1192:net43 m10|drn c=6.09551e-20
c3804 1192:net43 1084:cont_n3 c=6.78297e-19
c3803 1192:net43 531:cont_n2 c=9.27869e-18
c3802 1192:net43 m8|gate c=1.46714e-18
c3801 1192:net43 1150:control_p c=3.01789e-19
c3800 1192:net43 m14|gate c=1.69413e-18
c3799 1192:net43 521:cont_n2 c=2.08198e-17
c3798 1192:net43 523:cont_n2 c=1.21881e-18

c3797 1192:net43 998:primary_storage c=2.42444e-18
c3796 1192:net43 1148:control_p c=8.03629e-19
c3795 1192:net43 159:ln_n60 c=3.10634e-20
c3794 1192:net43 1165:control_p c=1.38409e-17
c3793 1192:net43 m15|gate c=3.50395e-21
c3792 1192:net43 1:ln_n124 c=8.75554e-18
cg3791 1191:net43 0 c=1.28904e-17
c3790 1191:net43 539:cont_n1 c=7.76495e-17
c3789 1191:net43 1168:control_p c=2.04816e-17
c3788 1191:net43 147:ln_n60 c=3.02644e-18
c3787 1191:net43 1007:primary_storage c=1.42493e-18
c3786 1191:net43 m6|gate c=1.66312e-18
c3785 1191:net43 1152:control_p c=2.13239e-17
c3784 1191:net43 352:vref1 c=2.01781e-20
c3783 1191:net43 1150:control_p c=1.05873e-17
c3782 1191:net43 m14|gate c=6.78611e-18
c3781 1191:net43 533:cont_n1 c=1.80025e-18
c3780 1191:net43 521:cont_n2 c=1.87126e-17
c3779 1191:net43 998:primary_storage c=2.25485e-17
c3778 1191:net43 334:vref1 c=1.48477e-18
c3777 1191:net43 1148:control_p c=4.82722e-20
c3776 1191:net43 350:vref1 c=5.77973e-17
c3775 1191:net43 159:ln_n60 c=1.86601e-17
c3774 1191:net43 1165:control_p c=2.93323e-19
c3773 1191:net43 m15|gate c=3.79096e-18
c3772 1191:net43 1:ln_n124 c=3.67057e-17
cg3771 1190:net43 0 c=5.84108e-19
c3770 1190:net43 m10|gate c=1.08089e-19
c3769 1190:net43 m10|drn c=3.41053e-20
c3768 1190:net43 1084:cont_n3 c=2.32575e-18
c3767 1190:net43 531:cont_n2 c=1.00678e-17
c3766 1190:net43 521:cont_n2 c=1.23284e-18
c3765 1190:net43 998:primary_storage c=1.01684e-18
c3764 1190:net43 334:vref1 c=1.34368e-18
c3763 1190:net43 350:vref1 c=9.54457e-18
c3762 1190:net43 159:ln_n60 c=3.00286e-21
c3761 1190:net43 m15|gate c=1.39766e-19
c3760 1190:net43 1:ln_n124 c=6.67292e-18
cg3759 1189:net43 0 c=2.41647e-18
c3758 1189:net43 1030:primary_storage c=2.12581e-17
c3757 1189:net43 1166:control_p c=2.38048e-19
c3756 1189:net43 1007:primary_storage c=5.39351e-17
c3755 1189:net43 1154:control_p c=9.52194e-20

c3754 1189:net43 m8|gate c=4.28087e-19
c3753 1189:net43 1150:control_p c=2.28138e-17
c3752 1189:net43 996:primary_storage c=1.0441e-17
c3751 1189:net43 998:primary_storage c=7.69196e-18
c3750 1189:net43 335:vref1 c=1.30557e-20
c3749 1189:net43 334:vref1 c=4.93626e-17
c3748 1189:net43 1148:control_p c=7.76367e-19
c3747 1189:net43 350:vref1 c=3.25924e-17
c3746 1189:net43 m15|gate c=5.74287e-18
c3745 1189:net43 1165:control_p c=2.61318e-18
c3744 1189:net43 1:ln_n124 c=2.54058e-17
cg3743 1188:net43 0 c=2.06694e-17
c3742 1188:net43 1062:primary_storage c=5.94351e-19
c3741 1188:net43 1058:primary_storage c=6.2563e-19
c3740 1188:net43 1055:primary_storage c=6.64844e-18
c3739 1188:net43 1030:primary_storage c=2.83971e-17
c3738 1188:net43 1007:primary_storage c=1.21357e-18
c3737 1188:net43 1059:primary_storage c=5.69054e-19
c3736 1188:net43 1009:primary_storage c=8.0744e-20
c3735 1188:net43 1150:control_p c=3.36909e-18
c3734 1188:net43 996:primary_storage c=2.07173e-17
c3733 1188:net43 997:primary_storage c=1.16241e-17
c3732 1188:net43 335:vref1 c=1.20673e-18
c3731 1188:net43 334:vref1 c=1.00962e-17
c3730 1188:net43 350:vref1 c=3.8353e-19
c3729 1188:net43 m5@2|src c=2.68576e-19
c3728 1188:net43 m7|drn c=1.80283e-19
c3727 1188:net43 m15|gate c=1.28967e-18
c3726 1188:net43 m15|src c=3.98402e-19
c3725 1188:net43 1:ln_n124 c=3.31943e-17
cg3724 m10|src 0 c=1.07903e-20
c3723 m10|src 1011:primary_storage c=8.86369e-20
c3722 m10|src m10|gate c=7.0969e-18
c3721 m10|src 1167:control_p c=2.76075e-19
c3720 m10|src m8|gate c=5.94036e-20
c3719 m10|src 1137:vol_loopback c=3.27989e-19
c3718 m10|src 1129:vol_loopback c=3.03681e-19
c3717 m10|src m15|gate c=9.62201e-20
c3716 m10|src m11|gate c=3.07613e-18
c3715 m10|src 1:ln_n124 c=1.17258e-16
cg3714 1183:net43 0 c=7.15653e-19
c3713 1183:net43 539:cont_n1 c=1.72204e-18
c3712 1183:net43 m10|drn c=2.45442e-20

c3711 1183:net43 1084:cont_n3 c=1.02983e-18
c3710 1183:net43 531:cont_n2 c=5.4665e-19
c3709 1183:net43 1150:control_p c=1.48417e-19
c3708 1183:net43 521:cont_n2 c=6.30339e-18
c3707 1183:net43 998:primary_storage c=2.43352e-18
c3706 1183:net43 159:ln_n60 c=4.92131e-20
c3705 1183:net43 1165:control_p c=1.3522e-18
c3704 1183:net43 1:ln_n124 c=7.16684e-18
cg3703 1177:net43 0 c=3.25461e-18
c3702 1177:net43 108:ln_n52 c=1.86057e-18
c3701 1177:net43 m9|gate c=6.15626e-19
c3700 1177:net43 1166:control_p c=9.87381e-19
c3699 1177:net43 1154:control_p c=6.42477e-19
c3698 1177:net43 m20|drn c=2.10046e-19
c3697 1177:net43 m8|drn c=2.7963e-19
c3696 1177:net43 1061:primary_storage c=1.60195e-18
c3695 1177:net43 334:vref1 c=1.82012e-20
c3694 1177:net43 1148:control_p c=2.92235e-19
c3693 1177:net43 m15|gate c=2.94095e-20
c3692 1177:net43 m11|gate c=4.87308e-18
c3691 1177:net43 1:ln_n124 c=2.62586e-16
c3690 1177:net43 m11|drn c=3.60133e-18
cg3689 1176:net43 0 c=3.71044e-17
c3688 1176:net43 108:ln_n52 c=2.65154e-17
c3687 1176:net43 m9|gate c=3.74262e-18
c3686 1176:net43 96:ln_n52 c=6.08003e-18
c3685 1176:net43 c15|plus c=3.05959e-18
c3684 1176:net43 1088:cont_n3 c=5.59082e-19
c3683 1176:net43 1126:vol_loopback c=1.25779e-17
c3682 1176:net43 1120:cont_n3 c=5.76215e-18
c3681 1176:net43 1087:cont_n3 c=1.81631e-19
c3680 1176:net43 1115:cont_n3 c=6.05704e-19
c3679 1176:net43 1:ln_n124 c=9.68927e-18
c3678 1176:net43 1118:cont_n3 c=4.24162e-20
c3677 1175:net43 m10|gate c=2.74068e-19
c3676 1175:net43 1167:control_p c=1.93955e-17
c3675 1175:net43 1166:control_p c=6.66812e-18
c3674 1175:net43 1084:cont_n3 c=1.33641e-17
c3673 1175:net43 1154:control_p c=7.2287e-19
c3672 1175:net43 531:cont_n2 c=1.49986e-17
c3671 1175:net43 m8|gate c=6.00756e-18
c3670 1175:net43 521:cont_n2 c=5.70356e-19
c3669 1175:net43 1137:vol_loopback c=2.40514e-18

c3668 1175:net43 1129:vol_loopback c=3.38467e-18
c3667 1175:net43 998:primary_storage c=9.8534e-19
c3666 1175:net43 334:vref1 c=3.55849e-18
c3665 1175:net43 1126:vol_loopback c=1.61811e-18
c3664 1175:net43 1117:cont_n3 c=6.28199e-17
c3663 1175:net43 m15|gate c=1.64403e-19
c3662 1175:net43 1165:control_p c=1.79475e-17
c3661 1175:net43 m11|gate c=1.25284e-18
c3660 1175:net43 1:ln_n124 c=8.90088e-18
c3659 1174:net43 108:ln_n52 c=4.8135e-20
c3658 1174:net43 m10|gate c=4.52654e-18
c3657 1174:net43 1167:control_p c=2.44231e-18
c3656 1174:net43 96:ln_n52 c=6.86145e-20
c3655 1174:net43 1154:control_p c=1.32185e-18
c3654 1174:net43 1084:cont_n3 c=9.06878e-18
c3653 1174:net43 531:cont_n2 c=1.17347e-16
c3652 1174:net43 1145:vol_loopback c=2.26464e-19
c3651 1174:net43 521:cont_n2 c=1.23485e-18
c3650 1174:net43 1137:vol_loopback c=1.46934e-18
c3649 1174:net43 1129:vol_loopback c=9.53609e-18
c3648 1174:net43 1088:cont_n3 c=4.41246e-18
c3647 1174:net43 523:cont_n2 c=4.22687e-20
c3646 1174:net43 998:primary_storage c=3.1894e-19
c3645 1174:net43 334:vref1 c=3.0018e-19
c3644 1174:net43 350:vref1 c=9.3649e-19
c3643 1174:net43 1126:vol_loopback c=2.13216e-18
c3642 1174:net43 1117:cont_n3 c=1.47212e-18
c3641 1174:net43 1119:cont_n3 c=1.70254e-18
c3640 1174:net43 m15|gate c=1.37383e-19
c3639 1174:net43 1:ln_n124 c=1.33643e-17
c3638 1174:net43 m11|gate c=4.77053e-19
cg3637 1173:net43 0 c=4.88713e-18
c3636 1173:net43 1168:control_p c=7.25379e-19
c3635 1173:net43 147:ln_n60 c=8.58701e-18
c3634 1173:net43 1007:primary_storage c=2.44383e-18
c3633 1173:net43 1150:control_p c=1.34058e-17
c3632 1173:net43 521:cont_n2 c=2.89202e-20
c3631 1173:net43 998:primary_storage c=3.29573e-18
c3630 1173:net43 334:vref1 c=4.97343e-20
c3629 1173:net43 350:vref1 c=1.96492e-17
c3628 1173:net43 159:ln_n60 c=5.7327e-17
c3627 1173:net43 m15|gate c=2.55667e-18
c3626 1173:net43 1:ln_n124 c=1.51161e-17

cg3625 1172:net43 0 c=1.43695e-17
c3624 1172:net43 1168:control_p c=8.74483e-21
c3623 1172:net43 147:ln_n60 c=1.12407e-17
c3622 1172:net43 1030:primary_storage c=2.09319e-17
c3621 1172:net43 1007:primary_storage c=5.05156e-17
c3620 1172:net43 1150:control_p c=2.11476e-17
c3619 1172:net43 996:primary_storage c=8.67595e-18
c3618 1172:net43 998:primary_storage c=3.30318e-18
c3617 1172:net43 334:vref1 c=6.55746e-20
c3616 1172:net43 350:vref1 c=1.15279e-17
c3615 1172:net43 159:ln_n60 c=5.58864e-17
c3614 1172:net43 m15|gate c=5.82285e-18
c3613 1172:net43 1:ln_n124 c=2.24255e-17
cg3612 m15@2|drn 0 c=1.55678e-18
c3611 m15@2|drn 1168:control_p c=1.23934e-19
c3610 m15@2|drn m7|drn c=1.27075e-19
c3609 m15@2|drn 159:ln_n60 c=1.07768e-18
c3608 m15@2|drn m15|gate c=8.46956e-18
c3607 m15@2|drn 1:ln_n124 c=2.33687e-16
cg3606 m9|src 0 c=8.57354e-19
c3605 m9|src 1:ln_n124 c=2.29267e-16
c3604 m9|src 1120:cont_n3 c=7.18212e-19
c3603 m9|src 1088:cont_n3 c=2.50453e-19
c3602 m9|src m14|src c=1.11833e-20
c3601 m9|src m24|drn c=1.9587e-19
c3600 m9|src m9|gate c=1.13604e-17
c3599 m9|src m6|drn c=4.62235e-22
cg3598 1171:net43 0 c=2.05656e-19
c3597 1171:net43 108:ln_n52 c=4.2515e-18
c3596 1171:net43 1167:control_p c=2.07743e-18
c3595 1171:net43 96:ln_n52 c=1.57191e-18
c3594 1171:net43 1084:cont_n3 c=8.38099e-19
c3593 1171:net43 1137:vol_loopback c=1.1716e-18
c3592 1171:net43 1061:primary_storage c=6.25811e-19
c3591 1171:net43 1117:cont_n3 c=3.83952e-19
c3590 1171:net43 1:ln_n124 c=3.01124e-18
c3589 1171:net43 m11|gate c=1.14631e-18
c3588 1170:net43 1:ln_n124 c=2.2589e-18
c3587 1170:net43 108:ln_n52 c=3.01168e-20
c3586 1170:net43 1167:control_p c=9.66505e-18
c3585 1170:net43 1137:vol_loopback c=5.35841e-19
c3584 1170:net43 1129:vol_loopback c=3.01864e-18
c3583 1170:net43 1126:vol_loopback c=9.88064e-19

c3582 1170:net43 1084:cont_n3 c=1.67114e-18
c3581 1170:net43 m11|gate c=3.17612e-19
c3580 m14|drn m9|gate c=1.77728e-20
c3579 m14|drn m8|gate c=7.6508e-19
c3578 m14|drn m14|gate c=1.1939e-17
c3577 m14|drn 521:cont_n2 c=1.38438e-18
c3576 m14|drn m5@2|src c=1.74143e-19
c3575 m14|drn 159:ln_n60 c=1.55504e-19
c3574 m14|drn m5|drn c=8.41596e-24
c3573 m14|drn 1:ln_n124 c=2.287e-16
c3572 m8|src 1:ln_n124 c=2.33191e-16
c3571 m8|src 531:cont_n2 c=1.72475e-18
c3570 m8|src m15|src c=8.68303e-21
c3569 m8|src m15|gate c=4.51717e-19
c3568 m8|src m5@2|src c=6.75535e-23
c3567 m8|src m14|gate c=7.54468e-19
c3566 m8|src m11|gate c=1.51222e-20
c3565 m8|src m8|gate c=1.19369e-17
c3564 m15|drn 1166:control_p c=9.10929e-20
c3563 m15|drn m6|gate c=5.9854e-19
c3562 m15|drn m8|gate c=9.43307e-19
c3561 m15|drn m8|drn c=3.88004e-19
c3560 m15|drn 350:vref1 c=1.01437e-18
c3559 m15|drn 1148:control_p c=6.04464e-20
c3558 m15|drn m15|gate c=8.46956e-18
c3557 m15|drn 1165:control_p c=9.28419e-20
c3556 m15|drn 1:ln_n124 c=2.3483e-16
c3555 m15|drn m11|gate c=9.6851e-20
c3554 m15|drn m11|drn c=3.77728e-19
cg3553 1168:control_p 0 c=4.57436e-19
c3552 1168:control_p 539:cont_n1 c=2.44977e-18
c3551 1168:control_p 147:ln_n60 c=1.26363e-18
c3550 1168:control_p 721:gnd c=5.13814e-20
c3549 1168:control_p 722:gnd c=3.7435e-19
c3548 1168:control_p 998:primary_storage c=1.3634e-18
c3547 1168:control_p 350:vref1 c=4.58407e-19
c3546 1168:control_p 159:ln_n60 c=6.33432e-18
c3545 1168:control_p 1:ln_n124 c=4.14853e-18
cg3544 1167:control_p 0 c=9.67003e-19
c3543 1167:control_p 1084:cont_n3 c=1.00143e-17
c3542 1167:control_p m8|drn c=1.52516e-20
c3541 1167:control_p 334:vref1 c=1.25653e-20
c3540 1167:control_p 1116:cont_n3 c=8.45076e-18

c3539 1167:control_p 1117:cont_n3 c=1.98198e-17
c3538 1167:control_p 1:ln_n124 c=9.37509e-18
cg3537 1166:control_p 0 c=1.47985e-18
c3536 1166:control_p 989:primary_storage c=9.79236e-19
c3535 1166:control_p 1058:primary_storage c=1.37174e-17
c3534 1166:control_p 1084:cont_n3 c=4.64046e-18
c3533 1166:control_p 1059:primary_storage c=3.72618e-19
c3532 1166:control_p 842:gnd c=8.15726e-20
c3531 1166:control_p 1061:primary_storage c=4.12959e-19
c3530 1166:control_p 334:vref1 c=6.04334e-18
c3529 1166:control_p 1116:cont_n3 c=3.52826e-17
c3528 1166:control_p 1117:cont_n3 c=6.55502e-17
c3527 1166:control_p 1:ln_n124 c=1.49417e-17
cg3526 1165:control_p 0 c=1.18652e-18
c3525 1165:control_p 1084:cont_n3 c=3.19722e-18
c3524 1165:control_p m8|drn c=3.29009e-20
c3523 1165:control_p 334:vref1 c=3.94063e-17
c3522 1165:control_p 1116:cont_n3 c=7.5225e-20
c3521 1165:control_p 1117:cont_n3 c=2.22732e-17
c3520 1165:control_p 1:ln_n124 c=1.35026e-17
cg3519 1154:control_p 0 c=6.00713e-18
c3518 1154:control_p 1011:primary_storage c=3.55196e-19
c3517 1154:control_p 989:primary_storage c=9.3702e-19
c3516 1154:control_p 1058:primary_storage c=4.19381e-17
c3515 1154:control_p 1084:cont_n3 c=5.46211e-19
c3514 1154:control_p 352:vref1 c=2.02183e-19
c3513 1154:control_p 1059:primary_storage c=3.80382e-18
c3512 1154:control_p 1009:primary_storage c=1.34887e-18
c3511 1154:control_p 842:gnd c=1.25963e-19
c3510 1154:control_p 1061:primary_storage c=1.54232e-19
c3509 1154:control_p 334:vref1 c=2.53971e-18
c3508 1154:control_p 1116:cont_n3 c=1.62772e-18
c3507 1154:control_p 1117:cont_n3 c=4.14579e-18
c3506 1154:control_p m5@2|src c=6.54472e-19
c3505 1154:control_p 1:ln_n124 c=2.43587e-17
cg3504 1152:control_p 0 c=1.82967e-18
c3503 1152:control_p 539:cont_n1 c=1.49339e-17
c3502 1152:control_p 923:gnd c=6.5668e-18
c3501 1152:control_p 734:gnd c=2.68344e-18
c3500 1152:control_p 147:ln_n60 c=2.55098e-18
c3499 1152:control_p m6|gate c=3.57077e-18
c3498 1152:control_p 880:gnd c=3.65987e-17
c3497 1152:control_p 722:gnd c=7.4136e-19

c3496 1152:control_p 730:gnd c=9.54226e-20
c3495 1152:control_p m14|src c=1.92043e-19
c3494 1152:control_p 533:cont_n1 c=1.11645e-17
c3493 1152:control_p 998:primary_storage c=1.50127e-17
c3492 1152:control_p 350:vref1 c=8.0583e-19
c3491 1152:control_p 159:ln_n60 c=1.26802e-17
c3490 1152:control_p m15|gate c=6.45864e-19
c3489 1152:control_p 1:ln_n124 c=1.02801e-17
cg3488 1150:control_p 0 c=2.01556e-18
c3487 1150:control_p 539:cont_n1 c=7.11155e-19
c3486 1150:control_p 147:ln_n60 c=1.14272e-16
c3485 1150:control_p 694:gnd c=1.3976e-18
c3484 1150:control_p 975:primary_storage c=6.17516e-18
c3483 1150:control_p 1030:primary_storage c=6.9586e-19
c3482 1150:control_p 1007:primary_storage c=1.60069e-17
c3481 1150:control_p 1084:cont_n3 c=4.98621e-18
c3480 1150:control_p 880:gnd c=1.83098e-18
c3479 1150:control_p 352:vref1 c=4.09719e-18
c3478 1150:control_p c1|minus c=1.24117e-17
c3477 1150:control_p 842:gnd c=1.53228e-18
c3476 1150:control_p 533:cont_n1 c=5.00121e-19
c3475 1150:control_p c21|minus c=3.73062e-20
c3474 1150:control_p 996:primary_storage c=3.4227e-17
c3473 1150:control_p 997:primary_storage c=1.94371e-17
c3472 1150:control_p 990:primary_storage c=6.25924e-19
c3471 1150:control_p 998:primary_storage c=1.8295e-16
c3470 1150:control_p 334:vref1 c=2.63184e-17
c3469 1150:control_p 350:vref1 c=1.58355e-17
c3468 1150:control_p 159:ln_n60 c=1.42854e-17
c3467 1150:control_p 1:ln_n124 c=6.29635e-17
cg3466 m11|drn 0 c=9.66297e-20
c3465 m11|drn 1:ln_n124 c=1.0481e-16
c3464 m11|drn 1058:primary_storage c=7.46036e-19
c3463 m11|drn m15|src c=4.80883e-20
c3462 m11|drn 1011:primary_storage c=3.49464e-19
c3461 m11|drn m15|gate c=7.73098e-20
c3460 m11|drn m8|drn c=3.44084e-20
cg3459 m6|drn 0 c=1.73116e-19
c3458 m6|drn 539:cont_n1 c=1.49128e-18
c3457 m6|drn m13|src c=3.4651e-22
c3456 m6|drn m6|gate c=1.16904e-17
c3455 m6|drn m14|src c=1.22121e-18
c3454 m6|drn 533:cont_n1 c=6.21558e-19

c3453 m6|drn 159:ln_n60 c=2.32788e-19
c3452 m6|drn m15|gate c=7.71236e-19
c3451 m6|drn m15|src c=3.05661e-19
c3450 m6|drn 1:ln_n124 c=1.00349e-16
cg3449 m11|gate 0 c=1.09888e-17
c3448 m11|gate 1011:primary_storage c=1.25523e-18
c3447 m11|gate m4|src c=2.40383e-19
c3446 m11|gate 708:gnd c=2.11236e-19
c3445 m11|gate m8|drn c=3.38881e-18
c3444 m11|gate 1061:primary_storage c=2.21584e-19
c3443 m11|gate m24|drn c=7.53484e-20
c3442 m11|gate 1116:cont_n3 c=5.4816e-19
c3441 m11|gate 1117:cont_n3 c=1.97575e-18
c3440 m11|gate m15|gate c=1.16299e-19
c3439 m11|gate 1:ln_n124 c=9.22261e-17
cg3438 m8|gate 0 c=2.93403e-18
c3437 m8|gate m8|drn c=1.19459e-17
c3436 m8|gate 1061:primary_storage c=2.53421e-20
c3435 m8|gate 334:vref1 c=1.86992e-18
c3434 m8|gate 1117:cont_n3 c=5.37436e-18
c3433 m8|gate m5@2|src c=3.99797e-19
c3432 m8|gate m15|gate c=4.1625e-19
c3431 m8|gate 1:ln_n124 c=6.85975e-17
cg3430 1148:control_p 0 c=1.78494e-18
c3429 1148:control_p 1084:cont_n3 c=8.65983e-19
c3428 1148:control_p 352:vref1 c=3.2763e-19
c3427 1148:control_p 1059:primary_storage c=8.02482e-18
c3426 1148:control_p 842:gnd c=1.47224e-19
c3425 1148:control_p 334:vref1 c=1.81207e-17
c3424 1148:control_p 1116:cont_n3 c=7.10244e-19
c3423 1148:control_p 1:ln_n124 c=1.04539e-17
cg3422 1147:vol_loopback 0 c=1.07316e-18
c3421 1147:vol_loopback 1:ln_n124 c=3.6478e-18
c3420 1147:vol_loopback 911:gnd c=6.87205e-18
c3419 1147:vol_loopback 433:vreg c=2.47207e-20
c3418 1147:vol_loopback 869:gnd c=3.69648e-18
c3417 1147:vol_loopback 640:gnd c=1.34423e-18
c3416 1147:vol_loopback 701:gnd c=1.04774e-17
c3415 1147:vol_loopback 642:gnd c=2.83503e-17
cg3414 1146:vol_loopback 0 c=3.83964e-18
c3413 1146:vol_loopback 428:vreg c=1.58021e-16
c3412 1146:vol_loopback 432:vreg c=4.22666e-18
c3411 1146:vol_loopback 386:load3_cont c=1.11304e-20

c3410 1146:vol_loopback 434:vreg c=1.55893e-18
c3409 1146:vol_loopback c15|plus c=4.58094e-18
c3408 1146:vol_loopback 701:gnd c=8.08116e-19
c3407 1146:vol_loopback 398:load3_cont c=8.68845e-20
c3406 1146:vol_loopback 487:vreg c=3.26065e-21
c3405 1146:vol_loopback 433:vreg c=1.66534e-16
c3404 1146:vol_loopback 911:gnd c=5.94514e-19
c3403 1146:vol_loopback 1120:cont_n3 c=4.26489e-18
c3402 1146:vol_loopback 467:vreg c=2.59656e-17
c3401 1146:vol_loopback 1115:cont_n3 c=2.08455e-18
c3400 1146:vol_loopback 739:gnd c=2.1747e-19
c3399 1146:vol_loopback 1:ln_n124 c=2.5571e-17
cg3398 1145:vol_loopback 0 c=1.62898e-17
c3397 1145:vol_loopback 108:ln_n52 c=3.6791e-19
c3396 1145:vol_loopback m9|gate c=6.43026e-20
c3395 1145:vol_loopback 1084:cont_n3 c=7.59846e-19
c3394 1145:vol_loopback 488:vreg c=5.67565e-19
c3393 1145:vol_loopback 1088:cont_n3 c=8.61164e-18
c3392 1145:vol_loopback 1:ln_n124 c=6.49378e-18
cg3391 1144:vol_loopback 0 c=1.30557e-17
c3390 1144:vol_loopback 428:vreg c=5.42846e-18
c3389 1144:vol_loopback c15|plus c=1.29029e-18
c3388 1144:vol_loopback 433:vreg c=1.11919e-18
c3387 1144:vol_loopback 911:gnd c=4.98414e-21
c3386 1144:vol_loopback 687:gnd c=1.1795e-20
c3385 1144:vol_loopback 123:ln_n36 c=1.76951e-18
c3384 1144:vol_loopback 1:ln_n124 c=4.65289e-18
cg3383 1143:vol_loopback 0 c=1.54158e-18
c3382 1143:vol_loopback 642:gnd c=1.59228e-18
c3381 1143:vol_loopback 701:gnd c=4.97751e-17
c3380 1143:vol_loopback 869:gnd c=5.42903e-18
c3379 1143:vol_loopback 398:load3_cont c=5.47867e-19
c3378 1143:vol_loopback 433:vreg c=2.92847e-19
c3377 1143:vol_loopback 911:gnd c=2.32885e-17
c3376 1143:vol_loopback 1:ln_n124 c=5.14607e-18
cg3375 1137:vol_loopback 0 c=5.90439e-19
c3374 1137:vol_loopback 108:ln_n52 c=5.6148e-19
c3373 1137:vol_loopback 96:ln_n52 c=3.30634e-19
c3372 1137:vol_loopback 1084:cont_n3 c=1.49098e-19
c3371 1137:vol_loopback 1088:cont_n3 c=2.8324e-19
c3370 1137:vol_loopback 1:ln_n124 c=3.70307e-18
cg3369 1133:vol_loopback 0 c=1.56469e-20
c3368 1133:vol_loopback 1:ln_n124 c=8.49153e-20

c3367 1133:vol_loopback 1115:cont_n3 c=5.05801e-19
c3366 1133:vol_loopback 388:load3_cont c=8.18248e-21
c3365 1133:vol_loopback 433:vreg c=7.95407e-20
c3364 1133:vol_loopback c15|plus c=1.13593e-19
c3363 1133:vol_loopback 428:vreg c=2.9363e-20
cg3362 1132:vol_loopback 0 c=3.8126e-17
c3361 1132:vol_loopback 428:vreg c=5.99403e-18
c3360 1132:vol_loopback 386:load3_cont c=1.15699e-17
c3359 1132:vol_loopback 385:load3_cont c=1.13417e-17
c3358 1132:vol_loopback 1084:cont_n3 c=1.48376e-19
c3357 1132:vol_loopback c15|plus c=3.90984e-18
c3356 1132:vol_loopback 642:gnd c=6.22017e-18
c3355 1132:vol_loopback 701:gnd c=1.1403e-17
c3354 1132:vol_loopback 433:vreg c=3.33183e-17
c3353 1132:vol_loopback 134:ln_n36 c=3.58364e-18
c3352 1132:vol_loopback 911:gnd c=2.44485e-17
c3351 1132:vol_loopback 1120:cont_n3 c=1.18784e-19
c3350 1132:vol_loopback 1115:cont_n3 c=2.08644e-18
c3349 1132:vol_loopback 739:gnd c=7.22371e-19
c3348 1132:vol_loopback 123:ln_n36 c=5.72963e-17
c3347 1132:vol_loopback 1:ln_n124 c=2.79013e-17
c3346 1132:vol_loopback 388:load3_cont c=1.99913e-17
cg3345 1129:vol_loopback 0 c=7.88161e-19
c3344 1129:vol_loopback 108:ln_n52 c=8.71632e-19
c3343 1129:vol_loopback m9|gate c=8.98689e-19
c3342 1129:vol_loopback 96:ln_n52 c=3.77417e-19
c3341 1129:vol_loopback 1084:cont_n3 c=3.62235e-19
c3340 1129:vol_loopback 488:vreg c=2.44052e-20
c3339 1129:vol_loopback 1088:cont_n3 c=5.06237e-18
c3338 1129:vol_loopback 1:ln_n124 c=4.77416e-18
cg3337 1126:vol_loopback 0 c=5.68939e-18
c3336 1126:vol_loopback 108:ln_n52 c=1.13415e-17
c3335 1126:vol_loopback 914:gnd c=7.31399e-19
c3334 1126:vol_loopback 96:ln_n52 c=8.14165e-17
c3333 1126:vol_loopback 434:vreg c=1.22293e-17
c3332 1126:vol_loopback 1084:cont_n3 c=4.57889e-17
c3331 1126:vol_loopback 488:vreg c=1.32398e-17
c3330 1126:vol_loopback c21|minus c=5.79728e-18
c3329 1126:vol_loopback 487:vreg c=8.14616e-18
c3328 1126:vol_loopback 433:vreg c=8.73615e-19
c3327 1126:vol_loopback 1088:cont_n3 c=1.74499e-17
c3326 1126:vol_loopback 1119:cont_n3 c=6.69027e-19
c3325 1126:vol_loopback 687:gnd c=1.6506e-18

c3324 1126:vol_loopback 1087:cont_n3 c=2.2055e-18
c3323 1126:vol_loopback 1115:cont_n3 c=2.64995e-17
c3322 1126:vol_loopback 740:gnd c=6.12923e-19
c3321 1126:vol_loopback 1:ln_n124 c=3.60122e-17
c3320 1126:vol_loopback 739:gnd c=4.28978e-19
cg3319 1125:vol_loopback 0 c=8.27824e-18
c3318 1125:vol_loopback 428:vreg c=9.49937e-18
c3317 1125:vol_loopback 385:load3_cont c=2.55388e-18
c3316 1125:vol_loopback 1084:cont_n3 c=3.96672e-20
c3315 1125:vol_loopback c15|plus c=1.00954e-17
c3314 1125:vol_loopback 701:gnd c=1.34429e-18
c3313 1125:vol_loopback 433:vreg c=1.54168e-17
c3312 1125:vol_loopback 911:gnd c=3.63897e-19
c3311 1125:vol_loopback 1120:cont_n3 c=1.06024e-18
c3310 1125:vol_loopback 1115:cont_n3 c=6.9569e-18
c3309 1125:vol_loopback 123:ln_n36 c=8.90678e-20
c3308 1125:vol_loopback 1:ln_n124 c=9.96537e-18
c3307 1125:vol_loopback 388:load3_cont c=8.67081e-20
cg3306 1123:vol_loopback 0 c=2.5032e-17
c3305 1123:vol_loopback 428:vreg c=1.68369e-18
c3304 1123:vol_loopback 540:ln_n125 c=3.56156e-17
c3303 1123:vol_loopback 385:load3_cont c=3.68105e-18
c3302 1123:vol_loopback c15|plus c=8.60325e-18
c3301 1123:vol_loopback 542:ln_n125 c=1.19801e-17
c3300 1123:vol_loopback 543:ln_n125 c=1.34062e-18
c3299 1123:vol_loopback 541:ln_n125 c=1.08497e-17
c3298 1123:vol_loopback 4:ln_n128 c=2.84503e-18
c3297 1123:vol_loopback 687:gnd c=4.65902e-18
c3296 1123:vol_loopback 1115:cont_n3 c=6.38685e-18
c3295 1123:vol_loopback 1:ln_n124 c=1.8034e-17
c3294 1123:vol_loopback 388:load3_cont c=1.12836e-19
cg3293 1122:vol_loopback 0 c=2.29528e-17
c3292 1122:vol_loopback 428:vreg c=1.99858e-18
c3291 1122:vol_loopback 391:load3_cont c=5.25766e-19
c3290 1122:vol_loopback 96:ln_n52 c=6.3907e-18
c3289 1122:vol_loopback 386:load3_cont c=9.77004e-19
c3288 1122:vol_loopback 385:load3_cont c=1.97708e-18
c3287 1122:vol_loopback 1084:cont_n3 c=7.00101e-18
c3286 1122:vol_loopback c15|plus c=6.20198e-17
c3285 1122:vol_loopback c21|minus c=7.19546e-19
c3284 1122:vol_loopback 433:vreg c=6.20248e-19
c3283 1122:vol_loopback 542:ln_n125 c=5.95231e-19
c3282 1122:vol_loopback 543:ln_n125 c=1.14465e-16

c3281 1122:vol_loopback 4:ln_n128 c=2.3473e-17
c3280 1122:vol_loopback 687:gnd c=3.32566e-17
c3279 1122:vol_loopback 1115:cont_n3 c=3.90358e-17
c3278 1122:vol_loopback 1:ln_n124 c=1.82752e-17
c3277 d9|cathode 1:ln_n124 c=7.9571e-16
c3276 d9|cathode 467:vreg c=1.01708e-16
c3275 d9|cathode 869:gnd c=1.57276e-19
c3274 d9|cathode 433:vreg c=1.13822e-17
c3273 d9|cathode m24|src c=1.07897e-23
c3272 d9|cathode 428:vreg c=1.14709e-17
cg3271 d10|cathode 0 c=4.63811e-23
c3270 d10|cathode 1:ln_n124 c=7.7658e-17
c3269 d10|cathode 911:gnd c=1.26615e-18
c3268 d10|cathode 467:vreg c=5.20781e-20
c3267 d10|cathode 869:gnd c=2.37452e-17
c3266 d10|cathode 701:gnd c=1.26965e-18
c3265 d10|cathode m23@2|gate c=1.20294e-19
c3264 d10|cathode 642:gnd c=3.08432e-19
cg3263 m10|gate 0 c=2.60257e-17
c3262 m10|gate 108:ln_n52 c=3.65725e-19
c3261 m10|gate m9|drn c=8.33615e-19
c3260 m10|gate 488:vreg c=4.99264e-19
c3259 m10|gate 1088:cont_n3 c=2.31159e-18
c3258 m10|gate 1:ln_n124 c=6.37567e-17
cg3257 1120:cont_n3 0 c=4.8163e-18
c3256 1120:cont_n3 108:ln_n52 c=3.57866e-18
c3255 1120:cont_n3 428:vreg c=6.70371e-17
c3254 1120:cont_n3 429:vreg c=8.56555e-17
c3253 1120:cont_n3 435:vreg c=4.54566e-17
c3252 1120:cont_n3 438:vreg c=4.53834e-17
c3251 1120:cont_n3 440:vreg c=4.61821e-17
c3250 1120:cont_n3 442:vreg c=4.61872e-17
c3249 1120:cont_n3 489:vreg c=5.65139e-17
c3248 1120:cont_n3 432:vreg c=2.1297e-17
c3247 1120:cont_n3 434:vreg c=2.9898e-17
c3246 1120:cont_n3 490:vreg c=2.66999e-21
c3245 1120:cont_n3 430:vreg c=2.13599e-20
c3244 1120:cont_n3 c15|minus c=5.65017e-20
c3243 1120:cont_n3 439:vreg c=2.13599e-20
c3242 1120:cont_n3 443:vreg c=4.27198e-20
c3241 1120:cont_n3 431:vreg c=2.66999e-21
c3240 1120:cont_n3 433:vreg c=1.58148e-17
c3239 1120:cont_n3 67:ln_n55 c=2.48687e-18

c3238 1120:cont_n3 220:ln_n38 c=2.48687e-18
c3237 1120:cont_n3 423:vreg c=1.53101e-18
c3236 1120:cont_n3 467:vreg c=1.00026e-17
c3235 1120:cont_n3 1:ln_n124 c=7.261e-17
cg3234 1119:cont_n3 0 c=6.77165e-19
c3233 1119:cont_n3 1:ln_n124 c=6.16101e-18
c3232 1119:cont_n3 108:ln_n52 c=6.13241e-20
c3231 1119:cont_n3 523:cont_n2 c=2.71641e-19
c3230 1119:cont_n3 521:cont_n2 c=6.87265e-18
c3229 1119:cont_n3 m14|gate c=3.53097e-19
c3228 1119:cont_n3 531:cont_n2 c=1.43963e-17
cg3227 1118:cont_n3 0 c=4.24876e-19
c3226 1118:cont_n3 914:gnd c=2.51351e-17
c3225 1118:cont_n3 913:gnd c=3.06262e-20
c3224 1118:cont_n3 m9|drn c=4.46682e-19
c3223 1118:cont_n3 531:cont_n2 c=1.99514e-19
c3222 1118:cont_n3 488:vreg c=4.87252e-18
c3221 1118:cont_n3 487:vreg c=9.41883e-18
c3220 1118:cont_n3 433:vreg c=7.0402e-21
c3219 1118:cont_n3 530:cont_n2 c=1.62987e-17
c3218 1118:cont_n3 523:cont_n2 c=1.61434e-18
c3217 1118:cont_n3 m24|gate c=3.93536e-18
c3216 1118:cont_n3 740:gnd c=1.84108e-18
c3215 1118:cont_n3 1:ln_n124 c=3.78837e-18
cg3214 1117:cont_n3 0 c=3.08943e-18
c3213 1117:cont_n3 1:ln_n124 c=1.47358e-17
cg3212 1116:cont_n3 0 c=4.9157e-19
c3211 1116:cont_n3 1:ln_n124 c=4.01186e-18
c3210 1115:cont_n3 914:gnd c=5.2487e-19
c3209 1115:cont_n3 429:vreg c=2.35871e-18
c3208 1115:cont_n3 96:ln_n52 c=1.59555e-18
c3207 1115:cont_n3 432:vreg c=3.91217e-19
c3206 1115:cont_n3 60:ln_n55 c=1.12514e-18
c3205 1115:cont_n3 434:vreg c=3.76855e-17
c3204 1115:cont_n3 c15|minus c=1.0418e-20
c3203 1115:cont_n3 487:vreg c=5.11367e-18
c3202 1115:cont_n3 433:vreg c=1.82363e-17
c3201 1115:cont_n3 909:gnd c=8.9602e-19
c3200 1115:cont_n3 912:gnd c=4.12998e-18
c3199 1115:cont_n3 523:cont_n2 c=3.50054e-19
c3198 1115:cont_n3 739:gnd c=1.05462e-17
c3197 1115:cont_n3 1:ln_n124 c=3.15124e-17
cg3196 1091:cont_n3 0 c=2.7615e-18

c3195 1091:cont_n3 438:vreg c=1.55392e-17
c3194 1091:cont_n3 440:vreg c=2.68143e-17
c3193 1091:cont_n3 442:vreg c=1.88358e-17
c3192 1091:cont_n3 213:ln_n38 c=1.30295e-17
c3191 1091:cont_n3 c15|minus c=4.5449e-16
c3190 1091:cont_n3 443:vreg c=8.46973e-19
c3189 1091:cont_n3 687:gnd c=2.10659e-17
c3188 1091:cont_n3 1:ln_n124 c=1.02173e-17
cg3187 1090:cont_n3 0 c=2.41572e-18
c3186 1090:cont_n3 429:vreg c=1.55392e-17
c3185 1090:cont_n3 435:vreg c=2.68143e-17
c3184 1090:cont_n3 438:vreg c=1.88358e-17
c3183 1090:cont_n3 96:ln_n52 c=3.24048e-19
c3182 1090:cont_n3 60:ln_n55 c=1.30295e-17
c3181 1090:cont_n3 430:vreg c=4.23486e-19
c3180 1090:cont_n3 c15|minus c=4.5449e-16
c3179 1090:cont_n3 439:vreg c=4.23486e-19
c3178 1090:cont_n3 687:gnd c=2.10659e-17
c3177 1090:cont_n3 1:ln_n124 c=1.04198e-17
cg3176 1088:cont_n3 0 c=1.9654e-18
c3175 1088:cont_n3 914:gnd c=2.67352e-19
c3174 1088:cont_n3 108:ln_n52 c=1.25547e-18
c3173 1088:cont_n3 428:vreg c=9.97053e-22
c3172 1088:cont_n3 m9|drn c=2.0522e-19
c3171 1088:cont_n3 96:ln_n52 c=4.0319e-19
c3170 1088:cont_n3 434:vreg c=1.38393e-18
c3169 1088:cont_n3 531:cont_n2 c=2.6513e-17
c3168 1088:cont_n3 m14|gate c=4.69056e-19
c3167 1088:cont_n3 488:vreg c=3.01374e-17
c3166 1088:cont_n3 521:cont_n2 c=5.39929e-18
c3165 1088:cont_n3 487:vreg c=1.36603e-17
c3164 1088:cont_n3 433:vreg c=1.69173e-20
c3163 1088:cont_n3 530:cont_n2 c=5.51751e-19
c3162 1088:cont_n3 523:cont_n2 c=1.02618e-18
c3161 1088:cont_n3 m24|gate c=3.17489e-19
c3160 1088:cont_n3 1:ln_n124 c=2.91674e-17
cg3159 1087:cont_n3 0 c=1.80029e-18
c3158 1087:cont_n3 914:gnd c=1.84108e-18
c3157 1087:cont_n3 108:ln_n52 c=4.34609e-20
c3156 1087:cont_n3 913:gnd c=8.57533e-20
c3155 1087:cont_n3 531:cont_n2 c=7.5624e-19
c3154 1087:cont_n3 m14|gate c=3.22104e-20
c3153 1087:cont_n3 488:vreg c=8.27156e-18

c3152 1087:cont_n3 521:cont_n2 c=1.1718e-18
c3151 1087:cont_n3 530:cont_n2 c=6.11623e-18
c3150 1087:cont_n3 523:cont_n2 c=9.73164e-18
c3149 1087:cont_n3 m24|gate c=1.3235e-18
c3148 1087:cont_n3 1:ln_n124 c=1.63685e-17
cg3147 1084:cont_n3 0 c=4.9296e-17
c3146 1084:cont_n3 914:gnd c=5.89121e-18
c3145 1084:cont_n3 913:gnd c=1.31445e-18
c3144 1084:cont_n3 96:ln_n52 c=1.03905e-17
c3143 1084:cont_n3 434:vreg c=6.30039e-18
c3142 1084:cont_n3 531:cont_n2 c=1.76056e-17
c3141 1084:cont_n3 488:vreg c=1.96457e-18
c3140 1084:cont_n3 521:cont_n2 c=1.27302e-17
c3139 1084:cont_n3 c21|minus c=6.57394e-19
c3138 1084:cont_n3 487:vreg c=1.44312e-18
c3137 1084:cont_n3 530:cont_n2 c=1.28304e-17
c3136 1084:cont_n3 909:gnd c=2.1727e-18
c3135 1084:cont_n3 523:cont_n2 c=1.97381e-17
c3134 1084:cont_n3 687:gnd c=9.44706e-21
c3133 1084:cont_n3 740:gnd c=2.5618e-18
c3132 1084:cont_n3 1:ln_n124 c=5.86961e-17
c3131 1084:cont_n3 739:gnd c=2.12713e-17
cg3130 m24|drn 0 c=1.10857e-20
c3129 m24|drn 1:ln_n124 c=1.03067e-16
c3128 m24|drn 467:vreg c=5.59166e-20
c3127 m24|drn 523:cont_n2 c=2.88626e-19
c3126 m24|drn m12|src c=7.40384e-19
c3125 m24|drn m9|drn c=1.36219e-18
c3124 m24|drn m24|gate c=1.16727e-17
c3123 m24|drn m12|gate c=5.28542e-20
cg3122 m8|drn 0 c=1.46252e-18
c3121 m8|drn 1:ln_n124 c=2.28197e-16
cg3120 m9|gate 0 c=1.93747e-18
c3119 m9|gate 914:gnd c=2.12819e-19
c3118 m9|gate 108:ln_n52 c=2.87284e-18
c3117 m9|gate m9|drn c=1.13687e-17
c3116 m9|gate 434:vreg c=7.37042e-19
c3115 m9|gate 924:gnd c=1.10379e-19
c3114 m9|gate m12|drn c=4.3147e-20
c3113 m9|gate m14|gate c=6.00301e-20
c3112 m9|gate 488:vreg c=3.32389e-18
c3111 m9|gate 521:cont_n2 c=2.31938e-20
c3110 m9|gate 487:vreg c=1.14217e-18

c3109 m9|gate 433:vreg c=3.54612e-19
c3108 m9|gate 523:cont_n2 c=8.81786e-20
c3107 m9|gate m24|src c=4.72074e-19
c3106 m9|gate m24|gate c=4.72561e-19
c3105 m9|gate 467:vreg c=1.55577e-19
c3104 m9|gate 1:ln_n124 c=7.08853e-17
cg3103 c15|plus 0 c=3.64361e-17
c3102 c15|plus 687:gnd c=6.55842e-17
c3101 c15|plus 60:ln_n55 c=5.62059e-17
c3100 c15|plus 213:ln_n38 c=5.47976e-17
c3099 c15|plus 432:vreg c=1.3935e-17
c3098 c15|plus 67:ln_n55 c=3.18072e-18
c3097 c15|plus 220:ln_n38 c=3.04789e-18
c3096 c15|plus 434:vreg c=1.18886e-17
c3095 c15|plus 428:vreg c=1.83112e-17
c3094 c15|plus 490:vreg c=8.63107e-22
c3093 c15|plus 96:ln_n52 c=3.06273e-17
c3092 c15|plus 431:vreg c=8.63107e-22
c3091 c15|plus 108:ln_n52 c=4.20711e-18
c3090 c15|plus 429:vreg c=1.21454e-17
c3089 c15|plus 435:vreg c=8.60687e-18
c3088 c15|plus 438:vreg c=8.33012e-18
c3087 c15|plus 440:vreg c=8.42008e-18
c3086 c15|plus 442:vreg c=8.17218e-18
c3085 c15|plus 489:vreg c=8.60348e-18
c3084 c15|plus c15|minus c=9.99506e-17
c3083 c15|plus 433:vreg c=4.68405e-18
c3082 c15|plus c21|minus c=3.44219e-19
c3081 c15|plus 1:ln_n124 c=2.79473e-17
cg3080 1083:load3 0 c=8.05002e-18
c3079 1083:load3 913:gnd c=2.2636e-20
c3078 1083:load3 975:primary_storage c=3.91336e-18
c3077 1083:load3 974:primary_storage c=6.73906e-18
c3076 1083:load3 1002:primary_storage c=7.66688e-19
c3075 1083:load3 m23@2|gate c=3.95404e-20
c3074 1083:load3 399:load3_cont c=7.05887e-18
c3073 1083:load3 m23|gate c=5.81061e-18
c3072 1083:load3 909:gnd c=7.68338e-19
c3071 1083:load3 912:gnd c=2.24173e-17
c3070 1083:load3 m21@2|gate c=1.23529e-19
c3069 1083:load3 1056:primary_storage c=2.56197e-17
c3068 1083:load3 370:load1_cont c=2.11921e-18
c3067 1083:load3 1008:primary_storage c=2.35423e-17

c3066 1083:load3 739:gnd c=1.48615e-17
c3065 1083:load3 1:ln_n124 c=2.70514e-17
cg3064 1082:load3 0 c=1.78301e-17
c3063 1082:load3 373:load2_cont c=5.53297e-18
c3062 1082:load3 378:load2_cont c=4.02139e-18
c3061 1082:load3 975:primary_storage c=2.99698e-19
c3060 1082:load3 990:primary_storage c=2.0293e-18
c3059 1082:load3 988:primary_storage c=1.97287e-19
c3058 1082:load3 379:load2_cont c=3.47172e-17
c3057 1082:load3 994:primary_storage c=3.52391e-18
c3056 1082:load3 371:load1_cont c=1.00877e-17
c3055 1082:load3 372:load1_cont c=2.70976e-17
c3054 1082:load3 1:ln_n124 c=5.61898e-18
cg3053 1081:load3 0 c=3.47585e-17
c3052 1081:load3 386:load3_cont c=8.7194e-18
c3051 1081:load3 974:primary_storage c=4.66059e-18
c3050 1081:load3 976:primary_storage c=4.72372e-20
c3049 1081:load3 186:ln_n64 c=7.55971e-20
c3048 1081:load3 701:gnd c=2.53628e-17
c3047 1081:load3 1002:primary_storage c=7.66688e-19
c3046 1081:load3 m23@2|gate c=5.79145e-18
c3045 1081:load3 399:load3_cont c=1.63153e-17
c3044 1081:load3 398:load3_cont c=1.03709e-17
c3043 1081:load3 m23|gate c=4.94717e-20
c3042 1081:load3 650:gnd c=8.34818e-20
c3041 1081:load3 911:gnd c=1.00116e-18
c3040 1081:load3 1008:primary_storage c=4.85033e-17
c3039 1081:load3 1:ln_n124 c=1.90345e-17
cg3038 1080:load3 0 c=3.66091e-18
c3037 1080:load3 1002:primary_storage c=1.2802e-19
c3036 1080:load3 m23|src c=3.75519e-19
c3035 1080:load3 1056:primary_storage c=2.18724e-19
c3034 1080:load3 1008:primary_storage c=1.01376e-18
c3033 1080:load3 739:gnd c=2.90412e-18
c3032 1080:load3 1:ln_n124 c=8.51303e-18
cg3031 1079:load3 0 c=2.44279e-18
c3030 1079:load3 975:primary_storage c=3.6093e-19
c3029 1079:load3 974:primary_storage c=6.52077e-21
c3028 1079:load3 1002:primary_storage c=2.74547e-19
c3027 1079:load3 399:load3_cont c=1.20613e-18
c3026 1079:load3 m23|gate c=1.63447e-18
c3025 1079:load3 m23|src c=4.35602e-19
c3024 1079:load3 1056:primary_storage c=2.41634e-18

c3023 1079:load3 1008:primary_storage c=4.82637e-18
c3022 1079:load3 739:gnd c=1.20388e-18
c3021 1079:load3 1:ln_n124 c=5.27709e-18
cg3020 1071:load3 0 c=4.3796e-18
c3019 1071:load3 975:primary_storage c=3.4863e-19
c3018 1071:load3 974:primary_storage c=1.84517e-19
c3017 1071:load3 1002:primary_storage c=1.64495e-18
c3016 1071:load3 399:load3_cont c=1.69488e-19
c3015 1071:load3 739:gnd c=2.98126e-18
c3014 1071:load3 1:ln_n124 c=4.60996e-18
cg3013 1070:load3 0 c=5.34756e-17
c3012 1070:load3 391:load3_cont c=1.4416e-17
c3011 1070:load3 373:load2_cont c=2.40518e-18
c3010 1070:load3 232:ln_n126 c=7.32734e-17
c3009 1070:load3 975:primary_storage c=9.13297e-17
c3008 1070:load3 386:load3_cont c=1.89751e-18
c3007 1070:load3 385:load3_cont c=5.45047e-17
c3006 1070:load3 974:primary_storage c=1.31535e-17
c3005 1070:load3 976:primary_storage c=3.84511e-18
c3004 1070:load3 1002:primary_storage c=1.34977e-17
c3003 1070:load3 545:ln_n127 c=2.99371e-18
c3002 1070:load3 c1|minus c=3.56013e-18
c3001 1070:load3 544:ln_n127 c=3.99569e-17
c3000 1070:load3 233:ln_n126 c=9.11925e-17
c2999 1070:load3 175:ln_n64 c=9.48825e-17
c2998 1070:load3 990:primary_storage c=1.51115e-18
c2997 1070:load3 988:primary_storage c=4.96182e-19
c2996 1070:load3 994:primary_storage c=5.48352e-17
c2995 1070:load3 370:load1_cont c=4.02012e-20
c2994 1070:load3 371:load1_cont c=9.51046e-19
c2993 1070:load3 1008:primary_storage c=4.1169e-19
c2992 1070:load3 372:load1_cont c=8.78489e-18
c2991 1070:load3 1:ln_n124 c=1.11624e-16
c2990 1070:load3 739:gnd c=1.83003e-18
cg2989 1069:load3 0 c=1.29698e-17
c2988 1069:load3 975:primary_storage c=2.67986e-19
c2987 1069:load3 974:primary_storage c=3.35629e-19
c2986 1069:load3 976:primary_storage c=6.22259e-19
c2985 1069:load3 1002:primary_storage c=3.74856e-18
c2984 1069:load3 399:load3_cont c=5.32772e-20
c2983 1069:load3 1056:primary_storage c=6.55425e-20
c2982 1069:load3 1008:primary_storage c=1.4733e-19
c2981 1069:load3 739:gnd c=2.79899e-20

c2980 1069:load3 1:ln_n124 c=4.55418e-18
cg2979 1068:load3 0 c=1.8781e-17
c2978 1068:load3 974:primary_storage c=5.10572e-22
c2977 1068:load3 976:primary_storage c=3.94781e-19
c2976 1068:load3 1002:primary_storage c=4.6314e-19
c2975 1068:load3 m23@2|gate c=1.63122e-18
c2974 1068:load3 399:load3_cont c=1.26311e-18
c2973 1068:load3 m23|gate c=3.48682e-20
c2972 1068:load3 m23|src c=7.7073e-19
c2971 1068:load3 1056:primary_storage c=1.21843e-19
c2970 1068:load3 1008:primary_storage c=1.3001e-17
c2969 1068:load3 739:gnd c=2.56267e-19
c2968 1068:load3 1:ln_n124 c=8.10717e-18
cg2967 1065:load3 0 c=4.73014e-19
c2966 1065:load3 391:load3_cont c=7.50259e-19
c2965 1065:load3 232:ln_n126 c=4.89636e-19
c2964 1065:load3 975:primary_storage c=2.73971e-18
c2963 1065:load3 385:load3_cont c=3.21541e-18
c2962 1065:load3 974:primary_storage c=6.83075e-18
c2961 1065:load3 1002:primary_storage c=8.42966e-18
c2960 1065:load3 233:ln_n126 c=6.70819e-17
c2959 1065:load3 1008:primary_storage c=5.20901e-19
c2958 1065:load3 371:load1_cont c=1.14126e-17
c2957 1065:load3 372:load1_cont c=1.10418e-17
c2956 1065:load3 1:ln_n124 c=3.16566e-18
cg2955 1064:load3 0 c=9.39784e-18
c2954 1064:load3 1:ln_n124 c=8.13545e-18
c2953 1064:load3 379:load2_cont c=4.33057e-18
c2952 1064:load3 371:load1_cont c=1.42962e-18
c2951 1064:load3 990:primary_storage c=2.93908e-19
c2950 1064:load3 231:ln_n126 c=2.80078e-17
c2949 1064:load3 975:primary_storage c=2.31207e-20
cg2948 m23@2|drn 0 c=1.7033e-18
c2947 m23@2|drn 186:ln_n64 c=2.67366e-19
c2946 m23@2|drn m23@2|gate c=9.03067e-18
c2945 m23@2|drn 869:gnd c=4.00144e-18
c2944 m23@2|drn 399:load3_cont c=1.68457e-18
c2943 m23@2|drn 398:load3_cont c=4.22506e-19
c2942 m23@2|drn 1:ln_n124 c=2.21887e-16
cg2941 m23|drn 0 c=1.51418e-18
c2940 m23|drn m12|src c=5.89604e-24
c2939 m23|drn 399:load3_cont c=5.00907e-19
c2938 m23|drn m23|gate c=9.04518e-18

c2937 m23|drn m21@2|gate c=1.1132e-20
c2936 m23|drn 370:load1_cont c=7.42024e-19
c2935 m23|drn 1:ln_n124 c=2.28086e-16
cg2934 1063:load3 0 c=2.45997e-18
c2933 1063:load3 974:primary_storage c=5.9498e-22
c2932 1063:load3 1002:primary_storage c=1.84981e-20
c2931 1063:load3 m23@2|gate c=1.46192e-20
c2930 1063:load3 m23|src c=5.35437e-20
c2929 1063:load3 650:gnd c=9.11438e-21
c2928 1063:load3 1008:primary_storage c=4.64877e-19
c2927 1063:load3 1:ln_n124 c=3.42911e-18
cg2926 1062:primary_storage 0 c=1.45859e-17
c2925 1062:primary_storage 971:vsec_storage c=1.78161e-17
c2924 1062:primary_storage 253:vref2 c=7.23441e-19
c2923 1062:primary_storage m30|src c=2.88785e-19
c2922 1062:primary_storage m0|gate c=1.50722e-18
c2921 1062:primary_storage 333:vref1 c=4.16792e-17
c2920 1062:primary_storage 256:vref2 c=2.3192e-18
c2919 1062:primary_storage 812:gnd c=6.61625e-18
c2918 1062:primary_storage m7|gate c=3.74032e-18
c2917 1062:primary_storage 958:vsec_storage c=7.75467e-17
c2916 1062:primary_storage 970:vsec_storage c=1.73774e-17
c2915 1062:primary_storage 1:ln_n124 c=4.19896e-17
cg2914 1061:primary_storage 0 c=1.87456e-17
c2913 1061:primary_storage 934:net36 c=2.16676e-17
c2912 1061:primary_storage m3|drn c=3.91688e-19
c2911 1061:primary_storage m3|gate c=1.49116e-18
c2910 1061:primary_storage 238:vref2 c=1.1834e-18
c2909 1061:primary_storage 253:vref2 c=1.17281e-17
c2908 1061:primary_storage m20|gate c=1.35607e-20
c2907 1061:primary_storage 915:gnd c=9.25438e-18
c2906 1061:primary_storage 237:vref2 c=8.54475e-19
c2905 1061:primary_storage 916:gnd c=3.27879e-18
c2904 1061:primary_storage 245:vref2 c=1.72737e-17
c2903 1061:primary_storage 333:vref1 c=2.76141e-18
c2902 1061:primary_storage 254:vref2 c=1.8744e-17
c2901 1061:primary_storage m5@2|gate c=1.93768e-18
c2900 1061:primary_storage 935:net36 c=1.45252e-18
c2899 1061:primary_storage 234:vref2 c=1.82081e-17
c2898 1061:primary_storage 580:vsec_storage_rf c=1.24229e-17
c2897 1061:primary_storage 1:ln_n124 c=6.71254e-17
c2896 1061:primary_storage m1|gate c=5.44113e-19
cg2895 1060:primary_storage 0 c=1.62965e-17

c2894 1060:primary_storage 934:net36 c=6.01259e-18
c2893 1060:primary_storage m3|gate c=8.43988e-19
c2892 1060:primary_storage 918:gnd c=4.21559e-18
c2891 1060:primary_storage 253:vref2 c=5.0482e-20
c2890 1060:primary_storage m20|gate c=3.80228e-18
c2889 1060:primary_storage 915:gnd c=1.9414e-17
c2888 1060:primary_storage 579:vsec_storage_rf c=8.20293e-18
c2887 1060:primary_storage 916:gnd c=2.67599e-17
c2886 1060:primary_storage 237:vref2 c=2.43225e-20
c2885 1060:primary_storage 333:vref1 c=4.55857e-19
c2884 1060:primary_storage 245:vref2 c=2.29294e-18
c2883 1060:primary_storage m25|src c=2.88785e-19
c2882 1060:primary_storage 599:gnd c=6.89281e-18
c2881 1060:primary_storage 935:net36 c=8.01747e-19
c2880 1060:primary_storage 597:vsec_storage_rf c=1.81316e-17
c2879 1060:primary_storage 595:vsec_storage_rf c=6.73938e-17
c2878 1060:primary_storage 580:vsec_storage_rf c=1.09945e-17
c2877 1060:primary_storage 234:vref2 c=4.95709e-18
c2876 1060:primary_storage 1:ln_n124 c=3.43544e-17
cg2875 1059:primary_storage 0 c=1.11149e-18
c2874 1059:primary_storage 1:ln_n124 c=1.37869e-17
c2873 1059:primary_storage m5@2|gate c=5.42443e-18
c2872 1059:primary_storage 334:vref1 c=2.30756e-17
c2871 1059:primary_storage 255:vref2 c=1.29969e-17
c2870 1059:primary_storage 352:vref1 c=4.65073e-17
cg2869 1058:primary_storage 0 c=4.67502e-18
c2868 1058:primary_storage 934:net36 c=7.98849e-19
c2867 1058:primary_storage m3|gate c=1.06453e-19
c2866 1058:primary_storage 842:gnd c=6.70379e-18
c2865 1058:primary_storage 254:vref2 c=1.47584e-18
c2864 1058:primary_storage 255:vref2 c=2.17124e-17
c2863 1058:primary_storage 334:vref1 c=6.59544e-18
c2862 1058:primary_storage m5@2|gate c=1.93975e-18
c2861 1058:primary_storage 234:vref2 c=1.07161e-18
c2860 1058:primary_storage 1:ln_n124 c=5.34549e-17
cg2859 1057:primary_storage 0 c=1.48086e-18
c2858 1057:primary_storage 238:vref2 c=9.48352e-20
c2857 1057:primary_storage 253:vref2 c=1.32219e-17
c2856 1057:primary_storage 352:vref1 c=1.85337e-17
c2855 1057:primary_storage 333:vref1 c=6.7015e-18
c2854 1057:primary_storage 254:vref2 c=6.18582e-18
c2853 1057:primary_storage m5@2|gate c=1.08559e-18
c2852 1057:primary_storage m5|gate c=1.41729e-18

c2851 1057:primary_storage 234:vref2 c=2.81238e-18
c2850 1057:primary_storage 1:ln_n124 c=7.57841e-18
cg2849 1056:primary_storage 0 c=1.19336e-19
c2848 1056:primary_storage m23@2|gate c=5.56769e-19
c2847 1056:primary_storage 399:load3_cont c=3.74946e-18
c2846 1056:primary_storage m23|gate c=2.53194e-18
c2845 1056:primary_storage 912:gnd c=2.10976e-18
c2844 1056:primary_storage 370:load1_cont c=2.95317e-21
c2843 1056:primary_storage 1:ln_n124 c=1.19706e-18
cg2842 1055:primary_storage 0 c=2.71902e-18
c2841 1055:primary_storage 971:vsec_storage c=1.30132e-20
c2840 1055:primary_storage 333:vref1 c=7.87759e-18
c2839 1055:primary_storage m7|gate c=1.46498e-20
c2838 1055:primary_storage 970:vsec_storage c=2.17914e-17
c2837 1055:primary_storage 1:ln_n124 c=1.20438e-17
cg2836 1054:primary_storage 0 c=1.09528e-19
c2835 1054:primary_storage 1:ln_n124 c=7.37021e-19
c2834 1054:primary_storage 379:load2_cont c=1.02182e-19
c2833 1054:primary_storage 493:load2 c=3.22433e-18
c2832 1054:primary_storage 492:load2 c=2.76241e-17
c2831 1054:primary_storage 509:load1 c=1.73897e-19
c2830 1054:primary_storage m22|gate c=2.53194e-18
c2829 1054:primary_storage m22@2|gate c=5.61189e-19
c2828 1054:primary_storage 378:load2_cont c=4.26152e-18
cg2827 m15|src 0 c=1.0623e-19
c2826 m15|src m6|gate c=3.4437e-20
c2825 m15|src m15|gate c=2.41688e-17
c2824 m15|src 1:ln_n124 c=2.44634e-16
cg2823 1040:primary_storage 0 c=1.97331e-19
c2822 1040:primary_storage 1:ln_n124 c=1.79142e-18
c2821 1040:primary_storage 352:vref1 c=1.08103e-18
c2820 1040:primary_storage 253:vref2 c=1.38908e-18
c2819 1040:primary_storage 234:vref2 c=1.27382e-18
c2818 1040:primary_storage 333:vref1 c=4.84396e-19
cg2817 1037:primary_storage 0 c=2.68021e-19
c2816 1037:primary_storage 1:ln_n124 c=2.38163e-18
c2815 1037:primary_storage 254:vref2 c=2.96894e-18
c2814 1037:primary_storage 352:vref1 c=2.62113e-17
c2813 1037:primary_storage 253:vref2 c=6.64053e-19
c2812 1037:primary_storage m5|gate c=2.44976e-18
c2811 1037:primary_storage 333:vref1 c=2.02497e-18
c2810 1037:primary_storage 234:vref2 c=5.81313e-19
cg2809 1030:primary_storage 0 c=4.64612e-19

c2808 1030:primary_storage 147:ln_n60 c=8.22391e-19
c2807 1030:primary_storage 950:vsec_storage c=8.74488e-21
c2806 1030:primary_storage 334:vref1 c=1.93019e-18
c2805 1030:primary_storage 159:ln_n60 c=1.08838e-18
c2804 1030:primary_storage m15|gate c=2.29883e-19
c2803 1030:primary_storage 1:ln_n124 c=7.66939e-18
cg2802 1024:primary_storage 0 c=1.45728e-19
c2801 1024:primary_storage 734:gnd c=2.08735e-18
c2800 1024:primary_storage 508:load1 c=2.51889e-17
c2799 1024:primary_storage m21@2|gate c=5.36043e-19
c2798 1024:primary_storage 509:load1 c=2.22618e-18
c2797 1024:primary_storage 492:load2 c=1.6909e-19
c2796 1024:primary_storage m21|gate c=2.53194e-18
c2795 1024:primary_storage 370:load1_cont c=3.19174e-18
c2794 1024:primary_storage 1:ln_n124 c=6.31339e-19
cg2793 1011:primary_storage 0 c=6.66282e-18
c2792 1011:primary_storage 583:vsec_storage_rf c=8.60009e-19
c2791 1011:primary_storage 577:vsec_storage_rf c=1.74449e-19
c2790 1011:primary_storage 254:vref2 c=2.97234e-19
c2789 1011:primary_storage 255:vref2 c=2.97234e-19
c2788 1011:primary_storage 334:vref1 c=2.03145e-20
c2787 1011:primary_storage m5@2|gate c=4.14233e-18
c2786 1011:primary_storage m5|drn c=4.30096e-19
c2785 1011:primary_storage 580:vsec_storage_rf c=9.16179e-19
c2784 1011:primary_storage 1:ln_n124 c=4.59365e-16
cg2783 1009:primary_storage 0 c=1.03242e-19
c2782 1009:primary_storage 1:ln_n124 c=9.31805e-19
c2781 1009:primary_storage 352:vref1 c=1.35605e-18
c2780 1009:primary_storage 334:vref1 c=1.02751e-18
cg2779 1008:primary_storage 0 c=1.87215e-19
c2778 1008:primary_storage 186:ln_n64 c=4.646e-20
c2777 1008:primary_storage 701:gnd c=2.11735e-18
c2776 1008:primary_storage m23@2|gate c=6.56303e-18
c2775 1008:primary_storage 399:load3_cont c=2.25418e-17
c2774 1008:primary_storage m23|gate c=2.47813e-18
c2773 1008:primary_storage 912:gnd c=5.10896e-19
c2772 1008:primary_storage 134:ln_n36 c=1.30357e-20
c2771 1008:primary_storage 370:load1_cont c=3.79643e-20
c2770 1008:primary_storage 371:load1_cont c=9.66363e-20
c2769 1008:primary_storage 1:ln_n124 c=1.89636e-18
c2768 1008:primary_storage 739:gnd c=6.53845e-19
cg2767 1007:primary_storage 0 c=2.01999e-18
c2766 1007:primary_storage 147:ln_n60 c=4.23656e-19

c2765 1007:primary_storage 334:vref1 c=3.91117e-18
c2764 1007:primary_storage 350:vref1 c=4.46355e-17
c2763 1007:primary_storage 159:ln_n60 c=2.01329e-18
c2762 1007:primary_storage m15|gate c=1.38214e-17
c2761 1007:primary_storage 1:ln_n124 c=1.41852e-18
cg2760 1005:primary_storage 0 c=3.37548e-19
c2759 1005:primary_storage 734:gnd c=1.59893e-18
c2758 1005:primary_storage 912:gnd c=4.54193e-19
c2757 1005:primary_storage 508:load1 c=2.23199e-17
c2756 1005:primary_storage m21@2|gate c=6.52442e-18
c2755 1005:primary_storage 509:load1 c=6.08568e-17
c2754 1005:primary_storage 492:load2 c=1.11637e-18
c2753 1005:primary_storage m21|gate c=2.73624e-18
c2752 1005:primary_storage 370:load1_cont c=2.0963e-17
c2751 1005:primary_storage 371:load1_cont c=4.2756e-18
c2750 1005:primary_storage 1:ln_n124 c=1.18004e-18
cg2749 1002:primary_storage 0 c=7.62076e-19
c2748 1002:primary_storage 386:load3_cont c=2.23233e-18
c2747 1002:primary_storage 399:load3_cont c=3.3677e-18
c2746 1002:primary_storage 175:ln_n64 c=2.35234e-19
c2745 1002:primary_storage 371:load1_cont c=2.29543e-18
c2744 1002:primary_storage 1:ln_n124 c=1.35382e-18
cg2743 998:primary_storage 0 c=4.53264e-17
c2742 998:primary_storage 539:cont_n1 c=6.03093e-18
c2741 998:primary_storage 913:gnd c=1.91284e-18
c2740 998:primary_storage 923:gnd c=2.0475e-18
c2739 998:primary_storage 734:gnd c=3.53448e-17
c2738 998:primary_storage 147:ln_n60 c=2.60083e-17
c2737 998:primary_storage 880:gnd c=1.39376e-17
c2736 998:primary_storage c1|minus c=8.6167e-18
c2735 998:primary_storage 533:cont_n1 c=3.26118e-17
c2734 998:primary_storage 909:gnd c=2.23657e-18
c2733 998:primary_storage 912:gnd c=1.82154e-17
c2732 998:primary_storage 509:load1 c=5.08721e-19
c2731 998:primary_storage 334:vref1 c=3.31135e-18
c2730 998:primary_storage 350:vref1 c=1.69787e-17
c2729 998:primary_storage 159:ln_n60 c=2.70394e-18
c2728 998:primary_storage 370:load1_cont c=1.78389e-18
c2727 998:primary_storage 519:load1 c=5.64786e-19
c2726 998:primary_storage 1:ln_n124 c=5.1754e-17
c2725 998:primary_storage 739:gnd c=4.89709e-18
cg2724 997:primary_storage 0 c=1.47778e-17
c2723 997:primary_storage 971:vsec_storage c=2.82889e-19

c2722 997:primary_storage 253:vref2 c=1.45369e-17
c2721 997:primary_storage 352:vref1 c=1.85731e-17
c2720 997:primary_storage 953:vsec_storage c=8.85347e-21
c2719 997:primary_storage 338:vref1 c=8.11588e-21
c2718 997:primary_storage 333:vref1 c=2.52665e-17
c2717 997:primary_storage 254:vref2 c=4.78667e-19
c2716 997:primary_storage 335:vref1 c=2.2669e-18
c2715 997:primary_storage 812:gnd c=2.09976e-17
c2714 997:primary_storage 234:vref2 c=1.80549e-17
c2713 997:primary_storage 970:vsec_storage c=6.45605e-18
c2712 997:primary_storage 1:ln_n124 c=4.27648e-17
cg2711 996:primary_storage 0 c=1.01973e-17
c2710 996:primary_storage 950:vsec_storage c=1.42778e-20
c2709 996:primary_storage 147:ln_n60 c=8.76679e-18
c2708 996:primary_storage 694:gnd c=1.82991e-17
c2707 996:primary_storage 352:vref1 c=4.97998e-18
c2706 996:primary_storage 953:vsec_storage c=2.68133e-20
c2705 996:primary_storage 333:vref1 c=4.25856e-18
c2704 996:primary_storage 254:vref2 c=5.36405e-21
c2703 996:primary_storage 255:vref2 c=5.63012e-20
c2702 996:primary_storage 954:vsec_storage c=1.79423e-18
c2701 996:primary_storage 334:vref1 c=9.32289e-18
c2700 996:primary_storage 812:gnd c=6.18691e-19
c2699 996:primary_storage 159:ln_n60 c=8.35585e-19
c2698 996:primary_storage 234:vref2 c=3.90192e-18
c2697 996:primary_storage 970:vsec_storage c=3.48362e-18
c2696 996:primary_storage 972:vsec_storage c=5.31672e-18
c2695 996:primary_storage 1:ln_n124 c=2.94844e-17
cg2694 994:primary_storage 0 c=2.14579e-17
c2693 994:primary_storage c16|minus c=1.28131e-16
c2692 994:primary_storage 226:ln_n46 c=5.78227e-17
c2691 994:primary_storage 391:load3_cont c=1.00142e-20
c2690 994:primary_storage 373:load2_cont c=9.44726e-19
c2689 994:primary_storage 386:load3_cont c=3.25246e-17
c2688 994:primary_storage 385:load3_cont c=6.87031e-17
c2687 994:primary_storage c1|plus c=2.44897e-20
c2686 994:primary_storage 186:ln_n64 c=2.33696e-17
c2685 994:primary_storage 701:gnd c=1.01077e-17
c2684 994:primary_storage 640:gnd c=4.07275e-18
c2683 994:primary_storage 174:ln_n47 c=2.81357e-18
c2682 994:primary_storage 398:load3_cont c=4.49558e-18
c2681 994:primary_storage 175:ln_n64 c=3.30543e-16
c2680 994:primary_storage 622:gnd c=7.75012e-19

c2679 994:primary_storage 170:ln_n47 c=5.30937e-17
c2678 994:primary_storage 625:gnd c=5.30819e-17
c2677 994:primary_storage 600:gnd c=1.25211e-18
c2676 994:primary_storage 637:gnd c=4.19131e-19
c2675 994:primary_storage 509:load1 c=5.57021e-19
c2674 994:primary_storage 601:gnd c=1.26136e-18
c2673 994:primary_storage 365:load1_cont c=5.03692e-18
c2672 994:primary_storage 371:load1_cont c=2.11574e-17
c2671 994:primary_storage 372:load1_cont c=3.52364e-17
c2670 994:primary_storage 390:load3_cont c=3.05918e-17
c2669 994:primary_storage 230:ln_n46 c=3.46271e-18
c2668 994:primary_storage 1:ln_n124 c=2.79644e-17
c2667 994:primary_storage 123:ln_n36 c=4.16448e-18
c2666 990:primary_storage 187:ln_n67 c=3.27498e-18
c2665 990:primary_storage 378:load2_cont c=2.22277e-18
c2664 990:primary_storage 147:ln_n60 c=1.12454e-18
c2663 990:primary_storage c1|plus c=9.37704e-19
c2662 990:primary_storage c1|minus c=1.43457e-18
c2661 990:primary_storage 175:ln_n64 c=2.53591e-18
c2660 990:primary_storage 912:gnd c=2.77628e-18
c2659 990:primary_storage 509:load1 c=2.9248e-18
c2658 990:primary_storage 379:load2_cont c=1.68301e-18
c2657 990:primary_storage 492:load2 c=3.71888e-18
c2656 990:primary_storage 493:load2 c=6.14071e-19
c2655 990:primary_storage 503:load2 c=8.73903e-19
c2654 990:primary_storage 370:load1_cont c=3.45767e-18
c2653 990:primary_storage 371:load1_cont c=3.49357e-19
c2652 990:primary_storage 1:ln_n124 c=4.52588e-18
cg2651 989:primary_storage 0 c=7.82687e-18
c2650 989:primary_storage 934:net36 c=7.09312e-19
c2649 989:primary_storage 352:vref1 c=9.54265e-19
c2648 989:primary_storage 577:vsec_storage_rf c=2.45119e-18
c2647 989:primary_storage 842:gnd c=4.07968e-19
c2646 989:primary_storage 254:vref2 c=8.45584e-18
c2645 989:primary_storage 255:vref2 c=9.66341e-18
c2644 989:primary_storage m5@2|gate c=2.84497e-18
c2643 989:primary_storage 580:vsec_storage_rf c=1.13838e-17
c2642 989:primary_storage 234:vref2 c=9.51373e-18
c2641 989:primary_storage 1:ln_n124 c=3.28595e-17
cg2640 m5|src 0 c=2.02748e-19
c2639 m5|src 1:ln_n124 c=2.2691e-16
c2638 m5|src 254:vref2 c=5.20762e-19
c2637 m5|src 253:vref2 c=1.65258e-18

c2636 m5|src 333:vref1 c=1.51318e-18
c2635 m5|src m1|drn c=4.11171e-19
c2634 m5|src m1|gate c=1.46189e-19
c2633 m5|src m5@2|gate c=9.0448e-18
c2632 m21|src 1:ln_n124 c=2.42134e-16
c2631 m21|src 509:load1 c=1.19016e-18
c2630 m21|src 492:load2 c=4.29333e-19
c2629 m21|src m21@2|gate c=2.04112e-17
c2628 m21|src m21|gate c=4.18706e-18
c2627 m21|src m22@2|gate c=3.33945e-19
c2626 m21|src m22|gate c=8.49347e-21
c2625 m22|src 1:ln_n124 c=2.41675e-16
c2624 m22|src 509:load1 c=6.4647e-19
c2623 m22|src 492:load2 c=1.1944e-18
c2622 m22|src m21@2|gate c=3.25452e-19
c2621 m22|src m21|gate c=1.69212e-20
c2620 m22|src m22@2|gate c=2.01338e-17
c2619 m22|src m22|gate c=4.46447e-18
cg2618 988:primary_storage 0 c=1.67712e-19
c2617 988:primary_storage 378:load2_cont c=2.37264e-17
c2616 988:primary_storage m22@2|gate c=6.74862e-18
c2615 988:primary_storage m22|gate c=2.60029e-18
c2614 988:primary_storage 509:load1 c=1.31328e-18
c2613 988:primary_storage 379:load2_cont c=3.14712e-20
c2612 988:primary_storage 492:load2 c=3.85428e-17
c2611 988:primary_storage 493:load2 c=2.56666e-18
c2610 988:primary_storage 503:load2 c=4.78907e-17
c2609 988:primary_storage 371:load1_cont c=4.09778e-18
c2608 988:primary_storage 1:ln_n124 c=1.61052e-18
cg2607 m20|drn 0 c=1.02978e-19
c2606 m20|drn 918:gnd c=4.63665e-19
c2605 m20|drn m4|src c=5.1383e-18
c2604 m20|drn m20|gate c=1.11193e-17
c2603 m20|drn 597:vsec_storage_rf c=4.72393e-19
c2602 m20|drn 1:ln_n124 c=9.64853e-17
cg2601 m5@2|src 0 c=4.86755e-19
c2600 m5@2|src m14|src c=8.41596e-24
c2599 m5@2|src 255:vref2 c=4.98961e-19
c2598 m5@2|src 334:vref1 c=2.13571e-18
c2597 m5@2|src m5@2|gate c=9.06199e-18
c2596 m5@2|src 1:ln_n124 c=2.27615e-16
cg2595 m23|src 0 c=5.89604e-24
c2594 m23|src m23@2|gate c=2.02336e-17

c2593 m23|src 869:gnd c=5.58191e-19
c2592 m23|src m14|src c=1.9853e-23
c2591 m23|src m23|gate c=4.46447e-18
c2590 m23|src 1:ln_n124 c=2.45748e-16
cg2589 m7|drn 0 c=1.02632e-19
c2588 m7|drn 1:ln_n124 c=1.00748e-16
c2587 m7|drn 971:vsec_storage c=2.19325e-19
c2586 m7|drn m0|src c=8.74171e-23
c2585 m7|drn m0|drn c=9.51971e-19
c2584 m7|drn m15|gate c=1.5054e-19
c2583 m7|drn m7|gate c=1.11275e-17
cg2582 977:primary_storage 0 c=1.49099e-17
c2581 977:primary_storage c16|minus c=4.94372e-17
c2580 977:primary_storage 386:load3_cont c=4.43713e-19
c2579 977:primary_storage 385:load3_cont c=2.4607e-17
c2578 977:primary_storage 642:gnd c=3.38954e-18
c2577 977:primary_storage 910:gnd c=1.79536e-17
c2576 977:primary_storage 701:gnd c=1.67374e-17
c2575 977:primary_storage 640:gnd c=1.71099e-17
c2574 977:primary_storage 174:ln_n47 c=1.65081e-17
c2573 977:primary_storage 398:load3_cont c=1.05816e-19
c2572 977:primary_storage 212:ln_n45 c=6.7926e-18
c2571 977:primary_storage 170:ln_n47 c=4.58895e-17
c2570 977:primary_storage 625:gnd c=7.87443e-19
c2569 977:primary_storage 650:gnd c=7.83806e-19
c2568 977:primary_storage 600:gnd c=9.52176e-19
c2567 977:primary_storage 134:ln_n36 c=7.21871e-18
c2566 977:primary_storage 201:ln_n45 c=2.12473e-17
c2565 977:primary_storage 911:gnd c=8.20296e-19
c2564 977:primary_storage 1:ln_n124 c=1.632e-17
c2563 977:primary_storage 123:ln_n36 c=2.77734e-17
c2562 977:primary_storage 388:load3_cont c=2.38856e-17
c2561 977:primary_storage 634:gnd c=6.28177e-19
cg2560 976:primary_storage 0 c=5.2641e-18
c2559 976:primary_storage 391:load3_cont c=5.72098e-19
c2558 976:primary_storage 386:load3_cont c=6.9363e-18
c2557 976:primary_storage 385:load3_cont c=7.77784e-19
c2556 976:primary_storage 186:ln_n64 c=3.67051e-18
c2555 976:primary_storage 399:load3_cont c=6.94125e-18
c2554 976:primary_storage 398:load3_cont c=9.42322e-19
c2553 976:primary_storage 175:ln_n64 c=1.05316e-17
c2552 976:primary_storage 625:gnd c=4.65912e-19
c2551 976:primary_storage 371:load1_cont c=4.3421e-19

c2550 976:primary_storage 1:ln_n124 c=7.08746e-18
c2549 976:primary_storage 123:ln_n36 c=7.88017e-20
cg2548 975:primary_storage 0 c=6.50297e-18
c2547 975:primary_storage 187:ln_n67 c=2.57597e-17
c2546 975:primary_storage 378:load2_cont c=4.16555e-20
c2545 975:primary_storage 734:gnd c=9.36283e-18
c2544 975:primary_storage 147:ln_n60 c=1.05565e-17
c2543 975:primary_storage c1|minus c=1.78069e-17
c2542 975:primary_storage 399:load3_cont c=5.09064e-18
c2541 975:primary_storage 533:cont_n1 c=3.22778e-20
c2540 975:primary_storage 175:ln_n64 c=3.41115e-17
c2539 975:primary_storage 912:gnd c=6.23405e-18
c2538 975:primary_storage 508:load1 c=1.46424e-17
c2537 975:primary_storage 509:load1 c=2.76133e-17
c2536 975:primary_storage 379:load2_cont c=3.90922e-19
c2535 975:primary_storage 492:load2 c=2.37939e-17
c2534 975:primary_storage 503:load2 c=1.04183e-17
c2533 975:primary_storage 493:load2 c=6.03947e-19
c2532 975:primary_storage 370:load1_cont c=2.57896e-17
c2531 975:primary_storage 371:load1_cont c=9.91888e-18
c2530 975:primary_storage 193:ln_n67 c=2.31338e-18
c2529 975:primary_storage 1:ln_n124 c=4.17904e-17
c2528 974:primary_storage 391:load3_cont c=2.58799e-18
c2527 974:primary_storage 386:load3_cont c=2.31438e-17
c2526 974:primary_storage 385:load3_cont c=4.05636e-19
c2525 974:primary_storage 701:gnd c=4.94187e-19
c2524 974:primary_storage 399:load3_cont c=9.37641e-18
c2523 974:primary_storage 398:load3_cont c=5.55935e-19
c2522 974:primary_storage 533:cont_n1 c=1.65086e-18
c2521 974:primary_storage 175:ln_n64 c=2.32035e-20
c2520 974:primary_storage 912:gnd c=5.14799e-18
c2519 974:primary_storage 371:load1_cont c=1.28081e-19
c2518 974:primary_storage 1:ln_n124 c=8.39742e-18
cg2517 973:primary_storage 0 c=1.62481e-17
c2516 973:primary_storage 221:ln_n33 c=1.83488e-18
c2515 973:primary_storage 400:ln_n35 c=1.83488e-18
c2514 973:primary_storage c16|minus c=5.43872e-19
c2513 973:primary_storage c18|minus c=5.9379e-18
c2512 973:primary_storage 386:load3_cont c=2.47306e-20
c2511 973:primary_storage 164:ln_n42 c=6.17403e-19
c2510 973:primary_storage 698:gnd c=3.68693e-19
c2509 973:primary_storage 910:gnd c=3.13738e-18
c2508 973:primary_storage 704:gnd c=1.24442e-17

c2507 973:primary_storage 68:ln_n32 c=5.00062e-19
c2506 973:primary_storage 169:ln_n44 c=4.99408e-19
c2505 973:primary_storage 118:ln_n34 c=1.83488e-18
c2504 973:primary_storage 671:gnd c=8.71663e-19
c2503 973:primary_storage 34:ln_n27 c=3.0037e-19
c2502 973:primary_storage c19|minus c=3.66976e-18
c2501 973:primary_storage 134:ln_n36 c=2.38084e-17
c2500 973:primary_storage 201:ln_n45 c=1.27644e-18
c2499 973:primary_storage 165:ln_n44 c=8.46965e-18
c2498 973:primary_storage 160:ln_n42 c=6.09925e-18
c2497 973:primary_storage 687:gnd c=1.17459e-18
c2496 973:primary_storage 95:ln_n37 c=1.6295e-17
c2495 973:primary_storage gnd_1 c=4.89745e-18
c2494 973:primary_storage 1:ln_n124 c=2.33354e-17
c2493 973:primary_storage 87:ln_n37 c=2.23216e-16
c2492 973:primary_storage 123:ln_n36 c=3.20619e-16
c2491 973:primary_storage 388:load3_cont c=6.60611e-18
c2490 973:primary_storage 23:ln_n27 c=3.99197e-18
c2489 973:primary_storage 634:gnd c=8.9854e-19
cg2488 c18|plus 0 c=4.97046e-18
c2487 c18|plus 400:ln_n35 c=8.17937e-18
c2486 c18|plus c18|minus c=4.67853e-16
c2485 c18|plus 704:gnd c=7.6738e-17
c2484 c18|plus 647:gnd c=1.61004e-17
c2483 c18|plus 602:gnd c=1.02554e-17
c2482 c18|plus 603:gnd c=8.0502e-18
c2481 c18|plus 646:gnd c=1.37855e-17
c2480 c18|plus 649:gnd c=1.37855e-17
c2479 c18|plus 160:ln_n42 c=8.17937e-18
c2478 c18|plus gnd_1 c=3.66976e-18
c2477 c18|plus 1:ln_n124 c=1.54983e-17
cg2476 c8|plus 0 c=7.03167e-18
c2475 c8|plus 604:gnd c=2.09981e-18
c2474 c8|plus 636:gnd c=1.23569e-17
c2473 c8|plus 221:ln_n33 c=4.63672e-17
c2472 c8|plus 400:ln_n35 c=4.63672e-17
c2471 c8|plus c16|minus c=6.13516e-17
c2470 c8|plus 605:gnd c=2.07486e-18
c2469 c8|plus 122:ln_n34 c=3.19042e-18
c2468 c8|plus 682:gnd c=1.0143e-17
c2467 c8|plus 630:gnd c=1.37468e-17
c2466 c8|plus 417:ln_n43 c=6.1914e-17
c2465 c8|plus c8|minus c=5.91826e-16

c2464 c8|plus 633:gnd c=1.37468e-17
c2463 c8|plus 72:ln_n32 c=2.83425e-19
c2462 c8|plus c18|minus c=1.22189e-16
c2461 c8|plus 164:ln_n42 c=3.63448e-18
c2460 c8|plus 698:gnd c=3.66976e-18
c2459 c8|plus 910:gnd c=8.17116e-19
c2458 c8|plus 704:gnd c=5.16091e-18
c2457 c8|plus 212:ln_n45 c=2.49827e-17
c2456 c8|plus 68:ln_n32 c=4.10793e-18
c2455 c8|plus 169:ln_n44 c=3.13508e-18
c2454 c8|plus 118:ln_n34 c=4.63672e-17
c2453 c8|plus 671:gnd c=4.69611e-19
c2452 c8|plus 647:gnd c=1.49451e-18
c2451 c8|plus c19|minus c=8.42263e-17
c2450 c8|plus 650:gnd c=5.34768e-17
c2449 c8|plus 602:gnd c=2.30821e-18
c2448 c8|plus 201:ln_n45 c=3.25979e-16
c2447 c8|plus 404:ln_n35 c=3.19042e-18
c2446 c8|plus 603:gnd c=2.09663e-18
c2445 c8|plus 165:ln_n44 c=5.57288e-17
c2444 c8|plus 160:ln_n42 c=5.45126e-17
c2443 c8|plus 687:gnd c=4.28252e-18
c2442 c8|plus 631:gnd c=1.75961e-17
c2441 c8|plus 225:ln_n33 c=3.19042e-18
c2440 c8|plus gnd_1 c=7.72057e-17
c2439 c8|plus 1:ln_n124 c=3.32944e-17
c2438 c8|plus 656:gnd c=3.30975e-17
c2437 c8|plus 87:ln_n37 c=9.17441e-18
c2436 c8|plus 123:ln_n36 c=1.31633e-17
c2435 c8|plus 421:ln_n43 c=3.66124e-18
c2434 c8|plus 23:ln_n27 c=5.00062e-19
c2433 c8|plus 634:gnd c=4.36329e-17
c2432 c8|plus 388:load3_cont c=1.06178e-18
cg2431 c16|plus 0 c=3.46174e-18
c2430 c16|plus c16|minus c=5.27996e-16
c2429 c16|plus 226:ln_n46 c=6.07229e-18
c2428 c16|plus 385:load3_cont c=2.53397e-17
c2427 c16|plus 175:ln_n64 c=2.58365e-18
c2426 c16|plus 622:gnd c=1.56316e-17
c2425 c16|plus 170:ln_n47 c=6.07229e-18
c2424 c16|plus 600:gnd c=1.0111e-17
c2423 c16|plus 601:gnd c=7.81582e-18
c2422 c16|plus 621:gnd c=1.38484e-17

c2421 c16|plus 624:gnd c=1.38485e-17
c2420 c16|plus 1:ln_n124 c=9.21215e-18
c2419 c16|plus 388:load3_cont c=1.58346e-17
cg2418 c17|plus 0 c=3.04783e-17
c2417 c17|plus 604:gnd c=9.13635e-18
c2416 c17|plus 221:ln_n33 c=8.17937e-18
c2415 c17|plus 605:gnd c=8.0502e-18
c2414 c17|plus 18:ln_n26 c=6.31884e-17
c2413 c17|plus 655:gnd c=1.3749e-17
c2412 c17|plus 72:ln_n32 c=2.86342e-18
c2411 c17|plus c18|minus c=5.89671e-16
c2410 c17|plus 704:gnd c=3.8369e-17
c2409 c17|plus 68:ln_n32 c=4.44508e-17
c2408 c17|plus 668:gnd c=1.75961e-17
c2407 c17|plus 118:ln_n34 c=8.17937e-18
c2406 c17|plus 671:gnd c=4.14131e-17
c2405 c17|plus 34:ln_n27 c=2.41133e-17
c2404 c17|plus 606:gnd c=1.10575e-17
c2403 c17|plus 22:ln_n26 c=3.70583e-18
c2402 c17|plus c19|minus c=2.33926e-16
c2401 c17|plus 607:gnd c=1.01517e-17
c2400 c17|plus 667:gnd c=1.37855e-17
c2399 c17|plus 670:gnd c=1.37822e-17
c2398 c17|plus 687:gnd c=2.89176e-17
c2397 c17|plus gnd_1 c=1.83488e-18
c2396 c17|plus c17|minus c=7.71214e-17
c2395 c17|plus 1:ln_n124 c=3.07589e-17
c2394 c17|plus 23:ln_n27 c=3.19498e-16
cg2393 972:vsec_storage 0 c=3.04208e-17
c2392 972:vsec_storage 302:piezo_inp c=5.04593e-19
c2391 972:vsec_storage 287:piezo_inp c=7.83463e-17
c2390 972:vsec_storage 727:gnd c=3.88637e-21
c2389 972:vsec_storage 922:gnd c=1.18012e-18
c2388 972:vsec_storage 694:gnd c=4.58484e-17
c2387 972:vsec_storage 712:gnd c=1.48625e-18
c2386 972:vsec_storage 330:piezo_inn c=7.1453e-19
c2385 972:vsec_storage m30|gate c=3.10554e-19
c2384 972:vsec_storage m30|drn c=5.76707e-20
c2383 972:vsec_storage m29|gate c=7.90255e-19
c2382 972:vsec_storage 307:piezo_inp c=6.24266e-19
c2381 972:vsec_storage 288:piezo_inp c=1.18006e-17
c2380 972:vsec_storage 289:piezo_inp c=3.06125e-18
c2379 972:vsec_storage 306:piezo_inp c=1.03667e-17

c2378 972:vsec_storage piezo_inp c=2.35198e-19
c2377 972:vsec_storage 324:piezo_inn c=2.19781e-18
c2376 972:vsec_storage 313:piezo_inn c=5.68554e-19
c2375 972:vsec_storage 200:ln_n80 c=2.39786e-17
c2374 972:vsec_storage 194:ln_n80 c=8.59851e-18
c2373 972:vsec_storage 1:ln_n124 c=5.40064e-17
cg2372 971:vsec_storage 0 c=1.70377e-17
c2371 971:vsec_storage 610:gnd c=9.19269e-18
c2370 971:vsec_storage 710:gnd c=3.30541e-20
c2369 971:vsec_storage 908:gnd c=6.6535e-18
c2368 971:vsec_storage 812:gnd c=1.90753e-17
c2367 971:vsec_storage 1:ln_n124 c=1.64279e-17
cg2366 970:vsec_storage 0 c=5.72498e-18
c2365 970:vsec_storage 287:piezo_inp c=1.78343e-18
c2364 970:vsec_storage 694:gnd c=7.56287e-19
c2363 970:vsec_storage 305:piezo_inp c=2.77458e-18
c2362 970:vsec_storage m30|gate c=1.48134e-18
c2361 970:vsec_storage m30|drn c=1.65906e-18
c2360 970:vsec_storage 288:piezo_inp c=7.78319e-20
c2359 970:vsec_storage 289:piezo_inp c=1.56814e-17
c2358 970:vsec_storage 306:piezo_inp c=3.37311e-19
c2357 970:vsec_storage 324:piezo_inn c=7.7488e-19
c2356 970:vsec_storage 286:piezo_inp c=6.59658e-18
c2355 970:vsec_storage 1:ln_n124 c=1.51414e-17
cg2354 958:vsec_storage 0 c=1.14398e-17
c2353 958:vsec_storage 302:piezo_inp c=1.09297e-18
c2352 958:vsec_storage 611:gnd c=3.43729e-20
c2351 958:vsec_storage 610:gnd c=5.29469e-19
c2350 958:vsec_storage 305:piezo_inp c=2.16079e-17
c2349 958:vsec_storage m30|gate c=6.20841e-18
c2348 958:vsec_storage 299:piezo_inp c=3.82811e-18
c2347 958:vsec_storage 293:piezo_inp c=2.07086e-17
c2346 958:vsec_storage 307:piezo_inp c=9.78925e-19
c2345 958:vsec_storage m32|gate c=7.08479e-19
c2344 958:vsec_storage 289:piezo_inp c=3.01029e-17
c2343 958:vsec_storage 724:gnd c=3.93201e-21
c2342 958:vsec_storage 908:gnd c=1.38133e-17
c2341 958:vsec_storage 812:gnd c=1.81099e-18
c2340 958:vsec_storage 331:piezo_inn c=2.92488e-18
c2339 958:vsec_storage 324:piezo_inn c=2.31817e-17
c2338 958:vsec_storage 329:piezo_inn c=2.98959e-18
c2337 958:vsec_storage 313:piezo_inn c=1.07808e-18
c2336 958:vsec_storage 286:piezo_inp c=3.97214e-17

c2335 958:vsec_storage 1:ln_n124 c=4.57631e-17
cg2334 957:vsec_storage 0 c=3.89352e-18
c2333 957:vsec_storage 287:piezo_inp c=3.46411e-19
c2332 957:vsec_storage 922:gnd c=6.53281e-19
c2331 957:vsec_storage m30|gate c=1.49221e-18
c2330 957:vsec_storage m30|drn c=9.9873e-18
c2329 957:vsec_storage 289:piezo_inp c=1.11385e-18
c2328 957:vsec_storage 1:ln_n124 c=2.31856e-16
cg2327 956:vsec_storage 0 c=4.00505e-18
c2326 956:vsec_storage 187:ln_n67 c=1.12592e-17
c2325 956:vsec_storage 936:ln_n61 c=2.55178e-18
c2324 956:vsec_storage 700:gnd c=3.70933e-19
c2323 956:vsec_storage 721:gnd c=3.70933e-19
c2322 956:vsec_storage 718:gnd c=1.18607e-17
c2321 956:vsec_storage 719:gnd c=2.46918e-17
c2320 956:vsec_storage 722:gnd c=1.51959e-17
c2319 956:vsec_storage c1|minus c=3.75066e-16
c2318 956:vsec_storage 1:ln_n124 c=1.08256e-17
cg2317 955:vsec_storage 0 c=4.06658e-18
c2316 955:vsec_storage 699:gnd c=3.70933e-19
c2315 955:vsec_storage 936:ln_n61 c=2.55178e-18
c2314 955:vsec_storage 728:gnd c=3.70933e-19
c2313 955:vsec_storage 712:gnd c=1.18607e-17
c2312 955:vsec_storage 716:gnd c=2.46918e-17
c2311 955:vsec_storage 718:gnd c=1.51959e-17
c2310 955:vsec_storage c1|minus c=3.75066e-16
c2309 955:vsec_storage 194:ln_n80 c=1.12592e-17
c2308 955:vsec_storage 1:ln_n124 c=1.07641e-17
cg2307 954:vsec_storage 0 c=1.7404e-17
c2306 954:vsec_storage 302:piezo_inp c=8.62691e-19
c2305 954:vsec_storage 287:piezo_inp c=1.92858e-17
c2304 954:vsec_storage 922:gnd c=6.51047e-18
c2303 954:vsec_storage 694:gnd c=1.56753e-17
c2302 954:vsec_storage 712:gnd c=2.80248e-19
c2301 954:vsec_storage m30|gate c=1.48082e-18
c2300 954:vsec_storage 289:piezo_inp c=2.63359e-17
c2299 954:vsec_storage 286:piezo_inp c=2.99478e-18
c2298 954:vsec_storage 1:ln_n124 c=1.9203e-17
cg2297 953:vsec_storage 0 c=7.40283e-18
c2296 953:vsec_storage 287:piezo_inp c=5.75064e-18
c2295 953:vsec_storage 694:gnd c=4.81358e-18
c2294 953:vsec_storage 330:piezo_inn c=1.14465e-18
c2293 953:vsec_storage 288:piezo_inp c=1.30976e-18

c2292 953:vsec_storage 297:piezo_inp c=1.10312e-18
c2291 953:vsec_storage 306:piezo_inp c=1.56958e-17
c2290 953:vsec_storage piezo_inp c=5.14395e-19
c2289 953:vsec_storage 200:ln_n80 c=2.36073e-19
c2288 953:vsec_storage 1:ln_n124 c=1.07969e-17
cg2287 952:vsec_storage 0 c=3.76712e-18
c2286 952:vsec_storage 948:ln_n61 c=2.13983e-18
c2285 952:vsec_storage 694:gnd c=2.93559e-18
c2284 952:vsec_storage 712:gnd c=1.45408e-19
c2283 952:vsec_storage 330:piezo_inn c=4.85243e-17
c2282 952:vsec_storage m29|gate c=6.32224e-18
c2281 952:vsec_storage 288:piezo_inp c=1.64757e-17
c2280 952:vsec_storage 297:piezo_inp c=4.67082e-19
c2279 952:vsec_storage 306:piezo_inp c=1.69062e-17
c2278 952:vsec_storage 314:piezo_inn c=3.16591e-18
c2277 952:vsec_storage piezo_inp c=1.02721e-18
c2276 952:vsec_storage 1:ln_n124 c=9.77436e-18
cg2275 950:vsec_storage 0 c=1.40985e-17
c2274 950:vsec_storage 1:ln_n124 c=3.00887e-17
c2273 950:vsec_storage 306:piezo_inp c=1.34299e-20
c2272 950:vsec_storage 712:gnd c=4.03029e-17
c2271 950:vsec_storage 694:gnd c=2.2097e-17
cg2270 949:vsec_storage 0 c=7.05124e-18
c2269 949:vsec_storage 948:ln_n61 c=5.64084e-19
c2268 949:vsec_storage 936:ln_n61 c=8.21095e-18
c2267 949:vsec_storage 694:gnd c=4.05287e-17
c2266 949:vsec_storage 712:gnd c=1.43993e-18
c2265 949:vsec_storage 306:piezo_inp c=2.9732e-18
c2264 949:vsec_storage 194:ln_n80 c=8.44989e-18
c2263 949:vsec_storage 200:ln_n80 c=5.28845e-19
c2262 949:vsec_storage 1:ln_n124 c=1.02523e-17
c2261 m29|src 1:ln_n124 c=2.36971e-16
c2260 m29|src 200:ln_n80 c=9.59401e-20
c2259 m29|src 297:piezo_inp c=2.29115e-19
c2258 m29|src 288:piezo_inp c=1.12234e-18
c2257 m29|src m30|drn c=4.94194e-19
c2256 m29|src m29|gate c=1.19305e-17
c2255 m29|src m31|drn c=7.9368e-23
c2254 m29|src m30|gate c=4.26059e-20
c2253 m30|src 738:gnd c=1.09629e-19
c2252 m30|src m30|gate c=1.1947e-17
c2251 m30|src m29|gate c=2.14288e-20
c2250 m30|src m29|drn c=5.36307e-19

c2249 m30|src 307:piezo_inp c=4.32125e-19
c2248 m30|src m32|src c=7.9368e-23
c2247 m30|src m32|gate c=2.82717e-19
c2246 m30|src m32|drn c=7.67449e-19
c2245 m30|src 324:piezo_inn c=1.61466e-18
c2244 m30|src 1:ln_n124 c=2.34558e-16
cg2243 m7|src 0 c=2.4944e-19
c2242 m7|src 1:ln_n124 c=9.94784e-17
c2241 m7|src m32|drn c=5.96365e-19
c2240 m7|src m32|gate c=2.1052e-19
cg2239 m7|gate 0 c=6.40668e-18
c2238 m7|gate m31|src c=1.164e-19
c2237 m7|gate 610:gnd c=2.77357e-19
c2236 m7|gate 299:piezo_inp c=2.41978e-19
c2235 m7|gate 293:piezo_inp c=4.93877e-19
c2234 m7|gate m32|src c=8.05185e-19
c2233 m7|gate m32|gate c=4.20164e-19
c2232 m7|gate m32|drn c=1.86176e-19
c2231 m7|gate 908:gnd c=9.28003e-19
c2230 m7|gate 331:piezo_inn c=7.7112e-20
c2229 m7|gate 1:ln_n124 c=6.70838e-17
cg2228 c1|plus 0 c=3.44512e-17
c2227 c1|plus 948:ln_n61 c=2.57688e-17
c2226 c1|plus 187:ln_n67 c=5.87694e-17
c2225 c1|plus 922:gnd c=2.00705e-20
c2224 c1|plus 936:ln_n61 c=3.48996e-16
c2223 c1|plus 694:gnd c=1.53524e-18
c2222 c1|plus 712:gnd c=1.12109e-17
c2221 c1|plus 716:gnd c=7.31654e-18
c2220 c1|plus 718:gnd c=8.18865e-18
c2219 c1|plus 719:gnd c=7.31654e-18
c2218 c1|plus 730:gnd c=2.00705e-20
c2217 c1|plus 722:gnd c=8.18865e-18
c2216 c1|plus 921:gnd c=5.57831e-18
c2215 c1|plus c1|minus c=1.10724e-16
c2214 c1|plus 200:ln_n80 c=3.78318e-18
c2213 c1|plus 194:ln_n80 c=5.03394e-17
c2212 c1|plus 193:ln_n67 c=4.1337e-18
c2211 c1|plus 1:ln_n124 c=8.72912e-18
cg2210 948:ln_n61 0 c=5.22085e-17
c2209 948:ln_n61 922:gnd c=5.84691e-21
c2208 948:ln_n61 699:gnd c=4.67752e-20
c2207 948:ln_n61 694:gnd c=6.13724e-18

c2206 948:ln_n61 728:gnd c=4.67752e-20
c2205 948:ln_n61 712:gnd c=9.0412e-17
c2204 948:ln_n61 700:gnd c=4.67752e-20
c2203 948:ln_n61 716:gnd c=4.84729e-17
c2202 948:ln_n61 718:gnd c=4.86724e-17
c2201 948:ln_n61 721:gnd c=4.67752e-20
c2200 948:ln_n61 719:gnd c=4.84159e-17
c2199 948:ln_n61 722:gnd c=4.72883e-17
c2198 948:ln_n61 730:gnd c=5.84691e-21
c2197 948:ln_n61 921:gnd c=5.29415e-17
c2196 948:ln_n61 c1|minus c=1.94325e-19
c2195 948:ln_n61 518:load1 c=4.3342e-17
c2194 948:ln_n61 510:load1 c=2.46479e-18
c2193 948:ln_n61 520:load1 c=2.5718e-18
c2192 948:ln_n61 506:load1 c=1.8688e-17
c2191 948:ln_n61 517:load1 c=7.44819e-17
c2190 948:ln_n61 1:ln_n124 c=8.09533e-17
cg2189 936:ln_n61 0 c=8.72822e-17
c2188 936:ln_n61 694:gnd c=1.85545e-17
c2187 936:ln_n61 712:gnd c=7.32555e-18
c2186 936:ln_n61 716:gnd c=3.59833e-18
c2185 936:ln_n61 718:gnd c=3.76729e-18
c2184 936:ln_n61 719:gnd c=3.59833e-18
c2183 936:ln_n61 722:gnd c=3.76729e-18
c2182 936:ln_n61 921:gnd c=4.67716e-18
c2181 936:ln_n61 c1|minus c=7.08669e-18
c2180 936:ln_n61 518:load1 c=8.12425e-18
c2179 936:ln_n61 520:load1 c=2.26383e-17
c2178 936:ln_n61 510:load1 c=5.34958e-19
c2177 936:ln_n61 506:load1 c=2.8902e-18
c2176 936:ln_n61 517:load1 c=1.21085e-17
c2175 936:ln_n61 1:ln_n124 c=3.29428e-17
cg2174 935:net36 0 c=7.91114e-18
c2173 935:net36 m2|gate c=4.50226e-18
c2172 935:net36 930:net37 c=3.48528e-17
c2171 935:net36 237:vref2 c=6.917e-18
c2170 935:net36 363:vref3 c=3.64042e-17
c2169 935:net36 360:vref3 c=2.07455e-18
c2168 935:net36 356:vref3 c=3.94943e-17
c2167 935:net36 1:ln_n124 c=3.90705e-18
c2166 935:net36 m1|gate c=1.5641e-19
cg2165 934:net36 0 c=2.22705e-18
c2164 934:net36 m2|gate c=1.13684e-19

c2163 934:net36 362:vref3 c=1.9831e-20
c2162 934:net36 361:vref3 c=9.46915e-20
c2161 934:net36 930:net37 c=1.95724e-17
c2160 934:net36 m4|gate c=2.12216e-19
c2159 934:net36 m4|drn c=4.8681e-19
c2158 934:net36 238:vref2 c=3.39789e-20
c2157 934:net36 253:vref2 c=1.18926e-19
c2156 934:net36 929:net37 c=6.73293e-19
c2155 934:net36 237:vref2 c=6.58494e-18
c2154 934:net36 245:vref2 c=6.95663e-18
c2153 934:net36 360:vref3 c=7.81537e-20
c2152 934:net36 356:vref3 c=9.84783e-19
c2151 934:net36 vref3 c=3.32803e-20
c2150 934:net36 234:vref2 c=4.3284e-18
c2149 934:net36 1:ln_n124 c=1.62829e-17
c2148 934:net36 m1|gate c=8.98489e-19
cg2147 m3|drn 0 c=5.896e-22
c2146 m3|drn m1|drn c=5.896e-22
c2145 m3|drn m2|drn c=5.2479e-19
c2144 m3|drn m2|gate c=1.03048e-17
c2143 m3|drn m4|drn c=5.2479e-19
c2142 m3|drn m0|src c=6.95771e-24
c2141 m3|drn 356:vref3 c=2.35438e-18
c2140 m3|drn 1:ln_n124 c=1.84956e-16
cg2139 m3|gate 0 c=1.6691e-18
c2138 m3|gate m2|gate c=1.00403e-17
c2137 m3|gate 361:vref3 c=1.08303e-19
c2136 m3|gate 930:net37 c=4.59751e-18
c2135 m3|gate m4|gate c=1.01196e-17
c2134 m3|gate m4|drn c=8.86959e-18
c2133 m3|gate 929:net37 c=1.13684e-19
c2132 m3|gate 237:vref2 c=4.14306e-19
c2131 m3|gate m0|src c=1.74913e-20
c2130 m3|gate 245:vref2 c=9.47994e-20
c2129 m3|gate 356:vref3 c=1.12597e-18
c2128 m3|gate 1:ln_n124 c=5.24183e-17
c2127 m3|gate m1|gate c=1.93285e-18
cg2126 930:net37 0 c=9.69175e-18
c2125 930:net37 m2|gate c=1.32841e-19
c2124 930:net37 361:vref3 c=2.1161e-19
c2123 930:net37 915:gnd c=2.21248e-17
c2122 930:net37 916:gnd c=1.07168e-17
c2121 930:net37 363:vref3 c=5.13396e-18

c2120 930:net37 599:gnd c=5.04631e-18
c2119 930:net37 356:vref3 c=3.74341e-18
c2118 930:net37 1:ln_n124 c=6.94339e-18
cg2117 929:net37 0 c=6.76223e-18
c2116 929:net37 m2|gate c=6.30856e-19
c2115 929:net37 362:vref3 c=1.36104e-19
c2114 929:net37 361:vref3 c=2.41682e-18
c2113 929:net37 918:gnd c=1.18234e-18
c2112 929:net37 m4|src c=1.34969e-19
c2111 929:net37 915:gnd c=6.51083e-19
c2110 929:net37 916:gnd c=1.66915e-17
c2109 929:net37 363:vref3 c=8.81124e-19
c2108 929:net37 599:gnd c=2.06545e-17
c2107 929:net37 674:gnd c=1.98527e-17
c2106 929:net37 812:gnd c=1.2223e-17
c2105 929:net37 360:vref3 c=4.01701e-18
c2104 929:net37 356:vref3 c=1.32437e-17
c2103 929:net37 vref3 c=1.31223e-18
c2102 929:net37 1:ln_n124 c=9.74675e-18
cg2101 m4|drn 0 c=1.94373e-18
c2100 m4|drn m2|drn c=6.28956e-22
c2099 m4|drn 361:vref3 c=4.48239e-19
c2098 m4|drn m4|src c=1.0749e-19
c2097 m4|drn 1:ln_n124 c=1.85621e-16
cg2096 m4|gate 0 c=8.20894e-18
c2095 m4|gate m2|gate c=1.92836e-18
c2094 m4|gate m4|src c=1.128e-17
c2093 m4|gate m27|src c=7.29447e-20
c2092 m4|gate 915:gnd c=2.72605e-18
c2091 m4|gate 916:gnd c=1.29907e-18
c2090 m4|gate 599:gnd c=2.10962e-18
c2089 m4|gate 674:gnd c=1.62643e-18
c2088 m4|gate 360:vref3 c=2.1431e-19
c2087 m4|gate 356:vref3 c=3.60585e-19
c2086 m4|gate 1:ln_n124 c=5.8313e-17
cg2085 925:gnd 0 c=1.82757e-18
c2084 925:gnd 108:ln_n52 c=5.84691e-21
c2083 925:gnd c21|plus c=5.29354e-18
c2082 925:gnd 575:vsec_storage_rf c=1.94758e-18
c2081 925:gnd 59:ln_n48 c=5.93513e-17
c2080 925:gnd 567:rf_inn c=1.61333e-19
c2079 925:gnd 79:ln_n56 c=5.57507e-17
c2078 925:gnd 598:vsec_storage_rf c=6.58295e-20

c2077 925:gnd 594:vsec_storage_rf c=2.89605e-17
c2076 925:gnd 578:vsec_storage_rf c=1.36778e-18
c2075 925:gnd 580:vsec_storage_rf c=1.4174e-19
c2074 925:gnd 47:ln_n48 c=4.84952e-18
c2073 925:gnd 1:ln_n124 c=9.01211e-18
c2072 925:gnd 73:ln_n56 c=4.22672e-18
cg2071 924:gnd 0 c=2.56747e-18
c2070 924:gnd 108:ln_n52 c=5.99772e-17
c2069 924:gnd 80:ln_n54 c=3.72173e-18
c2068 924:gnd 96:ln_n52 c=3.80435e-18
c2067 924:gnd 60:ln_n55 c=2.84392e-18
c2066 924:gnd c21|plus c=2.00705e-20
c2065 924:gnd 59:ln_n48 c=5.84691e-21
c2064 924:gnd 86:ln_n54 c=5.63732e-17
c2063 924:gnd 67:ln_n55 c=2.04493e-17
c2062 924:gnd 1:ln_n124 c=1.12158e-17
cg2061 923:gnd 0 c=3.31221e-18
c2060 923:gnd 147:ln_n60 c=9.55204e-18
c2059 923:gnd 533:cont_n1 c=2.16928e-18
c2058 923:gnd 159:ln_n60 c=4.98028e-17
c2057 923:gnd 518:load1 c=5.78115e-18
c2056 923:gnd 517:load1 c=4.35868e-20
c2055 923:gnd 1:ln_n124 c=1.12991e-17
cg2054 922:gnd 0 c=2.9479e-18
c2053 922:gnd 147:ln_n60 c=4.87653e-18
c2052 922:gnd 159:ln_n60 c=5.91454e-17
c2051 922:gnd 200:ln_n80 c=5.63732e-17
c2050 922:gnd 194:ln_n80 c=4.70193e-18
c2049 922:gnd 1:ln_n124 c=1.29723e-17
cg2048 921:gnd 0 c=5.00832e-18
c2047 921:gnd 187:ln_n67 c=4.05525e-18
c2046 921:gnd 518:load1 c=4.35425e-17
c2045 921:gnd 159:ln_n60 c=5.84691e-21
c2044 921:gnd 517:load1 c=2.52836e-18
c2043 921:gnd 193:ln_n67 c=5.0792e-17
c2042 921:gnd 1:ln_n124 c=1.00005e-17
cg2041 920:gnd 0 c=7.66915e-18
c2040 920:gnd 1:ln_n124 c=6.82847e-18
c2039 920:gnd 281:rf_inp c=5.51917e-18
c2038 920:gnd 261:rf_inp c=4.15626e-18
c2037 920:gnd 278:rf_inp c=1.41296e-20
c2036 920:gnd 277:rf_inp c=1.27554e-18
c2035 920:gnd 567:rf_inn c=3.07645e-19

cg2034 919:gnd 0 c=2.49612e-17
c2033 919:gnd 1:ln_n124 c=1.17767e-17
c2032 919:gnd 279:rf_inp c=7.70252e-18
c2031 919:gnd 273:rf_inp c=2.82673e-18
cg2030 918:gnd 0 c=7.87699e-19
c2029 918:gnd m20|gate c=1.73201e-18
c2028 918:gnd 573:rf_inn c=2.44243e-19
c2027 918:gnd 579:vsec_storage_rf c=8.70719e-19
c2026 918:gnd 280:rf_inp c=1.48614e-18
c2025 918:gnd 237:vref2 c=8.13556e-20
c2024 918:gnd 570:rf_inn c=5.60189e-21
c2023 918:gnd 549:rf_inn c=3.07978e-19
c2022 918:gnd 574:rf_inn c=1.48614e-18
c2021 918:gnd 571:rf_inn c=1.86594e-19
c2020 918:gnd 597:vsec_storage_rf c=3.20953e-17
c2019 918:gnd 595:vsec_storage_rf c=1.9158e-18
c2018 918:gnd 360:vref3 c=3.33313e-18
c2017 918:gnd 1:ln_n124 c=1.74393e-17
cg2016 917:gnd 0 c=3.12169e-18
c2015 917:gnd m27|gate c=3.57596e-18
c2014 917:gnd m20|gate c=9.28003e-19
c2013 917:gnd 573:rf_inn c=2.50629e-17
c2012 917:gnd 547:rf_inn c=1.29926e-17
c2011 917:gnd 579:vsec_storage_rf c=4.90802e-18
c2010 917:gnd 551:rf_inn c=5.25225e-19
c2009 917:gnd 280:rf_inp c=1.84505e-17
c2008 917:gnd m25|gate c=2.40256e-19
c2007 917:gnd 549:rf_inn c=3.04584e-18
c2006 917:gnd 574:rf_inn c=4.98966e-19
c2005 917:gnd 597:vsec_storage_rf c=6.4241e-18
c2004 917:gnd 595:vsec_storage_rf c=9.1714e-18
c2003 917:gnd 1:ln_n124 c=1.68149e-17
cg2002 916:gnd 0 c=2.42657e-18
c2001 916:gnd m20|gate c=4.04903e-19
c2000 916:gnd 595:vsec_storage_rf c=1.65395e-18
c1999 916:gnd 1:ln_n124 c=6.08112e-18
cg1998 915:gnd 0 c=3.63766e-18
c1997 915:gnd m20|gate c=5.90597e-19
c1996 915:gnd 595:vsec_storage_rf c=2.48131e-18
c1995 915:gnd 1:ln_n124 c=1.03333e-17
cg1994 914:gnd 0 c=2.04253e-17
c1993 914:gnd 108:ln_n52 c=1.53452e-19
c1992 914:gnd m12|drn c=3.06818e-20

c1991 914:gnd 530:cont_n2 c=4.88639e-18
c1990 914:gnd 523:cont_n2 c=2.07342e-17
c1989 914:gnd m24|gate c=2.96788e-18
c1988 914:gnd 1:ln_n124 c=3.05956e-18
cg1987 913:gnd 0 c=7.2246e-18
c1986 913:gnd 539:cont_n1 c=4.63454e-17
c1985 913:gnd m6|gate c=1.55746e-19
c1984 913:gnd m12|gate c=3.52786e-18
c1983 913:gnd m14|gate c=2.35662e-18
c1982 913:gnd m23|gate c=1.41878e-20
c1981 913:gnd 533:cont_n1 c=1.35578e-17
c1980 913:gnd 530:cont_n2 c=4.63142e-19
c1979 913:gnd 523:cont_n2 c=5.03943e-17
c1978 913:gnd 159:ln_n60 c=6.79321e-21
c1977 913:gnd 1:ln_n124 c=1.34475e-17
cg1976 912:gnd 0 c=1.68385e-18
c1975 912:gnd 539:cont_n1 c=1.21911e-18
c1974 912:gnd m13|gate c=1.60665e-18
c1973 912:gnd m23@2|gate c=2.38549e-20
c1972 912:gnd 399:load3_cont c=1.01258e-17
c1971 912:gnd 398:load3_cont c=2.00848e-20
c1970 912:gnd m23|gate c=2.51695e-18
c1969 912:gnd 533:cont_n1 c=6.96371e-17
c1968 912:gnd 508:load1 c=6.3587e-20
c1967 912:gnd m21@2|gate c=1.9022e-18
c1966 912:gnd 509:load1 c=1.00769e-19
c1965 912:gnd m21|drn c=2.41314e-20
c1964 912:gnd 370:load1_cont c=1.5193e-17
c1963 912:gnd 1:ln_n124 c=3.56078e-17
cg1962 911:gnd 0 c=2.28292e-17
c1961 911:gnd 1:ln_n124 c=1.37578e-17
cg1960 910:gnd 0 c=5.13149e-18
c1959 910:gnd 212:ln_n45 c=4.6937e-18
c1958 910:gnd 134:ln_n36 c=4.89277e-17
c1957 910:gnd 371:load1_cont c=1.70674e-19
c1956 910:gnd 123:ln_n36 c=1.09312e-17
c1955 910:gnd 1:ln_n124 c=2.79643e-17
cg1954 909:gnd 0 c=2.00642e-18
c1953 909:gnd m13|drn c=3.44873e-19
c1952 909:gnd 533:cont_n1 c=1.31794e-17
c1951 909:gnd 370:load1_cont c=2.31848e-20
c1950 909:gnd 1:ln_n124 c=1.07324e-17
cg1949 908:gnd 0 c=2.97945e-18

c1948 908:gnd m30|gate c=3.85142e-19
c1947 908:gnd 299:piezo_inp c=2.53454e-17
c1946 908:gnd 293:piezo_inp c=1.29926e-17
c1945 908:gnd 307:piezo_inp c=4.98966e-19
c1944 908:gnd m32|gate c=3.57596e-18
c1943 908:gnd 289:piezo_inp c=5.25225e-19
c1942 908:gnd 331:piezo_inn c=1.84505e-17
c1941 908:gnd 286:piezo_inp c=1.61903e-18
c1940 908:gnd 1:ln_n124 c=1.73779e-17
cg1939 903:gnd 0 c=3.49086e-18
c1938 903:gnd 1:ln_n124 c=7.29119e-18
c1937 903:gnd 281:rf_inp c=2.5867e-17
c1936 903:gnd 574:rf_inn c=1.75674e-17
c1935 903:gnd 280:rf_inp c=4.98966e-19
c1934 903:gnd 277:rf_inp c=2.61574e-19
c1933 903:gnd 261:rf_inp c=2.62311e-18
c1932 903:gnd m28|gate c=3.56694e-18
cg1931 880:gnd 0 c=2.62436e-18
c1930 880:gnd 147:ln_n60 c=2.59503e-18
c1929 880:gnd m13|gate c=7.00815e-19
c1928 880:gnd m6|gate c=3.67402e-18
c1927 880:gnd 533:cont_n1 c=2.62261e-17
c1926 880:gnd 159:ln_n60 c=7.89819e-18
c1925 880:gnd 1:ln_n124 c=6.49108e-18
cg1924 869:gnd 0 c=1.83432e-17
c1923 869:gnd m13|gate c=2.00006e-19
c1922 869:gnd m13|drn c=1.90996e-19
c1921 869:gnd 174:ln_n47 c=2.69978e-18
c1920 869:gnd m23@2|gate c=3.64482e-18
c1919 869:gnd 398:load3_cont c=1.45046e-18
c1918 869:gnd m23|gate c=3.07798e-20
c1917 869:gnd 134:ln_n36 c=4.84515e-18
c1916 869:gnd 1:ln_n124 c=1.04962e-15
cg1915 842:gnd 0 c=1.40361e-17
c1914 842:gnd 108:ln_n52 c=5.17429e-19
c1913 842:gnd 96:ln_n52 c=8.82282e-18
c1912 842:gnd 577:vsec_storage_rf c=1.94495e-17
c1911 842:gnd c21|plus c=1.56359e-18
c1910 842:gnd 575:vsec_storage_rf c=4.5661e-17
c1909 842:gnd 59:ln_n48 c=5.80283e-18
c1908 842:gnd 567:rf_inn c=1.63022e-16
c1907 842:gnd 570:rf_inn c=3.51226e-18
c1906 842:gnd 79:ln_n56 c=2.07347e-17

c1905 842:gnd 598:vsec_storage_rf c=1.8503e-18
c1904 842:gnd 549:rf_inn c=1.63223e-17
c1903 842:gnd 261:rf_inp c=6.80176e-18
c1902 842:gnd rf_inn c=9.7889e-18
c1901 842:gnd 594:vsec_storage_rf c=3.14477e-18
c1900 842:gnd 277:rf_inp c=3.66684e-19
c1899 842:gnd 578:vsec_storage_rf c=2.72393e-17
c1898 842:gnd 47:ln_n48 c=1.62921e-17
c1897 842:gnd 580:vsec_storage_rf c=1.99882e-17
c1896 842:gnd 565:rf_inn c=2.82695e-18
c1895 842:gnd 1:ln_n124 c=6.72392e-17
c1894 842:gnd 73:ln_n56 c=5.64229e-17
cg1893 812:gnd 0 c=5.25685e-17
c1892 812:gnd 351:vref1 c=2.33619e-17
c1891 812:gnd 361:vref3 c=3.38286e-18
c1890 812:gnd 253:vref2 c=1.82899e-18
c1889 812:gnd 252:vref2 c=6.25052e-19
c1888 812:gnd 338:vref1 c=6.12441e-19
c1887 812:gnd 299:piezo_inp c=4.5694e-19
c1886 812:gnd 237:vref2 c=2.64113e-17
c1885 812:gnd 335:vref1 c=4.03674e-17
c1884 812:gnd 256:vref2 c=6.34064e-18
c1883 812:gnd 360:vref3 c=2.20156e-17
c1882 812:gnd 356:vref3 c=1.52504e-17
c1881 812:gnd vref2 c=2.33551e-17
c1880 812:gnd vref3 c=2.19636e-17
c1879 812:gnd 234:vref2 c=7.75462e-18
c1878 812:gnd 286:piezo_inp c=7.30372e-18
c1877 812:gnd 1:ln_n124 c=1.03919e-16
c1876 812:gnd m1|gate c=2.53639e-19
cg1875 770:gnd 0 c=1.20133e-17
c1874 770:gnd 1:ln_n124 c=1.07015e-17
c1873 770:gnd 306:piezo_inp c=1.43491e-18
c1872 770:gnd 315:piezo_inn c=4.1918e-18
cg1871 769:gnd 0 c=6.37338e-18
c1870 769:gnd 1:ln_n124 c=1.66554e-17
c1869 769:gnd 314:piezo_inn c=2.61191e-19
c1868 769:gnd 315:piezo_inn c=3.87409e-18
cg1867 762:gnd 0 c=3.52628e-18
c1866 762:gnd 1:ln_n124 c=9.35302e-19
c1865 762:gnd 5:ln_n128 c=5.70526e-18
c1864 762:gnd 4:ln_n128 c=4.60362e-18
c1863 762:gnd 6:ln_n24 c=4.67787e-18

c1862 762:gnd 68:ln_n32 c=2.84824e-19
cg1861 756:gnd 0 c=1.61046e-17
c1860 756:gnd m27|gate c=5.73699e-18
c1859 756:gnd m27|drn c=3.28177e-19
c1858 756:gnd m28|gate c=4.49473e-18
c1857 756:gnd m28|drn c=8.5326e-20
c1856 756:gnd 280:rf_inp c=2.73811e-19
c1855 756:gnd 275:rf_inp c=6.83836e-20
c1854 756:gnd m25|src c=1.09629e-19
c1853 756:gnd 572:rf_inn c=3.17433e-21
c1852 756:gnd m25|drn c=4.32982e-24
c1851 756:gnd 570:rf_inn c=1.79615e-20
c1850 756:gnd m26|gate c=4.88241e-20
c1849 756:gnd 574:rf_inn c=2.73811e-19
c1848 756:gnd 571:rf_inn c=1.32381e-18
c1847 756:gnd 548:rf_inn c=3.12827e-21
c1846 756:gnd 360:vref3 c=1.27677e-20
c1845 756:gnd 1:ln_n124 c=6.72882e-16
cg1844 753:gnd 0 c=2.72538e-17
c1843 753:gnd 108:ln_n52 c=5.84691e-21
c1842 753:gnd 80:ln_n54 c=4.27435e-18
c1841 753:gnd c21|plus c=5.18944e-18
c1840 753:gnd 59:ln_n48 c=5.99633e-17
c1839 753:gnd 86:ln_n54 c=5.63732e-17
c1838 753:gnd 47:ln_n48 c=4.8974e-18
c1837 753:gnd 1:ln_n124 c=1.01503e-17
cg1836 752:gnd 0 c=1.09072e-18
c1835 752:gnd 1:ln_n124 c=4.16856e-18
c1834 752:gnd 59:ln_n48 c=1.56663e-17
c1833 752:gnd 108:ln_n52 c=4.67752e-20
c1832 752:gnd c21|plus c=5.72429e-18
c1831 752:gnd 47:ln_n48 c=1.1572e-18
cg1830 750:gnd 0 c=1.09072e-18
c1829 750:gnd 1:ln_n124 c=4.16856e-18
c1828 750:gnd 59:ln_n48 c=1.57111e-17
c1827 750:gnd 108:ln_n52 c=4.67752e-20
c1826 750:gnd c21|plus c=5.72429e-18
c1825 750:gnd 47:ln_n48 c=1.1572e-18
cg1824 749:gnd 0 c=1.09072e-18
c1823 749:gnd 1:ln_n124 c=4.16856e-18
c1822 749:gnd 59:ln_n48 c=1.56663e-17
c1821 749:gnd 108:ln_n52 c=4.67752e-20
c1820 749:gnd c21|plus c=5.72429e-18

c1819 749:gnd 47:ln_n48 c=1.1572e-18
cg1818 747:gnd 0 c=3.58418e-17
c1817 747:gnd m27|gate c=2.69486e-18
c1816 747:gnd m28|gate c=7.84758e-19
c1815 747:gnd 573:rf_inn c=1.69586e-18
c1814 747:gnd 547:rf_inn c=1.31404e-18
c1813 747:gnd 280:rf_inp c=1.40402e-17
c1812 747:gnd 275:rf_inp c=1.04918e-18
c1811 747:gnd 569:rf_inn c=1.01465e-19
c1810 747:gnd 572:rf_inn c=1.15049e-20
c1809 747:gnd 570:rf_inn c=1.30896e-19
c1808 747:gnd 281:rf_inp c=4.94166e-19
c1807 747:gnd 549:rf_inn c=6.55031e-19
c1806 747:gnd 574:rf_inn c=1.50273e-17
c1805 747:gnd 571:rf_inn c=2.76666e-18
c1804 747:gnd 261:rf_inp c=1.10681e-17
c1803 747:gnd 548:rf_inn c=1.26843e-20
c1802 747:gnd 360:vref3 c=6.74565e-19
c1801 747:gnd 277:rf_inp c=6.51608e-20
c1800 747:gnd 580:vsec_storage_rf c=9.71592e-21
c1799 747:gnd 1:ln_n124 c=5.75199e-17
cg1798 745:gnd 0 c=4.43678e-18
c1797 745:gnd 108:ln_n52 c=4.94428e-17
c1796 745:gnd 80:ln_n54 c=1.21666e-17
c1795 745:gnd 96:ln_n52 c=3.49678e-18
c1794 745:gnd c21|plus c=1.80312e-17
c1793 745:gnd 59:ln_n48 c=3.30003e-17
c1792 745:gnd 86:ln_n54 c=1.49538e-16
c1791 745:gnd 67:ln_n55 c=5.47794e-18
c1790 745:gnd 47:ln_n48 c=2.61009e-18
c1789 745:gnd 1:ln_n124 c=4.0116e-17
cg1788 742:gnd 0 c=3.3125e-18
c1787 742:gnd 108:ln_n52 c=4.74931e-17
c1786 742:gnd 96:ln_n52 c=3.83446e-18
c1785 742:gnd c21|plus c=2.66103e-17
c1784 742:gnd 59:ln_n48 c=3.29807e-17
c1783 742:gnd 79:ln_n56 c=6.91053e-18
c1782 742:gnd 47:ln_n48 c=2.44113e-18
c1781 742:gnd 1:ln_n124 c=4.00122e-17
cg1780 741:gnd 0 c=3.36522e-18
c1779 741:gnd 108:ln_n52 c=4.85287e-17
c1778 741:gnd 96:ln_n52 c=3.75619e-18
c1777 741:gnd c21|plus c=2.66103e-17

c1776 741:gnd 59:ln_n48 c=3.27904e-17
c1775 741:gnd 86:ln_n54 c=6.91053e-18
c1774 741:gnd 47:ln_n48 c=2.44113e-18
c1773 741:gnd 1:ln_n124 c=4.00847e-17
cg1772 740:gnd 0 c=1.00664e-17
c1771 740:gnd 108:ln_n52 c=4.54181e-20
c1770 740:gnd m13|gate c=2.83499e-22
c1769 740:gnd m12|drn c=8.50913e-21
c1768 740:gnd 533:cont_n1 c=1.84235e-20
c1767 740:gnd 523:cont_n2 c=5.06828e-18
c1766 740:gnd 1:ln_n124 c=4.34702e-18
cg1765 739:gnd 0 c=2.13099e-17
c1764 739:gnd 539:cont_n1 c=2.06682e-18
c1763 739:gnd 108:ln_n52 c=7.74779e-20
c1762 739:gnd m13|gate c=1.64413e-18
c1761 739:gnd m13|drn c=5.3233e-20
c1760 739:gnd 531:cont_n2 c=3.70078e-20
c1759 739:gnd m12|gate c=3.75923e-18
c1758 739:gnd m12|drn c=1.1044e-18
c1757 739:gnd m23|gate c=4.55004e-19
c1756 739:gnd 533:cont_n1 c=3.72619e-17
c1755 739:gnd 521:cont_n2 c=7.63047e-19
c1754 739:gnd 530:cont_n2 c=1.88886e-18
c1753 739:gnd 523:cont_n2 c=1.44325e-16
c1752 739:gnd 1:ln_n124 c=6.07484e-17
cg1751 738:gnd 0 c=1.58603e-17
c1750 738:gnd m31|gate c=4.49473e-18
c1749 738:gnd m31|drn c=8.5326e-20
c1748 738:gnd m30|gate c=5.10091e-20
c1747 738:gnd m30|drn c=4.32982e-24
c1746 738:gnd m29|gate c=1.08752e-20
c1745 738:gnd 307:piezo_inp c=2.73811e-19
c1744 738:gnd m32|gate c=5.73699e-18
c1743 738:gnd m32|drn c=3.28177e-19
c1742 738:gnd 288:piezo_inp c=1.35686e-18
c1741 738:gnd 297:piezo_inp c=2.40193e-20
c1740 738:gnd 335:vref1 c=3.71629e-20
c1739 738:gnd 331:piezo_inn c=2.73811e-19
c1738 738:gnd 329:piezo_inn c=6.83836e-20
c1737 738:gnd 1:ln_n124 c=6.73397e-16
cg1736 735:gnd 0 c=4.58552e-19
c1735 735:gnd 1:ln_n124 c=9.03351e-18
c1734 735:gnd 327:piezo_inn c=9.38734e-18

c1733 735:gnd 335:vref1 c=4.56808e-19
c1732 735:gnd 313:piezo_inn c=1.70605e-18
c1731 735:gnd 314:piezo_inn c=2.66158e-20
c1730 735:gnd m29|gate c=7.02912e-20
c1729 735:gnd m31|gate c=2.01683e-19
c1728 735:gnd 312:piezo_inn c=1.19421e-18
c1727 735:gnd 315:piezo_inn c=3.66209e-19
cg1726 734:gnd 0 c=1.46324e-18
c1725 734:gnd 147:ln_n60 c=2.38075e-18
c1724 734:gnd m13|gate c=5.70432e-18
c1723 734:gnd m13|drn c=1.49768e-18
c1722 734:gnd m6|gate c=7.00815e-19
c1721 734:gnd 533:cont_n1 c=7.94491e-17
c1720 734:gnd 508:load1 c=5.76251e-17
c1719 734:gnd m21@2|gate c=2.15643e-18
c1718 734:gnd 509:load1 c=1.90535e-19
c1717 734:gnd m21|drn c=9.81774e-19
c1716 734:gnd m21|gate c=1.87185e-18
c1715 734:gnd 518:load1 c=2.10055e-18
c1714 734:gnd 159:ln_n60 c=7.27313e-18
c1713 734:gnd 519:load1 c=1.36008e-17
c1712 734:gnd 370:load1_cont c=1.69435e-17
c1711 734:gnd 1:ln_n124 c=3.5219e-17
cg1710 730:gnd 0 c=5.62754e-19
c1709 730:gnd 187:ln_n67 c=4.49235e-18
c1708 730:gnd 147:ln_n60 c=4.65176e-18
c1707 730:gnd m6|gate c=7.36438e-20
c1706 730:gnd 533:cont_n1 c=1.43373e-20
c1705 730:gnd 159:ln_n60 c=5.47193e-17
c1704 730:gnd 193:ln_n67 c=5.09165e-17
c1703 730:gnd 1:ln_n124 c=9.49686e-18
cg1702 728:gnd 0 c=1.05981e-18
c1701 728:gnd 1:ln_n124 c=3.96422e-18
c1700 728:gnd 159:ln_n60 c=1.53041e-17
c1699 728:gnd 147:ln_n60 c=1.22437e-18
cg1698 727:gnd 0 c=3.67738e-17
c1697 727:gnd 287:piezo_inp c=4.54062e-20
c1696 727:gnd m31|gate c=1.66793e-18
c1695 727:gnd 327:piezo_inn c=1.24618e-19
c1694 727:gnd 299:piezo_inp c=1.62444e-18
c1693 727:gnd 293:piezo_inp c=1.31404e-18
c1692 727:gnd 307:piezo_inp c=1.44898e-17
c1691 727:gnd m32|gate c=2.69526e-18

c1690 727:gnd 288:piezo_inp c=2.94483e-18
c1689 727:gnd 297:piezo_inp c=1.50166e-19
c1688 727:gnd 335:vref1 c=4.6891e-19
c1687 727:gnd 331:piezo_inn c=1.44506e-17
c1686 727:gnd 329:piezo_inn c=1.16732e-18
c1685 727:gnd 313:piezo_inn c=1.00855e-17
c1684 727:gnd 286:piezo_inp c=6.14593e-19
c1683 727:gnd 1:ln_n124 c=5.68732e-17
cg1682 724:gnd 0 c=8.94929e-18
c1681 724:gnd 315:piezo_inn c=2.73433e-20
c1680 724:gnd 327:piezo_inn c=1.47284e-18
c1679 724:gnd 307:piezo_inp c=1.48614e-18
c1678 724:gnd 288:piezo_inp c=1.86594e-19
c1677 724:gnd 335:vref1 c=1.0567e-18
c1676 724:gnd 331:piezo_inn c=1.48614e-18
c1675 724:gnd 1:ln_n124 c=1.27264e-17
cg1674 722:gnd 0 c=2.1387e-18
c1673 722:gnd 187:ln_n67 c=1.21632e-17
c1672 722:gnd 147:ln_n60 c=2.44113e-18
c1671 722:gnd 508:load1 c=6.27363e-20
c1670 722:gnd m21|gate c=7.23993e-21
c1669 722:gnd 510:load1 c=1.53768e-19
c1668 722:gnd 518:load1 c=7.549e-18
c1667 722:gnd 159:ln_n60 c=3.2491e-17
c1666 722:gnd 506:load1 c=7.25393e-19
c1665 722:gnd 517:load1 c=1.19848e-18
c1664 722:gnd 193:ln_n67 c=1.47896e-16
c1663 722:gnd 1:ln_n124 c=4.02425e-17
cg1662 721:gnd 0 c=5.69494e-19
c1661 721:gnd 147:ln_n60 c=1.22437e-18
c1660 721:gnd 521:cont_n2 c=9.97053e-22
c1659 721:gnd 159:ln_n60 c=1.55558e-17
c1658 721:gnd 1:ln_n124 c=3.96422e-18
cg1657 719:gnd 0 c=3.55157e-18
c1656 719:gnd 147:ln_n60 c=2.50257e-18
c1655 719:gnd 159:ln_n60 c=3.36798e-17
c1654 719:gnd 506:load1 c=1.57694e-19
c1653 719:gnd 193:ln_n67 c=6.91053e-18
c1652 719:gnd 1:ln_n124 c=4.04817e-17
cg1651 718:gnd 0 c=3.36428e-18
c1650 718:gnd 147:ln_n60 c=2.44113e-18
c1649 718:gnd 159:ln_n60 c=3.2252e-17
c1648 718:gnd 1:ln_n124 c=4.02234e-17

cg1647 716:gnd 0 c=3.32653e-18
c1646 716:gnd 147:ln_n60 c=2.55445e-18
c1645 716:gnd 159:ln_n60 c=3.29862e-17
c1644 716:gnd 200:ln_n80 c=6.91053e-18
c1643 716:gnd 1:ln_n124 c=4.02926e-17
cg1642 712:gnd 0 c=3.87682e-18
c1641 712:gnd 147:ln_n60 c=2.32637e-18
c1640 712:gnd 306:piezo_inp c=6.8147e-20
c1639 712:gnd 159:ln_n60 c=3.38608e-17
c1638 712:gnd 200:ln_n80 c=2.05951e-16
c1637 712:gnd 194:ln_n80 c=1.626e-17
c1636 712:gnd 1:ln_n124 c=4.45791e-17
cg1635 711:gnd 0 c=3.41668e-17
c1634 711:gnd 1:ln_n124 c=3.60016e-17
c1633 711:gnd 335:vref1 c=3.26553e-19
c1632 711:gnd 315:piezo_inn c=7.8943e-19
cg1631 710:gnd 0 c=5.2979e-18
c1630 710:gnd 1:ln_n124 c=1.94664e-17
c1629 710:gnd 331:piezo_inn c=4.98966e-19
c1628 710:gnd 307:piezo_inp c=1.76033e-17
c1627 710:gnd 327:piezo_inn c=2.79839e-17
c1626 710:gnd 313:piezo_inn c=2.62311e-18
c1625 710:gnd 314:piezo_inn c=2.61574e-19
c1624 710:gnd m31|gate c=3.54316e-18
cg1623 709:gnd 0 c=3.39167e-18
c1622 709:gnd 108:ln_n52 c=4.74931e-17
c1621 709:gnd 96:ln_n52 c=3.6655e-18
c1620 709:gnd c21|plus c=2.99685e-17
c1619 709:gnd 59:ln_n48 c=3.30003e-17
c1618 709:gnd 47:ln_n48 c=2.61009e-18
c1617 709:gnd 1:ln_n124 c=3.99952e-17
cg1616 708:gnd 0 c=5.66398e-18
c1615 708:gnd 108:ln_n52 c=9.20764e-17
c1614 708:gnd 96:ln_n52 c=7.11891e-18
c1613 708:gnd 583:vsec_storage_rf c=7.7601e-19
c1612 708:gnd 577:vsec_storage_rf c=4.05717e-18
c1611 708:gnd c21|plus c=1.76803e-17
c1610 708:gnd 59:ln_n48 c=3.29594e-17
c1609 708:gnd 79:ln_n56 c=2.08316e-16
c1608 708:gnd 598:vsec_storage_rf c=8.98447e-21
c1607 708:gnd 594:vsec_storage_rf c=7.949e-19
c1606 708:gnd 580:vsec_storage_rf c=9.10396e-19
c1605 708:gnd 47:ln_n48 c=2.61009e-18

c1604 708:gnd 1:ln_n124 c=5.14334e-17
c1603 708:gnd 73:ln_n56 c=1.68685e-17
cg1602 707:gnd 0 c=1.09072e-18
c1601 707:gnd 1:ln_n124 c=4.16856e-18
c1600 707:gnd 59:ln_n48 c=1.56936e-17
c1599 707:gnd 108:ln_n52 c=4.67752e-20
c1598 707:gnd c21|plus c=5.72429e-18
c1597 707:gnd 47:ln_n48 c=1.1572e-18
cg1596 gnd_1 0 c=3.49657e-17
c1595 gnd_1 221:ln_n33 c=1.83488e-18
c1594 gnd_1 400:ln_n35 c=1.83488e-18
c1593 gnd_1 109:ln_n31 c=2.22757e-16
c1592 gnd_1 135:ln_n30 c=3.19667e-16
c1591 gnd_1 417:ln_n43 c=4.83689e-17
c1590 gnd_1 6:ln_n24 c=4.01835e-18
c1589 gnd_1 68:ln_n32 c=5.00062e-19
c1588 gnd_1 169:ln_n44 c=3.56314e-18
c1587 gnd_1 118:ln_n34 c=1.83488e-18
c1586 gnd_1 17:ln_n24 c=2.99328e-19
c1585 gnd_1 146:ln_n30 c=2.45714e-17
c1584 gnd_1 165:ln_n44 c=5.03597e-17
c1583 gnd_1 160:ln_n42 c=2.15399e-18
c1582 gnd_1 405:ln_n41 c=1.34824e-17
c1581 gnd_1 390:load3_cont c=7.74646e-20
c1580 gnd_1 1:ln_n124 c=1.59962e-17
c1579 gnd_1 421:ln_n43 c=3.5453e-18
c1578 gnd_1 117:ln_n31 c=1.72768e-17
cg1577 704:gnd 0 c=7.14716e-19
c1576 704:gnd 400:ln_n35 c=4.24981e-17
c1575 704:gnd 221:ln_n33 c=4.24981e-17
c1574 704:gnd 122:ln_n34 c=3.11908e-18
c1573 704:gnd 135:ln_n30 c=1.31633e-17
c1572 704:gnd 109:ln_n31 c=9.17441e-18
c1571 704:gnd 72:ln_n32 c=2.83425e-19
c1570 704:gnd 6:ln_n24 c=9.5733e-19
c1569 704:gnd 164:ln_n42 c=3.56314e-18
c1568 704:gnd 68:ln_n32 c=4.10793e-18
c1567 704:gnd 169:ln_n44 c=6.27787e-19
c1566 704:gnd 118:ln_n34 c=4.24981e-17
c1565 704:gnd 404:ln_n35 c=3.11908e-18
c1564 704:gnd 165:ln_n44 c=1.98351e-18
c1563 704:gnd 160:ln_n42 c=5.06894e-17
c1562 704:gnd 225:ln_n33 c=3.11908e-18

c1561 704:gnd 1:ln_n124 c=1.26815e-17
cg1560 c19|minus 0 c=2.82968e-18
c1559 c19|minus 400:ln_n35 c=8.17937e-18
c1558 c19|minus 221:ln_n33 c=1.13283e-16
c1557 c19|minus 122:ln_n34 c=9.12357e-18
c1556 c19|minus 68:ln_n32 c=5.7244e-18
c1555 c19|minus 118:ln_n34 c=1.13012e-16
c1554 c19|minus 225:ln_n33 c=7.74919e-18
c1553 c19|minus 1:ln_n124 c=1.5442e-17
cg1552 701:gnd 0 c=1.59238e-17
c1551 701:gnd 386:load3_cont c=8.22077e-18
c1550 701:gnd 385:load3_cont c=1.87567e-18
c1549 701:gnd 174:ln_n47 c=8.65789e-18
c1548 701:gnd m23@2|gate c=1.99652e-18
c1547 701:gnd 399:load3_cont c=1.4503e-18
c1546 701:gnd 398:load3_cont c=2.72051e-17
c1545 701:gnd 533:cont_n1 c=4.69242e-20
c1544 701:gnd 170:ln_n47 c=1.33047e-18
c1543 701:gnd 1:ln_n124 c=4.54759e-17
c1542 701:gnd 388:load3_cont c=2.37762e-19
cg1541 700:gnd 0 c=1.11981e-18
c1540 700:gnd 1:ln_n124 c=3.96422e-18
c1539 700:gnd 159:ln_n60 c=1.53041e-17
c1538 700:gnd 147:ln_n60 c=1.22437e-18
cg1537 699:gnd 0 c=1.14365e-18
c1536 699:gnd 147:ln_n60 c=1.16873e-18
c1535 699:gnd 159:ln_n60 c=1.55999e-17
c1534 699:gnd 1:ln_n124 c=4.01241e-18
cg1533 698:gnd 0 c=3.80299e-17
c1532 698:gnd 135:ln_n30 c=2.47055e-18
c1531 698:gnd 417:ln_n43 c=2.10413e-18
c1530 698:gnd 416:ln_n41 c=2.50314e-17
c1529 698:gnd 146:ln_n30 c=6.13587e-19
c1528 698:gnd 165:ln_n44 c=2.15399e-18
c1527 698:gnd 405:ln_n41 c=3.29554e-16
c1526 698:gnd 1:ln_n124 c=6.60373e-18
cg1525 694:gnd 0 c=1.76354e-17
c1524 694:gnd 287:piezo_inp c=1.41688e-17
c1523 694:gnd 147:ln_n60 c=6.35662e-17
c1522 694:gnd 352:vref1 c=1.30822e-19
c1521 694:gnd 289:piezo_inp c=1.22497e-18
c1520 694:gnd 288:piezo_inp c=1.11813e-18
c1519 694:gnd 306:piezo_inp c=1.72654e-16

c1518 694:gnd 334:vref1 c=4.48097e-19
c1517 694:gnd 314:piezo_inn c=2.2011e-18
c1516 694:gnd 159:ln_n60 c=4.82095e-18
c1515 694:gnd 506:load1 c=3.07223e-18
c1514 694:gnd 324:piezo_inn c=2.89792e-18
c1513 694:gnd 313:piezo_inn c=1.10857e-17
c1512 694:gnd 200:ln_n80 c=1.94877e-17
c1511 694:gnd 286:piezo_inp c=2.26719e-17
c1510 694:gnd 194:ln_n80 c=1.09328e-16
c1509 694:gnd 1:ln_n124 c=5.87824e-17
cg1508 687:gnd 0 c=1.31214e-16
c1507 687:gnd 221:ln_n33 c=1.42686e-17
c1506 687:gnd 80:ln_n54 c=9.37285e-18
c1505 687:gnd 96:ln_n52 c=8.87555e-18
c1504 687:gnd 540:ln_n125 c=1.85483e-18
c1503 687:gnd 6:ln_n24 c=6.0129e-19
c1502 687:gnd 60:ln_n55 c=1.88086e-17
c1501 687:gnd c21|plus c=1.30966e-18
c1500 687:gnd 68:ln_n32 c=2.06591e-17
c1499 687:gnd 543:ln_n125 c=2.29894e-17
c1498 687:gnd 541:ln_n125 c=2.21828e-19
c1497 687:gnd 5:ln_n128 c=9.36192e-18
c1496 687:gnd 4:ln_n128 c=2.7673e-16
c1495 687:gnd 1:ln_n124 c=6.00181e-17
c1494 687:gnd 87:ln_n37 c=7.3642e-18
c1493 687:gnd 123:ln_n36 c=1.50929e-18
c1492 687:gnd 23:ln_n27 c=2.87503e-17
cg1491 685:gnd 0 c=4.67087e-17
c1490 685:gnd m28|gate c=1.69568e-19
c1489 685:gnd 279:rf_inp c=1.2953e-17
c1488 685:gnd 567:rf_inn c=2.74768e-17
c1487 685:gnd 2:ln_n_generated_129 c=1.84164e-16
c1486 685:gnd 281:rf_inp c=3.07296e-18
c1485 685:gnd 269:rf_inp c=1.56675e-18
c1484 685:gnd m26|gate c=8.46211e-20
c1483 685:gnd 598:vsec_storage_rf c=1.5151e-18
c1482 685:gnd 549:rf_inn c=9.7379e-18
c1481 685:gnd 574:rf_inn c=6.50984e-19
c1480 685:gnd 261:rf_inp c=2.72574e-17
c1479 685:gnd rf_inn c=6.56787e-18
c1478 685:gnd 277:rf_inp c=7.71864e-19
c1477 685:gnd 47:ln_n48 c=2.48365e-18
c1476 685:gnd 565:rf_inn c=7.52064e-18

c1475 685:gnd 1:ln_n124 c=4.53793e-17
cg1474 684:gnd 0 c=4.53729e-17
c1473 684:gnd 315:piezo_inn c=8.26997e-20
c1472 684:gnd 312:piezo_inn c=1.5042e-18
c1471 684:gnd m31|gate c=1.5659e-19
c1470 684:gnd 3:ln_n_generated_130 c=1.58985e-16
c1469 684:gnd 327:piezo_inn c=2.82835e-18
c1468 684:gnd 307:piezo_inp c=5.40731e-19
c1467 684:gnd 306:piezo_inp c=2.67016e-17
c1466 684:gnd 314:piezo_inn c=6.06464e-19
c1465 684:gnd 317:piezo_inn c=1.38501e-17
c1464 684:gnd piezo_inp c=7.92422e-18
c1463 684:gnd 313:piezo_inn c=1.17101e-17
c1462 684:gnd 286:piezo_inp c=9.13077e-18
c1461 684:gnd 1:ln_n124 c=3.73024e-17
cg1460 682:gnd 0 c=9.79713e-19
c1459 682:gnd 164:ln_n42 c=6.56615e-19
c1458 682:gnd 212:ln_n45 c=1.54611e-18
c1457 682:gnd 169:ln_n44 c=1.15465e-16
c1456 682:gnd 416:ln_n41 c=1.54611e-18
c1455 682:gnd 165:ln_n44 c=8.72952e-18
c1454 682:gnd 1:ln_n124 c=2.04709e-17
c1453 m28|src 1:ln_n124 c=1.01716e-16
c1452 m28|src 574:rf_inn c=1.1706e-19
c1451 m28|src m28|gate c=1.16605e-17
c1450 m28|src m26|drn c=7.9368e-23
c1449 m28|src m27|gate c=2.90435e-20
c1448 m28|src m20|gate c=1.164e-19
c1447 m27|src 1:ln_n124 c=9.86969e-17
c1446 m27|src m28|gate c=2.90435e-20
c1445 m27|src m27|gate c=1.16587e-17
c1444 m27|src m20|gate c=8.05185e-19
c1443 m27|src m25|src c=7.9368e-23
cg1442 m4|src 0 c=5.896e-22
c1441 m4|src m27|drn c=8.48175e-20
c1440 m4|src m20|gate c=1.01966e-18
c1439 m4|src m20|src c=3.46509e-22
c1438 m4|src 595:vsec_storage_rf c=8.75616e-20
c1437 m4|src 1:ln_n124 c=1.61417e-16
cg1436 m24|src 0 c=1.66464e-18
c1435 m24|src 108:ln_n52 c=1.7636e-19
c1434 m24|src m12|gate c=3.61209e-20
c1433 m24|src m12|drn c=4.90743e-19

c1432 m24|src 530:cont_n2 c=1.32441e-19
c1431 m24|src 523:cont_n2 c=1.52991e-18
c1430 m24|src m24|gate c=1.16583e-17
c1429 m24|src 1:ln_n124 c=1.01798e-16
cg1428 m12|src 0 c=3.46509e-22
c1427 m12|src m6|gate c=1.05648e-19
c1426 m12|src m12|gate c=1.16803e-17
c1425 m12|src m14|src c=5.1383e-18
c1424 m12|src 533:cont_n1 c=2.26116e-19
c1423 m12|src 523:cont_n2 c=7.04071e-19
c1422 m12|src m24|gate c=5.28542e-20
c1421 m12|src 1:ln_n124 c=1.0025e-16
c1420 m13|src 1:ln_n124 c=9.70301e-17
c1419 m13|src 519:load1 c=5.76078e-20
c1418 m13|src 518:load1 c=4.4354e-20
c1417 m13|src 533:cont_n1 c=1.46584e-18
c1416 m13|src m13|gate c=1.16817e-17
c1415 m13|src m12|drn c=2.82077e-19
cg1414 m6|src 0 c=7.6086e-20
c1413 m6|src 1:ln_n124 c=9.75072e-17
c1412 m6|src 159:ln_n60 c=3.49571e-19
c1411 m6|src 533:cont_n1 c=1.34712e-18
c1410 m6|src m6|gate c=1.168e-17
c1409 m6|src m13|drn c=3.46509e-22
c1408 m6|src m12|gate c=1.88718e-20
c1407 m32|src 1:ln_n124 c=9.87607e-17
c1406 m32|src m31|gate c=2.90435e-20
c1405 m32|src m32|gate c=1.16587e-17
c1404 m32|src m0|gate c=9.65738e-20
c1403 m31|src 1:ln_n124 c=1.02057e-16
c1402 m31|src 307:piezo_inp c=1.1706e-19
c1401 m31|src m31|gate c=1.16605e-17
c1400 m31|src m29|drn c=7.9368e-23
c1399 m31|src m32|gate c=2.90435e-20
cg1398 681:gnd 0 c=4.94418e-17
c1397 681:gnd 1:ln_n124 c=5.5368e-17
c1396 681:gnd 279:rf_inp c=2.86928e-18
c1395 681:gnd 277:rf_inp c=2.44122e-19
c1394 681:gnd 360:vref3 c=3.72255e-19
c1393 681:gnd 273:rf_inp c=2.37863e-18
cg1392 678:gnd 0 c=2.47061e-17
c1391 678:gnd 1:ln_n124 c=3.37502e-17
c1390 678:gnd 281:rf_inp c=2.1169e-18

c1389 678:gnd 597:vsec_storage_rf c=3.30541e-20
cg1388 677:gnd 0 c=6.03062e-18
c1387 677:gnd 1:ln_n124 c=1.27954e-17
c1386 677:gnd 281:rf_inp c=1.04533e-17
c1385 677:gnd 277:rf_inp c=4.08455e-19
c1384 677:gnd 269:rf_inp c=7.35437e-19
cg1383 676:gnd 0 c=3.98523e-19
c1382 676:gnd m28|gate c=2.10053e-19
c1381 676:gnd 579:vsec_storage_rf c=1.52334e-19
c1380 676:gnd 237:vref2 c=9.76267e-20
c1379 676:gnd 281:rf_inp c=3.48383e-18
c1378 676:gnd 269:rf_inp c=1.60944e-19
c1377 676:gnd 278:rf_inp c=1.13888e-20
c1376 676:gnd 597:vsec_storage_rf c=3.00686e-18
c1375 676:gnd 273:rf_inp c=2.15245e-19
c1374 676:gnd 360:vref3 c=2.00784e-18
c1373 676:gnd 277:rf_inp c=9.46404e-19
c1372 676:gnd 1:ln_n124 c=5.7179e-18
cg1371 674:gnd 0 c=3.14029e-19
c1370 674:gnd 1:ln_n124 c=4.52478e-18
c1369 674:gnd 360:vref3 c=4.11497e-18
c1368 674:gnd 237:vref2 c=1.00881e-19
cg1367 673:gnd 0 c=1.63438e-18
c1366 673:gnd 1:ln_n124 c=1.26103e-17
c1365 673:gnd 597:vsec_storage_rf c=8.81609e-18
c1364 673:gnd 573:rf_inn c=4.26916e-19
c1363 673:gnd 579:vsec_storage_rf c=5.29469e-19
c1362 673:gnd m20|gate c=2.77887e-19
cg1361 671:gnd 0 c=1.82267e-17
c1360 671:gnd 18:ln_n26 c=3.82267e-18
c1359 671:gnd 72:ln_n32 c=4.14538e-17
c1358 671:gnd 68:ln_n32 c=3.09487e-18
c1357 671:gnd 34:ln_n27 c=3.04619e-16
c1356 671:gnd 22:ln_n26 c=4.72493e-17
c1355 671:gnd 1:ln_n124 c=4.48734e-17
c1354 671:gnd 23:ln_n27 c=2.34814e-17
cg1353 670:gnd 0 c=1.44155e-18
c1352 670:gnd 1:ln_n124 c=1.98472e-17
c1351 670:gnd 17:ln_n24 c=1.36771e-18
c1350 670:gnd 34:ln_n27 c=1.36771e-18
c1349 670:gnd 72:ln_n32 c=5.51632e-18
cg1348 668:gnd 0 c=1.42934e-18
c1347 668:gnd 1:ln_n124 c=1.96425e-17

c1346 668:gnd 17:ln_n24 c=1.36771e-18
c1345 668:gnd 34:ln_n27 c=1.36771e-18
cg1344 667:gnd 0 c=1.90236e-18
c1343 667:gnd 1:ln_n124 c=1.9385e-17
c1342 667:gnd 17:ln_n24 c=1.36771e-18
c1341 667:gnd 22:ln_n26 c=5.51632e-18
c1340 667:gnd 34:ln_n27 c=1.36771e-18
cg1339 656:gnd 0 c=1.06396e-17
c1338 656:gnd 221:ln_n33 c=3.2874e-18
c1337 656:gnd 122:ln_n34 c=4.14538e-17
c1336 656:gnd 118:ln_n34 c=3.2874e-18
c1335 656:gnd 95:ln_n37 c=2.15372e-16
c1334 656:gnd 225:ln_n33 c=4.14538e-17
c1333 656:gnd 87:ln_n37 c=8.81612e-18
c1332 656:gnd 1:ln_n124 c=3.18837e-17
cg1331 655:gnd 0 c=9.83872e-19
c1330 655:gnd 122:ln_n34 c=5.51632e-18
c1329 655:gnd 95:ln_n37 c=1.36771e-18
c1328 655:gnd 225:ln_n33 c=5.51632e-18
c1327 655:gnd 1:ln_n124 c=2.03176e-17
c1326 655:gnd 117:ln_n31 c=1.36771e-18
cg1325 650:gnd 0 c=7.87027e-18
c1324 650:gnd 400:ln_n35 c=3.2874e-18
c1323 650:gnd 164:ln_n42 c=4.72493e-17
c1322 650:gnd 134:ln_n36 c=3.14372e-16
c1321 650:gnd 404:ln_n35 c=4.14538e-17
c1320 650:gnd 160:ln_n42 c=3.82267e-18
c1319 650:gnd 123:ln_n36 c=1.26492e-17
c1318 650:gnd 1:ln_n124 c=4.6505e-17
cg1317 649:gnd 0 c=9.07203e-19
c1316 649:gnd 1:ln_n124 c=2.03802e-17
c1315 649:gnd 146:ln_n30 c=1.36771e-18
c1314 649:gnd 134:ln_n36 c=1.36771e-18
c1313 649:gnd 164:ln_n42 c=5.51632e-18
cg1312 647:gnd 0 c=6.89354e-19
c1311 647:gnd 1:ln_n124 c=2.03838e-17
c1310 647:gnd 146:ln_n30 c=1.36771e-18
c1309 647:gnd 134:ln_n36 c=1.36771e-18
cg1308 646:gnd 0 c=8.88321e-19
c1307 646:gnd 1:ln_n124 c=2.0399e-17
c1306 646:gnd 146:ln_n30 c=1.36771e-18
c1305 646:gnd 404:ln_n35 c=5.51632e-18
c1304 646:gnd 134:ln_n36 c=1.36771e-18

cg1303 642:gnd 0 c=8.28451e-18
c1302 642:gnd 174:ln_n47 c=1.95602e-19
c1301 642:gnd m23@2|gate c=1.64924e-20
c1300 642:gnd 134:ln_n36 c=3.45077e-18
c1299 642:gnd 1:ln_n124 c=7.79628e-18
cg1298 640:gnd 0 c=2.3942e-18
c1297 640:gnd 174:ln_n47 c=4.99205e-18
c1296 640:gnd 533:cont_n1 c=2.21701e-21
c1295 640:gnd m21@2|drn c=2.90648e-19
c1294 640:gnd 134:ln_n36 c=5.4446e-17
c1293 640:gnd m21@2|gate c=4.34156e-20
c1292 640:gnd 509:load1 c=3.81481e-20
c1291 640:gnd 370:load1_cont c=9.46372e-20
c1290 640:gnd 371:load1_cont c=2.15744e-18
c1289 640:gnd 1:ln_n124 c=3.40939e-17
c1288 640:gnd 123:ln_n36 c=1.10253e-17
cg1287 637:gnd 0 c=2.24835e-17
c1286 637:gnd 417:ln_n43 c=2.4847e-19
c1285 637:gnd 416:ln_n41 c=6.63572e-19
c1284 637:gnd 405:ln_n41 c=5.61486e-18
c1283 637:gnd 390:load3_cont c=2.21189e-18
c1282 637:gnd 1:ln_n124 c=8.91915e-18
c1281 637:gnd 388:load3_cont c=7.00463e-19
cg1280 636:gnd 0 c=1.69784e-17
c1279 636:gnd 417:ln_n43 c=1.31439e-17
c1278 636:gnd 212:ln_n45 c=1.54611e-18
c1277 636:gnd 169:ln_n44 c=4.72176e-17
c1276 636:gnd 416:ln_n41 c=3.20592e-16
c1275 636:gnd 165:ln_n44 c=3.74646e-18
c1274 636:gnd 405:ln_n41 c=1.29559e-17
c1273 636:gnd 1:ln_n124 c=6.10104e-17
c1272 636:gnd 421:ln_n43 c=1.62603e-16
cg1271 634:gnd 0 c=7.72229e-18
c1270 634:gnd 417:ln_n43 c=3.77476e-18
c1269 634:gnd 212:ln_n45 c=3.07262e-16
c1268 634:gnd 169:ln_n44 c=4.72493e-17
c1267 634:gnd 201:ln_n45 c=2.51518e-17
c1266 634:gnd 165:ln_n44 c=2.67954e-18
c1265 634:gnd 1:ln_n124 c=4.83341e-17
c1264 634:gnd 421:ln_n43 c=4.72493e-17
cg1263 633:gnd 0 c=1.84986e-18
c1262 633:gnd 1:ln_n124 c=1.94201e-17
c1261 633:gnd 416:ln_n41 c=1.36771e-18

c1260 633:gnd 212:ln_n45 c=1.36771e-18
c1259 633:gnd 421:ln_n43 c=5.51632e-18
cg1258 631:gnd 0 c=1.40351e-18
c1257 631:gnd 1:ln_n124 c=1.96684e-17
c1256 631:gnd 416:ln_n41 c=1.36771e-18
c1255 631:gnd 212:ln_n45 c=1.36771e-18
cg1254 630:gnd 0 c=1.41195e-18
c1253 630:gnd 1:ln_n124 c=1.9858e-17
c1252 630:gnd 416:ln_n41 c=1.36771e-18
c1251 630:gnd 169:ln_n44 c=5.51632e-18
c1250 630:gnd 212:ln_n45 c=1.36771e-18
cg1249 625:gnd 0 c=9.62302e-18
c1248 625:gnd 226:ln_n46 c=3.57899e-18
c1247 625:gnd 386:load3_cont c=2.47872e-19
c1246 625:gnd 186:ln_n64 c=3.08098e-16
c1245 625:gnd 174:ln_n47 c=4.72493e-17
c1244 625:gnd 399:load3_cont c=2.20932e-19
c1243 625:gnd 398:load3_cont c=2.14382e-18
c1242 625:gnd 175:ln_n64 c=2.3666e-17
c1241 625:gnd 170:ln_n47 c=3.57899e-18
c1240 625:gnd 371:load1_cont c=1.42294e-17
c1239 625:gnd 230:ln_n46 c=4.72493e-17
c1238 625:gnd 1:ln_n124 c=4.92298e-17
cg1237 624:gnd 0 c=1.8571e-18
c1236 624:gnd 1:ln_n124 c=1.94303e-17
c1235 624:gnd 212:ln_n45 c=1.36771e-18
c1234 624:gnd 186:ln_n64 c=1.36771e-18
c1233 624:gnd 230:ln_n46 c=5.51632e-18
cg1232 622:gnd 0 c=1.64799e-18
c1231 622:gnd 1:ln_n124 c=1.96684e-17
c1230 622:gnd 212:ln_n45 c=1.36771e-18
c1229 622:gnd 186:ln_n64 c=1.36771e-18
cg1228 621:gnd 0 c=1.41342e-18
c1227 621:gnd 1:ln_n124 c=1.9865e-17
c1226 621:gnd 212:ln_n45 c=1.36771e-18
c1225 621:gnd 174:ln_n47 c=5.51632e-18
c1224 621:gnd 186:ln_n64 c=1.36771e-18
cg1223 611:gnd 0 c=5.01336e-21
c1222 611:gnd 1:ln_n124 c=2.42952e-19
c1221 611:gnd 299:piezo_inp c=7.67889e-20
c1220 611:gnd 335:vref1 c=4.9058e-19
cg1219 610:gnd 0 c=1.47996e-18
c1218 610:gnd m0|gate c=1.58454e-20

c1217 610:gnd 299:piezo_inp c=4.26916e-19
c1216 610:gnd 335:vref1 c=1.86051e-18
c1215 610:gnd 1:ln_n124 c=1.15436e-17
cg1214 607:gnd 0 c=2.20613e-18
c1213 607:gnd 18:ln_n26 c=9.4454e-18
c1212 607:gnd 34:ln_n27 c=1.54611e-18
c1211 607:gnd 22:ln_n26 c=1.15385e-16
c1210 607:gnd 17:ln_n24 c=1.54611e-18
c1209 607:gnd 1:ln_n124 c=1.92653e-17
cg1208 606:gnd 0 c=1.46446e-17
c1207 606:gnd 18:ln_n26 c=3.74646e-18
c1206 606:gnd 72:ln_n32 c=1.53051e-16
c1205 606:gnd 6:ln_n24 c=2.39497e-17
c1204 606:gnd 68:ln_n32 c=1.03496e-17
c1203 606:gnd 34:ln_n27 c=1.54611e-18
c1202 606:gnd 22:ln_n26 c=4.72176e-17
c1201 606:gnd 17:ln_n24 c=3.12982e-16
c1200 606:gnd 225:ln_n33 c=5.24503e-18
c1199 606:gnd 1:ln_n124 c=5.79246e-17
cg1198 605:gnd 0 c=7.03095e-19
c1197 605:gnd 221:ln_n33 c=9.26576e-18
c1196 605:gnd 72:ln_n32 c=5.24503e-18
c1195 605:gnd 95:ln_n37 c=1.54611e-18
c1194 605:gnd 225:ln_n33 c=1.11551e-16
c1193 605:gnd 1:ln_n124 c=2.02239e-17
c1192 605:gnd 117:ln_n31 c=1.54611e-18
cg1191 604:gnd 0 c=6.64254e-18
c1190 604:gnd 221:ln_n33 c=1.76322e-18
c1189 604:gnd 122:ln_n34 c=1.53468e-16
c1188 604:gnd 109:ln_n31 c=1.76338e-17
c1187 604:gnd 118:ln_n34 c=1.00434e-17
c1186 604:gnd 404:ln_n35 c=5.24503e-18
c1185 604:gnd 95:ln_n37 c=1.54611e-18
c1184 604:gnd 225:ln_n33 c=4.1705e-17
c1183 604:gnd 1:ln_n124 c=4.62459e-17
c1182 604:gnd 117:ln_n31 c=2.19798e-16
cg1181 603:gnd 0 c=5.87854e-19
c1180 603:gnd 400:ln_n35 c=8.28018e-18
c1179 603:gnd 122:ln_n34 c=5.24503e-18
c1178 603:gnd 134:ln_n36 c=1.54611e-18
c1177 603:gnd 404:ln_n35 c=1.11696e-16
c1176 603:gnd 146:ln_n30 c=1.54611e-18
c1175 603:gnd 1:ln_n124 c=2.05863e-17

cg1174 602:gnd 0 c=9.35851e-18
c1173 602:gnd 400:ln_n35 c=1.76322e-18
c1172 602:gnd 135:ln_n30 c=2.5224e-17
c1171 602:gnd 164:ln_n42 c=1.62937e-16
c1170 602:gnd 169:ln_n44 c=6.56615e-19
c1169 602:gnd 134:ln_n36 c=1.54611e-18
c1168 602:gnd 404:ln_n35 c=4.1705e-17
c1167 602:gnd 146:ln_n30 c=3.12982e-16
c1166 602:gnd 160:ln_n42 c=1.06846e-17
c1165 602:gnd 1:ln_n124 c=5.94928e-17
cg1164 601:gnd 0 c=1.40524e-18
c1163 601:gnd 186:ln_n64 c=1.54611e-18
c1162 601:gnd 174:ln_n47 c=1.15325e-16
c1161 601:gnd 398:load3_cont c=8.69874e-20
c1160 601:gnd 212:ln_n45 c=1.54611e-18
c1159 601:gnd 170:ln_n47 c=8.57468e-18
c1158 601:gnd 1:ln_n124 c=2.0307e-17
cg1157 600:gnd 0 c=9.15835e-18
c1156 600:gnd 226:ln_n46 c=1.26531e-17
c1155 600:gnd 186:ln_n64 c=1.54611e-18
c1154 600:gnd 174:ln_n47 c=4.08351e-17
c1153 600:gnd 212:ln_n45 c=3.02823e-16
c1152 600:gnd 170:ln_n47 c=2.65781e-18
c1151 600:gnd m21@2|drn c=1.13538e-19
c1150 600:gnd 201:ln_n45 c=2.58729e-17
c1149 600:gnd 230:ln_n46 c=1.62527e-16
c1148 600:gnd 1:ln_n124 c=6.13056e-17
cg1147 599:gnd 0 c=1.24241e-18
c1146 599:gnd m20|gate c=1.98985e-18
c1145 599:gnd 363:vref3 c=1.04683e-19
c1144 599:gnd 597:vsec_storage_rf c=1.72872e-17
c1143 599:gnd 595:vsec_storage_rf c=3.00428e-18
c1142 599:gnd 360:vref3 c=2.91559e-18
c1141 599:gnd 356:vref3 c=3.72079e-19
c1140 599:gnd 1:ln_n124 c=1.16817e-17
cg1139 c21|minus 0 c=8.35944e-18
c1138 c21|minus 108:ln_n52 c=2.62251e-17
c1137 c21|minus 80:ln_n54 c=2.15144e-16
c1136 c21|minus 96:ln_n52 c=3.51317e-16
c1135 c21|minus 60:ln_n55 c=2.8491e-17
c1134 c21|minus c21|plus c=8.60802e-16
c1133 c21|minus 59:ln_n48 c=1.94325e-19
c1132 c21|minus 79:ln_n56 c=1.57522e-17

c1131 c21|minus 86:ln_n54 c=1.57464e-17
c1130 c21|minus 67:ln_n55 c=3.52672e-18
c1129 c21|minus 47:ln_n48 c=7.08669e-18
c1128 c21|minus 1:ln_n124 c=3.85709e-17
c1127 c21|minus 73:ln_n56 c=2.14092e-16
cg1126 c1|minus 0 c=1.39748e-17
c1125 c1|minus 187:ln_n67 c=2.22203e-16
c1124 c1|minus 147:ln_n60 c=2.92925e-16
c1123 c1|minus 533:cont_n1 c=3.15567e-20
c1122 c1|minus 508:load1 c=4.69012e-18
c1121 c1|minus 518:load1 c=3.09061e-18
c1120 c1|minus 159:ln_n60 c=2.17638e-17
c1119 c1|minus 519:load1 c=5.26643e-18
c1118 c1|minus 517:load1 c=1.08846e-20
c1117 c1|minus 200:ln_n80 c=1.23971e-17
c1116 c1|minus 194:ln_n80 c=1.59854e-16
c1115 c1|minus 193:ln_n67 c=1.52817e-17
c1114 c1|minus 1:ln_n124 c=4.53465e-17
cg1113 c18|minus 0 c=1.18362e-17
c1112 c18|minus 404:ln_n35 c=9.12357e-18
c1111 c18|minus 118:ln_n34 c=8.17937e-18
c1110 c18|minus 160:ln_n42 c=1.17446e-16
c1109 c18|minus 164:ln_n42 c=9.12357e-18
c1108 c18|minus 221:ln_n33 c=5.60226e-18
c1107 c18|minus 23:ln_n27 c=7.69486e-18
c1106 c18|minus 18:ln_n26 c=1.17644e-16
c1105 c18|minus 6:ln_n24 c=7.4193e-18
c1104 c18|minus 68:ln_n32 c=1.10522e-16
c1103 c18|minus 165:ln_n44 c=1.64523e-18
c1102 c18|minus 22:ln_n26 c=9.12357e-18
c1101 c18|minus 72:ln_n32 c=7.52722e-18
c1100 c18|minus 400:ln_n35 c=1.13012e-16
c1099 c18|minus 1:ln_n124 c=4.46375e-17
cg1098 c8|minus 0 c=7.67848e-18
c1097 c8|minus 417:ln_n43 c=1.13953e-16
c1096 c8|minus 169:ln_n44 c=9.12357e-18
c1095 c8|minus 201:ln_n45 c=8.31569e-18
c1094 c8|minus 165:ln_n44 c=1.17258e-16
c1093 c8|minus 160:ln_n42 c=1.64523e-18
c1092 c8|minus 1:ln_n124 c=2.26194e-17
c1091 c8|minus 421:ln_n43 c=9.12357e-18
cg1090 c16|minus 0 c=8.74908e-17
c1089 c16|minus 226:ln_n46 c=1.61598e-16

c1088 c16|minus 417:ln_n43 c=7.46261e-17
c1087 c16|minus 385:load3_cont c=2.81138e-17
c1086 c16|minus 174:ln_n47 c=1.16289e-17
c1085 c16|minus 212:ln_n45 c=3.25455e-17
c1084 c16|minus 175:ln_n64 c=3.72528e-18
c1083 c16|minus 170:ln_n47 c=1.60305e-16
c1082 c16|minus 201:ln_n45 c=3.57481e-16
c1081 c16|minus 365:load1_cont c=1.10009e-19
c1080 c16|minus 371:load1_cont c=9.36499e-19
c1079 c16|minus 390:load3_cont c=1.33865e-17
c1078 c16|minus 230:ln_n46 c=1.16289e-17
c1077 c16|minus 1:ln_n124 c=4.61375e-17
c1076 c16|minus 123:ln_n36 c=4.23198e-19
c1075 c16|minus 421:ln_n43 c=1.96737e-17
c1074 c16|minus 388:load3_cont c=7.79992e-17
cg1073 c17|minus 0 c=1.54529e-17
c1072 c17|minus 1:ln_n124 c=4.31985e-18
c1071 c17|minus 17:ln_n24 c=2.40589e-17
c1070 c17|minus 22:ln_n26 c=3.63448e-18
c1069 c17|minus 72:ln_n32 c=2.79468e-18
c1068 c17|minus 68:ln_n32 c=3.5701e-17
c1067 c17|minus 6:ln_n24 c=3.22074e-16
c1066 c17|minus 18:ln_n26 c=5.08561e-17
cg1065 598:vsec_storage_rf 0 c=5.46785e-18
c1064 598:vsec_storage_rf 59:ln_n48 c=2.71107e-18
c1063 598:vsec_storage_rf 569:rf_inn c=3.79826e-18
c1062 598:vsec_storage_rf m25|drn c=1.81569e-19
c1061 598:vsec_storage_rf 567:rf_inn c=9.25666e-18
c1060 598:vsec_storage_rf 281:rf_inp c=3.02963e-19
c1059 598:vsec_storage_rf m26|gate c=6.25167e-18
c1058 598:vsec_storage_rf 274:rf_inp c=1.84269e-18
c1057 598:vsec_storage_rf 278:rf_inp c=1.6188e-20
c1056 598:vsec_storage_rf 571:rf_inn c=1.32223e-17
c1055 598:vsec_storage_rf 277:rf_inp c=5.21261e-17
c1054 598:vsec_storage_rf 565:rf_inn c=6.24335e-19
c1053 598:vsec_storage_rf 1:ln_n124 c=1.37945e-17
cg1052 597:vsec_storage_rf 0 c=6.4005e-18
c1051 597:vsec_storage_rf 1:ln_n124 c=1.24602e-17
cg1050 596:vsec_storage_rf 0 c=6.30663e-18
c1049 596:vsec_storage_rf 1:ln_n124 c=1.3526e-17
c1048 596:vsec_storage_rf 277:rf_inp c=1.14465e-18
c1047 596:vsec_storage_rf 565:rf_inn c=3.98868e-18
c1046 596:vsec_storage_rf 548:rf_inn c=4.91813e-18

c1045 596:vsec_storage_rf 570:rf_inn c=3.45105e-18
c1044 596:vsec_storage_rf m25|drn c=5.21699e-20
c1043 596:vsec_storage_rf 572:rf_inn c=5.36409e-19
c1042 596:vsec_storage_rf 569:rf_inn c=1.06917e-19
cg1041 595:vsec_storage_rf 0 c=5.43734e-18
c1040 595:vsec_storage_rf m27|gate c=7.08479e-19
c1039 595:vsec_storage_rf 573:rf_inn c=3.82811e-18
c1038 595:vsec_storage_rf 547:rf_inn c=7.71794e-18
c1037 595:vsec_storage_rf 280:rf_inp c=2.92488e-18
c1036 595:vsec_storage_rf 276:rf_inp c=1.9778e-18
c1035 595:vsec_storage_rf 275:rf_inp c=2.98959e-18
c1034 595:vsec_storage_rf 549:rf_inn c=1.94262e-17
c1033 595:vsec_storage_rf 261:rf_inp c=1.07808e-18
c1032 595:vsec_storage_rf 1:ln_n124 c=2.5604e-17
cg1031 594:vsec_storage_rf 0 c=5.52499e-18
c1030 594:vsec_storage_rf 1:ln_n124 c=1.40222e-17
c1029 594:vsec_storage_rf 567:rf_inn c=2.9874e-20
cg1028 583:vsec_storage_rf 0 c=2.47362e-18
c1027 583:vsec_storage_rf 551:rf_inn c=1.12806e-18
c1026 583:vsec_storage_rf m25|gate c=1.49221e-18
c1025 583:vsec_storage_rf m25|drn c=9.9873e-18
c1024 583:vsec_storage_rf 570:rf_inn c=3.46839e-19
c1023 583:vsec_storage_rf 79:ln_n56 c=1.25453e-19
c1022 583:vsec_storage_rf 1:ln_n124 c=2.3179e-16
cg1021 580:vsec_storage_rf 0 c=1.53101e-17
c1020 580:vsec_storage_rf 568:rf_inn c=3.08118e-18
c1019 580:vsec_storage_rf 551:rf_inn c=1.94969e-17
c1018 580:vsec_storage_rf 553:rf_inn c=8.18561e-19
c1017 580:vsec_storage_rf 276:rf_inp c=3.69201e-18
c1016 580:vsec_storage_rf m25|gate c=2.49465e-18
c1015 580:vsec_storage_rf m25|drn c=1.68419e-18
c1014 580:vsec_storage_rf 567:rf_inn c=2.06928e-17
c1013 580:vsec_storage_rf 570:rf_inn c=4.7936e-17
c1012 580:vsec_storage_rf m26|gate c=8.19335e-19
c1011 580:vsec_storage_rf 79:ln_n56 c=1.07716e-17
c1010 580:vsec_storage_rf 549:rf_inn c=8.62262e-18
c1009 580:vsec_storage_rf 574:rf_inn c=6.07163e-19
c1008 580:vsec_storage_rf 571:rf_inn c=1.45935e-17
c1007 580:vsec_storage_rf 261:rf_inp c=2.35846e-18
c1006 580:vsec_storage_rf rf_inn c=6.88553e-19
c1005 580:vsec_storage_rf 548:rf_inn c=3.23719e-17
c1004 580:vsec_storage_rf 565:rf_inn c=2.27444e-17
c1003 580:vsec_storage_rf 1:ln_n124 c=7.84104e-17

c1002 580:vsec_storage_rf 73:ln_n56 c=5.39797e-19
cg1001 579:vsec_storage_rf 0 c=2.75691e-18
c1000 579:vsec_storage_rf 568:rf_inn c=2.16087e-17
c999 579:vsec_storage_rf m27|gate c=4.58895e-19
c998 579:vsec_storage_rf 547:rf_inn c=1.29496e-17
c997 579:vsec_storage_rf 551:rf_inn c=2.98284e-17
c996 579:vsec_storage_rf 553:rf_inn c=1.09297e-18
c995 579:vsec_storage_rf 276:rf_inp c=2.12039e-17
c994 579:vsec_storage_rf m25|gate c=6.31827e-18
c993 579:vsec_storage_rf 549:rf_inn c=2.01655e-17
c992 579:vsec_storage_rf 574:rf_inn c=9.78925e-19
c991 579:vsec_storage_rf 1:ln_n124 c=1.82063e-17
cg990 578:vsec_storage_rf 0 c=2.07253e-17
c989 578:vsec_storage_rf 567:rf_inn c=1.97869e-17
c988 578:vsec_storage_rf m26|gate c=2.69029e-20
c987 578:vsec_storage_rf rf_inn c=1.15303e-18
c986 578:vsec_storage_rf 565:rf_inn c=1.13575e-18
c985 578:vsec_storage_rf 1:ln_n124 c=3.46146e-17
cg984 577:vsec_storage_rf 0 c=4.7604e-18
c983 577:vsec_storage_rf 551:rf_inn c=2.66395e-17
c982 577:vsec_storage_rf 553:rf_inn c=1.30894e-18
c981 577:vsec_storage_rf m25|drn c=2.00311e-18
c980 577:vsec_storage_rf 567:rf_inn c=1.07559e-18
c979 577:vsec_storage_rf 570:rf_inn c=1.95127e-17
c978 577:vsec_storage_rf 79:ln_n56 c=1.27126e-18
c977 577:vsec_storage_rf 549:rf_inn c=4.83681e-18
c976 577:vsec_storage_rf 565:rf_inn c=2.97555e-18
c975 577:vsec_storage_rf 1:ln_n124 c=2.54409e-17
c974 577:vsec_storage_rf 73:ln_n56 c=3.85702e-19
cg973 575:vsec_storage_rf 0 c=7.27547e-18
c972 575:vsec_storage_rf 59:ln_n48 c=5.42727e-19
c971 575:vsec_storage_rf 567:rf_inn c=3.49555e-18
c970 575:vsec_storage_rf 79:ln_n56 c=5.07155e-19
c969 575:vsec_storage_rf 47:ln_n48 c=8.20749e-18
c968 575:vsec_storage_rf 1:ln_n124 c=9.72848e-18
c967 575:vsec_storage_rf 73:ln_n56 c=8.44667e-18
c966 m25|src m27|gate c=2.82717e-19
c965 m25|src m27|drn c=7.67449e-19
c964 m25|src 276:rf_inp c=1.61466e-18
c963 m25|src m25|gate c=1.1947e-17
c962 m25|src m26|gate c=2.14288e-20
c961 m25|src m26|drn c=5.36307e-19
c960 m25|src 574:rf_inn c=4.32125e-19

c959 m25|src 1:ln_n124 c=2.345e-16
c958 m20|src 1:ln_n124 c=9.98056e-17
c957 m20|src m27|drn c=5.96365e-19
c956 m20|src m27|gate c=2.1052e-19
c955 m26|src m28|drn c=7.9368e-23
c954 m26|src m25|gate c=4.26059e-20
c953 m26|src 569:rf_inn c=5.55736e-19
c952 m26|src m25|drn c=4.95106e-19
c951 m26|src 572:rf_inn c=1.12252e-19
c950 m26|src m26|gate c=1.19305e-17
c949 m26|src 571:rf_inn c=5.45659e-19
c948 m26|src 548:rf_inn c=1.12659e-19
c947 m26|src 1:ln_n124 c=2.36305e-16
cg946 m20|gate 0 c=5.47555e-18
c945 m20|gate m27|gate c=4.20164e-19
c944 m20|gate m27|drn c=1.86176e-19
c943 m20|gate 573:rf_inn c=2.41978e-19
c942 m20|gate m28|drn c=4.54179e-20
c941 m20|gate 547:rf_inn c=4.93877e-19
c940 m20|gate 280:rf_inp c=7.7112e-20
c939 m20|gate 1:ln_n124 c=6.50032e-17
cg938 c21|plus 0 c=4.88193e-17
c937 c21|plus 80:ln_n54 c=6.9248e-17
c936 c21|plus 59:ln_n48 c=2.62233e-17
c935 c21|plus 79:ln_n56 c=3.78318e-18
c934 c21|plus 86:ln_n54 c=3.34228e-18
c933 c21|plus 47:ln_n48 c=3.55899e-16
c932 c21|plus 1:ln_n124 c=2.80759e-17
c931 c21|plus 73:ln_n56 c=6.15987e-17
cg930 574:rf_inn 0 c=7.71158e-18
c929 574:rf_inn m27|gate c=1.38616e-18
c928 574:rf_inn m28|drn c=1.18903e-18
c927 574:rf_inn 280:rf_inp c=1.72144e-17
c926 574:rf_inn 276:rf_inp c=1.03252e-17
c925 574:rf_inn 275:rf_inp c=4.57641e-18
c924 574:rf_inn m25|gate c=2.16927e-19
c923 574:rf_inn 279:rf_inp c=8.58356e-19
c922 574:rf_inn 281:rf_inp c=2.70404e-17
c921 574:rf_inn 269:rf_inp c=5.73015e-18
c920 574:rf_inn 274:rf_inp c=4.86486e-18
c919 574:rf_inn m26|drn c=1.62331e-18
c918 574:rf_inn 278:rf_inp c=6.56178e-18
c917 574:rf_inn 261:rf_inp c=2.60651e-17

c916 574:rf_inn 273:rf_inp c=1.1264e-20
c915 574:rf_inn 277:rf_inp c=2.19722e-17
c914 574:rf_inn 1:ln_n124 c=3.45536e-17
cg913 573:rf_inn 0 c=1.80273e-18
c912 573:rf_inn m27|gate c=1.74939e-18
c911 573:rf_inn 280:rf_inp c=3.86861e-18
c910 573:rf_inn 1:ln_n124 c=4.13705e-18
cg909 572:rf_inn 0 c=9.26544e-20
c908 572:rf_inn 1:ln_n124 c=5.12133e-19
c907 572:rf_inn 261:rf_inp c=2.62664e-20
cg906 571:rf_inn 0 c=2.62816e-18
c905 571:rf_inn 276:rf_inp c=1.39831e-17
c904 571:rf_inn 279:rf_inp c=3.48846e-19
c903 571:rf_inn m25|gate c=1.24197e-18
c902 571:rf_inn 261:rf_inp c=9.27695e-18
c901 571:rf_inn 277:rf_inp c=2.43362e-20
c900 571:rf_inn 1:ln_n124 c=1.61859e-17
cg899 570:rf_inn 0 c=1.48714e-18
c898 570:rf_inn 276:rf_inp c=2.2663e-17
c897 570:rf_inn 275:rf_inp c=1.3734e-18
c896 570:rf_inn m25|gate c=1.89691e-18
c895 570:rf_inn 261:rf_inp c=1.92892e-18
c894 570:rf_inn 1:ln_n124 c=1.11844e-17
cg893 569:rf_inn 0 c=6.47005e-19
c892 569:rf_inn 1:ln_n124 c=3.42025e-18
c891 569:rf_inn 276:rf_inp c=3.11694e-18
cg890 568:rf_inn 0 c=7.81869e-19
c889 568:rf_inn 276:rf_inp c=4.24704e-18
c888 568:rf_inn m25|gate c=1.98075e-18
c887 568:rf_inn 1:ln_n124 c=2.38027e-18
cg886 567:rf_inn 0 c=7.3261e-18
c885 567:rf_inn 279:rf_inp c=1.43653e-18
c884 567:rf_inn 261:rf_inp c=1.59382e-17
c883 567:rf_inn 277:rf_inp c=4.33543e-18
c882 567:rf_inn 1:ln_n124 c=1.82406e-17
cg881 565:rf_inn 0 c=1.02631e-17
c880 565:rf_inn 261:rf_inp c=1.17806e-17
c879 565:rf_inn 1:ln_n124 c=9.82187e-18
cg878 rf_inn 0 c=1.84243e-17
c877 rf_inn 1:ln_n124 c=2.43045e-17
c876 rf_inn 279:rf_inp c=1.48383e-17
c875 rf_inn 277:rf_inp c=5.83996e-18
cg874 553:rf_inn 0 c=3.26125e-19

c873 553:rf_inn 1:ln_n124 c=1.15653e-18
cg872 551:rf_inn 0 c=2.72407e-18
c871 551:rf_inn 276:rf_inp c=1.1349e-17
c870 551:rf_inn m25|gate c=3.81993e-18
c869 551:rf_inn 1:ln_n124 c=1.03743e-17
cg868 549:rf_inn 0 c=2.65557e-17
c867 549:rf_inn 280:rf_inp c=7.51853e-18
c866 549:rf_inn 276:rf_inp c=8.05679e-18
c865 549:rf_inn 275:rf_inp c=2.04829e-18
c864 549:rf_inn 279:rf_inp c=2.36336e-18
c863 549:rf_inn 261:rf_inp c=2.28051e-17
c862 549:rf_inn 1:ln_n124 c=3.79869e-17
cg861 m25|drn 0 c=8.17704e-19
c860 m25|drn 276:rf_inp c=5.10147e-19
c859 m25|drn m25|gate c=1.19305e-17
c858 m25|drn 1:ln_n124 c=2.27377e-16
cg857 m27|drn 0 c=1.02636e-19
c856 m27|drn m27|gate c=1.16899e-17
c855 m27|drn m28|drn c=5.45527e-19
c854 m27|drn 280:rf_inp c=6.55589e-19
c853 m27|drn 1:ln_n124 c=1.02733e-16
cg852 m26|gate 0 c=2.92039e-18
c851 m26|gate 276:rf_inp c=5.2382e-19
c850 m26|gate 279:rf_inp c=3.25785e-19
c849 m26|gate m25|gate c=1.11523e-18
c848 m26|gate 281:rf_inp c=5.59548e-19
c847 m26|gate m26|drn c=1.1947e-17
c846 m26|gate 277:rf_inp c=6.67645e-18
c845 m26|gate 1:ln_n124 c=6.87634e-17
cg844 m28|gate 0 c=3.03421e-18
c843 m28|gate m27|gate c=1.1865e-18
c842 m28|gate m28|drn c=1.16916e-17
c841 m28|gate 280:rf_inp c=1.39186e-18
c840 m28|gate 281:rf_inp c=3.52396e-18
c839 m28|gate m26|drn c=2.91163e-19
c838 m28|gate 1:ln_n124 c=6.28348e-17
cg837 548:rf_inn 0 c=1.41198e-18
c836 548:rf_inn 261:rf_inp c=2.65047e-20
c835 548:rf_inn 1:ln_n124 c=6.43467e-18
cg834 547:rf_inn 0 c=2.19354e-18
c833 547:rf_inn m27|gate c=1.4603e-18
c832 547:rf_inn 280:rf_inp c=2.36789e-17
c831 547:rf_inn m25|gate c=8.91455e-19

c830 547:rf_inn 1:ln_n124 c=1.49509e-17
cg829 546:ln_n127 0 c=2.05601e-18
c828 546:ln_n127 1:ln_n124 c=8.18286e-19
c827 546:ln_n127 388:load3_cont c=2.32722e-18
c826 546:ln_n127 233:ln_n126 c=1.0658e-17
c825 546:ln_n127 540:ln_n125 c=3.08929e-18
c824 546:ln_n127 385:load3_cont c=5.46464e-17
cg823 545:ln_n127 0 c=1.35871e-17
c822 545:ln_n127 391:load3_cont c=5.46235e-18
c821 545:ln_n127 540:ln_n125 c=1.04593e-18
c820 545:ln_n127 385:load3_cont c=3.35278e-17
c819 545:ln_n127 233:ln_n126 c=2.12313e-17
c818 545:ln_n127 542:ln_n125 c=2.64143e-18
c817 545:ln_n127 543:ln_n125 c=4.80801e-18
c816 545:ln_n127 541:ln_n125 c=1.26778e-18
c815 545:ln_n127 1:ln_n124 c=1.84839e-17
cg814 544:ln_n127 0 c=7.98601e-17
c813 544:ln_n127 1:ln_n124 c=9.42625e-17
c812 544:ln_n127 541:ln_n125 c=4.58026e-19
c811 544:ln_n127 233:ln_n126 c=9.64499e-17
c810 544:ln_n127 388:load3_cont c=6.5489e-17
c809 544:ln_n127 385:load3_cont c=7.51759e-17
c808 544:ln_n127 540:ln_n125 c=8.19118e-19
cg807 543:ln_n125 0 c=3.43509e-17
c806 543:ln_n125 4:ln_n128 c=5.97499e-17
c805 543:ln_n125 1:ln_n124 c=5.18675e-17
cg804 542:ln_n125 0 c=5.23935e-18
c803 542:ln_n125 1:ln_n124 c=1.05995e-17
c802 541:ln_n125 1:ln_n124 c=7.19469e-18
c801 541:ln_n125 4:ln_n128 c=1.06449e-18
cg800 540:ln_n125 0 c=1.61657e-17
c799 540:ln_n125 1:ln_n124 c=1.58653e-17
c798 540:ln_n125 4:ln_n128 c=7.40722e-18
cg797 539:cont_n1 0 c=9.43474e-18
c796 539:cont_n1 m12|gate c=1.14292e-18
c795 539:cont_n1 m14|gate c=5.91178e-18
c794 539:cont_n1 523:cont_n2 c=3.12094e-17
c793 539:cont_n1 1:ln_n124 c=1.67043e-17
cg792 533:cont_n1 0 c=4.98056e-18
c791 533:cont_n1 m12|gate c=3.53353e-19
c790 533:cont_n1 m12|drn c=2.91992e-19
c789 533:cont_n1 523:cont_n2 c=3.45876e-18
c788 533:cont_n1 1:ln_n124 c=2.38752e-17

cg787 m13|gate 0 c=2.17477e-18
c786 m13|gate 1:ln_n124 c=6.52521e-17
cg785 m13|drn 0 c=6.55219e-19
c784 m13|drn 1:ln_n124 c=1.01408e-16
cg783 m14|src 0 c=1.10524e-18
c782 m14|src m12|gate c=1.01936e-18
c781 m14|src m12|drn c=3.46509e-22
c780 m14|src m14|gate c=1.19483e-17
c779 m14|src 523:cont_n2 c=8.22385e-20
c778 m14|src 1:ln_n124 c=2.28202e-16
cg777 m6|gate 0 c=3.00014e-18
c776 m6|gate 523:cont_n2 c=3.7016e-19
c775 m6|gate 1:ln_n124 c=6.53856e-17
cg774 531:cont_n2 0 c=2.38154e-17
c773 531:cont_n2 1:ln_n124 c=2.91093e-17
cg772 530:cont_n2 0 c=5.38266e-18
c771 530:cont_n2 1:ln_n124 c=7.80455e-18
cg770 523:cont_n2 0 c=9.0008e-18
c769 523:cont_n2 1:ln_n124 c=4.25557e-17
cg768 521:cont_n2 0 c=4.65897e-18
c767 521:cont_n2 1:ln_n124 c=2.25358e-17
cg766 m10|drn 0 c=1.23306e-17
c765 m10|drn 1:ln_n124 c=1.01946e-16
cg764 m12|drn 0 c=2.85023e-19
c763 m12|drn 1:ln_n124 c=1.02685e-16
cg762 m24|gate 0 c=4.76214e-18
c761 m24|gate 1:ln_n124 c=6.55499e-17
cg760 m12|gate 0 c=1.42053e-18
c759 m12|gate 1:ln_n124 c=6.31272e-17
cg758 m14|gate 0 c=4.2171e-18
c757 m14|gate 1:ln_n124 c=6.7696e-17
cg756 520:load1 0 c=2.59884e-17
c755 520:load1 1:ln_n124 c=1.44477e-17
c754 520:load1 504:load2 c=2.03738e-17
c753 520:load1 501:load2 c=4.50874e-18
cg752 519:load1 0 c=1.61152e-18
c751 519:load1 m22|drn c=1.04975e-19
c750 519:load1 502:load2 c=1.63125e-17
c749 519:load1 493:load2 c=1.84791e-17
c748 519:load1 504:load2 c=1.31403e-19
c747 519:load1 501:load2 c=3.85022e-20
c746 519:load1 193:ln_n67 c=1.36728e-18
c745 519:load1 1:ln_n124 c=9.46854e-18

cg744 518:load1 0 c=1.67576e-17
c743 518:load1 187:ln_n67 c=2.83888e-17
c742 518:load1 m22|drn c=1.55945e-18
c741 518:load1 502:load2 c=1.1394e-18
c740 518:load1 492:load2 c=5.21788e-18
c739 518:load1 493:load2 c=2.27237e-16
c738 518:load1 504:load2 c=5.37487e-18
c737 518:load1 501:load2 c=5.53917e-18
c736 518:load1 193:ln_n67 c=1.34062e-16
c735 518:load1 1:ln_n124 c=2.16486e-17
cg734 517:load1 0 c=1.35528e-17
c733 517:load1 493:load2 c=2.4177e-18
c732 517:load1 504:load2 c=3.95424e-18
c731 517:load1 501:load2 c=1.2024e-17
c730 517:load1 1:ln_n124 c=1.1949e-17
cg729 510:load1 0 c=6.42853e-18
c728 510:load1 1:ln_n124 c=1.16656e-17
cg727 509:load1 0 c=1.65279e-17
c726 509:load1 m22@2|drn c=7.36973e-19
c725 509:load1 m22|drn c=2.74703e-19
c724 509:load1 m21@2|gate c=4.29376e-18
c723 509:load1 492:load2 c=6.04551e-17
c722 509:load1 m21|gate c=4.58468e-18
c721 509:load1 503:load2 c=2.28187e-17
c720 509:load1 370:load1_cont c=2.85006e-17
c719 509:load1 371:load1_cont c=1.18353e-16
c718 509:load1 1:ln_n124 c=1.89948e-17
cg717 508:load1 0 c=9.9912e-19
c716 508:load1 187:ln_n67 c=9.51032e-19
c715 508:load1 m22|drn c=2.45491e-19
c714 508:load1 m21@2|gate c=5.81575e-19
c713 508:load1 492:load2 c=1.38898e-17
c712 508:load1 m21|gate c=5.68994e-18
c711 508:load1 370:load1_cont c=7.32849e-18
c710 508:load1 371:load1_cont c=1.04921e-19
c709 508:load1 193:ln_n67 c=2.29008e-18
c708 508:load1 1:ln_n124 c=1.1551e-17
cg707 506:load1 0 c=8.32105e-18
c706 506:load1 493:load2 c=8.54081e-19
c705 506:load1 504:load2 c=4.28113e-18
c704 506:load1 501:load2 c=9.79554e-18
c703 506:load1 1:ln_n124 c=2.09422e-17
cg702 m21@2|drn 0 c=1.83067e-18

c701 m21@2|drn m22@2|drn c=4.99931e-19
c700 m21@2|drn m21@2|gate c=4.79591e-18
c699 m21@2|drn 492:load2 c=1.93369e-19
c698 m21@2|drn m21|gate c=4.23476e-18
c697 m21@2|drn 503:load2 c=2.89287e-19
c696 m21@2|drn 370:load1_cont c=2.13011e-18
c695 m21@2|drn 371:load1_cont c=1.11603e-19
c694 m21@2|drn 1:ln_n124 c=2.28406e-16
cg693 m21|drn 0 c=1.16089e-18
c692 m21|drn m22|drn c=5.0332e-19
c691 m21|drn m21@2|gate c=4.79591e-18
c690 m21|drn 502:load2 c=9.32791e-20
c689 m21|drn 492:load2 c=3.39649e-19
c688 m21|drn m21|gate c=4.23476e-18
c687 m21|drn 370:load1_cont c=5.05836e-19
c686 m21|drn 1:ln_n124 c=2.28825e-16
cg685 504:load2 0 c=3.55278e-17
c684 504:load2 373:load2_cont c=1.97448e-19
c683 504:load2 379:load2_cont c=2.48597e-17
c682 504:load2 380:load2_cont c=1.15329e-18
c681 504:load2 1:ln_n124 c=9.40374e-18
cg680 503:load2 0 c=1.69432e-17
c679 503:load2 378:load2_cont c=7.38264e-18
c678 503:load2 m22@2|gate c=6.13469e-18
c677 503:load2 m22|gate c=9.89434e-20
c676 503:load2 371:load1_cont c=1.14018e-16
c675 503:load2 1:ln_n124 c=1.17489e-17
cg674 502:load2 0 c=1.80435e-18
c673 502:load2 373:load2_cont c=1.49837e-19
c672 502:load2 379:load2_cont c=4.66464e-19
c671 502:load2 380:load2_cont c=1.37594e-20
c670 502:load2 1:ln_n124 c=5.59724e-18
cg669 501:load2 0 c=8.75877e-18
c668 501:load2 373:load2_cont c=3.96561e-20
c667 501:load2 1:ln_n124 c=8.51004e-18
cg666 493:load2 0 c=4.94415e-17
c665 493:load2 373:load2_cont c=6.32299e-17
c664 493:load2 378:load2_cont c=1.82164e-18
c663 493:load2 m22|gate c=2.67845e-18
c662 493:load2 379:load2_cont c=7.58112e-18
c661 493:load2 380:load2_cont c=2.49215e-17
c660 493:load2 371:load1_cont c=9.85156e-19
c659 493:load2 1:ln_n124 c=3.42288e-17

cg658 492:load2 0 c=6.99369e-18
c657 492:load2 373:load2_cont c=1.36893e-17
c656 492:load2 378:load2_cont c=2.75054e-18
c655 492:load2 m22@2|gate c=1.60531e-18
c654 492:load2 m22|gate c=7.4992e-18
c653 492:load2 379:load2_cont c=1.15302e-18
c652 492:load2 m21|gate c=5.36973e-19
c651 492:load2 380:load2_cont c=7.07686e-19
c650 492:load2 370:load1_cont c=9.11072e-20
c649 492:load2 371:load1_cont c=1.27904e-18
c648 492:load2 1:ln_n124 c=1.36927e-17
cg647 m22@2|drn 0 c=1.68125e-18
c646 m22@2|drn 1:ln_n124 c=2.28452e-16
c645 m22@2|drn 371:load1_cont c=1.55323e-19
c644 m22@2|drn 378:load2_cont c=5.06135e-19
c643 m22@2|drn m21|gate c=9.34611e-21
c642 m22@2|drn m22@2|gate c=9.03067e-18
c641 m22|drn 373:load2_cont c=1.58417e-18
c640 m22|drn m22|gate c=9.03067e-18
c639 m22|drn m21|gate c=9.34611e-21
c638 m22|drn 380:load2_cont c=2.35533e-19
c637 m22|drn 1:ln_n124 c=2.28569e-16
cg636 490:vreg 0 c=2.71471e-17
c635 490:vreg 60:ln_n55 c=4.48222e-18
c634 490:vreg 46:ln_n25 c=5.67289e-17
c633 490:vreg 67:ln_n55 c=5.32433e-17
c632 490:vreg 35:ln_n25 c=3.70223e-18
c631 490:vreg 1:ln_n124 c=9.27195e-18
cg630 489:vreg 0 c=1.38948e-18
c629 489:vreg 1:ln_n124 c=1.10276e-17
c628 489:vreg 46:ln_n25 c=2.66999e-21
c627 489:vreg 220:ln_n38 c=5.32433e-17
c626 489:vreg 213:ln_n38 c=3.19129e-18
cg625 488:vreg 0 c=3.51603e-17
c624 488:vreg 1:ln_n124 c=7.75283e-18
cg623 487:vreg 0 c=1.83961e-17
c622 487:vreg 1:ln_n124 c=1.74419e-17
cg621 486:vreg 0 c=1.39456e-17
c620 486:vreg 1:ln_n124 c=1.35886e-17
cg619 467:vreg 0 c=3.44404e-17
c618 467:vreg 46:ln_n25 c=2.76891e-20
c617 467:vreg 134:ln_n36 c=5.01002e-18
c616 467:vreg 95:ln_n37 c=7.69697e-18

c615 467:vreg 1:ln_n124 c=1.79718e-15
cg614 466:vreg 0 c=3.48282e-17
c613 466:vreg 213:ln_n38 c=5.88475e-17
c612 466:vreg 46:ln_n25 c=1.13156e-17
c611 466:vreg 34:ln_n27 c=9.54804e-18
c610 466:vreg 220:ln_n38 c=3.04463e-18
c609 466:vreg 35:ln_n25 c=9.12418e-17
c608 466:vreg 1:ln_n124 c=4.16007e-17
c607 466:vreg 23:ln_n27 c=7.6136e-17
cg606 443:vreg 0 c=7.02094e-19
c605 443:vreg 1:ln_n124 c=8.18468e-18
c604 443:vreg 46:ln_n25 c=3.11657e-17
c603 443:vreg 35:ln_n25 c=1.6501e-18
cg602 442:vreg 0 c=3.35345e-18
c601 442:vreg 213:ln_n38 c=1.58208e-17
c600 442:vreg 46:ln_n25 c=2.98951e-17
c599 442:vreg 220:ln_n38 c=1.95065e-16
c598 442:vreg 35:ln_n25 c=1.69509e-18
c597 442:vreg 1:ln_n124 c=4.4829e-17
cg596 440:vreg 0 c=2.84913e-18
c595 440:vreg 1:ln_n124 c=4.48907e-17
c594 440:vreg 46:ln_n25 c=2.98951e-17
c593 440:vreg 220:ln_n38 c=9.49531e-18
c592 440:vreg 35:ln_n25 c=1.75889e-18
cg591 439:vreg 0 c=3.51047e-19
c590 439:vreg 1:ln_n124 c=4.09234e-18
c589 439:vreg 46:ln_n25 c=1.55828e-17
c588 439:vreg 35:ln_n25 c=8.25049e-19
cg587 438:vreg 0 c=3.15895e-18
c586 438:vreg 1:ln_n124 c=4.48675e-17
c585 438:vreg 46:ln_n25 c=2.9839e-17
c584 438:vreg 35:ln_n25 c=1.69509e-18
cg583 435:vreg 0 c=2.69564e-18
c582 435:vreg 1:ln_n124 c=4.49984e-17
c581 435:vreg 46:ln_n25 c=2.98951e-17
c580 435:vreg 67:ln_n55 c=9.49531e-18
c579 435:vreg 35:ln_n25 c=1.75889e-18
cg578 434:vreg 0 c=8.2023e-17
c577 434:vreg 1:ln_n124 c=7.24838e-17
cg576 433:vreg 0 c=8.6552e-17
c575 433:vreg 1:ln_n124 c=6.88974e-17
cg574 432:vreg 0 c=4.14214e-18
c573 432:vreg 213:ln_n38 c=2.91361e-17

c572 432:vreg 46:ln_n25 c=2.98907e-19
c571 432:vreg 34:ln_n27 c=1.19378e-17
c570 432:vreg 134:ln_n36 c=2.82241e-18
c569 432:vreg 220:ln_n38 c=8.37551e-17
c568 432:vreg 95:ln_n37 c=7.3243e-18
c567 432:vreg 1:ln_n124 c=3.00268e-17
c566 432:vreg 23:ln_n27 c=4.07263e-19
cg565 431:vreg 0 c=1.67296e-18
c564 431:vreg 213:ln_n38 c=4.24036e-18
c563 431:vreg 46:ln_n25 c=5.67289e-17
c562 431:vreg 220:ln_n38 c=5.31977e-17
c561 431:vreg 35:ln_n25 c=3.70223e-18
c560 431:vreg 1:ln_n124 c=9.60292e-18
cg559 430:vreg 0 c=3.51047e-19
c558 430:vreg 1:ln_n124 c=4.09234e-18
c557 430:vreg 46:ln_n25 c=1.55828e-17
c556 430:vreg 35:ln_n25 c=8.25049e-19
cg555 429:vreg 0 c=3.8157e-17
c554 429:vreg 60:ln_n55 c=1.89178e-17
c553 429:vreg 46:ln_n25 c=2.98764e-17
c552 429:vreg 67:ln_n55 c=2.48036e-16
c551 429:vreg 35:ln_n25 c=1.69509e-18
c550 429:vreg 1:ln_n124 c=5.0567e-17
cg549 m9|drn 0 c=1.55611e-18
c548 m9|drn 1:ln_n124 c=2.30181e-16
cg547 428:vreg 0 c=2.81129e-17
c546 428:vreg 34:ln_n27 c=1.63773e-16
c545 428:vreg 95:ln_n37 c=8.62023e-17
c544 428:vreg 1:ln_n124 c=4.68349e-17
c543 428:vreg 87:ln_n37 c=2.07737e-17
c542 428:vreg 23:ln_n27 c=3.63215e-17
cg541 423:vreg 0 c=1.64992e-17
c540 423:vreg 46:ln_n25 c=2.07275e-18
c539 423:vreg 34:ln_n27 c=3.90653e-19
c538 423:vreg 134:ln_n36 c=4.51794e-17
c537 423:vreg 220:ln_n38 c=6.35957e-18
c536 423:vreg 95:ln_n37 c=4.29976e-17
c535 423:vreg 35:ln_n25 c=4.12405e-19
c534 423:vreg 1:ln_n124 c=2.59313e-17
c533 423:vreg 87:ln_n37 c=9.87086e-18
c532 423:vreg 123:ln_n36 c=1.22437e-17
cg531 c15|minus 0 c=1.04141e-16
c530 c15|minus 60:ln_n55 c=2.57593e-16

c529 c15|minus 213:ln_n38 c=2.03353e-16
c528 c15|minus 46:ln_n25 c=2.08248e-17
c527 c15|minus 67:ln_n55 c=1.89143e-17
c526 c15|minus 220:ln_n38 c=1.60508e-17
c525 c15|minus 35:ln_n25 c=2.79717e-16
c524 c15|minus 1:ln_n124 c=3.48379e-17
c523 c15|minus 23:ln_n27 c=3.11658e-18
cg522 421:ln_n43 0 c=1.97535e-17
c521 421:ln_n43 1:ln_n124 c=2.53755e-17
cg520 417:ln_n43 0 c=1.01484e-17
c519 417:ln_n43 1:ln_n124 c=7.66548e-18
cg518 416:ln_n41 0 c=1.06098e-16
c517 416:ln_n41 1:ln_n124 c=4.86925e-17
cg516 405:ln_n41 0 c=1.03223e-16
c515 405:ln_n41 1:ln_n124 c=1.71993e-17
cg514 404:ln_n35 0 c=5.54419e-18
c513 404:ln_n35 122:ln_n34 c=1.06149e-16
c512 404:ln_n35 118:ln_n34 c=8.89359e-18
c511 404:ln_n35 1:ln_n124 c=1.81989e-17
cg510 400:ln_n35 0 c=2.55801e-18
c509 400:ln_n35 122:ln_n34 c=8.89359e-18
c508 400:ln_n35 118:ln_n34 c=1.09244e-16
c507 400:ln_n35 1:ln_n124 c=8.5123e-18
cg506 399:load3_cont 0 c=1.31964e-17
c505 399:load3_cont 1:ln_n124 c=2.01428e-17
cg504 398:load3_cont 0 c=4.02632e-18
c503 398:load3_cont 1:ln_n124 c=1.19274e-17
cg502 391:load3_cont 0 c=2.09513e-18
c501 391:load3_cont 233:ln_n126 c=3.99391e-18
c500 391:load3_cont 1:ln_n124 c=2.36955e-18
cg499 390:load3_cont 0 c=1.61633e-17
c498 390:load3_cont 1:ln_n124 c=9.94371e-18
c497 390:load3_cont 230:ln_n46 c=9.02551e-18
c496 390:load3_cont 226:ln_n46 c=1.78167e-17
cg495 388:load3_cont 0 c=5.46917e-17
c494 388:load3_cont 226:ln_n46 c=1.00801e-17
c493 388:load3_cont 170:ln_n47 c=9.829e-18
c492 388:load3_cont 1:ln_n124 c=4.4093e-17
cg491 386:load3_cont 0 c=5.68827e-18
c490 386:load3_cont 1:ln_n124 c=3.43735e-18
cg489 385:load3_cont 0 c=2.08364e-18
c488 385:load3_cont 226:ln_n46 c=1.21462e-17
c487 385:load3_cont 233:ln_n126 c=4.04645e-17

c486 385:load3_cont 170:ln_n47 c=1.21483e-17
c485 385:load3_cont 1:ln_n124 c=2.60309e-17
cg484 m23@2|gate 0 c=2.62962e-18
c483 m23@2|gate 1:ln_n124 c=7.392e-17
cg482 m23|gate 0 c=2.89989e-18
c481 m23|gate 1:ln_n124 c=4.48356e-17
cg480 380:load2_cont 0 c=3.73234e-18
c479 380:load2_cont 1:ln_n124 c=4.67039e-18
cg478 379:load2_cont 0 c=1.66148e-17
c477 379:load2_cont 371:load1_cont c=8.97779e-19
c476 379:load2_cont 372:load1_cont c=1.86502e-18
c475 379:load2_cont 1:ln_n124 c=7.4987e-18
cg474 378:load2_cont 0 c=1.18296e-17
c473 378:load2_cont 371:load1_cont c=1.65622e-17
c472 378:load2_cont 1:ln_n124 c=1.27339e-17
cg471 373:load2_cont 0 c=1.92017e-17
c470 373:load2_cont 365:load1_cont c=5.27875e-19
c469 373:load2_cont 371:load1_cont c=2.15626e-17
c468 373:load2_cont 1:ln_n124 c=3.5287e-17
cg467 m22@2|gate 0 c=3.25891e-18
c466 m22@2|gate m21@2|gate c=1.85658e-19
c465 m22@2|gate m21|gate c=6.31794e-19
c464 m22@2|gate 371:load1_cont c=4.05982e-18
c463 m22@2|gate 1:ln_n124 c=7.20861e-17
cg462 m22|gate 0 c=1.41049e-18
c461 m22|gate m21|gate c=6.63482e-19
c460 m22|gate 371:load1_cont c=3.28515e-20
c459 m22|gate 1:ln_n124 c=4.46155e-17
cg458 372:load1_cont 0 c=1.08613e-17
c457 372:load1_cont 1:ln_n124 c=5.75149e-18
c456 372:load1_cont 186:ln_n64 c=3.32171e-18
c455 372:load1_cont 175:ln_n64 c=1.59947e-17
cg454 371:load1_cont 0 c=9.16483e-18
c453 371:load1_cont 186:ln_n64 c=2.53974e-16
c452 371:load1_cont 175:ln_n64 c=4.86198e-17
c451 371:load1_cont 1:ln_n124 c=6.61021e-17
cg450 370:load1_cont 0 c=7.14345e-18
c449 370:load1_cont 1:ln_n124 c=2.02331e-17
cg448 365:load1_cont 0 c=1.13126e-17
c447 365:load1_cont 1:ln_n124 c=1.39602e-17
c446 365:load1_cont 186:ln_n64 c=8.97743e-18
c445 365:load1_cont 175:ln_n64 c=2.3747e-18
cg444 m21@2|gate 0 c=1.37707e-18

c443 m21@2|gate 1:ln_n124 c=8.57441e-17
cg442 m21|gate 0 c=1.0129e-18
c441 m21|gate 1:ln_n124 c=3.22986e-17
cg440 363:vref3 0 c=1.06231e-18
c439 363:vref3 237:vref2 c=7.4204e-17
c438 363:vref3 245:vref2 c=2.07455e-18
c437 363:vref3 256:vref2 c=3.58474e-18
c436 363:vref3 234:vref2 c=1.40388e-18
c435 363:vref3 1:ln_n124 c=4.67399e-18
c434 363:vref3 m1|gate c=4.56422e-18
cg433 362:vref3 0 c=1.24729e-17
c432 362:vref3 1:ln_n124 c=5.46153e-18
c431 362:vref3 vref2 c=3.92597e-19
c430 362:vref3 252:vref2 c=1.19694e-18
cg429 361:vref3 0 c=6.79671e-18
c428 361:vref3 252:vref2 c=4.30955e-18
c427 361:vref3 237:vref2 c=1.39206e-19
c426 361:vref3 vref2 c=2.12078e-18
c425 361:vref3 1:ln_n124 c=1.28634e-17
cg424 360:vref3 0 c=9.77265e-18
c423 360:vref3 252:vref2 c=2.11729e-18
c422 360:vref3 237:vref2 c=2.87595e-17
c421 360:vref3 vref2 c=1.88323e-18
c420 360:vref3 1:ln_n124 c=5.20499e-17
cg419 m2|drn 0 c=4.46186e-19
c418 m2|drn m1|drn c=1.0749e-19
c417 m2|drn 237:vref2 c=9.31961e-19
c416 m2|drn m0|src c=3.39752e-20
c415 m2|drn m5@2|gate c=5.30254e-19
c414 m2|drn 1:ln_n124 c=1.87033e-16
c413 m2|drn m1|gate c=9.04566e-18
cg412 356:vref3 0 c=5.0785e-18
c411 356:vref3 237:vref2 c=1.20616e-17
c410 356:vref3 1:ln_n124 c=9.72742e-18
c409 356:vref3 m1|gate c=1.19651e-19
cg408 vref3 0 c=2.4473e-17
c407 vref3 1:ln_n124 c=1.90115e-17
c406 vref3 252:vref2 c=3.67727e-18
c405 vref3 237:vref2 c=7.35564e-19
c404 vref3 vref2 c=2.30032e-17
cg403 m2|gate 0 c=2.79446e-18
c402 m2|gate 237:vref2 c=6.77054e-19
c401 m2|gate m0|src c=1.9571e-20

c400 m2|gate 1:ln_n124 c=5.1601e-17
c399 m2|gate m1|gate c=9.90511e-18
cg398 352:vref1 0 c=3.67414e-18
c397 352:vref1 253:vref2 c=4.82511e-18
c396 352:vref1 254:vref2 c=1.6396e-17
c395 352:vref1 255:vref2 c=8.05174e-18
c394 352:vref1 256:vref2 c=2.40711e-19
c393 352:vref1 m5@2|gate c=8.51908e-18
c392 352:vref1 m5|gate c=3.73147e-18
c391 352:vref1 234:vref2 c=5.48306e-18
c390 352:vref1 1:ln_n124 c=1.58812e-17
cg389 351:vref1 0 c=2.83062e-17
c388 351:vref1 1:ln_n124 c=2.35387e-17
c387 351:vref1 vref2 c=1.84889e-17
c386 351:vref1 237:vref2 c=4.40406e-19
c385 351:vref1 252:vref2 c=2.471e-18
cg384 350:vref1 0 c=1.27798e-17
c383 350:vref1 m5@2|gate c=4.661e-21
c382 350:vref1 1:ln_n124 c=3.32499e-17
cg381 338:vref1 0 c=1.13692e-17
c380 338:vref1 1:ln_n124 c=1.36792e-17
c379 338:vref1 252:vref2 c=2.99192e-18
c378 338:vref1 vref2 c=3.97236e-18
c377 338:vref1 237:vref2 c=6.02966e-19
cg376 335:vref1 0 c=2.86373e-17
c375 335:vref1 252:vref2 c=6.78478e-19
c374 335:vref1 237:vref2 c=2.11091e-17
c373 335:vref1 m0|src c=4.72393e-19
c372 335:vref1 256:vref2 c=1.77988e-17
c371 335:vref1 vref2 c=8.01063e-19
c370 335:vref1 1:ln_n124 c=7.80623e-17
cg369 334:vref1 0 c=1.24271e-17
c368 334:vref1 253:vref2 c=2.06802e-19
c367 334:vref1 254:vref2 c=4.41702e-19
c366 334:vref1 255:vref2 c=1.39844e-18
c365 334:vref1 m5@2|gate c=2.24654e-18
c364 334:vref1 234:vref2 c=4.28158e-19
c363 334:vref1 1:ln_n124 c=5.4737e-17
cg362 333:vref1 0 c=1.88735e-17
c361 333:vref1 238:vref2 c=4.7859e-19
c360 333:vref1 253:vref2 c=1.66921e-17
c359 333:vref1 237:vref2 c=5.81274e-18
c358 333:vref1 245:vref2 c=4.02173e-19

c357 333:vref1 254:vref2 c=9.00995e-19
c356 333:vref1 255:vref2 c=3.25422e-20
c355 333:vref1 256:vref2 c=5.48683e-17
c354 333:vref1 m5@2|gate c=1.21882e-18
c353 333:vref1 234:vref2 c=5.52835e-18
c352 333:vref1 1:ln_n124 c=5.18528e-17
cg351 m5|drn 0 c=1.61568e-18
c350 m5|drn m1|drn c=1.10793e-19
c349 m5|drn m5@2|gate c=2.45682e-17
c348 m5|drn 1:ln_n124 c=2.43526e-16
cg347 m0|drn 0 c=5.11684e-19
c346 m0|drn 1:ln_n124 c=2.26776e-16
c345 m0|drn m1|drn c=1.99027e-22
cg344 m0|gate 0 c=4.06899e-18
c343 m0|gate 253:vref2 c=1.20277e-19
c342 m0|gate 237:vref2 c=2.99555e-18
c341 m0|gate m0|src c=1.13874e-17
c340 m0|gate 256:vref2 c=6.11871e-18
c339 m0|gate 1:ln_n124 c=6.91512e-17
c338 m0|gate m1|gate c=1.72259e-18
cg337 m15|gate 0 c=5.23095e-18
c336 m15|gate 1:ln_n124 c=1.17361e-16
cg335 331:piezo_inn 0 c=5.35707e-18
c334 331:piezo_inn m31|gate c=1.39186e-18
c333 331:piezo_inn 299:piezo_inp c=3.86861e-18
c332 331:piezo_inn 293:piezo_inp c=2.36789e-17
c331 331:piezo_inn 307:piezo_inp c=1.72144e-17
c330 331:piezo_inn m32|drn c=6.55589e-19
c329 331:piezo_inn 286:piezo_inp c=7.58637e-18
c328 331:piezo_inn 1:ln_n124 c=2.06901e-17
cg327 330:piezo_inn 0 c=1.49338e-18
c326 330:piezo_inn 1:ln_n124 c=3.1067e-18
c325 330:piezo_inn 307:piezo_inp c=1.36235e-17
c324 330:piezo_inn 306:piezo_inp c=3.24556e-18
c323 330:piezo_inn m29|gate c=5.41813e-18
cg322 329:piezo_inn 0 c=5.92855e-19
c321 329:piezo_inn 1:ln_n124 c=4.44426e-18
c320 329:piezo_inn 307:piezo_inp c=4.57222e-18
c319 329:piezo_inn 286:piezo_inp c=2.04979e-18
c318 329:piezo_inn 287:piezo_inp c=7.95318e-19
cg317 327:piezo_inn 0 c=7.18035e-18
c316 327:piezo_inn 1:ln_n124 c=2.09473e-17
c315 327:piezo_inn 307:piezo_inp c=2.70404e-17

c314 327:piezo_inn m31|gate c=3.52417e-18
cg313 324:piezo_inn 0 c=2.80875e-18
c312 324:piezo_inn 287:piezo_inp c=2.26182e-17
c311 324:piezo_inn 305:piezo_inp c=4.24704e-18
c310 324:piezo_inn m30|drn c=5.10147e-19
c309 324:piezo_inn m29|gate c=5.2382e-19
c308 324:piezo_inn 307:piezo_inp c=1.03252e-17
c307 324:piezo_inn 289:piezo_inp c=1.13938e-17
c306 324:piezo_inn 288:piezo_inp c=1.71926e-17
c305 324:piezo_inn 286:piezo_inp c=8.12015e-18
c304 324:piezo_inn 1:ln_n124 c=1.6611e-17
cg303 319:piezo_inn 0 c=2.17854e-18
c302 319:piezo_inn 1:ln_n124 c=6.40744e-18
c301 319:piezo_inn 307:piezo_inp c=3.93983e-18
cg300 317:piezo_inn 0 c=2.02534e-17
c299 317:piezo_inn 1:ln_n124 c=1.31895e-17
c298 317:piezo_inn 307:piezo_inp c=6.65626e-19
c297 317:piezo_inn 306:piezo_inp c=2.42447e-18
c296 317:piezo_inn m29|gate c=1.91267e-19
c295 317:piezo_inn piezo_inp c=2.96696e-17
c294 317:piezo_inn 288:piezo_inp c=2.12736e-19
c293 317:piezo_inn 286:piezo_inp c=2.15772e-18
cg292 315:piezo_inn 0 c=3.79608e-18
c291 315:piezo_inn 307:piezo_inp c=1.02253e-18
c290 315:piezo_inn 1:ln_n124 c=1.12408e-17
cg289 314:piezo_inn 0 c=1.20633e-17
c288 314:piezo_inn m31|gate c=5.54094e-19
c287 314:piezo_inn m29|gate c=4.86605e-19
c286 314:piezo_inn 307:piezo_inp c=1.36414e-17
c285 314:piezo_inn 306:piezo_inp c=9.17845e-18
c284 314:piezo_inn piezo_inp c=6.39828e-19
c283 314:piezo_inn 1:ln_n124 c=3.02164e-17
cg282 313:piezo_inn 0 c=1.83702e-17
c281 313:piezo_inn 287:piezo_inp c=2.71728e-19
c280 313:piezo_inn 307:piezo_inp c=2.51601e-17
c279 313:piezo_inn 288:piezo_inp c=9.58771e-18
c278 313:piezo_inn 297:piezo_inp c=2.12367e-19
c277 313:piezo_inn 306:piezo_inp c=3.90964e-17
c276 313:piezo_inn 286:piezo_inp c=2.28594e-17
c275 313:piezo_inn 1:ln_n124 c=4.469e-17
cg274 312:piezo_inn 0 c=7.34394e-18
c273 312:piezo_inn 1:ln_n124 c=1.61798e-17
c272 312:piezo_inn 307:piezo_inp c=1.24844e-17

cg271 311:piezo_inn 0 c=4.74703e-18
c270 311:piezo_inn 1:ln_n124 c=1.07753e-17
c269 311:piezo_inn 288:piezo_inp c=7.24599e-20
c268 311:piezo_inn piezo_inp c=8.01532e-18
c267 311:piezo_inn m29|gate c=2.12354e-19
c266 m29|drn 1:ln_n124 c=2.33194e-16
c265 m29|drn 307:piezo_inp c=1.6245e-18
c264 m29|drn m29|gate c=1.1947e-17
c263 m29|drn m31|gate c=2.91163e-19
c262 m31|drn 1:ln_n124 c=9.9573e-17
c261 m31|drn 307:piezo_inp c=1.52349e-18
c260 m31|drn m32|drn c=4.65368e-19
c259 m31|drn m31|gate c=1.16916e-17
cg258 m30|gate 0 c=2.15954e-18
c257 m30|gate 287:piezo_inp c=1.05181e-18
c256 m30|gate 305:piezo_inp c=1.98075e-18
c255 m30|gate m30|drn c=1.19305e-17
c254 m30|gate m29|gate c=1.11523e-18
c253 m30|gate 293:piezo_inp c=8.91455e-19
c252 m30|gate 307:piezo_inp c=2.16927e-19
c251 m30|gate 289:piezo_inp c=3.81993e-18
c250 m30|gate 288:piezo_inp c=1.24197e-18
c249 m30|gate 1:ln_n124 c=6.94623e-17
cg248 m32|gate 0 c=2.49904e-18
c247 m32|gate m31|gate c=1.1865e-18
c246 m32|gate 299:piezo_inp c=1.74814e-18
c245 m32|gate 293:piezo_inp c=1.4603e-18
c244 m32|gate 307:piezo_inp c=1.38616e-18
c243 m32|gate m32|drn c=1.16899e-17
c242 m32|gate 1:ln_n124 c=6.19777e-17
cg241 307:piezo_inp 0 c=7.3104e-18
c240 307:piezo_inp 1:ln_n124 c=3.50735e-17
cg239 306:piezo_inp 0 c=4.64868e-18
c238 306:piezo_inp 1:ln_n124 c=2.1702e-17
cg237 305:piezo_inp 0 c=7.02582e-19
c236 305:piezo_inp 1:ln_n124 c=2.48274e-18
cg235 302:piezo_inp 0 c=2.7964e-19
c234 302:piezo_inp 1:ln_n124 c=1.12214e-18
cg233 299:piezo_inp 0 c=1.69716e-18
c232 299:piezo_inp 1:ln_n124 c=3.98962e-18
cg231 297:piezo_inp 0 c=1.69564e-20
c230 297:piezo_inp 1:ln_n124 c=1.2415e-19
cg229 293:piezo_inp 0 c=2.14181e-18

c228 293:piezo_inp 1:ln_n124 c=1.50026e-17
cg227 289:piezo_inp 0 c=2.99675e-18
c226 289:piezo_inp 1:ln_n124 c=1.00101e-17
cg225 288:piezo_inp 0 c=3.46459e-18
c224 288:piezo_inp 1:ln_n124 c=2.02523e-17
cg223 287:piezo_inp 0 c=2.86517e-18
c222 287:piezo_inp 1:ln_n124 c=1.53594e-17
cg221 286:piezo_inp 0 c=3.17417e-17
c220 286:piezo_inp 1:ln_n124 c=3.42944e-17
cg219 piezo_inp 0 c=2.33657e-17
c218 piezo_inp 1:ln_n124 c=1.61763e-17
cg217 m30|drn 0 c=6.05468e-19
c216 m30|drn 1:ln_n124 c=2.27533e-16
cg215 m32|drn 0 c=1.68066e-19
c214 m32|drn 1:ln_n124 c=1.02797e-16
cg213 m29|gate 0 c=2.62968e-18
c212 m29|gate 1:ln_n124 c=6.87491e-17
cg211 m31|gate 0 c=2.87144e-18
c210 m31|gate 1:ln_n124 c=6.30179e-17
cg209 281:rf_inp 0 c=6.16424e-18
c208 281:rf_inp 1:ln_n124 c=1.53788e-17
cg207 280:rf_inp 0 c=5.67567e-18
c206 280:rf_inp 1:ln_n124 c=2.03938e-17
cg205 279:rf_inp 0 c=1.73131e-17
c204 279:rf_inp 1:ln_n124 c=9.80659e-18
cg203 278:rf_inp 0 c=3.66823e-18
c202 278:rf_inp 1:ln_n124 c=5.84226e-18
cg201 277:rf_inp 0 c=1.01294e-17
c200 277:rf_inp 1:ln_n124 c=3.06878e-17
cg199 276:rf_inp 0 c=3.10209e-18
c198 276:rf_inp 1:ln_n124 c=1.751e-17
cg197 275:rf_inp 0 c=6.31171e-19
c196 275:rf_inp 1:ln_n124 c=4.40595e-18
cg195 274:rf_inp 0 c=2.77827e-18
c194 274:rf_inp 1:ln_n124 c=6.44846e-18
cg193 273:rf_inp 0 c=5.59835e-18
c192 273:rf_inp 1:ln_n124 c=8.15826e-18
cg191 269:rf_inp 0 c=4.95984e-18
c190 269:rf_inp 1:ln_n124 c=8.82499e-18
cg189 261:rf_inp 0 c=2.07622e-17
c188 261:rf_inp 1:ln_n124 c=4.11064e-17
c187 m26|drn 1:ln_n124 c=2.32594e-16
c186 m28|drn 1:ln_n124 c=1.00734e-16

cg185 m25|gate 0 c=2.20518e-18
c184 m25|gate 1:ln_n124 c=6.91164e-17
cg183 m27|gate 0 c=2.53554e-18
c182 m27|gate 1:ln_n124 c=6.19341e-17
cg181 256:vref2 0 c=3.05862e-18
c180 256:vref2 1:ln_n124 c=1.28805e-17
cg179 255:vref2 0 c=1.97056e-18
c178 255:vref2 1:ln_n124 c=1.00962e-17
cg177 254:vref2 0 c=1.17888e-18
c176 254:vref2 1:ln_n124 c=7.59252e-18
cg175 253:vref2 0 c=4.10699e-18
c174 253:vref2 1:ln_n124 c=2.90734e-17
cg173 252:vref2 0 c=1.07731e-17
c172 252:vref2 1:ln_n124 c=1.00269e-17
cg171 245:vref2 0 c=1.00945e-18
c170 245:vref2 1:ln_n124 c=1.05054e-17
cg169 238:vref2 0 c=3.11486e-20
c168 238:vref2 1:ln_n124 c=3.76596e-19
cg167 237:vref2 0 c=9.43206e-18
c166 237:vref2 1:ln_n124 c=6.39794e-17
cg165 234:vref2 0 c=2.77553e-17
c164 234:vref2 1:ln_n124 c=4.14679e-17
cg163 vref2 0 c=2.53394e-17
c162 vref2 1:ln_n124 c=1.21118e-17
cg161 m1|drn 0 c=5.9362e-20
c160 m1|drn 1:ln_n124 c=1.64892e-16
cg159 m0|src 0 c=3.88429e-24
c158 m0|src 1:ln_n124 c=2.25925e-16
cg157 m1|gate 0 c=1.44581e-18
c156 m1|gate 1:ln_n124 c=5.81151e-17
cg155 m5@2|gate 0 c=5.76031e-18
c154 m5@2|gate 1:ln_n124 c=1.17562e-16
cg153 m5|gate 0 c=2.10249e-20
c152 m5|gate 1:ln_n124 c=3.61935e-19
cg151 233:ln_n126 0 c=4.12518e-17
c150 233:ln_n126 1:ln_n124 c=3.98173e-17
cg149 232:ln_n126 0 c=3.21024e-17
c148 232:ln_n126 1:ln_n124 c=1.10449e-16
cg147 231:ln_n126 0 c=1.55529e-17
c146 231:ln_n126 1:ln_n124 c=1.85312e-17
cg145 230:ln_n46 0 c=1.97656e-17
c144 230:ln_n46 1:ln_n124 c=2.63763e-17
cg143 226:ln_n46 0 c=1.23391e-17

c142 226:ln_n46 1:ln_n124 c=8.35116e-18
cg141 225:ln_n33 0 c=5.71558e-18
c140 225:ln_n33 72:ln_n32 c=1.06149e-16
c139 225:ln_n33 68:ln_n32 c=7.75074e-18
c138 225:ln_n33 1:ln_n124 c=1.80275e-17
cg137 221:ln_n33 0 c=2.16232e-18
c136 221:ln_n33 72:ln_n32 c=7.47073e-18
c135 221:ln_n33 68:ln_n32 c=1.07621e-16
c134 221:ln_n33 1:ln_n124 c=6.66764e-18
cg133 220:ln_n38 0 c=7.65545e-18
c132 220:ln_n38 1:ln_n124 c=4.44261e-17
cg131 213:ln_n38 0 c=3.43897e-17
c130 213:ln_n38 1:ln_n124 c=1.48159e-17
c129 213:ln_n38 23:ln_n27 c=4.34848e-17
cg128 212:ln_n45 0 c=2.32756e-17
c127 212:ln_n45 1:ln_n124 c=5.17595e-17
cg126 201:ln_n45 0 c=1.48063e-17
c125 201:ln_n45 1:ln_n124 c=2.03719e-17
cg124 200:ln_n80 0 c=9.90584e-18
c123 200:ln_n80 1:ln_n124 c=4.12966e-17
cg122 194:ln_n80 0 c=1.38939e-17
c121 194:ln_n80 1:ln_n124 c=1.50542e-17
cg120 193:ln_n67 0 c=1.52062e-17
c119 193:ln_n67 1:ln_n124 c=2.93867e-17
cg118 187:ln_n67 0 c=2.18024e-17
c117 187:ln_n67 1:ln_n124 c=1.9568e-17
cg116 186:ln_n64 0 c=3.26323e-17
c115 186:ln_n64 1:ln_n124 c=6.31505e-17
cg114 175:ln_n64 0 c=4.09759e-17
c113 175:ln_n64 1:ln_n124 c=1.8561e-17
cg112 174:ln_n47 0 c=6.00952e-18
c111 174:ln_n47 1:ln_n124 c=3.27016e-17
cg110 170:ln_n47 0 c=2.77072e-18
c109 170:ln_n47 1:ln_n124 c=7.20995e-18
cg108 169:ln_n44 0 c=7.21596e-18
c107 169:ln_n44 164:ln_n42 c=1.25978e-17
c106 169:ln_n44 160:ln_n42 c=7.19912e-18
c105 169:ln_n44 1:ln_n124 c=3.03881e-17
cg104 165:ln_n44 0 c=5.1157e-18
c103 165:ln_n44 164:ln_n42 c=7.19448e-18
c102 165:ln_n44 160:ln_n42 c=1.86739e-17
c101 165:ln_n44 1:ln_n124 c=1.28492e-17
cg100 164:ln_n42 0 c=8.09658e-18

c99 164:ln_n42 1:ln_n124 c=2.9506e-17
cg98 160:ln_n42 0 c=3.97091e-18
c97 160:ln_n42 1:ln_n124 c=1.25098e-17
cg96 159:ln_n60 0 c=2.64596e-17
c95 159:ln_n60 1:ln_n124 c=7.23531e-17
cg94 147:ln_n60 0 c=3.16991e-17
c93 147:ln_n60 1:ln_n124 c=3.48847e-17
cg92 146:ln_n30 0 c=9.71517e-17
c91 146:ln_n30 109:ln_n31 c=3.90771e-18
c90 146:ln_n30 1:ln_n124 c=4.39249e-17
c89 146:ln_n30 117:ln_n31 c=5.33125e-17
cg88 135:ln_n30 0 c=9.39745e-17
c87 135:ln_n30 109:ln_n31 c=6.31325e-17
c86 135:ln_n30 1:ln_n124 c=1.40976e-17
c85 135:ln_n30 117:ln_n31 c=3.90771e-18
cg84 134:ln_n36 0 c=1.8566e-17
c83 134:ln_n36 95:ln_n37 c=5.13911e-17
c82 134:ln_n36 87:ln_n37 c=3.81245e-18
c81 134:ln_n36 1:ln_n124 c=6.27667e-17
cg80 123:ln_n36 0 c=3.26799e-17
c79 123:ln_n36 95:ln_n37 c=2.91573e-18
c78 123:ln_n36 1:ln_n124 c=2.68182e-17
c77 123:ln_n36 87:ln_n37 c=6.23013e-17
cg76 122:ln_n34 0 c=5.56007e-18
c75 122:ln_n34 1:ln_n124 c=1.8183e-17
cg74 118:ln_n34 0 c=2.58344e-18
c73 118:ln_n34 1:ln_n124 c=8.48686e-18
cg72 117:ln_n31 0 c=6.17872e-17
c71 117:ln_n31 6:ln_n24 c=3.90771e-18
c70 117:ln_n31 17:ln_n24 c=5.33125e-17
c69 117:ln_n31 1:ln_n124 c=2.77483e-17
cg68 109:ln_n31 0 c=5.28051e-17
c67 109:ln_n31 6:ln_n24 c=6.31325e-17
c66 109:ln_n31 17:ln_n24 c=3.90771e-18
c65 109:ln_n31 1:ln_n124 c=8.71412e-18
cg64 108:ln_n52 0 c=4.85278e-17
c63 108:ln_n52 1:ln_n124 c=7.54156e-17
cg62 96:ln_n52 0 c=2.91281e-17
c61 96:ln_n52 1:ln_n124 c=3.61074e-17
cg60 95:ln_n37 0 c=6.17003e-18
c59 95:ln_n37 34:ln_n27 c=5.20309e-17
c58 95:ln_n37 1:ln_n124 c=3.61996e-17
c57 95:ln_n37 23:ln_n27 c=2.8607e-18

cg56 87:ln_n37 0 c=5.87097e-17
c55 87:ln_n37 34:ln_n27 c=2.89011e-18
c54 87:ln_n37 1:ln_n124 c=1.59164e-17
c53 87:ln_n37 23:ln_n27 c=5.57421e-17
cg52 86:ln_n54 0 c=1.24096e-17
c51 86:ln_n54 60:ln_n55 c=1.36619e-17
c50 86:ln_n54 67:ln_n55 c=7.24892e-17
c49 86:ln_n54 1:ln_n124 c=3.28634e-17
cg48 80:ln_n54 0 c=1.96351e-17
c47 80:ln_n54 60:ln_n55 c=7.92421e-17
c46 80:ln_n54 67:ln_n55 c=1.3712e-17
c45 80:ln_n54 1:ln_n124 c=1.23102e-17
cg44 79:ln_n56 0 c=1.22746e-17
c43 79:ln_n56 1:ln_n124 c=4.64855e-17
cg42 73:ln_n56 0 c=1.18333e-17
c41 73:ln_n56 1:ln_n124 c=1.78425e-17
cg40 72:ln_n32 0 c=5.78876e-18
c39 72:ln_n32 1:ln_n124 c=1.79543e-17
cg38 68:ln_n32 0 c=3.42846e-18
c37 68:ln_n32 1:ln_n124 c=5.05435e-18
cg36 67:ln_n55 0 c=4.92856e-18
c35 67:ln_n55 1:ln_n124 c=4.04992e-17
cg34 60:ln_n55 0 c=4.14303e-18
c33 60:ln_n55 1:ln_n124 c=1.32076e-17
cg32 59:ln_n48 0 c=6.26769e-17
c31 59:ln_n48 46:ln_n25 c=2.59244e-17
c30 59:ln_n48 35:ln_n25 c=4.35674e-18
c29 59:ln_n48 1:ln_n124 c=9.12369e-17
cg28 47:ln_n48 0 c=7.14213e-17
c27 47:ln_n48 46:ln_n25 c=4.35136e-18
c26 47:ln_n48 35:ln_n25 c=3.624e-17
c25 47:ln_n48 1:ln_n124 c=3.37877e-17
cg24 46:ln_n25 0 c=6.61172e-17
c23 46:ln_n25 1:ln_n124 c=7.80771e-17
cg22 35:ln_n25 0 c=8.51932e-17
c21p 35:ln_n25 1:ln_n124 c=2.93394e-17
cg20 34:ln_n27 0 c=2.33359e-17
c19p 34:ln_n27 1:ln_n124 c=6.31694e-17
cg18 23:ln_n27 0 c=2.18434e-17
c17p 23:ln_n27 1:ln_n124 c=2.03644e-17
cg16 22:ln_n26 0 c=2.32273e-17
c15p 22:ln_n26 1:ln_n124 c=2.68591e-17
cg14 18:ln_n26 0 c=2.84489e-17

c13 18:ln_n26 1:ln_n124 c=1.26376e-17
cg12 17:ln_n24 0 c=9.42877e-17
c11 17:ln_n24 1:ln_n124 c=4.2914e-17
cg10 6:ln_n24 0 c=9.36802e-17
c9 6:ln_n24 1:ln_n124 c=9.931e-18
cg8 5:ln_n128 0 c=1.19637e-17
c7 5:ln_n128 1:ln_n124 c=6.88894e-18
cg6 4:ln_n128 0 c=9.26145e-17
c5 4:ln_n128 1:ln_n124 c=1.29945e-16
cg4 3:ln_n_generated_130 0 c=8.17969e-17
c3 3:ln_n_generated_130 1:ln_n124 c=7.36658e-17
cg2 2:ln_n_generated_129 0 c=9.25742e-17
c1p 2:ln_n_generated_129 1:ln_n124 c=8.42193e-17
r1776 1194:net43 1196:net43 r=1.07036
r1775 1190:net43 1192:net43 r=0.205714
r1774 1188:net43 1189:net43 r=0.604286
r1773 m10|src m11|src r=0.001
r1772 1187:net43 net43 r=0.0739286
r1771 1185:net43 1193:net43 r=0.045
r1770 1184:net43 1186:net43 r=0.045
r1769 1184:net43 1185:net43 r=0.1386
r1768 1183:net43 1192:net43 r=0.241072
r1767 1182:net43 1191:net43 r=1.125
r1766 1182:net43 1183:net43 r=0.324643
r1765 1180:net43 1181:net43 r=0.0739286
r1764 1179:net43 1189:net43 r=0.636429
r1763 1179:net43 1180:net43 r=0.295715
r1762 1178:net43 1188:net43 r=0.636429
r1761 1177:net43 1184:net43 r=10
r1760 1177:net43 1185:net43 r=10
r1759 1176:net43 1187:net43 r=0.311272
r1758 1176:net43 1196:net43 r=0.106072
r1757 1174:net43 m10|src r=10
r1756 1174:net43 1195:net43 r=0.155893
r1755 1173:net43 1191:net43 r=0.671786
r1754 1172:net43 1178:net43 r=0.311272
r1753 1172:net43 1173:net43 r=0.115714
r1752 m15@2|drn 1178:net43 r=10
r1751 m15@2|drn 1172:net43 r=10
r1750 m9|src 1187:net43 r=10
r1749 m9|src 1176:net43 r=10
r1748 1171:net43 1193:net43 r=0.0997827
r1747 1171:net43 1195:net43 r=0.430715

r1746 1171:net43 1194:net43 r=0.170357
r1745 1170:net43 1175:net43 r=0.655715
r1744 1170:net43 1174:net43 r=0.0487975
r1743 m14|drn 1182:net43 r=10
r1742 m14|drn 1183:net43 r=10
r1741 1169:net43 1190:net43 r=0.315
r1740 1169:net43 1175:net43 r=0.353572
r1739 m8|src 1175:net43 r=10
r1738 m8|src 1169:net43 r=10
r1737 m15|drn 1179:net43 r=10
r1736 m15|drn 1180:net43 r=10
r1735 1164:control_p control_p r=0.388125
r1734 1162:control_p 1163:control_p r=0.0739286
r1733 1161:control_p 1167:control_p r=0.183214
r1732 1159:control_p 1165:control_p r=0.286072
r1731 1159:control_p 1160:control_p r=0.0739286
r1730 1158:control_p control_p r=0.225
r1729 1155:control_p 1157:control_p r=0.295313
r1728 1155:control_p 1156:control_p r=0.0675001
r1727 1154:control_p 1161:control_p r=0.196457
r1726 1153:control_p 1168:control_p r=0.0720001
r1725 1153:control_p 1155:control_p r=0.900001
r1724 1152:control_p 1153:control_p r=0.0720001
r1723 1152:control_p 1162:control_p r=0.27
r1722 1151:control_p 1164:control_p r=0.264375
r1721 1150:control_p 1157:control_p r=0.548438
r1720 1150:control_p 1151:control_p r=0.253125
r1719 1149:control_p 1166:control_p r=0.0739286
r1718 1149:control_p 1154:control_p r=0.192857
r1717 m11|drn 1149:control_p r=10
r1716 m6|drn 1162:control_p r=10
r1715 m11|gate 1161:control_p r=8
r1714 m8|gate 1159:control_p r=8
r1713 1148:control_p 1154:control_p r=1.12179
r1712 1148:control_p 1158:control_p r=0.900001
r1711 1148:control_p 1165:control_p r=0.0780001
r1710 1141:vol_loopback 1142:vol_loopback r=0.0234
r1709 1139:vol_loopback 1140:vol_loopback r=0.0216
r1708 1136:vol_loopback 1138:vol_loopback r=0.0675001
r1707 1135:vol_loopback vol_loopback r=0.332609
r1706 1134:vol_loopback 1146:vol_loopback r=0.01035
r1705 1134:vol_loopback 1142:vol_loopback r=0.01125
r1704 1133:vol_loopback 1144:vol_loopback r=0.0045

r1703 1133:vol_loopback 1135:vol_loopback r=0.109565
r1702 1133:vol_loopback 1134:vol_loopback r=0.900001
r1701 1132:vol_loopback vol_loopback r=0.107609
r1700 1130:vol_loopback 1137:vol_loopback r=0.0720001
r1699 1130:vol_loopback 1136:vol_loopback r=0.900001
r1698 1129:vol_loopback 1130:vol_loopback r=0.0720001
r1697 1129:vol_loopback 1145:vol_loopback r=0.0527587
r1696 1128:vol_loopback 1131:vol_loopback r=0.0675001
r1695 1127:vol_loopback 1141:vol_loopback r=0.00540001
r1694 1127:vol_loopback 1139:vol_loopback r=0.018
r1693 1126:vol_loopback 1128:vol_loopback r=0.810001
r1692 1126:vol_loopback 1136:vol_loopback r=0.525938
r1691 1125:vol_loopback 1127:vol_loopback r=0.900001
r1690 1123:vol_loopback 1124:vol_loopback r=0.823126
r1689 1123:vol_loopback 1125:vol_loopback r=0.900001
r1688 1122:vol_loopback 1124:vol_loopback r=0.12375
r1687 1122:vol_loopback 1128:vol_loopback r=0.900001
r1686 d9|cathode 1142:vol_loopback r=3.33333
r1685 d9|cathode 1141:vol_loopback r=3.33333
r1684 d9|cathode 1139:vol_loopback r=3.33333
r1683 1121:vol_loopback 1147:vol_loopback r=0.029325
r1682 1121:vol_loopback 1143:vol_loopback r=0.029325
r1681 1121:vol_loopback 1132:vol_loopback r=0.900001
r1680 d10|cathode 1121:vol_loopback r=10
r1679 m10|gate 1145:vol_loopback r=8
r1678 cont_n3 1115:cont_n3 r=0.233438
r1677 1113:cont_n3 1118:cont_n3 r=0.0273913
r1676 1113:cont_n3 1114:cont_n3 r=0.0273913
r1675 1112:cont_n3 1116:cont_n3 r=0.212143
r1674 1110:cont_n3 1112:cont_n3 r=0.295715
r1673 1110:cont_n3 1111:cont_n3 r=0.0739286
r1672 1108:cont_n3 1109:cont_n3 r=0.0739286
r1671 1107:cont_n3 1120:cont_n3 r=0.0540001
r1670 1106:cont_n3 1107:cont_n3 r=0.0900001
r1669 1105:cont_n3 1106:cont_n3 r=0.0900001
r1668 1104:cont_n3 1105:cont_n3 r=0.0900001
r1667 1103:cont_n3 1104:cont_n3 r=0.0900001
r1666 1102:cont_n3 1103:cont_n3 r=0.0900001
r1665 1101:cont_n3 1102:cont_n3 r=0.0900001
r1664 1100:cont_n3 1101:cont_n3 r=0.0900001
r1663 1099:cont_n3 1100:cont_n3 r=0.0900001
r1662 1096:cont_n3 cont_n3 r=0.165938
r1661 1095:cont_n3 1097:cont_n3 r=0.368438

r1660 1095:cont_n3 1096:cont_n3 r=0.4725
r1659 1093:cont_n3 1117:cont_n3 r=0.0720001
r1658 1093:cont_n3 1116:cont_n3 r=0.0720001
r1657 1092:cont_n3 1094:cont_n3 r=0.0675001
r1656 1092:cont_n3 1093:cont_n3 r=0.900001
r1655 1089:cont_n3 1119:cont_n3 r=0.0720001
r1654 1089:cont_n3 1095:cont_n3 r=0.900001
r1653 1088:cont_n3 1089:cont_n3 r=0.0720001
r1652 1087:cont_n3 1108:cont_n3 r=0.748929
r1651 1087:cont_n3 1088:cont_n3 r=0.0713794
r1650 1087:cont_n3 1118:cont_n3 r=0.414643
r1649 1086:cont_n3 1099:cont_n3 r=0.0900001
r1648 1086:cont_n3 1098:cont_n3 r=0.0540001
r1647 1085:cont_n3 1115:cont_n3 r=0.2235
r1646 1084:cont_n3 1097:cont_n3 r=0.188438
r1645 1084:cont_n3 1092:cont_n3 r=0.340313
r1644 m24|drn 1113:cont_n3 r=10
r1643 m8|drn 1112:cont_n3 r=10
r1642 m8|drn 1110:cont_n3 r=10
r1641 m9|gate 1108:cont_n3 r=8
r1640 c15|plus 1090:cont_n3 r=0.648001
r1639 c15|plus 1091:cont_n3 r=0.693001
r1638 c15|plus 1086:cont_n3 r=0.900001
r1637 c15|plus 1099:cont_n3 r=0.900001
r1636 c15|plus 1100:cont_n3 r=0.900001
r1635 c15|plus 1101:cont_n3 r=0.900001
r1634 c15|plus 1085:cont_n3 r=0.297
r1633 c15|plus 1102:cont_n3 r=0.900001
r1632 c15|plus 1103:cont_n3 r=0.900001
r1631 c15|plus 1104:cont_n3 r=0.900001
r1630 c15|plus 1105:cont_n3 r=0.900001
r1629 c15|plus 1106:cont_n3 r=0.900001
r1628 c15|plus 1107:cont_n3 r=0.900001
r1627 1077:load3 1081:load3 r=0.311786
r1626 1076:load3 1079:load3 r=0.311786
r1625 1074:load3 1077:load3 r=0.295715
r1624 1074:load3 1075:load3 r=0.0739286
r1623 1073:load3 1083:load3 r=0.0739286
r1622 1073:load3 1076:load3 r=0.295715
r1621 1070:load3 1072:load3 r=0.0690001
r1620 1069:load3 1070:load3 r=0.900001
r1619 1069:load3 1071:load3 r=0.0630001
r1618 1069:load3 1078:load3 r=0.0225

r1617 1068:load3 1079:load3 r=0.315
r1616 1068:load3 1081:load3 r=0.289286
r1615 1067:load3 1070:load3 r=0.350011
r1614 1066:load3 1082:load3 r=0.045
r1613 1066:load3 load3 r=0.0288282
r1612 1065:load3 1067:load3 r=1.84655
r1611 1064:load3 1066:load3 r=0.900001
r1610 1064:load3 1065:load3 r=0.0937501
r1609 m23@2|drn 1077:load3 r=10
r1608 m23@2|drn 1074:load3 r=10
r1607 m23|drn 1076:load3 r=10
r1606 m23|drn 1073:load3 r=10
r1605 1063:load3 1068:load3 r=0.109465
r1604 1063:load3 1069:load3 r=0.900001
r1603 1063:load3 1080:load3 r=0.0955637
r1602 1052:primary_storage 1053:primary_storage r=0.0960001
r1601 1051:primary_storage 1052:primary_storage r=0.141
r1600 1050:primary_storage 1051:primary_storage r=0.153
r1599 1049:primary_storage 1050:primary_storage r=0.141
r1598 1048:primary_storage 1049:primary_storage r=0.1575
r1597 m15|src m15@2|src r=0.001
r1596 1046:primary_storage 1060:primary_storage r=0.925715
r1595 1046:primary_storage 1047:primary_storage r=0.0739286
r1594 1045:primary_storage 1058:primary_storage r=0.858215
r1593 1044:primary_storage 1045:primary_storage r=0.0656251
r1592 1043:primary_storage 1044:primary_storage r=0.1275
r1591 1042:primary_storage 1043:primary_storage r=0.1275
r1590 1041:primary_storage 1042:primary_storage r=0.1275
r1589 1040:primary_storage 1061:primary_storage r=0.141429
r1588 1040:primary_storage 1057:primary_storage r=0.154286
r1587 1039:primary_storage 1059:primary_storage r=0.0739286
r1586 1036:primary_storage 1038:primary_storage r=0.0739286
r1585 1036:primary_storage 1037:primary_storage r=0.0160714
r1584 1034:primary_storage 1035:primary_storage r=0.0739286
r1583 1032:primary_storage 1056:primary_storage r=0.0353572
r1582 1032:primary_storage 1033:primary_storage r=0.0739286
r1581 1031:primary_storage m15|src r=10
r1580 1029:primary_storage m15|src r=10
r1579 1029:primary_storage 1030:primary_storage r=0.286072
r1578 1028:primary_storage 1062:primary_storage r=0.0739286
r1577 1028:primary_storage 1055:primary_storage r=0.99643
r1576 1026:primary_storage 1027:primary_storage r=0.0739286
r1575 1023:primary_storage 1025:primary_storage r=0.0739286

r1574 1023:primary_storage 1024:primary_storage r=0.0771429
r1573 1021:primary_storage 1022:primary_storage r=0.0739286
r1572 1019:primary_storage 1054:primary_storage r=0.0385715
r1571 1019:primary_storage 1020:primary_storage r=0.0739286
r1570 1013:primary_storage 1055:primary_storage r=0.0720001
r1569 1013:primary_storage 1014:primary_storage r=0.0720001
r1568 1012:primary_storage 1016:primary_storage r=0.607501
r1567 1012:primary_storage 1015:primary_storage r=0.0703126
r1566 1012:primary_storage 1013:primary_storage r=0.900001
r1565 1011:primary_storage 1041:primary_storage r=10
r1564 1011:primary_storage 1042:primary_storage r=10
r1563 1011:primary_storage 1043:primary_storage r=10
r1562 1011:primary_storage 1044:primary_storage r=10
r1561 1010:primary_storage 1056:primary_storage r=0.0720001
r1560 1009:primary_storage 1058:primary_storage r=0.0932144
r1559 1009:primary_storage 1039:primary_storage r=0.324643
r1558 1008:primary_storage 1010:primary_storage r=0.0720001
r1557 1008:primary_storage 1034:primary_storage r=0.106072
r1556 1007:primary_storage 1029:primary_storage r=0.27
r1555 1007:primary_storage 1031:primary_storage r=0.024
r1554 1006:primary_storage 1024:primary_storage r=0.0720001
r1553 1005:primary_storage 1006:primary_storage r=0.0720001
r1552 1005:primary_storage 1026:primary_storage r=0.0642858
r1551 1004:primary_storage 1054:primary_storage r=0.0720001
r1550 1003:primary_storage 1010:primary_storage r=0.900001
r1549 1002:primary_storage 1003:primary_storage r=0.164507
r1548 1001:primary_storage 1048:primary_storage r=0.204
r1547 1000:primary_storage 1031:primary_storage r=0.048
r1546 1000:primary_storage 1018:primary_storage r=0.0720001
r1545 999:primary_storage 1030:primary_storage r=0.0720001
r1544 999:primary_storage 1017:primary_storage r=0.0720001
r1543 998:primary_storage 1000:primary_storage r=0.900001
r1542 998:primary_storage 1001:primary_storage r=0.0630001
r1541 997:primary_storage 1012:primary_storage r=0.444375
r1540 996:primary_storage 999:primary_storage r=0.900001
r1539 996:primary_storage 1016:primary_storage r=0.171563
r1538 994:primary_storage primary_storage r=0.0682032
r1537 993:primary_storage 1006:primary_storage r=0.900001
r1536 993:primary_storage 995:primary_storage r=0.166278
r1535 992:primary_storage 993:primary_storage r=0.25347
r1534 991:primary_storage 992:primary_storage r=0.183234
r1533 990:primary_storage 1004:primary_storage r=0.900001
r1532 990:primary_storage 991:primary_storage r=0.216416

r1531 989:primary_storage 1041:primary_storage r=0.0543751
r1530 989:primary_storage 1060:primary_storage r=0.565715
r1529 989:primary_storage 1061:primary_storage r=0.858215
r1528 m5|src 1040:primary_storage r=10
r1527 m5|src 1036:primary_storage r=10
r1526 m21|src m21@2|src r=0.001
r1525 m21|src 1026:primary_storage r=10
r1524 m21|src 1023:primary_storage r=10
r1523 m22|src m22@2|src r=0.001
r1522 m22|src 1021:primary_storage r=10
r1521 m22|src 1019:primary_storage r=10
r1520 988:primary_storage 1004:primary_storage r=0.0720001
r1519 988:primary_storage 1021:primary_storage r=0.102857
r1518 m20|drn 1046:primary_storage r=10
r1517 m5@2|src 1009:primary_storage r=10
r1516 m5@2|src 1039:primary_storage r=10
r1515 m23|src 1034:primary_storage r=10
r1514 m23|src m23@2|src r=0.001
r1513 m23|src 1032:primary_storage r=10
r1512 m7|drn 1028:primary_storage r=10
r1511 980:primary_storage 997:primary_storage r=0.43875
r1510 979:primary_storage 1057:primary_storage r=0.0696775
r1509 979:primary_storage 1037:primary_storage r=0.0696775
r1508 979:primary_storage 980:primary_storage r=0.900001
r1507 977:primary_storage 978:primary_storage r=0.486
r1506 976:primary_storage 977:primary_storage r=0.2265
r1505 975:primary_storage 995:primary_storage r=0.402293
r1504 975:primary_storage 1053:primary_storage r=0.0553501
r1503 974:primary_storage 975:primary_storage r=0.23994
r1502 974:primary_storage 976:primary_storage r=0.22635
r1501 974:primary_storage 1002:primary_storage r=0.222304
r1500 973:primary_storage 978:primary_storage r=0.2745
r1499 c18|plus 973:primary_storage r=0.186693
r1498 c18|plus 983:primary_storage r=0.348
r1497 c18|plus 984:primary_storage r=0.348
r1496 c8|plus 973:primary_storage r=0.183763
r1495 c16|plus 981:primary_storage r=0.348
r1494 c16|plus 982:primary_storage r=0.348
r1493 c16|plus 977:primary_storage r=0.1845
r1492 c16|plus 994:primary_storage r=0.177
r1491 c17|plus c18|plus r=0.001
r1490 c17|plus 985:primary_storage r=0.348
r1489 c17|plus c19|plus r=0.001

r1488 c17|plus 986:primary_storage r=0.348
r1487 c17|plus 987:primary_storage r=0.348
r1486 vsec_storage 971:vsec_storage r=0.263572
r1485 966:vsec_storage 968:vsec_storage r=0.045
r1484 966:vsec_storage 967:vsec_storage r=0.0990001
r1483 964:vsec_storage 969:vsec_storage r=0.315
r1482 964:vsec_storage 965:vsec_storage r=0.0739286
r1481 962:vsec_storage 963:vsec_storage r=0.0739286
r1480 960:vsec_storage 971:vsec_storage r=0.151072
r1479 960:vsec_storage 961:vsec_storage r=0.0739286
r1478 958:vsec_storage vsec_storage r=0.707144
r1477 958:vsec_storage 969:vsec_storage r=0.379286
r1476 958:vsec_storage 970:vsec_storage r=0.691072
r1475 957:vsec_storage 966:vsec_storage r=10
r1474 957:vsec_storage 967:vsec_storage r=10
r1473 954:vsec_storage 967:vsec_storage r=0.045
r1472 954:vsec_storage 970:vsec_storage r=0.331072
r1471 953:vsec_storage 968:vsec_storage r=1.19893
r1470 953:vsec_storage 972:vsec_storage r=0.167143
r1469 952:vsec_storage 953:vsec_storage r=0.366429
r1468 952:vsec_storage 962:vsec_storage r=0.343929
r1467 951:vsec_storage 959:vsec_storage r=0.0720001
r1466 950:vsec_storage 972:vsec_storage r=0.401786
r1465 950:vsec_storage 951:vsec_storage r=0.0720001
r1464 949:vsec_storage 951:vsec_storage r=0.900001
r1463 m29|src 962:vsec_storage r=10
r1462 m29|src 952:vsec_storage r=10
r1461 m30|src 964:vsec_storage r=10
r1460 m30|src 969:vsec_storage r=10
r1459 m7|src vsec_storage r=10
r1458 m7|gate 960:vsec_storage r=8
r1457 c1|plus 956:vsec_storage r=0.497728
r1456 c1|plus 949:vsec_storage r=0.0504546
r1455 c1|plus 955:vsec_storage r=0.452728
r1454 ln_n61 948:ln_n61 r=0.0368182
r1453 947:ln_n61 ln_n61 r=0.0818183
r1452 946:ln_n61 947:ln_n61 r=0.0818183
r1451 945:ln_n61 946:ln_n61 r=0.0818183
r1450 944:ln_n61 945:ln_n61 r=0.0818183
r1449 943:ln_n61 944:ln_n61 r=0.0818183
r1448 942:ln_n61 943:ln_n61 r=0.0818183
r1447 941:ln_n61 942:ln_n61 r=0.0818183
r1446 940:ln_n61 941:ln_n61 r=0.0818183

r1445 938:ln_n61 940:ln_n61 r=0.0818183
r1444 937:ln_n61 939:ln_n61 r=0.0368182
r1443 937:ln_n61 938:ln_n61 r=0.0818183
r1442 936:ln_n61 943:ln_n61 r=0.900001
r1441 936:ln_n61 944:ln_n61 r=0.900001
r1440 936:ln_n61 945:ln_n61 r=0.900001
r1439 936:ln_n61 946:ln_n61 r=0.900001
r1438 936:ln_n61 947:ln_n61 r=0.900001
r1437 936:ln_n61 ln_n61 r=0.900001
r1436 936:ln_n61 937:ln_n61 r=0.900001
r1435 936:ln_n61 938:ln_n61 r=0.900001
r1434 936:ln_n61 940:ln_n61 r=0.900001
r1433 936:ln_n61 941:ln_n61 r=0.900001
r1432 936:ln_n61 942:ln_n61 r=0.900001
r1431 m3|drn m2|src r=0.001
r1430 net36 935:net36 r=0.0739286
r1429 net36 m3|drn r=10
r1428 933:net36 934:net36 r=0.244286
r1427 933:net36 m3|drn r=10
r1426 933:net36 net36 r=0.167143
r1425 931:net36 934:net36 r=0.151072
r1424 931:net36 932:net36 r=0.0739286
r1423 m3|gate 931:net36 r=8
r1422 m4|drn m3|src r=0.001
r1421 net37 929:net37 r=0.244286
r1420 net37 m4|drn r=10
r1419 928:net37 930:net37 r=0.0739286
r1418 928:net37 m4|drn r=10
r1417 928:net37 net37 r=0.167143
r1416 926:net37 929:net37 r=0.151072
r1415 926:net37 927:net37 r=0.0739286
r1414 m4|gate 926:net37 r=8
r1413 915:gnd 916:gnd r=0.147857
r1412 909:gnd 912:gnd r=0.829287
r1411 905:gnd 906:gnd r=0.545626
r1410 901:gnd 903:gnd r=0.0273913
r1409 901:gnd 902:gnd r=0.0273913
r1408 899:gnd 917:gnd r=0.366429
r1407 899:gnd 900:gnd r=0.0739286
r1406 897:gnd 915:gnd r=0.176786
r1405 897:gnd 898:gnd r=0.0739286
r1404 895:gnd 914:gnd r=0.0273913
r1403 895:gnd 896:gnd r=0.0273913

r1402 893:gnd 913:gnd r=0.340715
r1401 893:gnd 894:gnd r=0.0739286
r1400 891:gnd 892:gnd r=0.101739
r1399 890:gnd 891:gnd r=0.101739
r1398 889:gnd 911:gnd r=0.0469566
r1397 889:gnd 890:gnd r=0.101739
r1396 888:gnd 911:gnd r=0.148696
r1395 887:gnd 888:gnd r=0.101739
r1394 885:gnd 886:gnd r=0.101739
r1393 884:gnd 885:gnd r=0.101739
r1392 883:gnd 884:gnd r=0.101739
r1391 881:gnd 882:gnd r=0.0739286
r1390 878:gnd 880:gnd r=0.225
r1389 878:gnd 879:gnd r=0.0739286
r1388 876:gnd 908:gnd r=0.366429
r1387 876:gnd 877:gnd r=0.0739286
r1386 874:gnd 875:gnd r=0.0273913
r1385 871:gnd 873:gnd r=0.045
r1384 869:gnd 889:gnd r=10
r1383 869:gnd 890:gnd r=10
r1382 869:gnd 891:gnd r=10
r1381 869:gnd 892:gnd r=10
r1380 869:gnd 883:gnd r=10
r1379 869:gnd 884:gnd r=10
r1378 869:gnd 885:gnd r=10
r1377 869:gnd 886:gnd r=10
r1376 869:gnd 887:gnd r=10
r1375 869:gnd 888:gnd r=10
r1374 867:gnd 868:gnd r=0.0900001
r1373 861:gnd 863:gnd r=0.0900001
r1372 860:gnd 870:gnd r=0.0540001
r1371 859:gnd 861:gnd r=0.0900001
r1370 858:gnd 866:gnd r=0.0818183
r1369 857:gnd 865:gnd r=0.0818183
r1368 856:gnd 864:gnd r=0.0818183
r1367 855:gnd 863:gnd r=0.129
r1366 854:gnd 858:gnd r=0.0818183
r1365 853:gnd 857:gnd r=0.0818183
r1364 852:gnd 856:gnd r=0.0818183
r1363 851:gnd 854:gnd r=0.0818183
r1362 850:gnd 853:gnd r=0.0818183
r1361 849:gnd 852:gnd r=0.0818183
r1360 847:gnd 848:gnd r=0.0900001

r1359 845:gnd 847:gnd r=0.0900001
r1358 844:gnd 924:gnd r=0.0368182
r1357 842:gnd 907:gnd r=0.643501
r1356 837:gnd 838:gnd r=0.0900001
r1355 835:gnd 836:gnd r=0.0900001
r1354 834:gnd 840:gnd r=0.0540001
r1353 831:gnd 839:gnd r=0.051
r1352 827:gnd 828:gnd r=0.0900001
r1351 825:gnd 826:gnd r=0.0900001
r1350 823:gnd 824:gnd r=0.0900001
r1349 822:gnd 825:gnd r=0.0900001
r1348 821:gnd 826:gnd r=0.129
r1347 819:gnd 830:gnd r=0.051
r1346 818:gnd 823:gnd r=0.0900001
r1345 817:gnd 829:gnd r=0.0540001
r1344 816:gnd 827:gnd r=0.0900001
r1343 813:gnd 905:gnd r=0.855001
r1342 812:gnd 813:gnd r=0.922501
r1341 806:gnd 807:gnd r=0.0368182
r1340 803:gnd 804:gnd r=0.0900001
r1339 801:gnd 802:gnd r=0.0900001
r1338 797:gnd 800:gnd r=0.0818183
r1337 796:gnd 799:gnd r=0.0818183
r1336 795:gnd 798:gnd r=0.0818183
r1335 793:gnd 794:gnd r=0.0900001
r1334 791:gnd 792:gnd r=0.0900001
r1333 790:gnd 797:gnd r=0.0818183
r1332 789:gnd 796:gnd r=0.0818183
r1331 788:gnd 795:gnd r=0.0818183
r1330 787:gnd 793:gnd r=0.0900001
r1329 786:gnd 790:gnd r=0.0818183
r1328 785:gnd 789:gnd r=0.0818183
r1327 784:gnd 788:gnd r=0.0818183
r1326 783:gnd 922:gnd r=0.0368182
r1325 783:gnd 805:gnd r=0.0818183
r1324 782:gnd 794:gnd r=0.129
r1323 780:gnd 781:gnd r=0.0900001
r1322 778:gnd 779:gnd r=0.0900001
r1321 777:gnd 780:gnd r=0.0900001
r1320 773:gnd 792:gnd r=0.129
r1319 772:gnd 809:gnd r=0.0540001
r1318 767:gnd 769:gnd r=0.0720001
r1317 767:gnd 768:gnd r=0.0720001

r1316 766:gnd 770:gnd r=0.049091
r1315 766:gnd 767:gnd r=0.900001
r1314 764:gnd 907:gnd r=0.2385
r1313 764:gnd 765:gnd r=0.036
r1312 763:gnd 904:gnd r=0.11625
r1311 763:gnd 764:gnd r=0.900001
r1310 759:gnd 761:gnd r=0.129375
r1309 757:gnd 872:gnd r=0.0720001
r1308 757:gnd 871:gnd r=0.900001
r1307 754:gnd 851:gnd r=0.0818183
r1306 753:gnd 866:gnd r=0.151364
r1305 753:gnd 755:gnd r=0.178637
r1304 752:gnd 753:gnd r=0.133637
r1303 751:gnd 850:gnd r=0.0818183
r1302 750:gnd 865:gnd r=0.151364
r1301 750:gnd 752:gnd r=0.133637
r1300 749:gnd 750:gnd r=0.133637
r1299 748:gnd 849:gnd r=0.0818183
r1298 747:gnd 756:gnd r=10
r1297 746:gnd 844:gnd r=0.0818183
r1296 745:gnd 746:gnd r=0.0600001
r1295 745:gnd 754:gnd r=0.107727
r1294 742:gnd 749:gnd r=0.586364
r1293 742:gnd 743:gnd r=0.0518182
r1292 741:gnd 752:gnd r=0.586364
r1291 741:gnd 744:gnd r=0.0518182
r1290 741:gnd 745:gnd r=0.133637
r1289 740:gnd 914:gnd r=0.189643
r1288 739:gnd 913:gnd r=0.536786
r1287 739:gnd 909:gnd r=0.308572
r1286 739:gnd 740:gnd r=1.36929
r1285 737:gnd 814:gnd r=0.0540001
r1284 736:gnd 923:gnd r=0.045
r1283 736:gnd 808:gnd r=0.900001
r1282 734:gnd 736:gnd r=0.372273
r1281 734:gnd 880:gnd r=0.263572
r1280 734:gnd 881:gnd r=0.225
r1279 734:gnd 912:gnd r=0.45
r1278 732:gnd 806:gnd r=0.0818183
r1277 730:gnd 732:gnd r=0.0426
r1276 730:gnd 800:gnd r=0.107727
r1275 728:gnd 729:gnd r=0.0391364
r1274 726:gnd 810:gnd r=0.0540001

r1273 724:gnd 738:gnd r=10
r1272 723:gnd 786:gnd r=0.0818183
r1271 722:gnd 921:gnd r=0.178637
r1270 722:gnd 723:gnd r=0.151364
r1269 721:gnd 730:gnd r=0.120955
r1268 721:gnd 731:gnd r=0.0518182
r1267 720:gnd 785:gnd r=0.0818183
r1266 719:gnd 721:gnd r=0.586364
r1265 719:gnd 722:gnd r=0.133637
r1264 718:gnd 719:gnd r=0.133637
r1263 718:gnd 720:gnd r=0.151364
r1262 717:gnd 784:gnd r=0.0818183
r1261 716:gnd 728:gnd r=0.586364
r1260 716:gnd 718:gnd r=0.133637
r1259 714:gnd 776:gnd r=0.0540001
r1258 713:gnd 771:gnd r=0.0540001
r1257 712:gnd 715:gnd r=0.178637
r1256 712:gnd 716:gnd r=0.133637
r1255 712:gnd 717:gnd r=0.151364
r1254 711:gnd 769:gnd r=0.144643
r1253 711:gnd 727:gnd r=0.633215
r1252 710:gnd 874:gnd r=0.0273913
r1251 710:gnd 727:gnd r=0.321429
r1250 709:gnd 743:gnd r=0.0818183
r1249 709:gnd 744:gnd r=0.0818183
r1248 709:gnd 751:gnd r=0.107727
r1247 708:gnd 843:gnd r=0.0600001
r1246 708:gnd 742:gnd r=0.133637
r1245 708:gnd 748:gnd r=0.107727
r1244 707:gnd 925:gnd r=0.178637
r1243 707:gnd 864:gnd r=0.151364
r1242 707:gnd 749:gnd r=0.133637
r1241 706:gnd 867:gnd r=0.0900001
r1240 705:gnd 843:gnd r=0.0818183
r1239 705:gnd 841:gnd r=0.0368182
r1238 c19|minus 835:gnd r=0.900001
r1237 c19|minus 836:gnd r=0.900001
r1236 c19|minus 837:gnd r=0.900001
r1235 c19|minus 838:gnd r=0.900001
r1234 703:gnd 837:gnd r=0.0900001
r1233 703:gnd c19|minus r=0.900001
r1232 702:gnd 835:gnd r=0.0900001
r1231 702:gnd c19|minus r=0.900001

r1230 701:gnd 883:gnd r=0.0469566
r1229 701:gnd 887:gnd r=0.148696
r1228 700:gnd 799:gnd r=0.107727
r1227 700:gnd 731:gnd r=0.059291
r1226 700:gnd 729:gnd r=0.0818183
r1225 699:gnd 728:gnd r=0.116236
r1224 699:gnd 798:gnd r=0.107727
r1223 699:gnd 805:gnd r=0.0600001
r1222 698:gnd 704:gnd r=0.0885247
r1221 697:gnd 760:gnd r=0.0327273
r1220 697:gnd 759:gnd r=0.900001
r1219 696:gnd 803:gnd r=0.0900001
r1218 695:gnd 801:gnd r=0.0900001
r1217 694:gnd 697:gnd r=0.255
r1216 693:gnd 791:gnd r=0.0900001
r1215 692:gnd 778:gnd r=0.0900001
r1214 691:gnd 920:gnd r=0.045
r1213 691:gnd 871:gnd r=0.0375
r1212 690:gnd 904:gnd r=0.686251
r1211 688:gnd 689:gnd r=0.2325
r1210 687:gnd 688:gnd r=0.148125
r1209 686:gnd 766:gnd r=0.049091
r1208 686:gnd 758:gnd r=0.049091
r1207 685:gnd 690:gnd r=0.46875
r1206 685:gnd 691:gnd r=0.900001
r1205 684:gnd 686:gnd r=0.900001
r1204 684:gnd 761:gnd r=0.907501
r1203 682:gnd 725:gnd r=0.051
r1202 682:gnd 726:gnd r=0.129
r1201 682:gnd 804:gnd r=0.129
r1200 m28|src 901:gnd r=10
r1199 m27|src 899:gnd r=10
r1198 m4|src 897:gnd r=10
r1197 m24|src 895:gnd r=10
r1196 m12|src 893:gnd r=10
r1195 m13|src 881:gnd r=10
r1194 m6|src 878:gnd r=10
r1193 m32|src 876:gnd r=10
r1192 m31|src 874:gnd r=10
r1191 680:gnd 919:gnd r=0.045
r1190 680:gnd 683:gnd r=0.045
r1189 680:gnd gnd_1 r=0.1
r1188 679:gnd 681:gnd r=0.0720001

r1187 679:gnd 680:gnd r=0.900001
r1186 678:gnd 903:gnd r=0.2628
r1185 677:gnd 757:gnd r=0.18006
r1184 677:gnd 678:gnd r=0.3763
r1183 676:gnd 677:gnd r=0.303961
r1182 676:gnd 679:gnd r=0.0720001
r1181 675:gnd 906:gnd r=0.368438
r1180 674:gnd 918:gnd r=0.108
r1179 674:gnd 675:gnd r=0.900001
r1178 673:gnd 917:gnd r=0.356786
r1177 673:gnd 747:gnd r=0.297322
r1176 671:gnd 672:gnd r=0.183
r1175 671:gnd 845:gnd r=0.177
r1174 670:gnd 855:gnd r=0.138
r1173 670:gnd 846:gnd r=0.0870001
r1172 669:gnd 671:gnd r=0.138
r1171 669:gnd 670:gnd r=0.486
r1170 668:gnd 859:gnd r=0.177
r1169 668:gnd 669:gnd r=0.138
r1168 667:gnd 862:gnd r=0.0870001
r1167 667:gnd 855:gnd r=0.138
r1166 665:gnd 706:gnd r=0.177
r1165 665:gnd 666:gnd r=0.183
r1164 664:gnd 668:gnd r=0.138
r1163 664:gnd 667:gnd r=0.486
r1162 664:gnd 665:gnd r=0.138
r1161 663:gnd 756:gnd r=10
r1160 663:gnd 678:gnd r=0.0954001
r1159 662:gnd 756:gnd r=10
r1158 662:gnd 663:gnd r=0.1008
r1157 661:gnd 756:gnd r=10
r1156 661:gnd 662:gnd r=0.1008
r1155 660:gnd 756:gnd r=10
r1154 660:gnd 661:gnd r=0.1026
r1153 659:gnd 756:gnd r=10
r1152 659:gnd 747:gnd r=0.118254
r1151 658:gnd 756:gnd r=10
r1150 658:gnd 660:gnd r=0.1044
r1149 658:gnd 659:gnd r=0.1008
r1148 656:gnd 657:gnd r=0.183
r1147 656:gnd 702:gnd r=0.177
r1146 655:gnd 832:gnd r=0.0900001
r1145 655:gnd 833:gnd r=0.0900001

r1144 653:gnd 703:gnd r=0.177
r1143 653:gnd 654:gnd r=0.183
r1142 652:gnd 656:gnd r=0.138
r1141 652:gnd 655:gnd r=0.486
r1140 652:gnd 653:gnd r=0.138
r1139 650:gnd 651:gnd r=0.183
r1138 650:gnd 818:gnd r=0.177
r1137 649:gnd 821:gnd r=0.138
r1136 649:gnd 820:gnd r=0.0870001
r1135 648:gnd 650:gnd r=0.138
r1134 648:gnd 649:gnd r=0.486
r1133 647:gnd 822:gnd r=0.177
r1132 647:gnd 648:gnd r=0.138
r1131 646:gnd 821:gnd r=0.138
r1130 646:gnd 815:gnd r=0.0870001
r1129 644:gnd 816:gnd r=0.177
r1128 644:gnd 645:gnd r=0.183
r1127 643:gnd 647:gnd r=0.138
r1126 643:gnd 646:gnd r=0.486
r1125 643:gnd 644:gnd r=0.138
r1124 642:gnd 892:gnd r=0.0469566
r1123 641:gnd 910:gnd r=0.0720001
r1122 641:gnd 811:gnd r=0.900001
r1121 640:gnd 641:gnd r=0.861614
r1120 640:gnd 886:gnd r=0.058187
r1119 639:gnd 869:gnd r=10
r1118 639:gnd 640:gnd r=0.123202
r1117 638:gnd 869:gnd r=10
r1116 638:gnd 642:gnd r=0.111954
r1115 638:gnd 639:gnd r=0.101739
r1114 637:gnd gnd_1 r=0.104323
r1113 636:gnd 733:gnd r=0.051
r1112 636:gnd 714:gnd r=0.129
r1111 636:gnd 781:gnd r=0.129
r1110 634:gnd 635:gnd r=0.183
r1109 634:gnd 777:gnd r=0.177
r1108 633:gnd 782:gnd r=0.138
r1107 633:gnd 733:gnd r=0.0870001
r1106 632:gnd 634:gnd r=0.138
r1105 632:gnd 633:gnd r=0.486
r1104 631:gnd 787:gnd r=0.177
r1103 631:gnd 632:gnd r=0.138
r1102 630:gnd 782:gnd r=0.138

r1101 630:gnd 725:gnd r=0.0870001
r1100 628:gnd 696:gnd r=0.177
r1099 628:gnd 629:gnd r=0.183
r1098 627:gnd 631:gnd r=0.138
r1097 627:gnd 630:gnd r=0.486
r1096 627:gnd 628:gnd r=0.138
r1095 625:gnd 626:gnd r=0.183
r1094 625:gnd 692:gnd r=0.177
r1093 624:gnd 775:gnd r=0.0870001
r1092 624:gnd 773:gnd r=0.138
r1091 623:gnd 625:gnd r=0.138
r1090 623:gnd 624:gnd r=0.486
r1089 622:gnd 693:gnd r=0.177
r1088 622:gnd 623:gnd r=0.138
r1087 621:gnd 774:gnd r=0.0870001
r1086 621:gnd 773:gnd r=0.138
r1085 619:gnd 695:gnd r=0.177
r1084 619:gnd 620:gnd r=0.183
r1083 618:gnd 622:gnd r=0.138
r1082 618:gnd 621:gnd r=0.486
r1081 618:gnd 619:gnd r=0.138
r1080 617:gnd 738:gnd r=10
r1079 617:gnd 724:gnd r=0.108263
r1078 616:gnd 738:gnd r=10
r1077 616:gnd 617:gnd r=0.1008
r1076 615:gnd 738:gnd r=10
r1075 615:gnd 616:gnd r=0.1026
r1074 614:gnd 738:gnd r=10
r1073 614:gnd 615:gnd r=0.1044
r1072 613:gnd 738:gnd r=10
r1071 613:gnd 727:gnd r=0.0954001
r1070 612:gnd 738:gnd r=10
r1069 612:gnd 614:gnd r=0.1008
r1068 612:gnd 613:gnd r=0.1008
r1067 611:gnd 724:gnd r=0.166535
r1066 610:gnd 611:gnd r=0.0929033
r1065 610:gnd 908:gnd r=0.347143
r1064 610:gnd 735:gnd r=0.048
r1063 610:gnd 812:gnd r=0.900001
r1062 609:gnd 762:gnd r=0.0600001
r1061 608:gnd 689:gnd r=0.2325
r1060 608:gnd 609:gnd r=0.34125
r1059 607:gnd 868:gnd r=0.129

r1058 607:gnd 860:gnd r=0.129
r1057 607:gnd 862:gnd r=0.051
r1056 606:gnd 846:gnd r=0.051
r1055 606:gnd 848:gnd r=0.129
r1054 606:gnd 834:gnd r=0.129
r1053 605:gnd 831:gnd r=0.132
r1052 605:gnd 833:gnd r=0.048
r1051 605:gnd 838:gnd r=0.129
r1050 604:gnd 836:gnd r=0.129
r1049 604:gnd 832:gnd r=0.048
r1048 604:gnd 819:gnd r=0.132
r1047 603:gnd 828:gnd r=0.129
r1046 603:gnd 817:gnd r=0.129
r1045 603:gnd 815:gnd r=0.051
r1044 602:gnd 737:gnd r=0.129
r1043 602:gnd 824:gnd r=0.129
r1042 602:gnd 820:gnd r=0.051
r1041 601:gnd 772:gnd r=0.129
r1040 601:gnd 802:gnd r=0.129
r1039 601:gnd 774:gnd r=0.051
r1038 600:gnd 713:gnd r=0.129
r1037 600:gnd 779:gnd r=0.129
r1036 600:gnd 775:gnd r=0.051
r1035 599:gnd 916:gnd r=0.572143
r1034 599:gnd 673:gnd r=0.810001
r1033 599:gnd 674:gnd r=0.196072
r1032 c21|minus 843:gnd r=0.900001
r1031 c21|minus 864:gnd r=0.900001
r1030 c21|minus 865:gnd r=0.900001
r1029 c21|minus 866:gnd r=0.900001
r1028 c21|minus 743:gnd r=0.900001
r1027 c21|minus 744:gnd r=0.900001
r1026 c21|minus 708:gnd r=0.900001
r1025 c21|minus 742:gnd r=0.900001
r1024 c21|minus 709:gnd r=0.900001
r1023 c21|minus 741:gnd r=0.900001
r1022 c21|minus 745:gnd r=0.900001
r1021 c21|minus 746:gnd r=0.900001
r1020 c21|minus 844:gnd r=0.900001
r1019 c21|minus 849:gnd r=0.900001
r1018 c21|minus 850:gnd r=0.900001
r1017 c21|minus 851:gnd r=0.900001
r1016 c21|minus 842:gnd r=0.2715

r1015 c21|minus 856:gnd r=0.900001
r1014 c21|minus 857:gnd r=0.900001
r1013 c21|minus 858:gnd r=0.900001
r1012 c21|minus 687:gnd r=0.900001
r1011 c21|minus 748:gnd r=0.900001
r1010 c21|minus 852:gnd r=0.900001
r1009 c21|minus 751:gnd r=0.900001
r1008 c21|minus 853:gnd r=0.900001
r1007 c21|minus 754:gnd r=0.900001
r1006 c21|minus 854:gnd r=0.900001
r1005 c21|minus 705:gnd r=0.900001
r1004 c1|minus 732:gnd r=0.900001
r1003 c1|minus 806:gnd r=0.900001
r1002 c1|minus 699:gnd r=0.900001
r1001 c1|minus 728:gnd r=0.900001
r1000 c1|minus 694:gnd r=0.228055
r999 c1|minus 808:gnd r=0.0739637
r998 c1|minus 700:gnd r=0.900001
r997 c1|minus 798:gnd r=0.900001
r996 c1|minus 721:gnd r=0.900001
r995 c1|minus 799:gnd r=0.900001
r994 c1|minus 730:gnd r=0.900001
r993 c1|minus 800:gnd r=0.900001
r992 c1|minus 717:gnd r=0.900001
r991 c1|minus 788:gnd r=0.900001
r990 c1|minus 720:gnd r=0.900001
r989 c1|minus 789:gnd r=0.900001
r988 c1|minus 723:gnd r=0.900001
r987 c1|minus 790:gnd r=0.900001
r986 c1|minus 783:gnd r=0.900001
r985 c1|minus 805:gnd r=0.900001
r984 c1|minus 784:gnd r=0.900001
r983 c1|minus 795:gnd r=0.900001
r982 c1|minus 785:gnd r=0.900001
r981 c1|minus 796:gnd r=0.900001
r980 c1|minus 786:gnd r=0.900001
r979 c1|minus 797:gnd r=0.900001
r978 c1|minus 729:gnd r=0.900001
r977 c1|minus 731:gnd r=0.900001
r976 c18|minus 649:gnd r=0.900001
r975 c18|minus 762:gnd r=0.900001
r974 c18|minus 833:gnd r=0.900001
r973 c18|minus 706:gnd r=0.900001

r972 c18|minus 604:gnd r=0.900001
r971 c18|minus 867:gnd r=0.900001
r970 c18|minus 868:gnd r=0.900001
r969 c18|minus 605:gnd r=0.900001
r968 c18|minus 855:gnd r=0.45
r967 c18|minus 655:gnd r=0.900001
r966 c18|minus 815:gnd r=0.900001
r965 c18|minus 816:gnd r=0.900001
r964 c18|minus 859:gnd r=0.900001
r963 c18|minus 827:gnd r=0.900001
r962 c18|minus 861:gnd r=0.900001
r961 c18|minus 828:gnd r=0.900001
r960 c18|minus 863:gnd r=0.900001
r959 c18|minus 860:gnd r=0.900001
r958 c18|minus 845:gnd r=0.900001
r957 c18|minus 847:gnd r=0.900001
r956 c18|minus 848:gnd r=0.900001
r955 c18|minus 817:gnd r=0.900001
r954 c18|minus 606:gnd r=0.900001
r953 c18|minus 831:gnd r=0.900001
r952 c18|minus 737:gnd r=0.900001
r951 c18|minus 607:gnd r=0.900001
r950 c18|minus 667:gnd r=0.900001
r949 c18|minus 670:gnd r=0.900001
r948 c18|minus c19|minus r=0.001
r947 c18|minus 818:gnd r=0.900001
r946 c18|minus 823:gnd r=0.900001
r945 c18|minus 846:gnd r=0.900001
r944 c18|minus 824:gnd r=0.900001
r943 c18|minus 819:gnd r=0.900001
r942 c18|minus 820:gnd r=0.900001
r941 c18|minus 834:gnd r=0.900001
r940 c18|minus 862:gnd r=0.900001
r939 c18|minus 821:gnd r=0.45
r938 c18|minus 822:gnd r=0.900001
r937 c18|minus 832:gnd r=0.900001
r936 c18|minus 825:gnd r=0.900001
r935 c18|minus 698:gnd r=0.1455
r934 c18|minus 826:gnd r=0.900001
r933 c18|minus 602:gnd r=0.900001
r932 c18|minus 603:gnd r=0.900001
r931 c18|minus 646:gnd r=0.900001
r930 c8|minus 787:gnd r=0.900001

r929 c8|minus 636:gnd r=0.900001
r928 c8|minus 733:gnd r=0.900001
r927 c8|minus 725:gnd r=0.900001
r926 c8|minus 793:gnd r=0.900001
r925 c8|minus 794:gnd r=0.900001
r924 c8|minus 682:gnd r=0.900001
r923 c8|minus 630:gnd r=0.900001
r922 c8|minus 633:gnd r=0.900001
r921 c8|minus 726:gnd r=0.900001
r920 c8|minus 714:gnd r=0.900001
r919 c8|minus 704:gnd r=0.138
r918 c8|minus 696:gnd r=0.900001
r917 c8|minus 803:gnd r=0.900001
r916 c8|minus 804:gnd r=0.900001
r915 c8|minus 782:gnd r=0.45
r914 c8|minus 637:gnd r=0.131016
r913 c8|minus 777:gnd r=0.900001
r912 c8|minus 780:gnd r=0.900001
r911 c8|minus 781:gnd r=0.900001
r910 c16|minus 772:gnd r=0.900001
r909 c16|minus 713:gnd r=0.900001
r908 c16|minus 695:gnd r=0.900001
r907 c16|minus 801:gnd r=0.900001
r906 c16|minus 802:gnd r=0.900001
r905 c16|minus 773:gnd r=0.45
r904 c16|minus 600:gnd r=0.900001
r903 c16|minus 637:gnd r=0.714601
r902 c16|minus 692:gnd r=0.900001
r901 c16|minus 601:gnd r=0.900001
r900 c16|minus 778:gnd r=0.900001
r899 c16|minus 621:gnd r=0.900001
r898 c16|minus 624:gnd r=0.900001
r897 c16|minus 779:gnd r=0.900001
r896 c16|minus 811:gnd r=0.012
r895 c16|minus 693:gnd r=0.900001
r894 c16|minus 791:gnd r=0.900001
r893 c16|minus 775:gnd r=0.900001
r892 c16|minus 774:gnd r=0.900001
r891 c16|minus 792:gnd r=0.900001
r890 c17|minus c18|minus r=0.001
r889 592:vsec_storage_rf 598:vsec_storage_rf r=0.0739286
r888 591:vsec_storage_rf 596:vsec_storage_rf r=0.311786
r887 591:vsec_storage_rf 592:vsec_storage_rf r=0.343929

r886 589:vsec_storage_rf 590:vsec_storage_rf r=0.0739286
r885 586:vsec_storage_rf 588:vsec_storage_rf r=0.045
r884 586:vsec_storage_rf 587:vsec_storage_rf r=0.0990001
r883 585:vsec_storage_rf 589:vsec_storage_rf r=0.315
r882 584:vsec_storage_rf 595:vsec_storage_rf r=0.263572
r881 583:vsec_storage_rf 586:vsec_storage_rf r=10
r880 583:vsec_storage_rf 587:vsec_storage_rf r=10
r879 582:vsec_storage_rf 597:vsec_storage_rf r=0.0739286
r878 582:vsec_storage_rf 595:vsec_storage_rf r=0.151072
r877 580:vsec_storage_rf 588:vsec_storage_rf r=0.331072
r876 579:vsec_storage_rf 584:vsec_storage_rf r=0.707144
r875 579:vsec_storage_rf 585:vsec_storage_rf r=0.379286
r874 579:vsec_storage_rf 580:vsec_storage_rf r=0.691072
r873 578:vsec_storage_rf 594:vsec_storage_rf r=0.732858
r872 578:vsec_storage_rf 596:vsec_storage_rf r=0.0720001
r871 577:vsec_storage_rf 587:vsec_storage_rf r=0.045
r870 577:vsec_storage_rf 578:vsec_storage_rf r=1.19269
r869 576:vsec_storage_rf 594:vsec_storage_rf r=0.0720001
r868 576:vsec_storage_rf 581:vsec_storage_rf r=0.0720001
r867 575:vsec_storage_rf 576:vsec_storage_rf r=0.900001
r866 m25|src 585:vsec_storage_rf r=10
r865 m25|src 589:vsec_storage_rf r=10
r864 m20|src 584:vsec_storage_rf r=10
r863 m26|src 591:vsec_storage_rf r=10
r862 m26|src 592:vsec_storage_rf r=10
r861 m20|gate 582:vsec_storage_rf r=8
r860 c21|plus 575:vsec_storage_rf r=0.0695455
r859 c21|plus 593:vsec_storage_rf r=0.452728
r858 c21|plus vsec_storage_rf r=0.452728
r857 569:rf_inn 572:rf_inn r=0.0155173
r856 569:rf_inn 571:rf_inn r=0.170357
r855 566:rf_inn 567:rf_inn r=0.666563
r854 565:rf_inn 566:rf_inn r=0.506251
r853 rf_inn 567:rf_inn r=0.0689063
r852 563:rf_inn 564:rf_inn r=0.0739286
r851 561:rf_inn 568:rf_inn r=0.109286
r850 561:rf_inn 562:rf_inn r=0.0739286
r849 560:rf_inn 571:rf_inn r=0.347143
r848 559:rf_inn 574:rf_inn r=0.0739286
r847 559:rf_inn 560:rf_inn r=0.980358
r846 558:rf_inn 565:rf_inn r=0.151875
r845 557:rf_inn 558:rf_inn r=0.194063
r844 554:rf_inn 555:rf_inn r=0.0675001

r843 553:rf_inn 570:rf_inn r=0.135
r842 552:rf_inn 568:rf_inn r=0.028125
r841 552:rf_inn 556:rf_inn r=0.900001
r840 551:rf_inn 552:rf_inn r=0.028125
r839 551:rf_inn 553:rf_inn r=0.138214
r838 550:rf_inn 573:rf_inn r=0.0720001
r837 550:rf_inn 554:rf_inn r=0.900001
r836 549:rf_inn 554:rf_inn r=0.568126
r835 549:rf_inn 556:rf_inn r=0.253125
r834 m25|drn 561:rf_inn r=10
r833 m25|drn 553:rf_inn r=10
r832 m27|drn 563:rf_inn r=10
r831 m26|gate 560:rf_inn r=8
r830 m28|gate 559:rf_inn r=8
r829 548:rf_inn 557:rf_inn r=0.900001
r828 548:rf_inn 572:rf_inn r=0.0900001
r827 548:rf_inn 570:rf_inn r=0.556072
r826 547:rf_inn 563:rf_inn r=0.0803572
r825 547:rf_inn 550:rf_inn r=0.0720001
r824 544:ln_n127 545:ln_n127 r=0.333075
r823 ln_n127 544:ln_n127 r=2.25553
r822 ln_n127 546:ln_n127 r=0.001
r821 541:ln_n125 542:ln_n125 r=0.108
r820 540:ln_n125 541:ln_n125 r=0.345
r819 ln_n125 540:ln_n125 r=1.19438
r818 ln_n125 543:ln_n125 r=0.001
r817 538:cont_n1 cont_n1 r=0.0739286
r816 537:cont_n1 539:cont_n1 r=0.385715
r815 537:cont_n1 538:cont_n1 r=0.295715
r814 535:cont_n1 536:cont_n1 r=0.0739286
r813 533:cont_n1 534:cont_n1 r=0.151072
r812 533:cont_n1 535:cont_n1 r=0.263572
r811 532:cont_n1 539:cont_n1 r=0.186429
r810 532:cont_n1 534:cont_n1 r=0.630001
r809 m13|gate 534:cont_n1 r=8
r808 m13|drn 535:cont_n1 r=10
r807 m14|src 537:cont_n1 r=10
r806 m14|src 538:cont_n1 r=10
r805 m6|gate 532:cont_n1 r=8
r804 529:cont_n2 531:cont_n2 r=0.838929
r803 529:cont_n2 cont_n2 r=0.0739286
r802 527:cont_n2 528:cont_n2 r=0.0739286
r801 525:cont_n2 530:cont_n2 r=0.151072

r800 525:cont_n2 526:cont_n2 r=0.0739286
r799 523:cont_n2 524:cont_n2 r=0.151072
r798 523:cont_n2 527:cont_n2 r=0.263572
r797 523:cont_n2 530:cont_n2 r=0.501429
r796 522:cont_n2 524:cont_n2 r=0.658929
r795 521:cont_n2 522:cont_n2 r=0.215357
r794 521:cont_n2 531:cont_n2 r=0.376072
r793 m10|drn 529:cont_n2 r=10
r792 m12|drn 527:cont_n2 r=10
r791 m24|gate 525:cont_n2 r=8
r790 m12|gate 524:cont_n2 r=8
r789 m14|gate 522:cont_n2 r=8
r788 518:load1 519:load1 r=1.57822
r787 517:load1 518:load1 r=0.694286
r786 515:load1 516:load1 r=0.0739286
r785 513:load1 514:load1 r=0.0739286
r784 512:load1 515:load1 r=0.295715
r783 511:load1 513:load1 r=0.295715
r782 509:load1 512:load1 r=0.363215
r781 508:load1 511:load1 r=0.363215
r780 508:load1 509:load1 r=0.604286
r779 508:load1 519:load1 r=0.417858
r778 507:load1 510:load1 r=0.0720001
r777 506:load1 507:load1 r=0.0720001
r776 506:load1 517:load1 r=0.237857
r775 m21@2|drn 515:load1 r=10
r774 m21@2|drn 512:load1 r=10
r773 load1 520:load1 r=0.045
r772 load1 507:load1 r=0.900001
r771 load1 505:load1 r=0.045
r770 m21|drn 511:load1 r=10
r769 m21|drn 513:load1 r=10
r768 500:load2 503:load2 r=0.324643
r767 497:load2 500:load2 r=0.295715
r766 497:load2 498:load2 r=0.0739286
r765 495:load2 499:load2 r=0.295715
r764 495:load2 496:load2 r=0.0739286
r763 493:load2 502:load2 r=1.56
r762 493:load2 501:load2 r=0.189
r761 492:load2 499:load2 r=0.318215
r760 492:load2 502:load2 r=0.207
r759 492:load2 503:load2 r=0.604286
r758 load2 504:load2 r=0.0562501

r757 491:load2 501:load2 r=0.028125
r756 491:load2 494:load2 r=0.028125
r755 491:load2 load2 r=0.900001
r754 m22@2|drn 500:load2 r=10
r753 m22@2|drn 497:load2 r=10
r752 m22|drn 495:load2 r=10
r751 m22|drn 499:load2 r=10
r750 486:vreg vreg r=0.0717188
r749 484:vreg 485:vreg r=0.101739
r748 483:vreg 484:vreg r=0.101739
r747 482:vreg 483:vreg r=0.101739
r746 481:vreg 482:vreg r=0.101739
r745 480:vreg 481:vreg r=0.146739
r744 479:vreg 487:vreg r=0.27
r743 478:vreg 488:vreg r=0.0739286
r742 478:vreg 479:vreg r=0.324643
r741 476:vreg 477:vreg r=0.101739
r740 475:vreg 476:vreg r=0.101739
r739 473:vreg 474:vreg r=0.101739
r738 472:vreg 473:vreg r=0.101739
r737 471:vreg 472:vreg r=0.101739
r736 470:vreg 475:vreg r=0.101739
r735 469:vreg 471:vreg r=0.101739
r734 468:vreg 480:vreg r=0.146739
r733 467:vreg 471:vreg r=10
r732 467:vreg 472:vreg r=10
r731 467:vreg 473:vreg r=10
r730 467:vreg 474:vreg r=10
r729 467:vreg 477:vreg r=10
r728 467:vreg 470:vreg r=10
r727 467:vreg 468:vreg r=10
r726 467:vreg 475:vreg r=10
r725 467:vreg 480:vreg r=10
r724 467:vreg 481:vreg r=10
r723 467:vreg 482:vreg r=10
r722 467:vreg 476:vreg r=10
r721 467:vreg 483:vreg r=10
r720 467:vreg 484:vreg r=10
r719 467:vreg 485:vreg r=10
r718 467:vreg 469:vreg r=10
r717 466:vreg 486:vreg r=0.288
r716 462:vreg 464:vreg r=0.144
r715 460:vreg 463:vreg r=0.0900001

r714 459:vreg 462:vreg r=0.0900001
r713 458:vreg 461:vreg r=0.0900001
r712 457:vreg 460:vreg r=0.0900001
r711 456:vreg 459:vreg r=0.0900001
r710 455:vreg 458:vreg r=0.0900001
r709 454:vreg 457:vreg r=0.0900001
r708 453:vreg 456:vreg r=0.0900001
r707 452:vreg 455:vreg r=0.0900001
r706 451:vreg 454:vreg r=0.0900001
r705 450:vreg 453:vreg r=0.0900001
r704 449:vreg 452:vreg r=0.0900001
r703 447:vreg 465:vreg r=0.0540001
r702 445:vreg 490:vreg r=0.0540001
r701 444:vreg 451:vreg r=0.0900001
r700 443:vreg 464:vreg r=0.138
r699 443:vreg 448:vreg r=0.0643801
r698 442:vreg 489:vreg r=0.183
r697 442:vreg 444:vreg r=0.192
r696 441:vreg 450:vreg r=0.0900001
r695 440:vreg 443:vreg r=0.786001
r694 440:vreg 442:vreg r=0.138
r693 439:vreg 464:vreg r=0.11538
r692 439:vreg 446:vreg r=0.0870001
r691 438:vreg 441:vreg r=0.192
r690 438:vreg 440:vreg r=0.138
r689 436:vreg 449:vreg r=0.0900001
r688 435:vreg 439:vreg r=0.786001
r687 435:vreg 438:vreg r=0.138
r686 434:vreg 477:vreg r=0.0469566
r685 434:vreg 487:vreg r=1.70357
r684 434:vreg 468:vreg r=0.0547827
r683 433:vreg 470:vreg r=0.146739
r682 433:vreg 467:vreg r=10
r681 433:vreg 469:vreg r=0.146739
r680 432:vreg 466:vreg r=0.900001
r679 431:vreg 447:vreg r=0.12324
r678 431:vreg 463:vreg r=0.144
r677 431:vreg 448:vreg r=0.051
r676 430:vreg 461:vreg r=0.144
r675 430:vreg 446:vreg r=0.04524
r674 430:vreg 445:vreg r=0.129
r673 429:vreg 435:vreg r=0.138
r672 429:vreg 436:vreg r=0.192

r671 429:vreg 437:vreg r=0.183
r670 m9|drn 478:vreg r=10
r669 m9|drn 479:vreg r=10
r668 428:vreg 432:vreg r=1.48176
r667 428:vreg 485:vreg r=0.146739
r666 428:vreg 467:vreg r=10
r665 427:vreg 467:vreg r=10
r664 427:vreg 428:vreg r=0.124379
r663 426:vreg 467:vreg r=10
r662 426:vreg 427:vreg r=0.101739
r661 425:vreg 467:vreg r=10
r660 425:vreg 426:vreg r=0.101739
r659 424:vreg 467:vreg r=10
r658 424:vreg 425:vreg r=0.101739
r657 423:vreg 474:vreg r=0.146739
r656 423:vreg 467:vreg r=10
r655 422:vreg 467:vreg r=10
r654 422:vreg 424:vreg r=0.101739
r653 422:vreg 423:vreg r=0.124379
r652 c15|minus 447:vreg r=0.900001
r651 c15|minus 449:vreg r=0.900001
r650 c15|minus 455:vreg r=0.900001
r649 c15|minus 466:vreg r=0.14574
r648 c15|minus 450:vreg r=0.900001
r647 c15|minus 456:vreg r=0.900001
r646 c15|minus 451:vreg r=0.900001
r645 c15|minus 457:vreg r=0.900001
r644 c15|minus 430:vreg r=0.900001
r643 c15|minus 461:vreg r=0.900001
r642 c15|minus 439:vreg r=0.900001
r641 c15|minus 462:vreg r=0.900001
r640 c15|minus 463:vreg r=0.900001
r639 c15|minus 443:vreg r=0.900001
r638 c15|minus 431:vreg r=0.900001
r637 c15|minus 436:vreg r=0.900001
r636 c15|minus 441:vreg r=0.900001
r635 c15|minus 444:vreg r=0.900001
r634 c15|minus 452:vreg r=0.900001
r633 c15|minus 445:vreg r=0.900001
r632 c15|minus 453:vreg r=0.900001
r631 c15|minus 454:vreg r=0.900001
r630 c15|minus 446:vreg r=0.900001
r629 c15|minus 458:vreg r=0.900001

r628 c15|minus 464:vreg r=0.45
r627 c15|minus 459:vreg r=0.900001
r626 c15|minus 460:vreg r=0.900001
r625 c15|minus 448:vreg r=0.900001
r624 ln_n43 421:ln_n43 r=0.0600001
r623 419:ln_n43 ln_n43 r=0.0900001
r622 418:ln_n43 420:ln_n43 r=0.0600001
r621 418:ln_n43 419:ln_n43 r=0.0900001
r620 417:ln_n43 418:ln_n43 r=0.900001
r619 417:ln_n43 419:ln_n43 r=0.900001
r618 417:ln_n43 ln_n43 r=0.900001
r617 ln_n41 416:ln_n41 r=0.0540001
r616 415:ln_n41 ln_n41 r=0.0900001
r615 414:ln_n41 415:ln_n41 r=0.0900001
r614 413:ln_n41 414:ln_n41 r=0.0900001
r613 412:ln_n41 413:ln_n41 r=0.0900001
r612 411:ln_n41 412:ln_n41 r=0.0900001
r611 410:ln_n41 411:ln_n41 r=0.0900001
r610 409:ln_n41 410:ln_n41 r=0.0900001
r609 407:ln_n41 409:ln_n41 r=0.0900001
r608 406:ln_n41 408:ln_n41 r=0.0540001
r607 406:ln_n41 407:ln_n41 r=0.0900001
r606 405:ln_n41 409:ln_n41 r=0.900001
r605 405:ln_n41 414:ln_n41 r=0.900001
r604 405:ln_n41 412:ln_n41 r=0.900001
r603 405:ln_n41 410:ln_n41 r=0.900001
r602 405:ln_n41 ln_n41 r=0.900001
r601 405:ln_n41 406:ln_n41 r=0.900001
r600 405:ln_n41 407:ln_n41 r=0.900001
r599 405:ln_n41 411:ln_n41 r=0.900001
r598 405:ln_n41 413:ln_n41 r=0.900001
r597 405:ln_n41 415:ln_n41 r=0.900001
r596 ln_n35 404:ln_n35 r=0.0600001
r595 402:ln_n35 ln_n35 r=0.0900001
r594 401:ln_n35 403:ln_n35 r=0.0600001
r593 401:ln_n35 402:ln_n35 r=0.0900001
r592 400:ln_n35 401:ln_n35 r=0.900001
r591 400:ln_n35 402:ln_n35 r=0.900001
r590 400:ln_n35 ln_n35 r=0.900001
r589 397:load3_cont 398:load3_cont r=0.282857
r588 396:load3_cont 399:load3_cont r=0.0739286
r587 396:load3_cont 397:load3_cont r=0.218572
r586 393:load3_cont 398:load3_cont r=0.0720001

r585 393:load3_cont 394:load3_cont r=0.0720001
r584 392:load3_cont 395:load3_cont r=0.0720001
r583 392:load3_cont 393:load3_cont r=0.900001
r582 389:load3_cont load3_cont r=0.0253125
r581 389:load3_cont 390:load3_cont r=0.045
r580 388:load3_cont 389:load3_cont r=0.900001
r579 387:load3_cont 392:load3_cont r=0.170965
r578 387:load3_cont 391:load3_cont r=0.900001
r577 386:load3_cont 387:load3_cont r=0.0675001
r576 385:load3_cont 391:load3_cont r=0.258075
r575 m23@2|gate 396:load3_cont r=8
r574 m23@2|gate 397:load3_cont r=8
r573 m23|gate m23@2|gate r=0.001
r572 384:load3_cont 388:load3_cont r=0.25125
r571 383:load3_cont 384:load3_cont r=0.2325
r570 382:load3_cont 385:load3_cont r=0.47126
r569 381:load3_cont 383:load3_cont r=0.2325
r568 381:load3_cont 382:load3_cont r=0.174375
r567 376:load2_cont 377:load2_cont r=0.0739286
r566 375:load2_cont 378:load2_cont r=0.115714
r565 375:load2_cont 376:load2_cont r=0.218572
r564 load2_cont 379:load2_cont r=0.028125
r563 374:load2_cont 380:load2_cont r=0.0720001
r562 374:load2_cont load2_cont r=0.900001
r561 373:load2_cont 378:load2_cont r=0.726429
r560 373:load2_cont 374:load2_cont r=0.0720001
r559 m22@2|gate 375:load2_cont r=8
r558 m22@2|gate 376:load2_cont r=8
r557 m22|gate m22@2|gate r=0.001
r556 370:load1_cont 371:load1_cont r=2.82215
r555 368:load1_cont 370:load1_cont r=0.398572
r554 load1_cont 372:load1_cont r=0.0492188
r553 366:load1_cont 367:load1_cont r=0.0720001
r552 366:load1_cont load1_cont r=0.900001
r551 365:load1_cont 371:load1_cont r=0.0803572
r550 365:load1_cont 366:load1_cont r=0.0720001
r549 364:load1_cont 369:load1_cont r=0.0739286
r548 364:load1_cont 368:load1_cont r=0.218572
r547 m21@2|gate 364:load1_cont r=8
r546 m21@2|gate 368:load1_cont r=8
r545 m21|gate m21@2|gate r=0.001
r544 360:vref3 361:vref3 r=1.05107
r543 m2|drn m1|src r=0.001

r542 358:vref3 363:vref3 r=0.0739286
r541 358:vref3 m2|drn r=10
r540 357:vref3 m2|drn r=10
r539 357:vref3 358:vref3 r=0.167143
r538 356:vref3 357:vref3 r=0.244286
r537 355:vref3 360:vref3 r=0.170357
r536 355:vref3 356:vref3 r=0.151072
r535 354:vref3 361:vref3 r=0.124138
r534 353:vref3 362:vref3 r=0.0720001
r533 353:vref3 354:vref3 r=0.0720001
r532 vref3 359:vref3 r=0.03375
r531 vref3 353:vref3 r=0.900001
r530 m2|gate 355:vref3 r=8
r529 348:vref1 349:vref1 r=0.0739286
r528 347:vref1 348:vref1 r=0.295715
r527 346:vref1 352:vref1 r=1.09286
r526 345:vref1 346:vref1 r=0.295715
r525 343:vref1 344:vref1 r=0.0739286
r524 341:vref1 350:vref1 r=0.636429
r523 340:vref1 342:vref1 r=0.0739286
r522 340:vref1 341:vref1 r=0.218572
r521 337:vref1 339:vref1 r=0.0720001
r520 337:vref1 338:vref1 r=0.0720001
r519 336:vref1 351:vref1 r=0.045
r518 336:vref1 337:vref1 r=0.900001
r517 335:vref1 338:vref1 r=0.954644
r516 334:vref1 352:vref1 r=0.237857
r515 334:vref1 347:vref1 r=0.2925
r514 334:vref1 350:vref1 r=1.62
r513 333:vref1 343:vref1 r=0.151072
r512 333:vref1 335:vref1 r=0.170357
r511 333:vref1 345:vref1 r=0.263572
r510 vref1 336:vref1 r=0.0464063
r509 vref1 332:vref1 r=0.0773438
r508 m5|drn 347:vref1 r=10
r507 m5|drn m5@2|drn r=0.001
r506 m5|drn 348:vref1 r=10
r505 m0|drn 346:vref1 r=10
r504 m0|drn 345:vref1 r=10
r503 m0|gate 343:vref1 r=8
r502 m15|gate m15@2|gate r=0.001
r501 m15|gate 340:vref1 r=8
r500 m15|gate 341:vref1 r=8

r499 328:piezo_inn 330:piezo_inn r=0.0739286
r498 325:piezo_inn 327:piezo_inn r=0.343929
r497 325:piezo_inn 326:piezo_inn r=0.0739286
r496 322:piezo_inn 324:piezo_inn r=0.414643
r495 322:piezo_inn 323:piezo_inn r=0.0739286
r494 321:piezo_inn 331:piezo_inn r=0.0739286
r493 321:piezo_inn 329:piezo_inn r=0.440358
r492 318:piezo_inn 320:piezo_inn r=0.0703126
r491 316:piezo_inn 319:piezo_inn r=0.0720001
r490 316:piezo_inn 318:piezo_inn r=0.900001
r489 315:piezo_inn 319:piezo_inn r=0.215357
r488 315:piezo_inn 328:piezo_inn r=0.343929
r487 314:piezo_inn 315:piezo_inn r=0.6506
r486 313:piezo_inn 318:piezo_inn r=0.531563
r485 312:piezo_inn 327:piezo_inn r=0.260357
r484 312:piezo_inn 316:piezo_inn r=0.0720001
r483 311:piezo_inn 314:piezo_inn r=0.0316184
r482 piezo_inn 317:piezo_inn r=0.0421875
r481 piezo_inn 314:piezo_inn r=0.900001
r480 piezo_inn 310:piezo_inn r=0.0478125
r479 m29|drn 328:piezo_inn r=10
r478 m29|drn 315:piezo_inn r=10
r477 m31|drn 325:piezo_inn r=10
r476 m30|gate 322:piezo_inn r=8
r475 m32|gate 321:piezo_inn r=8
r474 309:piezo_inn 313:piezo_inn r=0.523126
r473 308:piezo_inn 329:piezo_inn r=0.028125
r472 308:piezo_inn 324:piezo_inn r=0.028125
r471 308:piezo_inn 309:piezo_inn r=0.900001
r470 303:piezo_inp 304:piezo_inp r=0.0739286
r469 301:piezo_inp 305:piezo_inp r=0.138214
r468 301:piezo_inp 302:piezo_inp r=0.1575
r467 298:piezo_inp 300:piezo_inp r=0.0739286
r466 298:piezo_inp 299:piezo_inp r=0.0803572
r465 292:piezo_inp 299:piezo_inp r=0.0720001
r464 292:piezo_inp 293:piezo_inp r=0.0720001
r463 291:piezo_inp 294:piezo_inp r=0.0675001
r462 291:piezo_inp 292:piezo_inp r=0.900001
r461 290:piezo_inp 305:piezo_inp r=0.028125
r460 290:piezo_inp 295:piezo_inp r=0.900001
r459 289:piezo_inp 290:piezo_inp r=0.028125
r458 289:piezo_inp 303:piezo_inp r=0.109286
r457 288:piezo_inp 296:piezo_inp r=0.347143

r456 287:piezo_inp 302:piezo_inp r=0.491786
r455 286:piezo_inp 291:piezo_inp r=0.568126
r454 286:piezo_inp 295:piezo_inp r=0.253125
r453 285:piezo_inp 307:piezo_inp r=0.0739286
r452 285:piezo_inp 296:piezo_inp r=0.980358
r451 piezo_inp 306:piezo_inp r=0.0675001
r450 284:piezo_inp piezo_inp r=0.891563
r449 m30|drn 301:piezo_inp r=10
r448 m30|drn 303:piezo_inp r=10
r447 m32|drn 298:piezo_inp r=10
r446 m29|gate 296:piezo_inp r=8
r445 m31|gate 285:piezo_inp r=8
r444 283:piezo_inp 284:piezo_inp r=0.435938
r443 282:piezo_inp 297:piezo_inp r=0.0780001
r442 282:piezo_inp 288:piezo_inp r=0.276429
r441 282:piezo_inp 287:piezo_inp r=0.0660001
r440 282:piezo_inp 283:piezo_inp r=0.900001
r439 272:rf_inp 274:rf_inp r=0.234643
r438 272:rf_inp 273:rf_inp r=0.405
r437 270:rf_inp 272:rf_inp r=0.343929
r436 270:rf_inp 271:rf_inp r=0.0739286
r435 269:rf_inp 278:rf_inp r=0.260357
r434 268:rf_inp 281:rf_inp r=0.0739286
r433 268:rf_inp 269:rf_inp r=0.343929
r432 264:rf_inp 278:rf_inp r=0.0720001
r431 264:rf_inp 274:rf_inp r=0.0720001
r430 263:rf_inp 266:rf_inp r=0.531563
r429 263:rf_inp 265:rf_inp r=0.0703126
r428 263:rf_inp 264:rf_inp r=0.900001
r427 262:rf_inp 276:rf_inp r=0.028125
r426 262:rf_inp 275:rf_inp r=0.028125
r425 261:rf_inp 266:rf_inp r=0.523126
r424 261:rf_inp 262:rf_inp r=0.900001
r423 260:rf_inp 276:rf_inp r=0.414643
r422 260:rf_inp 267:rf_inp r=0.0739286
r421 259:rf_inp 280:rf_inp r=0.0739286
r420 259:rf_inp 275:rf_inp r=0.440358
r419 258:rf_inp 277:rf_inp r=0.0720001
r418 258:rf_inp 273:rf_inp r=0.0720001
r417 rf_inp 279:rf_inp r=0.0464063
r416 rf_inp 258:rf_inp r=0.900001
r415 rf_inp 257:rf_inp r=0.0435938
r414 m26|drn 272:rf_inp r=10

r413 m26|drn 270:rf_inp r=10
r412 m28|drn 268:rf_inp r=10
r411 m25|gate 260:rf_inp r=8
r410 m27|gate 259:rf_inp r=8
r409 251:vref2 252:vref2 r=1.215
r408 250:vref2 253:vref2 r=0.250715
r407 249:vref2 256:vref2 r=0.0739286
r406 249:vref2 250:vref2 r=0.295715
r405 247:vref2 255:vref2 r=0.109286
r404 247:vref2 248:vref2 r=0.0739286
r403 246:vref2 254:vref2 r=0.0346616
r402 244:vref2 245:vref2 r=0.0734211
r401 242:vref2 255:vref2 r=0.0553847
r400 242:vref2 246:vref2 r=0.0230769
r399 241:vref2 243:vref2 r=0.227813
r398 241:vref2 242:vref2 r=0.900001
r397 238:vref2 253:vref2 r=0.504643
r396 237:vref2 244:vref2 r=0.111316
r395 237:vref2 238:vref2 r=0.102857
r394 237:vref2 251:vref2 r=0.411429
r393 236:vref2 245:vref2 r=0.117
r392 236:vref2 240:vref2 r=0.0720001
r391 235:vref2 252:vref2 r=0.0720001
r390 235:vref2 239:vref2 r=0.0720001
r389 234:vref2 236:vref2 r=0.900001
r388 234:vref2 243:vref2 r=0.264375
r387 vref2 235:vref2 r=0.900001
r386 m1|drn 251:vref2 r=10
r385 m0|src 249:vref2 r=10
r384 m0|src 250:vref2 r=10
r383 m1|gate 244:vref2 r=8
r382 m5@2|gate 246:vref2 r=8
r381 m5@2|gate 247:vref2 r=8
r380 m5|gate m5@2|gate r=0.001
r379 232:ln_n126 233:ln_n126 r=2.74688
r378 ln_n126 231:ln_n126 r=0.226875
r377 ln_n126 232:ln_n126 r=0.001
r376 ln_n46 230:ln_n46 r=0.0600001
r375 228:ln_n46 ln_n46 r=0.0900001
r374 227:ln_n46 229:ln_n46 r=0.0600001
r373 227:ln_n46 228:ln_n46 r=0.0900001
r372 226:ln_n46 227:ln_n46 r=0.900001
r371 226:ln_n46 228:ln_n46 r=0.900001

r370 226:ln_n46 ln_n46 r=0.900001
r369 ln_n33 225:ln_n33 r=0.0600001
r368 223:ln_n33 ln_n33 r=0.0900001
r367 222:ln_n33 224:ln_n33 r=0.0600001
r366 222:ln_n33 223:ln_n33 r=0.0900001
r365 221:ln_n33 222:ln_n33 r=0.900001
r364 221:ln_n33 223:ln_n33 r=0.900001
r363 221:ln_n33 ln_n33 r=0.900001
r362 ln_n38 220:ln_n38 r=0.0750001
r361 219:ln_n38 ln_n38 r=0.0900001
r360 218:ln_n38 219:ln_n38 r=0.0900001
r359 217:ln_n38 218:ln_n38 r=0.0900001
r358 215:ln_n38 217:ln_n38 r=0.0900001
r357 214:ln_n38 216:ln_n38 r=0.0750001
r356 214:ln_n38 215:ln_n38 r=0.0900001
r355 213:ln_n38 215:ln_n38 r=0.900001
r354 213:ln_n38 218:ln_n38 r=0.900001
r353 213:ln_n38 ln_n38 r=0.900001
r352 213:ln_n38 214:ln_n38 r=0.900001
r351 213:ln_n38 217:ln_n38 r=0.900001
r350 213:ln_n38 219:ln_n38 r=0.900001
r349 ln_n45 212:ln_n45 r=0.0540001
r348 211:ln_n45 ln_n45 r=0.0900001
r347 210:ln_n45 211:ln_n45 r=0.0900001
r346 209:ln_n45 210:ln_n45 r=0.0900001
r345 208:ln_n45 209:ln_n45 r=0.0900001
r344 207:ln_n45 208:ln_n45 r=0.0900001
r343 206:ln_n45 207:ln_n45 r=0.0900001
r342 205:ln_n45 206:ln_n45 r=0.0900001
r341 203:ln_n45 205:ln_n45 r=0.0900001
r340 202:ln_n45 204:ln_n45 r=0.0540001
r339 202:ln_n45 203:ln_n45 r=0.0900001
r338 201:ln_n45 210:ln_n45 r=0.900001
r337 201:ln_n45 205:ln_n45 r=0.900001
r336 201:ln_n45 208:ln_n45 r=0.900001
r335 201:ln_n45 206:ln_n45 r=0.900001
r334 201:ln_n45 ln_n45 r=0.900001
r333 201:ln_n45 202:ln_n45 r=0.900001
r332 201:ln_n45 203:ln_n45 r=0.900001
r331 201:ln_n45 207:ln_n45 r=0.900001
r330 201:ln_n45 209:ln_n45 r=0.900001
r329 201:ln_n45 211:ln_n45 r=0.900001
r328 ln_n80 200:ln_n80 r=0.0409091

r327 199:ln_n80 ln_n80 r=0.0818183
r326 198:ln_n80 199:ln_n80 r=0.0818183
r325 196:ln_n80 198:ln_n80 r=0.0818183
r324 195:ln_n80 197:ln_n80 r=0.0409091
r323 195:ln_n80 196:ln_n80 r=0.0818183
r322 194:ln_n80 ln_n80 r=0.900001
r321 194:ln_n80 198:ln_n80 r=0.900001
r320 194:ln_n80 195:ln_n80 r=0.900001
r319 194:ln_n80 196:ln_n80 r=0.900001
r318 194:ln_n80 199:ln_n80 r=0.900001
r317 ln_n67 193:ln_n67 r=0.0409091
r316 192:ln_n67 ln_n67 r=0.0818183
r315 191:ln_n67 192:ln_n67 r=0.0818183
r314 189:ln_n67 191:ln_n67 r=0.0818183
r313 188:ln_n67 190:ln_n67 r=0.0409091
r312 188:ln_n67 189:ln_n67 r=0.0818183
r311 187:ln_n67 ln_n67 r=0.900001
r310 187:ln_n67 191:ln_n67 r=0.900001
r309 187:ln_n67 188:ln_n67 r=0.900001
r308 187:ln_n67 189:ln_n67 r=0.900001
r307 187:ln_n67 192:ln_n67 r=0.900001
r306 ln_n64 186:ln_n64 r=0.0540001
r305 185:ln_n64 ln_n64 r=0.0900001
r304 184:ln_n64 185:ln_n64 r=0.0900001
r303 183:ln_n64 184:ln_n64 r=0.0900001
r302 182:ln_n64 183:ln_n64 r=0.0900001
r301 181:ln_n64 182:ln_n64 r=0.0900001
r300 180:ln_n64 181:ln_n64 r=0.0900001
r299 179:ln_n64 180:ln_n64 r=0.0900001
r298 177:ln_n64 179:ln_n64 r=0.0900001
r297 176:ln_n64 178:ln_n64 r=0.0540001
r296 176:ln_n64 177:ln_n64 r=0.0900001
r295 175:ln_n64 182:ln_n64 r=0.900001
r294 175:ln_n64 180:ln_n64 r=0.900001
r293 175:ln_n64 ln_n64 r=0.900001
r292 175:ln_n64 176:ln_n64 r=0.900001
r291 175:ln_n64 177:ln_n64 r=0.900001
r290 175:ln_n64 181:ln_n64 r=0.900001
r289 175:ln_n64 183:ln_n64 r=0.900001
r288 175:ln_n64 185:ln_n64 r=0.900001
r287 175:ln_n64 184:ln_n64 r=0.900001
r286 175:ln_n64 179:ln_n64 r=0.900001
r285 ln_n47 174:ln_n47 r=0.0600001

r284 172:ln_n47 ln_n47 r=0.0900001
r283 171:ln_n47 173:ln_n47 r=0.0600001
r282 171:ln_n47 172:ln_n47 r=0.0900001
r281 170:ln_n47 171:ln_n47 r=0.900001
r280 170:ln_n47 172:ln_n47 r=0.900001
r279 170:ln_n47 ln_n47 r=0.900001
r278 ln_n44 169:ln_n44 r=0.0600001
r277 167:ln_n44 ln_n44 r=0.0900001
r276 166:ln_n44 168:ln_n44 r=0.0600001
r275 166:ln_n44 167:ln_n44 r=0.0900001
r274 165:ln_n44 166:ln_n44 r=0.900001
r273 165:ln_n44 167:ln_n44 r=0.900001
r272 165:ln_n44 ln_n44 r=0.900001
r271 ln_n42 164:ln_n42 r=0.0600001
r270 162:ln_n42 ln_n42 r=0.0900001
r269 161:ln_n42 163:ln_n42 r=0.0600001
r268 161:ln_n42 162:ln_n42 r=0.0900001
r267 160:ln_n42 161:ln_n42 r=0.900001
r266 160:ln_n42 162:ln_n42 r=0.900001
r265 160:ln_n42 ln_n42 r=0.900001
r264 ln_n60 159:ln_n60 r=0.0368182
r263 158:ln_n60 ln_n60 r=0.0818183
r262 157:ln_n60 158:ln_n60 r=0.0818183
r261 156:ln_n60 157:ln_n60 r=0.0818183
r260 155:ln_n60 156:ln_n60 r=0.0818183
r259 154:ln_n60 155:ln_n60 r=0.0818183
r258 153:ln_n60 154:ln_n60 r=0.0818183
r257 152:ln_n60 153:ln_n60 r=0.0818183
r256 151:ln_n60 152:ln_n60 r=0.0818183
r255 149:ln_n60 151:ln_n60 r=0.0818183
r254 148:ln_n60 150:ln_n60 r=0.0368182
r253 148:ln_n60 149:ln_n60 r=0.0818183
r252 147:ln_n60 154:ln_n60 r=0.900001
r251 147:ln_n60 155:ln_n60 r=0.900001
r250 147:ln_n60 156:ln_n60 r=0.900001
r249 147:ln_n60 157:ln_n60 r=0.900001
r248 147:ln_n60 158:ln_n60 r=0.900001
r247 147:ln_n60 ln_n60 r=0.900001
r246 147:ln_n60 148:ln_n60 r=0.900001
r245 147:ln_n60 149:ln_n60 r=0.900001
r244 147:ln_n60 151:ln_n60 r=0.900001
r243 147:ln_n60 152:ln_n60 r=0.900001
r242 147:ln_n60 153:ln_n60 r=0.900001

r241 ln_n30 146:ln_n30 r=0.0540001
r240 145:ln_n30 ln_n30 r=0.0900001
r239 144:ln_n30 145:ln_n30 r=0.0900001
r238 143:ln_n30 144:ln_n30 r=0.0900001
r237 142:ln_n30 143:ln_n30 r=0.0900001
r236 141:ln_n30 142:ln_n30 r=0.0900001
r235 140:ln_n30 141:ln_n30 r=0.0900001
r234 139:ln_n30 140:ln_n30 r=0.0900001
r233 137:ln_n30 139:ln_n30 r=0.0900001
r232 136:ln_n30 138:ln_n30 r=0.0540001
r231 136:ln_n30 137:ln_n30 r=0.0900001
r230 135:ln_n30 140:ln_n30 r=0.900001
r229 135:ln_n30 145:ln_n30 r=0.900001
r228 135:ln_n30 141:ln_n30 r=0.900001
r227 135:ln_n30 ln_n30 r=0.900001
r226 135:ln_n30 142:ln_n30 r=0.900001
r225 135:ln_n30 136:ln_n30 r=0.900001
r224 135:ln_n30 137:ln_n30 r=0.900001
r223 135:ln_n30 143:ln_n30 r=0.900001
r222 135:ln_n30 144:ln_n30 r=0.900001
r221 135:ln_n30 139:ln_n30 r=0.900001
r220 ln_n36 134:ln_n36 r=0.0540001
r219 133:ln_n36 ln_n36 r=0.0900001
r218 132:ln_n36 133:ln_n36 r=0.0900001
r217 131:ln_n36 132:ln_n36 r=0.0900001
r216 130:ln_n36 131:ln_n36 r=0.0900001
r215 129:ln_n36 130:ln_n36 r=0.0900001
r214 128:ln_n36 129:ln_n36 r=0.0900001
r213 127:ln_n36 128:ln_n36 r=0.0900001
r212 125:ln_n36 127:ln_n36 r=0.0900001
r211 124:ln_n36 126:ln_n36 r=0.0540001
r210 124:ln_n36 125:ln_n36 r=0.0900001
r209 123:ln_n36 128:ln_n36 r=0.900001
r208 123:ln_n36 133:ln_n36 r=0.900001
r207 123:ln_n36 129:ln_n36 r=0.900001
r206 123:ln_n36 ln_n36 r=0.900001
r205 123:ln_n36 130:ln_n36 r=0.900001
r204 123:ln_n36 124:ln_n36 r=0.900001
r203 123:ln_n36 125:ln_n36 r=0.900001
r202 123:ln_n36 131:ln_n36 r=0.900001
r201 123:ln_n36 132:ln_n36 r=0.900001
r200 123:ln_n36 127:ln_n36 r=0.900001
r199 ln_n34 122:ln_n34 r=0.0600001

r198 120:ln_n34 ln_n34 r=0.0900001
r197 119:ln_n34 121:ln_n34 r=0.0600001
r196 119:ln_n34 120:ln_n34 r=0.0900001
r195 118:ln_n34 119:ln_n34 r=0.900001
r194 118:ln_n34 120:ln_n34 r=0.900001
r193 118:ln_n34 ln_n34 r=0.900001
r192 ln_n31 117:ln_n31 r=0.051
r191 116:ln_n31 ln_n31 r=0.0900001
r190 115:ln_n31 116:ln_n31 r=0.0900001
r189 114:ln_n31 115:ln_n31 r=0.0900001
r188 113:ln_n31 114:ln_n31 r=0.0900001
r187 111:ln_n31 113:ln_n31 r=0.0900001
r186 110:ln_n31 112:ln_n31 r=0.051
r185 110:ln_n31 111:ln_n31 r=0.0900001
r184 109:ln_n31 116:ln_n31 r=0.900001
r183 109:ln_n31 ln_n31 r=0.900001
r182 109:ln_n31 113:ln_n31 r=0.900001
r181 109:ln_n31 114:ln_n31 r=0.900001
r180 109:ln_n31 115:ln_n31 r=0.900001
r179 109:ln_n31 110:ln_n31 r=0.900001
r178 109:ln_n31 111:ln_n31 r=0.900001
r177 ln_n52 108:ln_n52 r=0.0368182
r176 107:ln_n52 ln_n52 r=0.0818183
r175 106:ln_n52 107:ln_n52 r=0.0818183
r174 105:ln_n52 106:ln_n52 r=0.0818183
r173 104:ln_n52 105:ln_n52 r=0.0818183
r172 103:ln_n52 104:ln_n52 r=0.0818183
r171 102:ln_n52 103:ln_n52 r=0.0818183
r170 101:ln_n52 102:ln_n52 r=0.0818183
r169 100:ln_n52 101:ln_n52 r=0.0818183
r168 98:ln_n52 100:ln_n52 r=0.0818183
r167 97:ln_n52 99:ln_n52 r=0.0368182
r166 97:ln_n52 98:ln_n52 r=0.0818183
r165 96:ln_n52 97:ln_n52 r=0.900001
r164 96:ln_n52 98:ln_n52 r=0.900001
r163 96:ln_n52 100:ln_n52 r=0.900001
r162 96:ln_n52 101:ln_n52 r=0.900001
r161 96:ln_n52 102:ln_n52 r=0.900001
r160 96:ln_n52 103:ln_n52 r=0.900001
r159 96:ln_n52 104:ln_n52 r=0.900001
r158 96:ln_n52 105:ln_n52 r=0.900001
r157 96:ln_n52 106:ln_n52 r=0.900001
r156 96:ln_n52 107:ln_n52 r=0.900001

r155 96:ln_n52 ln_n52 r=0.900001
r154 ln_n37 95:ln_n37 r=0.051
r153 94:ln_n37 ln_n37 r=0.0900001
r152 93:ln_n37 94:ln_n37 r=0.0900001
r151 92:ln_n37 93:ln_n37 r=0.0900001
r150 91:ln_n37 92:ln_n37 r=0.0900001
r149 89:ln_n37 91:ln_n37 r=0.0900001
r148 88:ln_n37 90:ln_n37 r=0.051
r147 88:ln_n37 89:ln_n37 r=0.0900001
r146 87:ln_n37 94:ln_n37 r=0.900001
r145 87:ln_n37 ln_n37 r=0.900001
r144 87:ln_n37 91:ln_n37 r=0.900001
r143 87:ln_n37 92:ln_n37 r=0.900001
r142 87:ln_n37 93:ln_n37 r=0.900001
r141 87:ln_n37 88:ln_n37 r=0.900001
r140 87:ln_n37 89:ln_n37 r=0.900001
r139 ln_n54 86:ln_n54 r=0.0409091
r138 85:ln_n54 ln_n54 r=0.0818183
r137 84:ln_n54 85:ln_n54 r=0.0818183
r136 82:ln_n54 84:ln_n54 r=0.0818183
r135 81:ln_n54 83:ln_n54 r=0.0409091
r134 81:ln_n54 82:ln_n54 r=0.0818183
r133 80:ln_n54 81:ln_n54 r=0.900001
r132 80:ln_n54 84:ln_n54 r=0.900001
r131 80:ln_n54 ln_n54 r=0.900001
r130 80:ln_n54 82:ln_n54 r=0.900001
r129 80:ln_n54 85:ln_n54 r=0.900001
r128 ln_n56 79:ln_n56 r=0.0409091
r127 78:ln_n56 ln_n56 r=0.0818183
r126 77:ln_n56 78:ln_n56 r=0.0818183
r125 75:ln_n56 77:ln_n56 r=0.0818183
r124 74:ln_n56 76:ln_n56 r=0.0409091
r123 74:ln_n56 75:ln_n56 r=0.0818183
r122 73:ln_n56 74:ln_n56 r=0.900001
r121 73:ln_n56 77:ln_n56 r=0.900001
r120 73:ln_n56 ln_n56 r=0.900001
r119 73:ln_n56 75:ln_n56 r=0.900001
r118 73:ln_n56 78:ln_n56 r=0.900001
r117 ln_n32 72:ln_n32 r=0.0600001
r116 70:ln_n32 ln_n32 r=0.0900001
r115 69:ln_n32 71:ln_n32 r=0.0600001
r114 69:ln_n32 70:ln_n32 r=0.0900001
r113 68:ln_n32 69:ln_n32 r=0.900001

r112 68:ln_n32 70:ln_n32 r=0.900001
r111 68:ln_n32 ln_n32 r=0.900001
r110 ln_n55 67:ln_n55 r=0.0750001
r109 66:ln_n55 ln_n55 r=0.0900001
r108 65:ln_n55 66:ln_n55 r=0.0900001
r107 64:ln_n55 65:ln_n55 r=0.0900001
r106 62:ln_n55 64:ln_n55 r=0.0900001
r105 61:ln_n55 63:ln_n55 r=0.0750001
r104 61:ln_n55 62:ln_n55 r=0.0900001
r103 60:ln_n55 62:ln_n55 r=0.900001
r102 60:ln_n55 65:ln_n55 r=0.900001
r101 60:ln_n55 ln_n55 r=0.900001
r100 60:ln_n55 61:ln_n55 r=0.900001
r99 60:ln_n55 64:ln_n55 r=0.900001
r98 60:ln_n55 66:ln_n55 r=0.900001
r97 ln_n48 59:ln_n48 r=0.0368182
r96 58:ln_n48 ln_n48 r=0.0818183
r95 57:ln_n48 58:ln_n48 r=0.0818183
r94 56:ln_n48 57:ln_n48 r=0.0818183
r93 55:ln_n48 56:ln_n48 r=0.0818183
r92 54:ln_n48 55:ln_n48 r=0.0818183
r91 53:ln_n48 54:ln_n48 r=0.0818183
r90 52:ln_n48 53:ln_n48 r=0.0818183
r89 51:ln_n48 52:ln_n48 r=0.0818183
r88 49:ln_n48 51:ln_n48 r=0.0818183
r87 48:ln_n48 50:ln_n48 r=0.0368182
r86 48:ln_n48 49:ln_n48 r=0.0818183
r85 47:ln_n48 48:ln_n48 r=0.900001
r84 47:ln_n48 49:ln_n48 r=0.900001
r83 47:ln_n48 51:ln_n48 r=0.900001
r82 47:ln_n48 52:ln_n48 r=0.900001
r81 47:ln_n48 53:ln_n48 r=0.900001
r80 47:ln_n48 54:ln_n48 r=0.900001
r79 47:ln_n48 55:ln_n48 r=0.900001
r78 47:ln_n48 56:ln_n48 r=0.900001
r77 47:ln_n48 57:ln_n48 r=0.900001
r76 47:ln_n48 58:ln_n48 r=0.900001
r75 47:ln_n48 ln_n48 r=0.900001
r74 ln_n25 46:ln_n25 r=0.0540001
r73 45:ln_n25 ln_n25 r=0.0900001
r72 44:ln_n25 45:ln_n25 r=0.0900001
r71 43:ln_n25 44:ln_n25 r=0.0900001
r70 42:ln_n25 43:ln_n25 r=0.0900001

r69 41:ln_n25 42:ln_n25 r=0.0900001
r68 40:ln_n25 41:ln_n25 r=0.0900001
r67 39:ln_n25 40:ln_n25 r=0.0900001
r66 37:ln_n25 39:ln_n25 r=0.0900001
r65 36:ln_n25 38:ln_n25 r=0.0540001
r64 36:ln_n25 37:ln_n25 r=0.0900001
r63 35:ln_n25 41:ln_n25 r=0.900001
r62 35:ln_n25 42:ln_n25 r=0.900001
r61 35:ln_n25 43:ln_n25 r=0.900001
r60 35:ln_n25 44:ln_n25 r=0.900001
r59 35:ln_n25 45:ln_n25 r=0.900001
r58 35:ln_n25 ln_n25 r=0.900001
r57 35:ln_n25 36:ln_n25 r=0.900001
r56 35:ln_n25 37:ln_n25 r=0.900001
r55 35:ln_n25 39:ln_n25 r=0.900001
r54 35:ln_n25 40:ln_n25 r=0.900001
r53 ln_n27 34:ln_n27 r=0.0540001
r52 33:ln_n27 ln_n27 r=0.0900001
r51 32:ln_n27 33:ln_n27 r=0.0900001
r50 31:ln_n27 32:ln_n27 r=0.0900001
r49 30:ln_n27 31:ln_n27 r=0.0900001
r48 29:ln_n27 30:ln_n27 r=0.0900001
r47 28:ln_n27 29:ln_n27 r=0.0900001
r46 27:ln_n27 28:ln_n27 r=0.0900001
r45 25:ln_n27 27:ln_n27 r=0.0900001
r44 24:ln_n27 26:ln_n27 r=0.0540001
r43 24:ln_n27 25:ln_n27 r=0.0900001
r42 23:ln_n27 33:ln_n27 r=0.900001
r41 23:ln_n27 27:ln_n27 r=0.900001
r40 23:ln_n27 29:ln_n27 r=0.900001
r39 23:ln_n27 ln_n27 r=0.900001
r38 23:ln_n27 31:ln_n27 r=0.900001
r37 23:ln_n27 32:ln_n27 r=0.900001
r36 23:ln_n27 25:ln_n27 r=0.900001
r35 23:ln_n27 28:ln_n27 r=0.900001
r34 23:ln_n27 24:ln_n27 r=0.900001
r33 23:ln_n27 30:ln_n27 r=0.900001
r32 ln_n26 22:ln_n26 r=0.0600001
r31 20:ln_n26 ln_n26 r=0.0900001
r30 19:ln_n26 21:ln_n26 r=0.0600001
r29 19:ln_n26 20:ln_n26 r=0.0900001
r28 18:ln_n26 19:ln_n26 r=0.900001
r27 18:ln_n26 20:ln_n26 r=0.900001

```
r26 18:ln_n26 ln_n26  r=0.900001
r25 ln_n24 17:ln_n24  r=0.0540001
r24 16:ln_n24 ln_n24  r=0.0900001
r23 15:ln_n24 16:ln_n24  r=0.0900001
r22 14:ln_n24 15:ln_n24  r=0.0900001
r21 13:ln_n24 14:ln_n24  r=0.0900001
r20 12:ln_n24 13:ln_n24  r=0.0900001
r19 11:ln_n24 12:ln_n24  r=0.0900001
r18 10:ln_n24 11:ln_n24  r=0.0900001
r17 8:ln_n24 10:ln_n24  r=0.0900001
r16 7:ln_n24 9:ln_n24  r=0.0540001
r15 7:ln_n24 8:ln_n24  r=0.0900001
r14 6:ln_n24 13:ln_n24  r=0.900001
r13 6:ln_n24 16:ln_n24  r=0.900001
r12 6:ln_n24 10:ln_n24  r=0.900001
r11 6:ln_n24 12:ln_n24  r=0.900001
r10 6:ln_n24 ln_n24  r=0.900001
r9 6:ln_n24 14:ln_n24  r=0.900001
r8 6:ln_n24 15:ln_n24  r=0.900001
r7 6:ln_n24 8:ln_n24  r=0.900001
r6 6:ln_n24 11:ln_n24  r=0.900001
r5 6:ln_n24 7:ln_n24  r=0.900001
r4 ln_n128 4:ln_n128  r=2.5737
r3 ln_n128 5:ln_n128  r=0.001
r2 3:ln_n_generated_130 ln_n_generated_130  r=1.24688
r1 2:ln_n_generated_129 ln_n_generated_129  r=1.4625
d9 vreg d9|cathode pd area=1p l=1u w=1u m=1
d10 gnd_1 d10|cathode pd area=0.0529p l=0.23u w=0.23u m=1
m6 m6|drn m6|gate m6|src gnd_1 n12_lvt w=0.23u l=0.1u nf=1 m=1
m32 m32|drn m32|gate m32|src gnd_1 n12_lvt w=0.23u l=0.1u nf=1 m=1
m31 m31|drn m31|gate m31|src gnd_1 n12_lvt w=0.23u l=0.1u nf=1 m=1
m28 m28|drn m28|gate m28|src gnd_1 n12_lvt w=0.23u l=0.1u nf=1 m=1
m27 m27|drn m27|gate m27|src gnd_1 n12_lvt w=0.23u l=0.1u nf=1 m=1
m24 m24|drn m24|gate m24|src gnd_1 n12_lvt w=0.23u l=0.1u nf=1 m=1
m15@2 m15@2|drn m15@2|gate m15@2|src gnd_1 n12_lvt w=0.69u l=0.1u nf=1 m=1
m15 m15|drn m15|gate m15|src gnd_1 n12_lvt w=0.69u l=0.1u nf=1 m=1
m14 m14|drn m14|gate m14|src gnd_1 n12_lvt w=0.69u l=0.1u nf=1 m=1
m13 m13|drn m13|gate m13|src gnd_1 n12_lvt w=0.23u l=0.1u nf=1 m=1
m12 m12|drn m12|gate m12|src gnd_1 n12_lvt w=0.23u l=0.1u nf=1 m=1
m0 m0|drn m0|gate m0|src gnd_1 n12_lvt w=0.69u l=0.1u nf=1 m=1
m7 m7|drn m7|gate m7|src gnd_1 n12_hvt w=0.23u l=0.1u nf=1 m=1
m4 m4|drn m4|gate m4|src gnd_1 n12_hvt w=0.46u l=0.1u nf=1 m=1
m3 m3|drn m3|gate m3|src gnd_1 n12_hvt w=0.46u l=0.1u nf=1 m=1
```

```
m23@2 m23@2|drn m23@2|gate m23@2|src gnd_1 n12_hvt w=0.69u l=0.1u nf=1 m=1
m23 m23|drn m23|gate m23|src gnd_1 n12_hvt w=0.69u l=0.1u nf=1 m=1
m22@2 m22@2|drn m22@2|gate m22@2|src gnd_1 n12_hvt w=0.69u l=0.1u nf=1 m=1
m22 m22|drn m22|gate m22|src gnd_1 n12_hvt w=0.69u l=0.1u nf=1 m=1
m21@2 m21@2|drn m21@2|gate m21@2|src gnd_1 n12_hvt w=1.69u l=0.1u nf=1 m=1
m21 m21|drn m21|gate m21|src gnd_1 n12_hvt w=0.69u l=0.1u nf=1 m=1
m20 m20|drn m20|gate m20|src gnd_1 n12_hvt w=0.23u l=0.1u nf=1 m=1
m2 m2|drn m2|gate m2|src gnd_1 n12_hvt w=0.46u l=0.1u nf=1 m=1
m1 m1|drn m1|gate m1|src gnd_1 n12_hvt w=0.46u l=0.1u nf=1 m=1
m9 m9|drn m9|gate m9|src m15|drn p12_hvt w=0.69u l=0.1u nf=1 m=1
m11 m11|drn m11|gate m11|src m15|drn p12_hvt w=0.23u l=0.4u nf=1 m=1
m10 m10|drn m10|gate m10|src m15|drn p12_hvt w=0.23u l=0.1u nf=1 m=1
m8 m8|drn m8|gate m8|src m15|drn p12_lvt w=0.69u l=0.1u nf=1 m=1
m5@2 m5@2|drn m5@2|gate m5@2|src primary_storage p12_lvt w=0.69u l=0.1u nf=1
m=1
m5 m5|drn m5|gate m5|src primary_storage p12_lvt w=0.69u l=0.1u nf=1 m=1
m30 m30|drn m30|gate m30|src c1|plus p12_lvt w=0.72u l=0.1u nf=1 m=1
m29 m29|drn m29|gate m29|src c1|plus p12_lvt w=0.72u l=0.1u nf=1 m=1
m26 m26|drn m26|gate m26|src c21|plus p12_lvt w=0.72u l=0.1u nf=1 m=1
m25 m25|drn m25|gate m25|src c21|plus p12_lvt w=0.72u l=0.1u nf=1 m=1
xc8 c8|plus c8|minus ccap w=0.266338 l=4u nf=4 startmetal=1 endmetal=2
xc21 c21|plus c21|minus ccap w=0.300324 l=4u nf=4 startmetal=1 endmetal=2
xc19 c19|plus c19|minus ccap w=0.3 l=4u nf=4 startmetal=1 endmetal=2
xc18 c18|plus c18|minus ccap w=0.266338 l=4u nf=4 startmetal=1 endmetal=2
xc17 c17|plus c17|minus ccap w=0.266338 l=4u nf=4 startmetal=1 endmetal=2
xc16 c16|plus c16|minus ccap w=0.266338 l=4u nf=4 startmetal=1 endmetal=2
xc15 c15|plus c15|minus ccap w=0.280684 l=4u nf=4 startmetal=1 endmetal=2
xc1 c1|plus c1|minus ccap w=0.300324 l=4u nf=4 startmetal=1 endmetal=2
.ends ehs_ip
************************************************************************
* Library          : Rectifier
* Cell             : load_3c
* View             : schematic
* View Search List : schematic symbol hspice
* View Stop List   : symbol
************************************************************************
.subckt load_3c vsupply lm_done
r33 net74 net73 r=2180k
r32 net72 net71 r=2180k
r31 net67 net68 r=2180k
r0 vsupply gnd! r=255k
xc29 net73 gnd! gnd! fm3m1 sp_length=4.22232m m=1
xc19 lm_done gnd! gnd! fm3m1 sp_length=4.22232m m=1
```

```
xc24 net55 gnd! gnd! fm3m1 sp_length=4.22232m m=1
xc25 net68 gnd! gnd! fm3m1 sp_length=4.22232m m=1
xc27 net71 gnd! gnd! fm3m1 sp_length=4.22232m m=1
xc1 vsupply gnd! ccap w=0.300324 l=0.94u nf=4 startmetal=1 endmetal=2
m15 lm_done net73 gnd! gnd! n12_lvt w=0.23u l=0.1u nf=1.0 m=1 ad=0.3456n
as=0.35904n
+  pd=1.30160m ps=1.36644m nrd=0.3375 nrs=0.350625 sa=0.48u sb=0.48u sd=0.54u
m13 net74 net71 gnd! gnd! n12_lvt w=0.23u l=0.1u nf=1.0 m=1 ad=0.3456n
as=0.35904n
+  pd=1.30160m ps=1.36644m nrd=0.3375 nrs=0.350625 sa=0.48u sb=0.48u sd=0.54u
m11 net72 net68 gnd! gnd! n12_lvt w=0.23u l=0.1u nf=1.0 m=1 ad=0.3456n
as=0.35904n
+  pd=1.30160m ps=1.36644m nrd=0.3375 nrs=0.350625 sa=0.48u sb=0.48u sd=0.54u
m9 net67 net55 gnd! gnd! n12_lvt w=0.23u l=0.1u nf=1.0 m=1 ad=0.3456n as=0.35904n
+  pd=1.30160m ps=1.36644m nrd=0.3375 nrs=0.350625 sa=0.48u sb=0.48u sd=0.54u
m3 net55 net13 gnd! gnd! n12_lvt w=0.23u l=0.1u nf=1.0 m=1 ad=0.3456n as=0.35904n
+  pd=1.30160m ps=1.36644m nrd=0.3375 nrs=0.350625 sa=0.48u sb=0.48u sd=0.54u
m141 net13 vsupply gnd! gnd! n12_lvt w=0.23u l=0.1u nf=1.0 m=1 ad=0.3456n
as=0.35904n
+  pd=1.30160m ps=1.36644m nrd=0.3375 nrs=0.350625 sa=0.48u sb=0.48u sd=0.54u
v23 net86 gnd! dc=1.2
m14 lm_done net73 net86 net86 p12_hvt w=0.23u l=0.1u nf=1.0 m=1 ad=0.108n
as=0.1164n
+  pd=0.410800m ps=0.451640m nrd=0.27 nrs=0.291 sa=0.48u sb=0.48u sd=0.54u
m12 net74 net71 net86 net86 p12_hvt w=0.23u l=0.1u nf=1.0 m=1 ad=0.3456n
as=0.35904n
+  pd=1.30160m ps=1.36644m nrd=0.3375 nrs=0.350625 sa=0.48u sb=0.48u sd=0.54u
m10 net72 net68 net86 net86 p12_hvt w=0.23u l=0.1u nf=1.0 m=1 ad=0.3456n
as=0.35904n
+  pd=1.30160m ps=1.36644m nrd=0.3375 nrs=0.350625 sa=0.48u sb=0.48u sd=0.54u
m8 net67 net55 net86 net86 p12_hvt w=0.23u l=0.1u nf=1.0 m=1 ad=0.3456n
as=0.35904n
+  pd=1.30160m ps=1.36644m nrd=0.3375 nrs=0.350625 sa=0.48u sb=0.48u sd=0.54u
m2 net55 net13 vsupply vsupply p12_hvt w=0.23u l=0.1u nf=1.0 m=1 ad=0.3456n
as=0.35904n
+  pd=1.30160m ps=1.36644m nrd=0.3375 nrs=0.350625 sa=0.48u sb=0.48u sd=0.54u
m140 net13 vsupply vsupply vsupply p12_hvt w=0.23u l=0.1u nf=1.0 m=1
ad=0.3456n
+ as=0.35904n pd=1.30160m ps=1.36644m nrd=0.3375 nrs=0.350625 sa=0.48u sb=0.48u
+ sd=0.54u
.ends load_3c
*******************************************************************************
* Library          : Rectifier
```

```
* Cell             : load_3e
* View             : schematic
* View Search List : schematic symbol hspice
* View Stop List   : symbol
******************************************************************************
.subckt load_3e vsupply lm_done
r33 net74 net73 r=111k
r32 net72 net71 r=121k
r31 net67 net68 r=121k
r0 vsupply gnd! r=315k
xc29 net73 gnd! gnd! fm3m1 sp_length=4.22232m m=1
xc19 lm_done gnd! gnd! fm3m1 sp_length=4.22232m m=1
xc24 net55 gnd! gnd! fm3m1 sp_length=4.22232m m=1
xc25 net68 gnd! gnd! fm3m1 sp_length=4.22232m m=1
xc27 net71 gnd! gnd! fm3m1 sp_length=4.22232m m=1
xc1 net71 gnd! ccap w=0.300324 l=2u nf=4 startmetal=1 endmetal=2
xc2 net71 gnd! ccap w=0.300324 l=2u nf=4 startmetal=1 endmetal=2
xc3 net55 gnd! ccap w=0.300324 l=1u nf=4 startmetal=1 endmetal=2
xc14 vsupply gnd! gnd! fm3m1 sp_length=0.12232m m=1
m15 lm_done net73 gnd! gnd! n12_lvt w=0.23u l=0.1u nf=1.0 m=1 ad=0.3456n
as=0.35904n
+  pd=1.30160m ps=1.36644m nrd=0.3375 nrs=0.350625 sa=0.48u sb=0.48u sd=0.54u
m13 net74 net71 gnd! gnd! n12_lvt w=0.23u l=0.1u nf=1.0 m=1 ad=0.3456n
as=0.35904n
+  pd=1.30160m ps=1.36644m nrd=0.3375 nrs=0.350625 sa=0.48u sb=0.48u sd=0.54u
m11 net72 net68 gnd! gnd! n12_lvt w=0.23u l=0.1u nf=1.0 m=1 ad=0.3456n
as=0.35904n
+  pd=1.30160m ps=1.36644m nrd=0.3375 nrs=0.350625 sa=0.48u sb=0.48u sd=0.54u
m9 net67 net55 gnd! gnd! n12_lvt w=0.23u l=0.1u nf=1.0 m=1 ad=0.3456n as=0.35904n
+  pd=1.30160m ps=1.36644m nrd=0.3375 nrs=0.350625 sa=0.48u sb=0.48u sd=0.54u
m3 net55 net13 gnd! gnd! n12_lvt w=0.23u l=0.1u nf=1.0 m=1 ad=0.3456n as=0.35904n
+  pd=1.30160m ps=1.36644m nrd=0.3375 nrs=0.350625 sa=0.48u sb=0.48u sd=0.54u
m141 net13 vsupply gnd! gnd! n12_lvt w=0.23u l=0.1u nf=1.0 m=1 ad=0.3456n
as=0.35904n
+  pd=1.30160m ps=1.36644m nrd=0.3375 nrs=0.350625 sa=0.48u sb=0.48u sd=0.54u
v23 net86 gnd! dc=1.2
m14 lm_done net73 net86 net86 p12_hvt w=0.23u l=0.1u nf=1.0 m=1 ad=0.108n
as=0.1164n
+  pd=0.410800m ps=0.451640m nrd=0.27 nrs=0.291 sa=0.48u sb=0.48u sd=0.54u
m12 net74 net71 net86 net86 p12_hvt w=0.23u l=0.1u nf=1.0 m=1 ad=0.3456n
as=0.35904n
+  pd=1.30160m ps=1.36644m nrd=0.3375 nrs=0.350625 sa=0.48u sb=0.48u sd=0.54u
```

```
m10 net72 net68 net86 net86 p12_hvt w=0.23u l=0.1u nf=1.0 m=1 ad=0.3456n
as=0.35904n
+  pd=1.30160m ps=1.36644m nrd=0.3375 nrs=0.350625 sa=0.48u sb=0.48u sd=0.54u
m8 net67 net55 net86 net86 p12_hvt w=0.23u l=0.1u nf=1.0 m=1 ad=0.3456n
as=0.35904n
+  pd=1.30160m ps=1.36644m nrd=0.3375 nrs=0.350625 sa=0.48u sb=0.48u sd=0.54u
m2 net55 net13 vsupply vsupply p12_hvt w=0.23u l=0.1u nf=1.0 m=1 ad=0.3456n
as=0.35904n
+  pd=1.30160m ps=1.36644m nrd=0.3375 nrs=0.350625 sa=0.48u sb=0.48u sd=0.54u
m140 net13 vsupply vsupply vsupply p12_hvt w=0.23u l=0.1u nf=1.0 m=1
ad=0.3456n
+ as=0.35904n pd=1.30160m ps=1.36644m nrd=0.3375 nrs=0.350625 sa=0.48u sb=0.48u
+ sd=0.54u

.ends load_3e
************************************************************************
* Library          : Rectifier0
* Cell             : mux0
* View             : schematic
* View Search List : schematic symbol hspice
* View Stop List   : symbol
************************************************************************
.subckt mux0 clk in in1 out0
xm15 net46 clk gnd! gnd! nhp w=64u l=0.18u as=24p ad=17.28p ps=99u pd=66.16u
+ nrs=93.750m nrd=67.50m m=1 sa=0.48u sb=0.48u sd=0.54u nf=4
xm1 out0 net46 in gnd! nhp w=64u l=0.18u as=30.72p ad=17.28p ps=0.129920m
pd=65.08u
+ nrs=30m nrd=16.8750m m=1 sa=0.48u sb=0.48u sd=0.54u nf=2
xm0 out0 clk in1 gnd! nhp w=64u l=0.18u as=30.72p ad=17.28p ps=0.129920m
pd=65.08u
+ nrs=30m nrd=16.8750m m=1 sa=0.48u sb=0.48u sd=0.54u nf=2
xm16 net46 clk net51 net51 php w=0.256000m l=0.18u as=82.56p ad=69.12p
ps=0.325160m
+ pd=0.260320m nrs=80.6250m nrd=67.50m m=1 sa=0.48u sb=0.48u sd=0.54u nf=8
xm3 out0 net46 in1 net51 php w=64u l=0.18u as=30.72p ad=17.28p ps=0.129920m
pd=65.08u
+ nrs=30m nrd=16.8750m m=1 sa=0.48u sb=0.48u sd=0.54u nf=2
xm2 out0 clk in net51 php w=64u l=0.18u as=30.72p ad=17.28p ps=0.129920m
pd=65.08u
+ nrs=30m nrd=16.8750m m=1 sa=0.48u sb=0.48u sd=0.54u nf=2
v7 net51 gnd! dc=3.3
.ends mux0
```

```
******************************************************************************
* Library          : Rectifier_Compile
* Cell             : RF_input
* View             : schematic
* View Search List : schematic symbol hspice
* View Stop List   : symbol
******************************************************************************
.subckt rf_input out outn
v5 net28 net57 dc=0 sin ( 0.8 3.3 865meg 1n 0 0 )
*v5 net28 net57 dc=0 sin ( 0.8 3.3 0.0865meg 1n 0 0 )
*v5 net28 net57 dc=0 vmrf amp=3.0 freq=865e3 phase=0 filcoef=0.95 rate=1e6
mod=qpsk
*+  filter=cos bitstream=ffff0000ffff000000000000h
v3 clk gnd! dc=3.3 pulse ( 3.3 0.2 56u 1n 1n 96u 192u )
v10 net41 gnd! pulse ( 3.3 0.2 70u 1n 1n 96u 10368u )
*v10 net41 gnd! pulse ( 3.3 0.2 310u 1n 1n 96u 576u )
l16 net58 net57 l=100f
x4 clk net28 gnd! out0p mux0
xi12 clk net58 gnd! out0n mux0
xi11 net41 out0n gnd! outn mux0
xi9 net41 out0p gnd! out mux0
.ends rf_input

.subckt rf_input01 out outn
v5 net28 net57 dc=0 sin ( 0.8 1.3 8.65meg 1n 0 0 )
*v5 net28 net57 dc=0 vmrf amp=3.0 freq=865e3 phase=0 filcoef=0.95 rate=1e6
mod=qpsk
*+  filter=cos bitstream=ffff0000ffff000000000000h
v3 clk gnd! dc=3.3 pulse ( 3.3 0.2 2u 1n 1n 96u 192u )
v10 net41 gnd! pulse ( 1.3 0.2 2u 1n 1n 96u 576u )
l16 net58 net57 l=100f
x4 clk net28 gnd! out0p mux0
xi12 clk net58 gnd! out0n mux0
xi11 net41 out0n gnd! outn mux0
xi9 net41 out0p gnd! out mux0
.ends rf_input01
******************************************************************************
* Library          : EHS
* Cell             : EHS_tb
* View             : schematic
* View Search List : schematic symbol hspice
* View Stop List   : symbol
******************************************************************************
```

```
.tran 1u 250u start=0u
*.tran 100n 450u start=0u
.option opfile=1 split_dp=1
.option probe=1

.probe tran v(pmu_top.ehs.piezo_inn) v(pmu_top.ehs.piezo_inp)
v(pmu_top.ehs.primary_storage) v(pmu_top.ehs.vref1) v(pmu_top.ehs.vref2)
v(pmu_top.ehs.vref3) v(pmu_top.ehs.vreg)
.probe tran v(pmu_top.ehs.rf_inn) v(pmu_top.ehs.rf_inp) v(pmu_top.ehs.rf_inp)
*power
.probe tran v(pmu_top.ehs.rf_inn)+v(pmu_top.ehs.rf_inp)
.probe tran (v(pmu_top.ehs.rf_inn)+v(pmu_top.ehs.rf_inp))*(pmu_top.ehs.r864)
.probe tran v(pmu_top.ehs.piezo_inn)+v(pmu_top.ehs.piezo_inp)
*power after rectification
.probe tran v(pmu_top.ehs.vsec_storage)*i(pmu_top.ehs.r1512)
.probe tran abs(v(pmu_top.ehs.vsec_storage_rf)*(i(pmu_top.ehs.r866)))
.probe v(pmu_top.ehs.piezo_inn)*abs((i(pmu_top.ehs.r479)+i(pmu_top.ehs.r478)))
.probe
abs((v(pmu_top.ehs.rf_inn)*abs((i(pmu_top.ehs.r834))))+(v(pmu_top.ehs.rf_inp)*abs(
i(pmu_top.ehs.r413))))

*current from rectifier
.probe abs(i(pmu_top.ehs.r479)+i(pmu_top.ehs.r478))
.probe i(pmu_top.ehs.r449) i(pmu_top.ehs.r448) i(pmu_top.ehs.r479)
i(pmu_top.ehs.r478)
.probe i(pmu_top.ehs.r834) i(pmu_top.ehs.r833) i(pmu_top.ehs.r414)
i(pmu_top.ehs.r413)
.probe i(pmu_top.ehs.r866) i(pmu_top.ehs.r865) i(pmu_top.ehs.r863)
i(pmu_top.ehs.r862)


.probe tran v(pmu_top.ehs.vsec_storage) v(pmu_top.ehs.vsec_storage_rf)
.probe tran i(pmu_top.ehs.r1512) i(pmu_top.ehs.r864)

.probe tran abs(v(pmu_top.ehs.m21|drn)*i(pmu_top.ehs.m21))
.probe tran abs(v(pmu_top.ehs.m22|drn)*i(pmu_top.ehs.m22))
.probe tran abs(v(pmu_top.ehs.m23|drn)*i(pmu_top.ehs.m23))
.end

//
// Milkyway Hierarchical Verilog Dump:
// Generated on 04/21/2020 at 14:29:02
```

```
// Design Generated by Cell Based Verilog Reader
// File produced by Consolidated Verilog Writer
// Library Name :MW_PRTOJECT_LIB
// Cell Name    :pmu
// Hierarchy delimiter:'/'
// Write Command : write_verilog -pg ../netlist/pmu_pg.v
//


module pmu (ref1 , ref2 , ref3 , l1_done , l2_done , l3_done ,
    load1_cont , load2_cont , load3_cont , VDD , VSS);
input  ref1 ;
input  ref2 ;
input  ref3 ;
input  l1_done ;
input  l2_done ;
input  l3_done ;
input VDD;
input VSS;
output load1_cont ;
output load2_cont ;
output load3_cont ;

//Digital block
//supply1 VDD ;
//supply0 VSS ;
INVX0 U212 (.ZN ( load1_cont ) , .VDD ( VDD ) , .INP ( load1_con ) , .VSS ( VSS ) ) ;
INVX0 U213 (.ZN ( load2_cont ) , .VDD ( VDD ) , .INP ( load2_con ) , .VSS ( VSS ) ) ;
INVX0 U214 (.ZN ( load3_cont ) , .VDD ( VDD ) , .INP ( load3_con ) , .VSS ( VSS ) ) ;

AND3X1 U22 (.IN2 ( ref1 ) , .IN1 ( ref2 ) , .IN3 ( ref3 ) , .Q ( ldo_sw_reg )
    , .VDD ( VDD ) , .VSS ( VSS ) ) ;
INVX0 U21 (.ZN ( n1 ) , .VDD ( VDD ) , .INP ( l1_done ) , .VSS ( VSS ) ) ;
INVX0 U20 (.ZN ( n3 ) , .VDD ( VDD ) , .INP ( l3_done ) , .VSS ( VSS ) ) ;
NAND4X0 U19 (.IN1 ( ldo_sw_reg ) , .QN ( load1_con ) , .IN2 ( n1 )
    , .VSS ( VSS ) , .VDD ( VDD ) , .IN3 ( n3 ) , .IN4 ( n8 ) ) ;
INVX0 U18 (.ZN ( n2 ) , .VDD ( VDD ) , .INP ( l2_done ) , .VSS ( VSS ) ) ;
NAND2X0 U17 (.VDD ( VDD ) , .IN1 ( n1 ) , .VSS ( VSS ) , .IN2 ( n8 )
    , .QN ( n11 ) ) ;
AND3X1 U16 (.IN2 ( n3 ) , .IN1 ( n11 ) , .IN3 ( ldo_sw_reg ) , .Q ( n10 )
    , .VDD ( VDD ) , .VSS ( VSS ) ) ;
NAND3X0 U15 (.VDD ( VDD ) , .VSS ( VSS ) , .QN ( load2_con ) , .IN3 ( n10 )
    , .IN2 ( n7 ) , .IN1 ( n2 ) ) ;
```

```
NAND2X0 U14 (.VDD ( VDD ) , .IN1 ( n2 ) , .VSS ( VSS ) , .IN2 ( n7 ) , .QN ( n9 ) ) ;
NAND3X0 U13 (.VDD ( VDD ) , .VSS ( VSS ) , .QN ( load3_con ) , .IN3 ( n10 )
    , .IN2 ( l3a ) , .IN1 ( n9 ) ) ;
DFFARX1 l3a_reg (.VDD ( VDD ) , .VSS ( VSS ) , .CLK ( ldo_sw_reg )
    , .RSTB ( n3 ) , .Q ( l3a ) , .D ( VDD ) ) ;
DFFARX1 l2a_reg (.VDD ( VDD ) , .VSS ( VSS ) , .CLK ( ldo_sw_reg )
    , .RSTB ( n2 ) , .Q ( n7 ) , .D ( VDD ) ) ;
DFFARX1 l1a_reg (.VDD ( VDD ) , .VSS ( VSS ) , .CLK ( ldo_sw_reg )
    , .RSTB ( n1 ) , .Q ( n8 ) , .D ( VDD ) ) ;
endmodule
```
